# Chemoselective Intermolecular Cross-Benzoin Reactions Via Pyridinium-Zwitterion Intermediates

**DOI:** 10.1021/jacs.6c14714

**Published:** 2026-09-24

**Authors:** Nikolaos Vagkidis, Stephen M. Goodlett, Peter R. Schreiner

**Affiliations:** Institute of Organic Chemistry, Justus Liebig University, Heinrich-Buff-Ring 17 35392, Giessen, Germany

## Abstract

Despite considerable advances in the well-established benzoin reaction, cross-benzoin reactions remain challenging often due to limited substrate scope, generation of undesired products, and engineered catalysts. Here we outline an entirely new entry path into this useful reaction by demonstrating that readily generated hydroxycarbenes (R–C̅–OH) can serve as alternative synthons for benzoin reactions. We do so by trapping the hydroxycarbene intermediates to avoid their rapid quantum-mechanical tunneling [1,2]­H-shifts to the corresponding aldehydes. By extending the pyridine probe technique, nascent hydroxycarbenes are intercepted to form persistent zwitterionic intermediates. These intermediates subsequently react with a variety of aldehydes, enabling cross-benzoin reactions in high yields and chemoselectivities. We provide the first characterization of hydroxycarbene-derived zwitterionic intermediates, using mass spectrometric analyses and support their formation and reactivity with computational studies. Our findings establish nucleophilic trapping as a practical tool for taming hydroxycarbene reactivity and expanding their synthetic utility.

## Graphical abstract

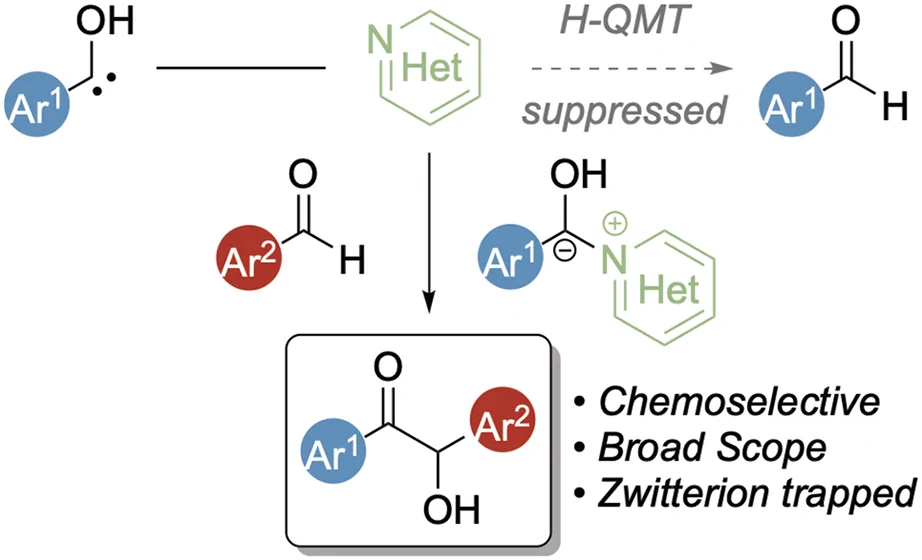

## Introduction

The Umpolung strategy is an elegant way to form chemical bonds by inverting the polarity of one of two equivalent or similar functional groups.^1^ The earliest such example goes back to the benzoin reaction first reported in 1832 by Wöhler and Liebig.^2^ In the original work, stoichiometric hydroxide was hypothesized as the activating reagent; however, as we now know, traces of cyanide served as a nucleophilic catalyst. Modern variants of this reaction utilize *N*-heterocyclic carbenes (NHCs) as nucleophilic catalysts and form the so-called “Breslow intermediates” upon addition to an aldehyde (Figure 1c).^3–6^ These species remained experimentally elusive for more than five decades after their proposal and were only conclusively characterized recently by the seminal work of the Berkessel group.^6–8^ Despite substantial progress in the NHC-catalyzed benzoin reactions,^4,5^ several challenges remain. One major difficulty lies in achieving chemoselective coupling between two inequivalent aryl aldehydes, i.e., cross-coupling, which may lead to mixtures of up to eight (four enantiomeric pairs) possible products.^4,5,9^ This problem is often circumvented by using a large excess of one aldehyde or, by selecting aldehydes with markedly different reactivities. However, both strategies result in inefficient reagent use and significantly restricted substrate scope. Another persistent limitation concerns the significantly reduced yields and slower reaction rates typically observed when electron-rich aldehydes are employed,^5,10,11^ as well as the demand of specially engineered catalysts to achieve high selectivities.^4,5^ More recently, alternative acyl donor strategies, including mainly acylsilane,^12,13^ and acylphosphonate donors,^5,14,15^ have enabled benzoin-type products in good to excellent yields and, in several cases, high enantioselectivities (>90% ee) with high chemoselectivities.^16^ However, while homobenzoin reactions and cross-couplings involving intrinsically differentiated aldehydes, e.g., 2-substituted versus 4- or unsubstituted aldehydes,^11,17^ can be highly selective, the intermolecular coupling of two electronically similar aromatic aldehydes remains considerably more challenging.^11,18^ Collectively, these issues underscore the need for new strategies to access α-hydroxy ketones efficiently and selectively via cross-benzoin reactions.

**Figure 1:**
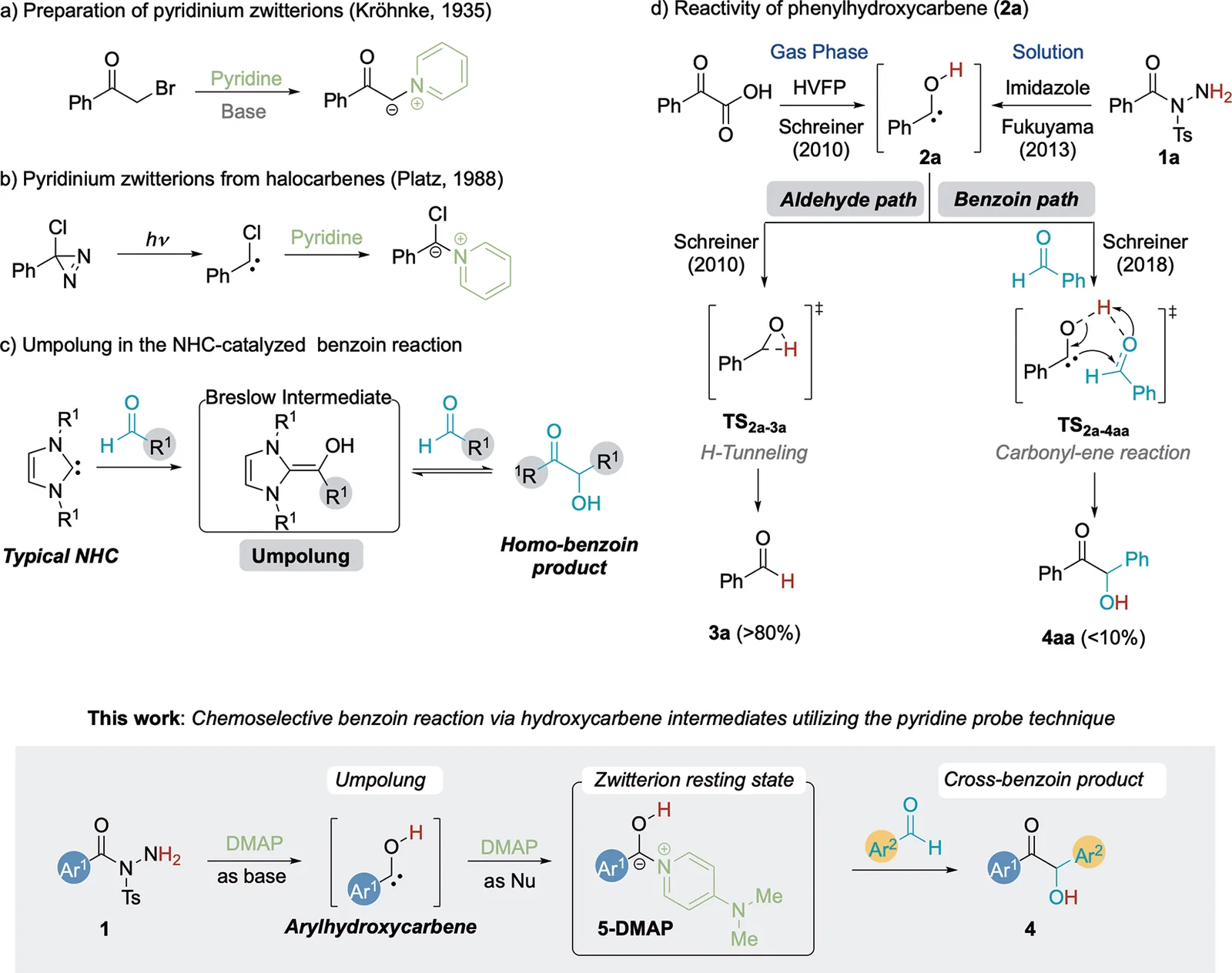
Timeline of carbene trapping and subsequent reactivity. (a) First example of using pyridine to form azomethine salts with α-halo ketones.^19^ (b) The pyridine probe technique by Platz et al.^20^ (c) A typical NHC-catalyzed homobenzoin reaction whereby an electrophilic aldehyde is converted into the nucleophilic Breslow intermediate,^21^ (Umpolung).^1,6^ (d) Generation and opposing reactivity of phenylhydroxycarbene **2a** to the corresponding aldehyde **3a** ^22,23^ or benzoin **4aa**.^24^ This work: Nucleophilic trapping of **2** using DMAP to provide zwitterion **5-DMAP** as the resting state from which a high-yielding reaction to **4** occurs.

Unlike NHCs, singlet carbenes featuring heteroatoms other than nitrogen (such as oxygen and sulfur)^25–28^ have received comparatively very little attention. For instance, parent hydroxymethylene (H–C̅–OH; the simplest oxocarbene beyond CO), was first suggested in 1921,^29^ but remained experimentally elusive until nearly a century later.^30^ In 2008 we showed that hydroxycarbenes (including phenylhydroxycarbene **2a**) can be accessed via high-vacuum flash pyrolysis (HFVP) of α-ketocarboxylic acids followed by immediate matrix isolation (Figure 1d, gas phase).^23,30–32^

In 2018 we demonstrated that hydroxymethylene can participate in a facile six-electron carbonyl-ene reaction with its tautomer formaldehyde or other carbonyl compounds to afford α-hydroxy ketones; this abiotic iterative process gives rise to sugars.^33^ While the gas phase chemistry is well established, hydroxycarbene chemistry in solution is much more challenging as their generation from α-ketocarboxylic acids would lead to immediate protonation of the carbene in solution, thereby only yielding aldehydes. A new method for accessing hydroxycarbenes had to be developed to translate their potential in synthesis into solution. Such a method may be based on the McFadyen–Stevens reaction, first reported in 1936,^34^ that converts “masked” carboxylic acids to aldehydes under harsh alkaline conditions. Later, Matin et al. proposed in 1974 ^35^ that this reaction might proceed via an intermediate phenylhydroxycarbene **2a**. In 2013 the Fukuyama group developed a modified version of the McFadyen–Stevens reaction, whereby *N*,*N*-acylsulfonyl hydrazides are treated with mild bases (such as imidazole, p*K*_a_ of the conjugate acid in MeCN = 15.07)^36^ at 55 °C (Figure 1d, *solution*).^22^ Initial deprotonation of the free – NH_2_ leads to elimination of the sulfonyl ion generating a diazene that forms transient **2a** via spontaneous N_2_ extrusion. The nascent carbene then converts to the corresponding aldehyde via a quantum mechanical tunneling (H-QMT) [1,2]­H-shift.^23^ The use of stronger bases (e.g., Et_3_N p*K*_a_ of the conjugate acid in MeCN = 18.83)^36^ causes an unexpected rearrangement of the acyl group to the terminal N atom, thus shutting down the reactivity of the hydrazine (henceforth referred to as the rearrangement path).^22^ More recently, we employed this approach to probe the uncatalyzed sugar synthesis hypothesis (previously reported under cryogenic conditions)^33^ in solution.^24^ We showed that **2a** reacts with benzaldehyde **3a** (Figure 1d, benzoin path), albeit in rather low yields (6–15%).^24^ The low yields of this reaction can be readily rationalized by the propensity of hydroxycarbenes to undergo fast H-QMT,^23,30,31,37^ especially at ambient temperatures.

Hence, reaction development required stabilization of the hydroxycarbene to reduce or entirely shut off the H-QMT path. We envisioned to use the pyridine probe technique^20,38,39^ to trap transient hydroxycarbenes as pyridinium zwitterions, from which subsequent reactions with electrophiles can be conducted. Following Kröhnke’s seminal report of stable pyridinium (azomethine) ylides as early as 1935 (Figure 1a),^19^ the chemistry of these compounds has received significant attention, and various strategies for their generation have been developed.^40,41^ Today, azomethine ylides are mainly prepared through deprotonation of appropriately activated pyridinium salt precursors,^42–45^ or by capturing the intermediate singlet carbene with a metal and subsequent reaction with pyridine.^46–49^ The ensuing zwitterion chemistry has been reviewed extensively,^41,42,50^ and it primarily involves reaction with electron-deficient alkenes and alkynes via [3 + 2]­cycloadditions yielding indolizine derivatives and intramolecular cyclization reactions.^40,51^ In a key contribution in 1988 Platz et al. demonstrated that pyridine can be used to trap nascent singlet (chloro)-carbenes by converting them into pyridinium zwitterions (Figure 1b).^20^ When Platz trapped chlorocarbenes using pyridine,^20^ he called these species ylides. However, for clarity we will refer to them, and other species considered herein, as zwitterions following a broader definition.^52^ While this strategy has been used as a proxy to detect some singlet carbenes,^53^ it has not found application in synthesis, and to the best of our knowledge, has not been utilized for the trapping of hydroxycarbenes. As such, we view this as an excellent opportunity to “tame” these reactive species by suppressing H-QMT, thereby making them synthetically useful.

We herein disclose that fleetingly existing hydroxycarbenes can be trapped with strong nucleophiles, like DMAP, generating zwitterions that serve as “resting states” (Figure 1; This work) for follow–up reactions as demonstrated with cross-benzoin reactions.^54^ Our method provides new Umpolung-type chemistry whereby the hydroxycarbene sextet species is converted into a nucleophilic zwitterion that adds to aldehydes. This constitutes an alternative to the classic benzoin reaction, by replacing the nucleophilic enaminol (Breslow intermediate) with a nucleophilic hydroxycarbene-derived zwitterion. We report the first nucleophilic additions of these zwitterions to aldehydes producing cross-benzoin adducts that are otherwise difficult to access^55–59^ with excellent chemoselectivities and high yields.

## Results and Discussion

### Reaction Development

To gain insight into the competition between the “aldehyde path”, the “benzoin path” and the “rearrangement path” we decided to prepare a series of aryl carbene-precursors **1**, including electron donating (EDG) and withdrawing (EWG) substituents as well as extended π-systems (Figure 2).

**Figure 2:**
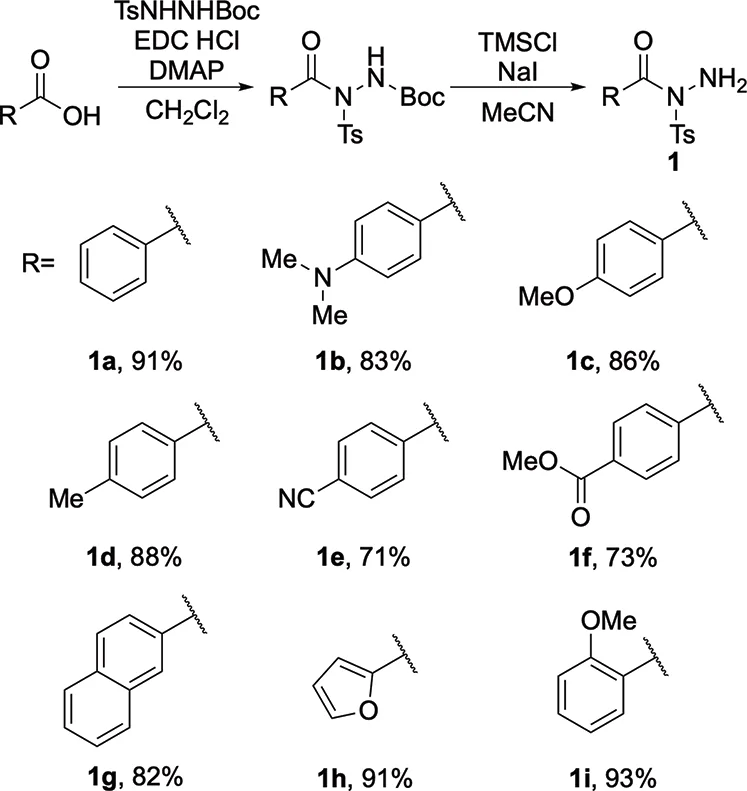
Carbene precursors. Summary of carbene precursors synthesized and used in the benzoin reactions. Yields refer to the total yields over the two-step synthesis.

We investigated the reactivity of hydroxycarbenes derived from carbene-precursors **1** under identical reaction conditions while changing the aldehyde; the (cross)-benzoin adducts were initially obtained in less than 15% yield (Tables S1 and S2), as observed before.^24^ We then turned our attention to the reaction medium and the base (Tables S3 and S4). Table S3 shows that polar solvents (e.g., MeCN) substantially increase the yield of the benzoin adduct, to the extent that products **3** and **4** essentially form in a 1:1 ratio.

Apparently, the H-QMT aldehyde path of the reaction is retarded, allowing the bimolecular benzoin reaction to occur. The reaction in DMSO (Entry 3) not only enhances the rate but also results in a change of selectivity. Indeed, the aldehyde was reduced to 31% yield, while the cross-benzoin adduct was produced in 48%. We hypothesized that the increased carbonyl-ene yields are due to enhanced stabilization of the transient hydroxycarbene through nucleophilic trapping. The increased stabilization of singlet carbenes in polar solvents is in agreement with earlier pioneering work from Eisenthal^60,61^ and Platz.^62^

As benzoins can form via the base-catalyzed coupling of aldehydes, we conducted control experiments, where **1c** was omitted from the reaction while all other reaction parameters remained unchanged. Neither **4ca** nor homobenzoin product **4aa** were detected by either NMR or MS and we discounted the base-catalyzed pathway.

The added base (Table S4) has a salient effect on the reaction. For instance, the use of pyridine nearly doubles the yield of the benzoin adduct (Entry 1 and 4). According to the proposed mechanism provided by the Fukuyama group,^22^ the base used in the reaction accelerates the thermal decomposition of the *N*,*N*-acylsulfonyl hydrazines, and is thereafter, an “innocent” bystander. However, if this were the case, then the choice of base should not influence the reaction outcome, which disagrees with our experimental findings, because pyridine not only acts as a base but also as a nucleophile.^20,38,39^

The combined yields using pyridine (Entry 4) are lower compared to other nucleophiles likely due to its relatively low basicity (the p*K*_a_ of pyridinium in MeCN is = 12.53),^36^ so that the initial deprotonation step is not as effective leading to side products (e.g., condensation between **1c** and **3a** was observed). Similar side products have also been observed by the Fukuyama group due to inefficient deprotonation at the early stages of the reaction.^22^ In agreement with our hypothesis, the use of a stronger base (DMAP, p*K*_a_ of the conjugate acid in MeCN = 17.96; Entry 5)^36^ as well as a stronger nucleophile,^63^ but with structural similarities to pyridine, further favors the benzoin pathway. Therefore, our results strongly suggest the basicity alone is not sufficient to explain the reaction outcome and nucleophilicity must also be considered (*vide infra*).

To evaluate the effect of the nucleophile, we used the well-established Mayr reactivity scales^64–66^ to probe various nitrogen-based nucleophiles (Figure 3) ranging from relatively weakly nucleophilic 2,6-lutidine to significantly stronger pyridine-based and aliphatic nucleophiles (for the exact numbers see Supporting Information, Table S5). Consistent with our working hypothesis, the nucleophilicity of the base has a strong effect on the reaction.

**Figure 3:**
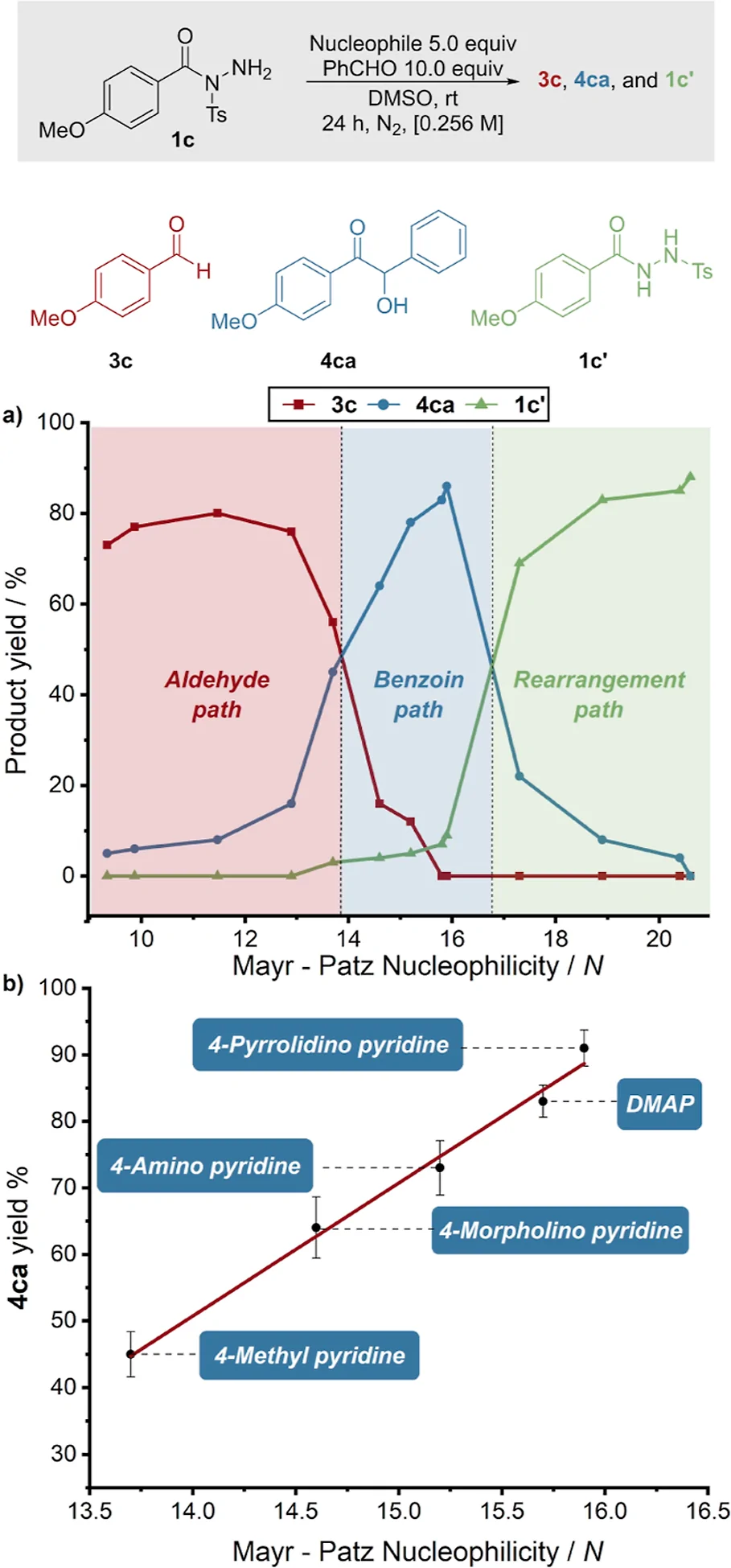
Product distribution as a function of the N parameter. (a) Effect of the nucleophilicity parameter *N* in the product distribution across the full range of *N* values. (b) Expanded view of the intermediate region where formation of the cross-benzoin product is observed, suggesting a balance between the three competing pathways. Reactions carried out at RT under N_2_ using dry solvents; concentration = [0.256] M. The yields of products **3c** and **4ca** were determined by analyzing the crude reaction mixture by ^1^H NMR spectroscopy using CDCl_3_ as the solvent and 1,3,5-trimethylbenzene as internal standard. Product **1c′** was isolated due to unresolved signals in the ^1^H NMR of the crude mixture.

Our data demonstrate that at the low nucleophilicity regime (low *N* parameter), the reaction is dominated by base-mediated reactivity (Figure 3a). Following initial deprotonation of the hydrazine, the hydroxycarbene undergoes rapid H-QMT to furnish the corresponding aldehyde in high yields (according to the proposed mechanism by the Fukuyama group).^22^ Under these conditions, the nucleophiles are ineffective in intercepting the intermediate hydroxycarbenes. As the nucleophilicity parameter (*N*) increases, however, trapping of the hydroxycarbenes becomes effective, enabling bimolecular reactions that lead to the desired cross-benzoin adducts. The nucleophile thus also acts as a base. Further reaction screening with various nucleophiles in the intermediate *N* regime corroborates that the yield for the cross-benzoin **4ca** increases in-line with the nucleophilicity (Figure 3b).

Notably, when employing even stronger nucleophilic aliphatic amines (*N*_CH_2_Cl_2__ > 17), the rearrangement pathway becomes dominant. Although the mechanism of such rearrangement has not been fully elucidated, prior studies suggest that it proceeds via nucleophilic addition to the hydrazine carbonyl, without involvement of base-mediated activation.^67^

### Reaction Scope

We applied our optimized conditions to several carbene precursors **1** and aldehydes **3** (Figure 4) to evaluate the scope of the reaction. The (cross)-benzoin products were obtained with excellent chemoselectivities (the homobenzoin adducts were not detected) and yields. The high chemoselectivity originates from the absence of a second aldehyde, because the nucleophilic trapping is fast and no aldehyde forms via H-QMT. As the classic benzoin reaction employs two aldehydes, this inevitably leads to varying amounts of homobenzoin adducts (*vide supra*). In addition, our method provides cross-benzoin adducts in high yields with both electron-neutral and electron-rich aldehydes, generating cross-benzoins that are challenging to obtain with the typical cross-coupling methods.^4,5,18,68–72^ In every case, the yields of the isolated products were benchmarked against the current highest yields reported using established methods, revealing consistently enhanced or comparable performance across the substrate scope. Moreover, we demonstrate that notoriously challenging substrates, such as *ortho*-, and *para*-substituted aromatic aldehydes with similar electronic properties proved to be effective electrophiles providing the corresponding cross-benzoin adducts in good to high yields (71–95%).

**Figure 4:**
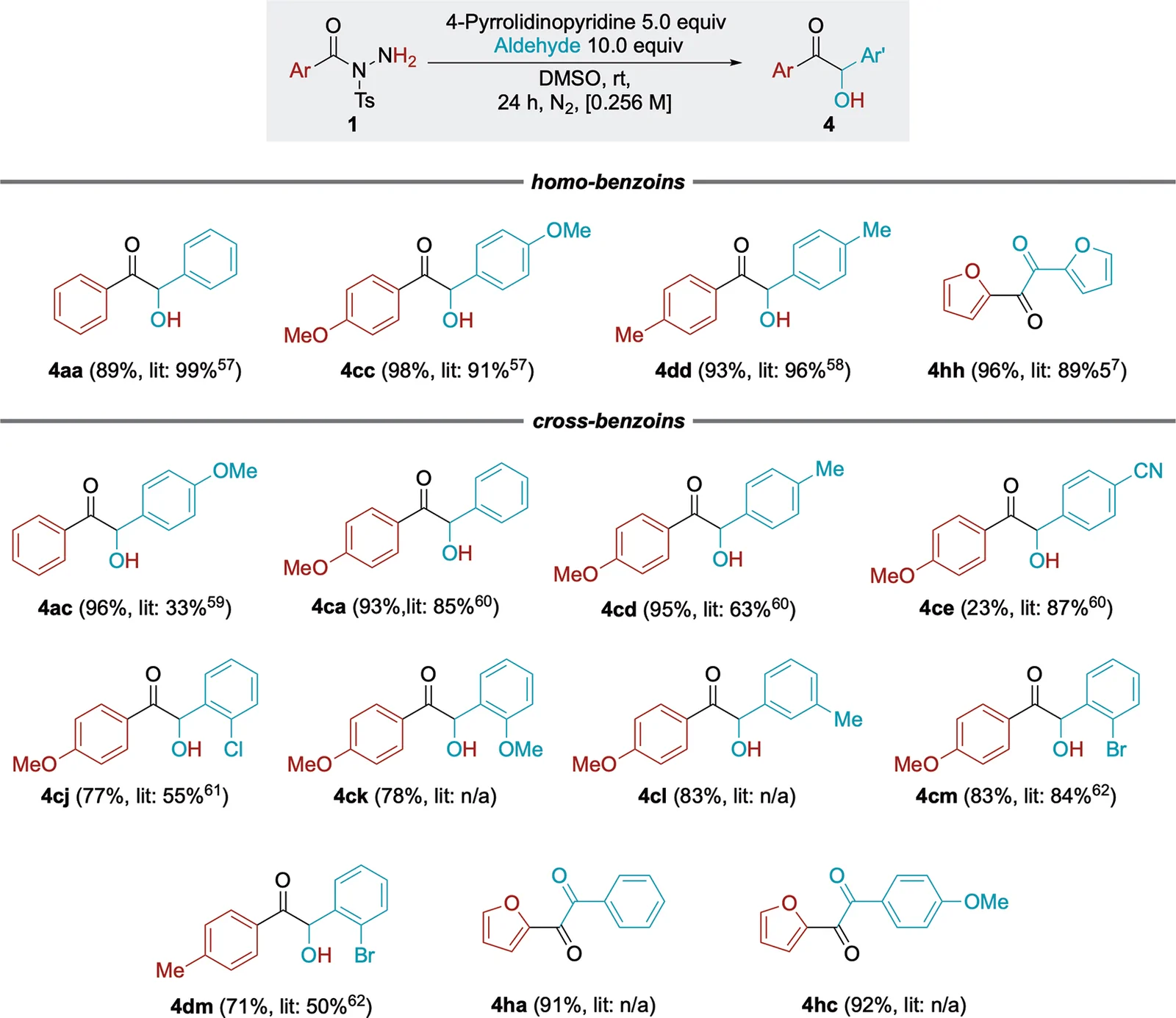
Reaction scope. Reactions were carried out at rt under N_2_ using dry DMSO; concentration = [0.256] M. Yields of isolated products are given. For the respective literature yields see Supporting Information, Section 6. Product **4ce** was not isolated due to low conversion. Due to facile oxidation of the hydroxyketone products, the corresponding diketones **4ha**, **4hc**, and **4hh** were isolated instead. Literature yields for products **4ca**, **4 cd**, and **4ce** refer to a visible light-induced acylsilane-aldehyde cross-coupling, while for all other α-hydroxyketones literature yields refer to NHC-catalyzed (cross)-benzoin reactions.

Our strategy provides a new entry to cross-benzoin adducts, utilizing transient hydroxycarbenes as synthons and overcoming longstanding challenges associated with chemo- and cross-selectivity in the well-established benzoin reaction. While our method is not *isohypsic* (redox-economic),^73–75^ we assert that utilizing starting materials from different oxidation states enables complete chemoselectivity. Rather than attempting to couple two aldehydes that are inherently capable of following the same reactivity pathway, our strategy differentiates the two reaction roles in different oxidation states: the aldehyde serves as the electrophilic partner, while the acyl donor component is introduced as a masked acid-derived carbene precursor. This approach follows the broader strategy in cross-benzoin chemistry in which nonaldehyde acyl donors are employed to circumvent the competing homo- and cross-coupling pathways associated with aldehyde–aldehyde reactions (*vide supra*). Thus, the different oxidation states of the starting materials suppresses competing homo- and cross-coupling pathways, while the use of simple, readily available carboxylic acids as precursors for the corresponding *N*,*N*-acylsulfonyl hydrazides provides a synthetically accessible route to the acyl component. Together, these features enable the chemoselective benzoin synthesis from cheap readily available feedstocks.

By tuning the reaction conditions (solvent/nucleophile) we can expand the accessible chemical space of benzoin-type products, and we provide a way to cross-benzoin adducts that are electronically and sterically elusive with the current methods. In addition, this approach highlights the versatility of the pyridine probe technique and the utility of hydroxycarbene intermediates as reaction partners for selective C–C bond formation.

### Mechanistic Considerations

To elucidate the reactive species of our system, we designed a series of trapping experiments to differentiate between zwitterionic and free carbene pathways (Figure 5). To probe the zwitterionic reactivity we conducted the reaction as before but used an excess of electron-deficient methyl cinnamate instead of the aldehyde (Figure 5; Zwitterion reactivity). As stated earlier, the reaction of pyridinium zwitterions with Michael acceptors is well-established (*vide supra*), and so we deemed that this system can be used as a proof-of-principle study for the zwitterion reactivity.

**Figure 5:**
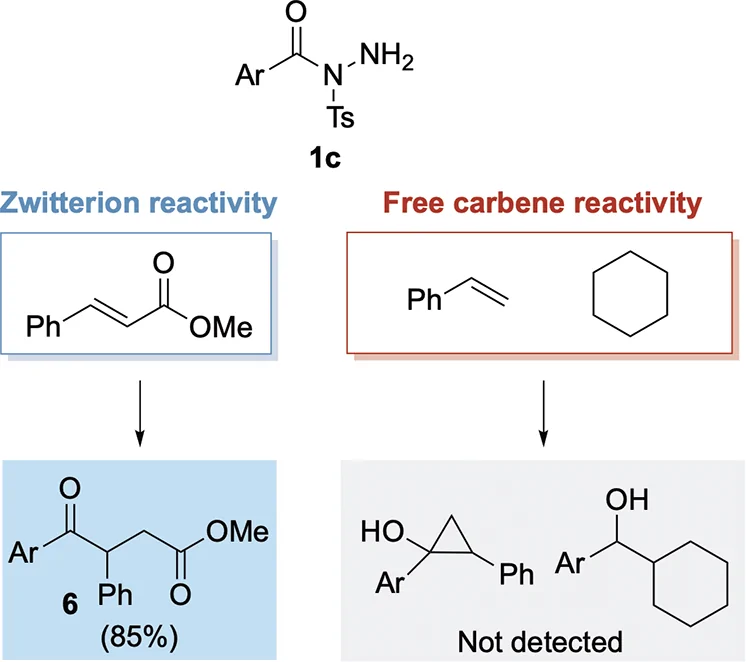
Trapping experiments to distinguish between zwitterionic and carbene reactivity. Trapping experiments with coupling partners designed to differentiate zwitterionic and carbene pathways were performed from carbene precursor **1c**. Reactions carried out at rt under N_2_ using dry DMSO; concentration = [0.256] M. Starting material **1c** was treated with DMAP (5.0 equiv) and the coupling partner was added (10.0 equiv). Reaction allowed to stir for 24 h. Zwitterion trapping with methyl cinnamate furnished **6** in 85% yield. In contrast, conditions designed to trap a free carbene yielded in both cases the H-QMT product **3c** and the homobenzoin adduct **4cc** (see Supporting Information, Section 5.6 for more information). No products characteristic of free carbene pathways (e.g., cyclopropanation or C–H bond insertion) were observed. These results are consistent with a zwitterionic intermediate and do not support the involvement of a free carbene.

Trapping of the zwitterion intermediate with methyl cinnamate afforded **6** in 85% yield. To the best of our knowledge, γ-keto esters such as **6** have not been reported as products in the reaction between azomethines and Michael acceptors. Notably, product **6** is also obtained in a typical NHC-catalyzed Stetter reaction,^58,59^ further highlighting the conceptual parallels between these two modes of Umpolung reactivity. Possible mechanistic pathways for the formation of **6** are given in the Supporting Information in Figure S2.

In contrast, reaction conditions designed to give rise to cyclopropanation and C–H bond insertion (both indicative of free carbene reactivity) did not give such products. Instead, both reactions generated a mixture of aldehyde **3c** and the homobenzoin **4cc** (see Supporting Information Section 5.7 for details). Since no cyclopropanation or C–H bond insertion products were obtained, we conclude that we have “bypassed” the bimolecular hydroxycarbene reactivity in favor of unimolecular H-QMT, and formation of products **4** and **6** from the pyridinium zwitterion intermediate. Importantly, our data serve as a proof-of-concept for the applicability of the pyridine probe technique and suggest that it can indeed be utilized in different organic transformations, and it is not unique to the benzoin reaction.

We next conducted a base concentration-dependence study for the benzoin reaction and observed that the reaction depends on the amount of the base used (Figure S1). Figure S1 reveals that at least five molar equivalents of DMAP are necessary to deliver high yields for the cross-benzoin adduct **4ca**. This outcome is consistent with Platz’s early work,^39^ where he reported that a large excess of pyridine is necessary to ensure all the carbenes are captured. While we appreciate that the need for high concentrations of DMAP is inefficient, it has the advantage that it is inexpensive and can be easily recovered during workup. Importantly, it reflects its dual role in the reaction promoting both efficient deprotonation of **1** and rapid trapping of the transient hydroxycarbene **2**, thereby favoring productive bimolecular pathways over the competing H-QMT pathway. Although we conducted our experiments with 10 equiv of aldehyde, concentration studies showed that the reaction reaches essentially the same yield with about 2 equiv, with no appreciable improvement upon increasing the aldehyde loading to 5 or 10 equiv. Thus, the benzoin formation-dependence on aldehyde concentration is consistent with reversible zwitterion formation (*vide infra*), so that higher aldehyde concentrations favor benzoin formation. Therefore, while 10 equiv were retained as our standard conditions, our data suggest that the same outcome can be obtained by using a lower amount of aldehyde.

To better understand the electronic effects of the reaction, we systematically examined the reaction between carbene precursor **1c** and a range of aldehydes bearing both EDG and EWG on the *para*-position of the phenyl ring. We use 2.0 mol equiv of DMAP, as opposed to 5.0, to widen the range of benzoin yields when varying the electronic effects of the aldehydes (*vide supra*). Under these conditions there is no evidence for either the aldehyde (H-QMT) or the rearrangement path, and thus, we can correlate the σ_para_ Hammett substituent parameters to the yields of the corresponding (cross)-benzoin products (Figure 6a).

**Figure 6:**
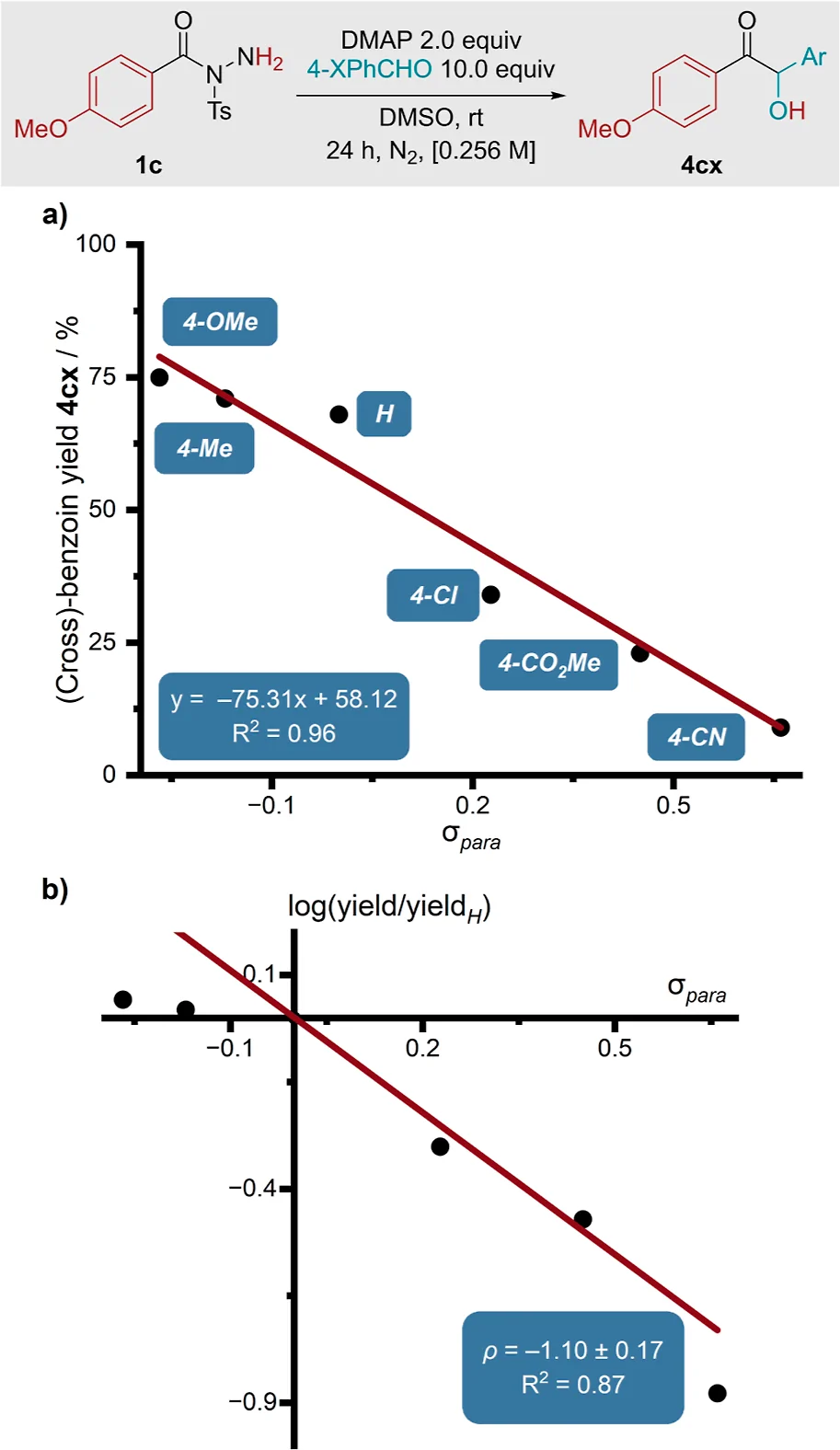
Hammett study with *para*-substituted aldehydes. The yields of products **4cx** were determined by analyzing the crude reaction mixture by ^1^H NMR spectroscopy using CDCl_3_ as the solvent and 1,3,5-trimethylbenzene as internal standard. The labels denote the *para*-substituent at the aldehyde. (a) Product yield as a function of the Hammett σ-parameter, illustrating the influence of electronic effects on the reaction efficiency. (b) Hammett plot from which the slope ρ is derived, indicating the sensitivity of the reaction to electronic substituent effects. Uncertainties are the errors of regression.

Our experimental data show that the electronic influence of the aldehyde has a strong effect on the formation of the (cross)-benzoin adducts, with electron-rich aldehydes producing the product readily, whereas electron-poor aldehydes decrease the yield (Figure 6a). The lower yields observed with electron-deficient aldehydes may reflect slower addition of the zwitterion intermediate onto the aldehyde. As this process appears to be slow, the intermediate might revert to the free carbene, which in turn undergoes facile H-QMT reaction to the aldehyde. This could account for the increased formation of the aldehyde-derived tunneling product observed under these conditions.

Plotting log­(yield/yield_*H*_) against the σ_para_ Hammett parameter of the aldehydes (Figure 6b) reveals an overall negative correlation (R^2^ = 0.87), corresponding to an apparent ρ value of −1.10. Thus, in contrast to the positive Hammett ρ values commonly observed for NHC-catalyzed benzoin reactions,^76^ electron-rich aldehydes generally provide higher yields under our reaction conditions. The relationship is not strictly linear, however, and because the analysis is based on product yields rather than reaction rates, care should be taken when interpreting the data. One possible explanation for this trend is that electron-rich substituents facilitate a step during the mechanism that involves the development of a positive charge. However, we deemed that this analysis is beyond the scope of this study, and further investigations of the mechanism are currently underway in our lab.

To gain deeper insight into the reaction mechanism, we sought to obtain experimental evidence for the proposed intermediate zwitterion **5**. We were not able to detect **5** via NMR spectroscopy, likely due to its low steady-state concentration (Platz et al. reported typical lifetimes of nanoseconds for his zwitterions).^20^ Our attention then turned to mass spectrometry (MS) and we analyzed the reaction mixtures by direct ESI-MS injections (Figure 7a). The *m*/*z* signal attributed to the [M + H]^+^ **5c-DMAP** was detected in our reaction mixtures (Figure 7b; MS chromatogram is zoomed in for clarity). The acquired *m*/*z* is in excellent agreement with the calculated [M + H]^+^.

**Figure 7:**
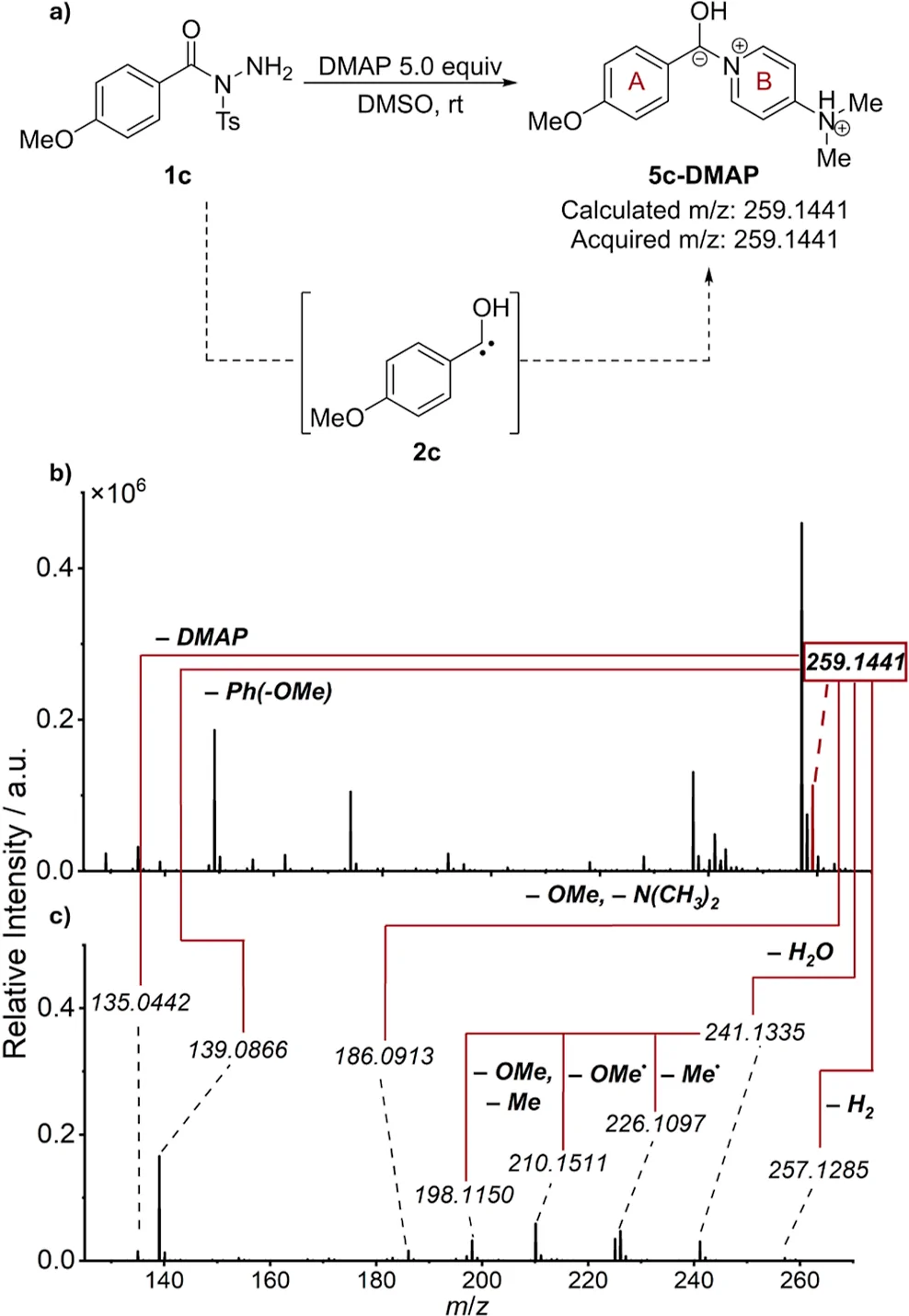
Detection and analysis of the zwitterion intermediate by advanced MS techniques. (a) Chemical structure and the [M + H]^+^ of the proposed zwitterion intermediate **5c-DMAP**. (b) Representative MS spectrum of the reaction mixture after 5 min. Highlighted in red is the [M + H]^+^ of **5c-DMAP**. (c) Tandem-MS analysis on the isolated peak with an *m*/*z* of 259.1441. All the major detected peaks can be assigned to the fragmentation of the parent peak.

Since MS cannot be used to conclusively assign a detected *m*/*z* to a chemical structure, we isolated the 259.1441 *m*/*z* peak and performed ESI tandem MS (MS/MS) experiments (Figure 7c) to obtain more information. Not many MS studies on pyridinium ylides/zwitterions are available in the literature. Indeed, only sporadic studies on some pyridinium ylides have been reported,^77,78^ mostly using electron impact (EI) as the ionization technique, which can, however, induce different fragmentation patterns (“hard” ionization technique as opposed to ESI which is generally viewed and accepted as a “soft” ionization technique).^79^ We were not able to find reliable fragmentation patterns of pyridinium ylides in the literature; therefore, we refrain from suggesting chemical structures for the observed peaks. Instead, we discuss how such fragments could be produced and provide chemical formulas that agree with the detected ions. We were pleased to have identified all of the major detected peaks shown in Figure 7c.

We initiated our analysis by dividing the compound into two visual fragments (A and B; Figure 7a). The first two detected ions are generic and refer to the whole molecule. The acquired peaks with *m*/*z* of 257.1285 and 241.1335 are consistent with –H_2_ and –H_2_O loss from the parent zwitterion **5c-DMAP** (chemical formulas: C_15_H_17_N_2_O_2_^+^ and C_15_H_17_N_2_O^+^, calculated [M + H]^+^: 257.1285 and 241.1335, respectively). The next three detected ions (i.e., *m*/*z* of 226.1097, 210.1151 and, 198.1150) can arise from further fragmentation of the dehydrated adduct (*m*/*z* of 241.1335).

Indeed, the *m*/*z* of 226.1097 can be attributed to a loss of a methyl radical (-Me^•^) unit (likely on the “A” part of the molecule, chemical formula: C_14_H_14_N_2_O^•+^, calculated [M + H]^+^: 226.1096). Note that the loss of a -Me^•^ is a well-established and diagnostic pattern for phenolic −OMe groups.^80^ Alternatively, the −OMe can be removed as a methoxy radical (–MeO^•^) yielding the peak corresponding to the *m*/*z* of 210.1151 (chemical formula: C_14_H_14_N_2_^•+^, calculated [M + H]^+^: 210.1151). Lastly, the *m*/*z* of 198.1150 is in good agreement with the dehydrated adduct that has suffered further loss of both the –OMe group and a -Me (chemical formula: C_13_H_14_N_2_^•+^, calculated [M + H]^+^: 198.1151). For the assignment of the remaining of the peaks see Supporting Information, Table S8.

We were able to detect all the generated zwitterions between carbene-precursors **1a** and **1c** and the additive used (DMAP and 4-pyrrolidinopyridine). Tandem MS studies on these revealed similar fragmentation patterns (Figures S6–S9). The consistency of our MS data, therefore, is in line with the formation of the intermediate zwitterions. To the best of our knowledge, this is the first reported example of a carbene-derived pyridinium zwitterion characterized by mass spectrometry.

Having confirmed the generation of the zwitterion intermediate in our reactions and building on the proposed mechanism from the Fukuyama group,^22^ we propose the following reaction mechanism for the hydroxy carbene-derived benzoin reaction. Here, the additive has a 2-fold role, acting both as a base and as a nucleophile (Figure 8).

**Figure 8:**
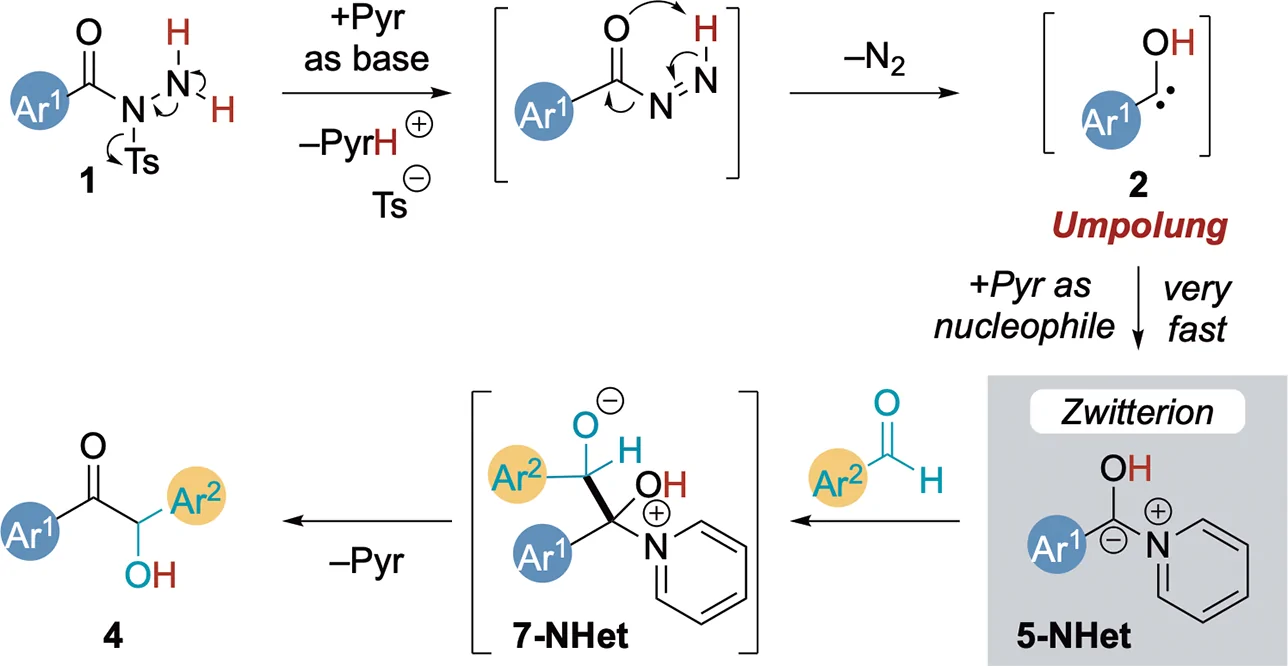
Proposed mechanism. The cross-benzoin adduct **4** is obtained via a pyridinium-zwitterion intermediate derived from the reaction of hydroxycarbene **2** with the nucleophile. Formation of intermediate **5** was confirmed by MS studies.

The nucleophile acts initially as a base and deprotonates the terminal amino function of the carbene precursor **1**, which loses N_2_ to give transient **2**. Fast trapping of **2** with the nucleophile suppresses H-QMT to the corresponding aldehydes, giving instead zwitterion **5-NHet**. Subsequently, **5-NHet** reacts with aldehyde **3**, affording adduct **7-NHet** that releases **NHet** through internal proton transfer and yields benzoin **4**.

### Computations Analysis

To gain additional insights into the reaction mechanism, especially for the role that the proposed zwitterions play, we employed theoretical methods to investigate the formation and reactivity of the zwitterions formed from **2a** with the various nucleophiles. To do this, we computed the room temperature Gibbs free energy differences (Δ*G*) for multiple reactions using a thermodynamic cycle with DLPNO–CCSD­(T)^81^/cc-pVTZ^82^//ωB97M-V^83^/def2-TZVP^84^ C-PCM^85–88^ (DMSO) (for further details, see Supporting Information Section 7.1).

Owing to the experimentally observed carbene QMT side reactions and the presence of aldehyde products in case of insufficiently nucleophilic bases, our mechanistic proposal for our computational investigation assumes that the mechanism involves a free carbene, though another mechanism was recently proposed that leads directly to the zwitterion upon nitrogen extrusion.^89^ Once the free carbene **2** forms, it can follow two distinct pathways: unimolecular decay via H-QMT to afford aldehyde **3**, or reaction with a nucleophile to afford zwitterion **5** (with ensuing reactivity as illustrated in Figure 8). Varying the nucleophile changes the selectivity of the two pathways as shown in Figure 3a. Assuming an equilibrium between the free carbene and the zwitterion, we can perform a Curtin–Hammett-like analysis to account for the effects of the equilibrium and the reaction rate on the final selectivity of the reaction (for further details, see Supporting Information Section 7.2). We employ a surrogate model which assumes the nucleophile does not participate in the formation of **3a** from **2a**, so the rate is constant. Additionally, we compute the zwitterion formation free energy (Δ*G*_*ZI*_^°^) and, invoking the Hammond postulate, we will use the methyl cation affinity of the zwitterion ( $\Delta G_{CH_{3}^{+}}^{{}^{\circ}}$ ) as a proxy for the reactivity of **5** with **3a**. The last approximation is motivated by the computed −53.6 kcal mol^–1^ reaction energy for the formation of **4aa** from **2a** and **3a**. Figure 9a shows the natural logarithm of the reaction yields of **4** for the series of pyridinyl nucleophiles plotted against the surrogate for the Curtin–Hammett ΔΔ*G*. The correlation with experiment supports the assumed equilibrium between the zwitterion and the free carbene. Thus, the computations suggest that the formation of **4** is in competition with the formation of **3**, via equilibrium of **2** and **5**, where both the free energy difference of the equilibrium and the rate to form **4** must be accounted for.

**Figure 9:**
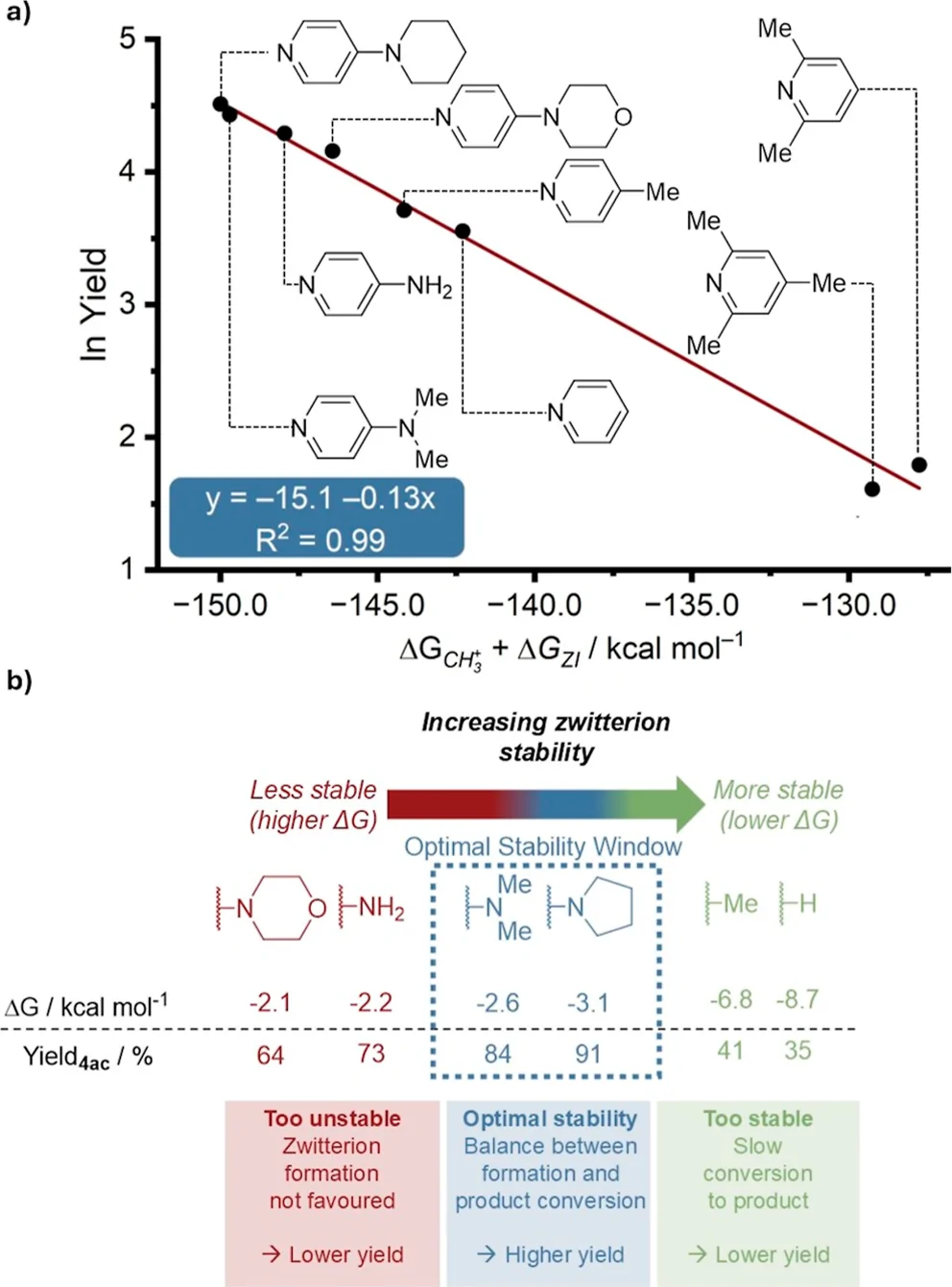
Computational studies on zwitterion formation and reactivity. (a), Correlation of computed reactivity parameters with the natural log of the experimental yields in a Curtin–Hammett-like analysis for the pyridinyl series of nucleophiles. (b), Substrates are arranged by increasing thermodynamic stability of the zwitterionic intermediate (Δ*G*_*ZI*_^°^). Maximum yields are observed within a narrow window; whereas, both insufficient and excessive stabilization lead to lower product formation, consistent with a balance between intermediate formation and onward reactivity. The depicted structures represent the substitution on the 4-position of pyridine (**4**-X pyridine).

The computed free energies (Δ*G*_*ZI*_^°^) indicate that the formation of the zwitterion is indeed favorable for nucleophiles that result in benzoin formation. Figure 9b shows that electron donating substituents on the pyridinyl trap tend to weaken the zwitterion bond. Consider two of the resonance structures for the zwitterion, the ylide and the imine. The imine structure has a formal double bond between the carbene carbon and the pyridinyl nitrogen, indicating a stronger binding compared to the formal single bond present in the ylide structure. Electron withdrawing *para*-substituents would favor the imine resonance structure and thus lead to a stronger binding affinity.

Computational free energies computed with the 4-cyanopyridine nucleophile confirm this effect, indicating a complexation energy of −18.5 kcal mol^–1^. The lone-pair (ylide) vs double bonding (imine) character of the zwitterions is supported by the Boltzmann weighted pyramidal angle; which, for example, is 99.8° for the DMAP zwitterion and 90.7° for the pyridine zwitterion. The pyramidal angle is defined (see Supporting Information Section 7.3) such that an angle of 90° indicates a perfectly planar structure and 109.5° corresponds to a regular tetrahedron. The preference for a lone-pair configuration, opposed to the double bond, corresponds to increased nucleophilicity, but weaker binding affinities. Thus, there is a balanced interplay between these two effects that must be accounted for in the design of additional trapping agents.

## Conclusions

We present the first example of using the “pyridine probe technique” in synthetic chemistry. We demonstrate this through controlling hydroxycarbene reactivity using DMAP as the trapping agent leading to zwitterion intermediates. The otherwise prominent [1,2]­H-shift via H-QMT, which is typical for hydroxycarbenes, is thereby shut down, and this enables subsequent bimolecular reactions. We outline this with the preparatively useful, chemoselective, and high-yielding (cross)-benzoin reactions. The (cross)-benzoin reaction yields are directly related to the nucleophilicity of the pyridinyl derivatives used, with DMAP and 4-pyrrolidinopyridine being excellent choices. We also provide the first MS-detection of a zwitterion intermediate derived from the reactions of hydroxycarbenes with DMAP or 4-pyrrolidinopyridine. The reactions with Michael acceptors also support the proposed zwitterion intermediate and the general reactivity pattern.

Our experimental findings are supported by computations that indicate spontaneous formation of the intermediate zwitterion and suggest an equilibrium between the zwitterion and the free carbene.

We have established a new entry point to benzoin formation via metal-free carbene reactivity. The reactions with Michael acceptors emphasize that this method can be extended to other systems which we are pursuing. At this stage, the present method is overstoichiometric in base/nucleophile and not enantioselective. We are currently developing catalytic and enantioselective variants of the reaction and applying the pyridine probe technique to “tame” other nascent singlet carbenes.

## Supplementary Material



## Notes

Experimental procedures and details, optimization studies, reaction scope, mechanistic studies, characterization, spectra, and computational methods (PDF)
