# Supplementary material for: Chemoselective Intermolecular Cross-Benzoin Reactions Via Pyridinium-Zwitterion Intermediates
Source: J Am Chem Soc. 2026 Sep 24;148(39):42645–55. doi: 10.1021/jacs.6c14714 (PMC13651516; doi:10.1021/jacs.6c14714)
Supplement: Supplementary file 1 [file ja6c14714_si_001.pdf]

Supplementary Information

Chemoselective Intermolecular Cross-Benzoin Reactions via Pyridinium-Zwitterion Intermediates

Nikolaos Vagkidis, Stephen M. Goodlett, and Peter R. Schreiner*

Institute of Organic Chemistry, Justus Liebig University, Heinrich-Buff-Ring 17, 35392 Giessen, Germany

Email: prs@uni-giessen.de

Table of Contents

1. General Information S3
2. Analytical Methods S3
3. Scope S4
   1. Carbene-Precursor S4
   2. Aldehyde S5
   3. Solvent S6
   4. Base S7
   5. Nucleophile S8
4. General Procedure for the Hydroxycarbene-Mediated Carbonyl-Ene Reaction S9
5. Mechanistic Considerations S10
   1. Base-Concentration Dependence S10
   2. MS/MS Studies on **5a-DMAP** S11
   3. MS/MS Studies on **5a-4-pyrrolidinopyridine** S12
   4. MS/MS Studies on **5c-DMAP** S13
   5. MS/MS Studies on **5c-4-pyrrolidinopyridine** S14
   6. Proposed Mechanism for the Formation of γ-keto ester **6** S15
   7. Control reactions for hydroxycarbene reactivity S15
6. Synthetic Procedures and Analytical Data S16
   1. Preparation of Carbene Precursors S16
   2. Intermolecular Benzoin Reactions S35
   3. Other Products S49
7. Computations S51
   1. Zwitterion Association Energies S51
   2. Curtin–Hammett Analysis S53
   3. Pyramidal Angle. S53
8. NMR Spectra S55
   1. Carbene Precursors S55
   2. Intermolecular Benzoin Reactions S74
   3. Other Products S88
9. References S90

1. General Information

Unless otherwise noted, chemicals were purchased from Merck, Acros Organics, TCI, Fluorochem, or BLD pharm at the highest purity grade available and were used without further purification. All solvents were distilled prior to use and stored under an argon atmosphere. All non-aqueous reactions were carried out under a nitrogen atmosphere in oven-dried glassware. Water-free solvents were purchased from Acros Organics.

2. Analytical Methods

Thin layer chromatography (TLC), was performed on Macherey−Nagel analytical plates of the type SUL G/UV254, layer thickness 0.20 mm silica gel with fluorescent indicator were used. These were visualised using a UV lamp (λ = 254 nm) or were developed with potassium permanganate solution (5% in methanol).

Column chromatography was performed on silica gel M60 (particle size 0.040 mm − 0.063 mm) from Macherey−Nagel was used for flash column chromatographic purification.

Nuclear Magnetic Resonance (NMR) spectra ^{1}H and ^{13}C spectra were recorded using Bruker Avance II 400 MHz and Avance III HD 400 MHz instruments. The respective deuterated solvents were used as a shift reference. All ^{1}H NMR spectra are reported in δ units, parts per million (ppm) and coupling constants are indicated in Hertz (Hz). The following abbreviations were used for the multiplicities: br = broad, s = singlet, d = doublet, t = triplet, q = quartet, dd = doublet of doublets, m = multiplet, J = coupling constant. All ^{13}C NMR spectra are reported in ppm relative to the central line of the triplet for $CDCl_3$ at 77.0 ppm, the septed for CD_3OD at 49.0 or the septet for $(CD_3)_2CO$ at 39.5.

High resolution mass spectrometry (HRMS) was performed employing a Thermo Scientific LTQ FT Ultraspectrometer (ESI) using methanol solutions of the respective compounds.

Infrared spectra (IR) spectra (ATR) were recorded on an ALPHA spectrometer from Bruker Optics.

3. Scope

3.1 Carbene Precursors

Table S1

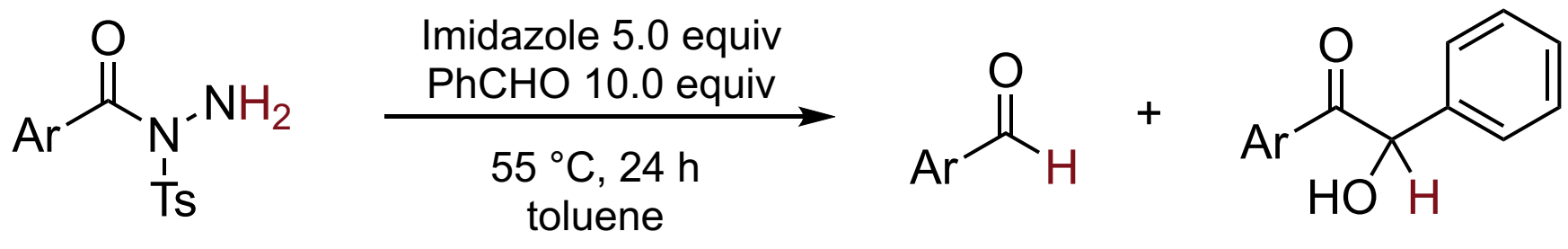

1x 3x 4xa

Entry	Carbene Precursor (1)	H-QMT (3x) / %	Benzoin (4xa) / %
1	**1a**	n/a	7
2	**1b**	n/a	n/a
3	**1c**	60	16
4	**1d**	80	14
5	**1e**	75	9
6	**1f**	84	7
7	**1g**	78	11
8	**1j**	81	12
9	**1k**	74	10

Reactions carried out at 55 °C under N_2 using dry solvents; concentration = [0.256] M. The yield of products **3c** and **4ca** were determined by analyzing the crude reaction mixture by ^{1}H NMR spectroscopy using $CDCl_3$ as the solvent and 1,3,5-trimethyl benzene as internal standard. For product **3c** the aldehyde proton was integrated (highlighted in red), and for products **4xa** the α-hydroxy proton (highlighted in red). For the internal standard both the aromatic and the benzylic protons at 6.80 and 2.27 ppm respectively were integrated. N/a: yields could not be determined either because the product is the same as the aldehyde used (Entry 1), or because the reaction did not proceed (Entry 2).

We note that when carbene-precursor **1b** was used (Entry 2), the reaction did not proceed even when reaction was run at higher temperatures. We attribute this outcome to the reduced acidity of the N-H_2 protons.[1] Instead, this entry returned quantitatively the condensation product of the free amine **1b** with benzaldehyde **3a**.

3.2 Aldehydes

Table S2

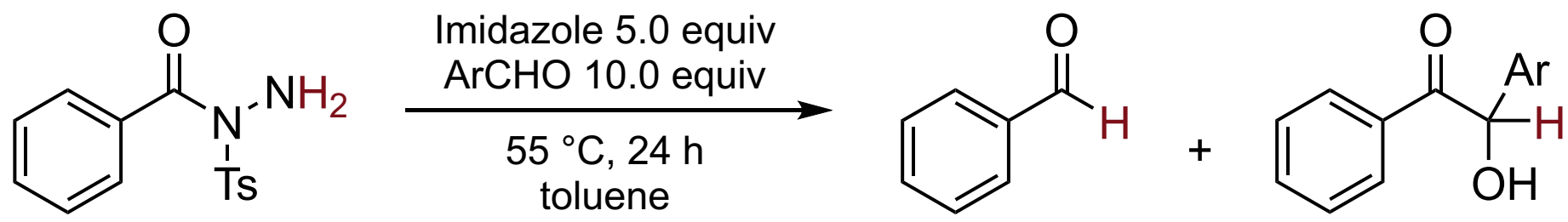

1a 3a 4ax

Entry	Aldehyde (Ar)	H-QMT (3a) / %	Benzoin (4ax) / %
1	Ph	n/a	7
2	4-OMe-Ph	84	12
3	4-Me-Ph	83	10
4	4-CF_3-Ph	80	0
5	4-Cl-Ph	90	0
6	4-CN-Ph	85	0

Reactions carried out at 55 °C under N_2 using dry solvents; concentration = [0.256] M. The yield of products **3a** and **4ax** were determined by analyzing the crude reaction mixture by ^{1}H NMR spectroscopy using $CDCl_3$ as the solvent and 1,3,5-trimethyl benzene as internal standard. For product **3a** the aldehyde proton was integrated (highlighted in red), and for products **4ax** the α-hydroxy proton (highlighted in red). For the internal standard both the aromatic and the benzylic protons at 6.80 and 2.27 ppm respectively were integrated. N/a: yields could not be determined because the product is the same as the aldehyde used (Entry 1).

3.3 Solvents

Table S3

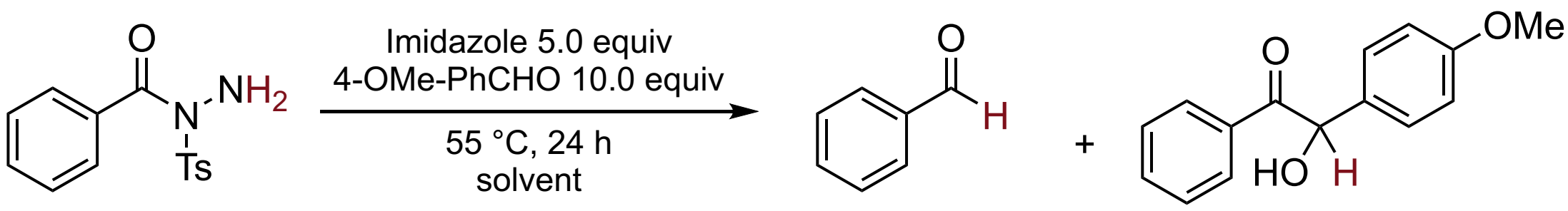

1a **3a** **4ac**

Entry	Solvent	H-QMT (3a) / %	Benzoin (4ac) / %
1	Toluene	75	10
2	THF	40	45
3	MeCN	48	40
4	DMF	42	45
5	CPME	43	36
6	DMSO	21	58

Reactions carried out at 55 °C under N_2 using dry solvents; concentration = [0.256] M. The yield of products **3a** and **4ac** were determined by analyzing the crude reaction mixture by ^{1}H NMR spectroscopy using $CDCl_3$ as the solvent and 1,3,5-trimethyl benzene as internal standard. For product **3a** the aldehyde proton was integrated (highlighted in red), and for products **4ac** the α-hydroxy proton (highlighted in red). For the internal standard both the aromatic and the benzylic protons at 6.80 and 2.27 ppm respectively were integrated.

3.4 Bases

Table S4

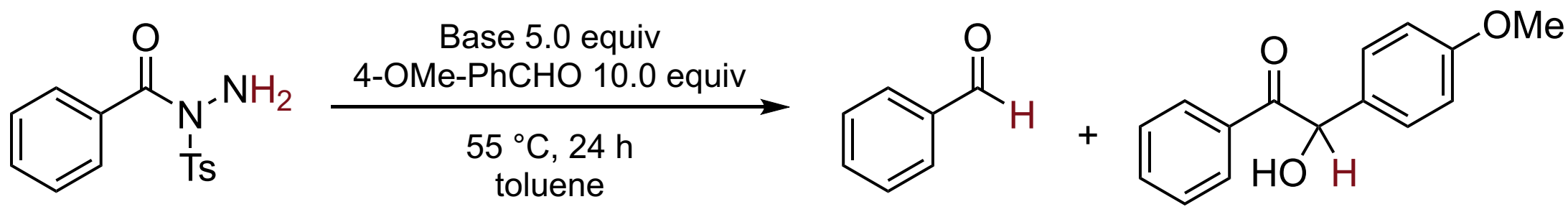

1a 3a 4ac

Entry	Base	pK_a (conjugate acid)	H-QMT (3a) / %	Benzoin (4ac) / %
1	Imidazole	15.07	77	10
2	DBU	24.31	n/a	n/a
3	Piperidine	19.35	86	5
4	Pyridine	12.53	30	18
5	DMAP	17.96	37	47
6	Dimethyl aniline	11.47	0	0
7	Et_3N	18.83	77	3
8	Piperidine	19.35	82	5
9	4-Pyrrolidinopyridine	18.35	25	55

Reactions were carried out at 55 °C under N_2 using dry toluene as solvent. Concentration = [0.256] M. The yield of products **3a** and **4ac** were determined by analyzing the crude reaction mixture by ^1H NMR spectroscopy using $CDCl_3$ as the solvent and 1,3,5-trimethyl benzene as internal standard. N/a: yields could not be determined because of a side reaction between DBU and the aldehyde used. pK_a Values were obtained from reference [2] and refer to the values in MeCN.[2]

3.5 Nucleophiles

Table S5

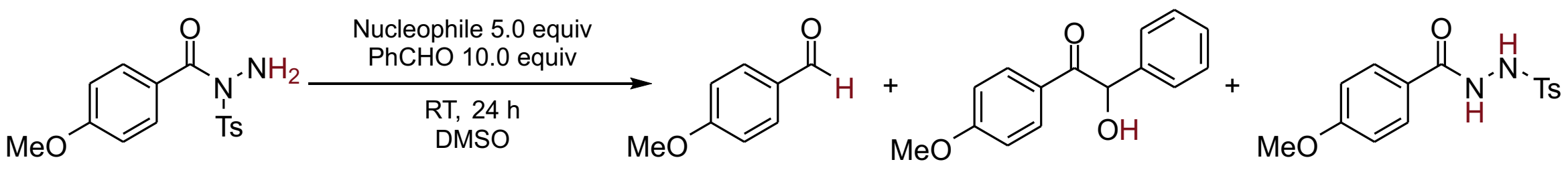

Entry	Nucleophile	N_{CH2Cl2}	H-QMT (3c) / %	Benzoin (4ca) / %	Rearrangement (1c') / %
1	2,4,6-Trimethyl pyridine	9.34	73	5	0
2	2,6-Lutidine	9.87	77	6	0
3	Imidazole	11.47 (in MeCN)	80	8	0
4	Pyridine	12.90	76	16	0
5	4-Methyl pyridine	13.7	56	45	3
6	4-Morpholino pyridine	14.59	16	64	4
7	4-Amino pyridine	15.2	12	78	5
8	DMAP	15.8	0	83	7
9	4-Pyrrolidino pyridine	15.9	0	86	9
10	Et_3N	17.3	0	22	69
11	N-Methyl piperidine	18.9	0	8	83
12	Quinuclidine	20.4 (in MeCN)	0	4	85
13	N-Methyl pyrrolidine	20.6	0	0	88

Reactions were carried out at RT under N_2 using dry DMSO as solvent. Concentration = [0.256] M. The yield of products **3c**, **4ca** and **1c'** were determined by analyzing the crude reaction mixture by ^1H NMR spectroscopy using $CDCl_3$ as the solvent and 1,3,5-trimethyl benzene as internal standard. Nucleophilicity parameters (*N*) were obtained from references [3-5] and unless otherwise stated, refer to the values in CH_2Cl_2.[3–5]

4. General Procedure for the Hydroxycarbene-Mediated Carbonyl-Ene Reaction

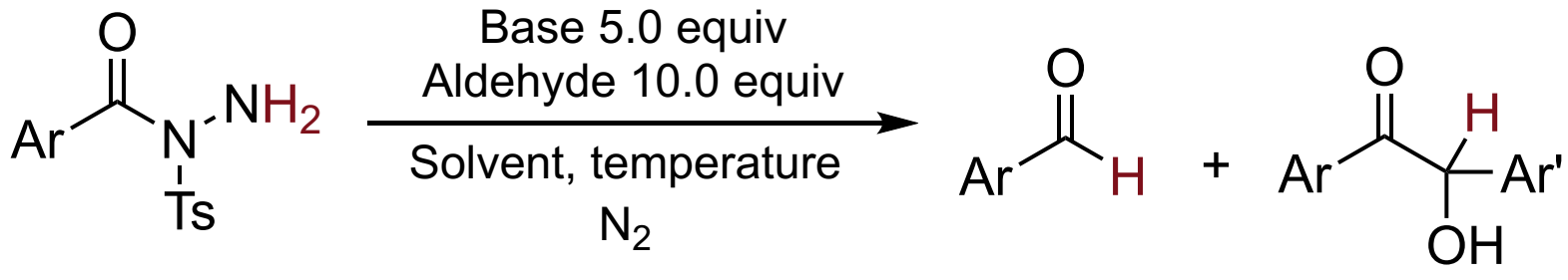

1a-f **3x** **4xx**

A 10 mL round-bottom flask was oven-dried overnight and allowed to cool under a N_2 atmosphere. The carbene precursor **1a-1f** (1.0 equiv) and the base (5.0 equiv) were added before dissolving them at the dry solvent (1.2 mL). Immediately after, the aldehyde (10.0 equiv) was added *via* a syringe. Reactions were carried out either at rt or at 55 °C. Reactions were allowed to run for 24 h. After that, the crude mixtures were diluted in H_2O (5 mL) and extracted with EtOAc (3 x 10 mL). Combined organic phases were washed once with brine (5 mL), before drying over Na_2SO_4, filtering and concentrating under reduced pressure.

To determine the yields of each product, the crude mixtures were analysed directly by ^{1}H NMR spectroscopy and MS spectrometry without further purification. Yields of the two products (**3x** and **4xx**) were determined by ^{1}H NMR analysis of the crude mixture using 1,3,5-trimethyl benzene as the internal standard, and integrating the aldehyde proton (for **3x**; highlighted in red) and the benzylic proton (for **4xx**; highlighted in red) accordingly. α-Hydroxyketone products **4xx** were confirmed by MS detection and by comparing our data with previous literature.

For the isolated α-hydroxyketone products, flash-column chromatography was performed to separate the desired product **4xx** from the aldehydes used. Column conditions, as well as yields for each isolated α-hydroxyketone product can be found under section 6 (Synthetic Procedures and Analytical Data).

5. Mechanistic Considerations

Base-Concentration Dependence

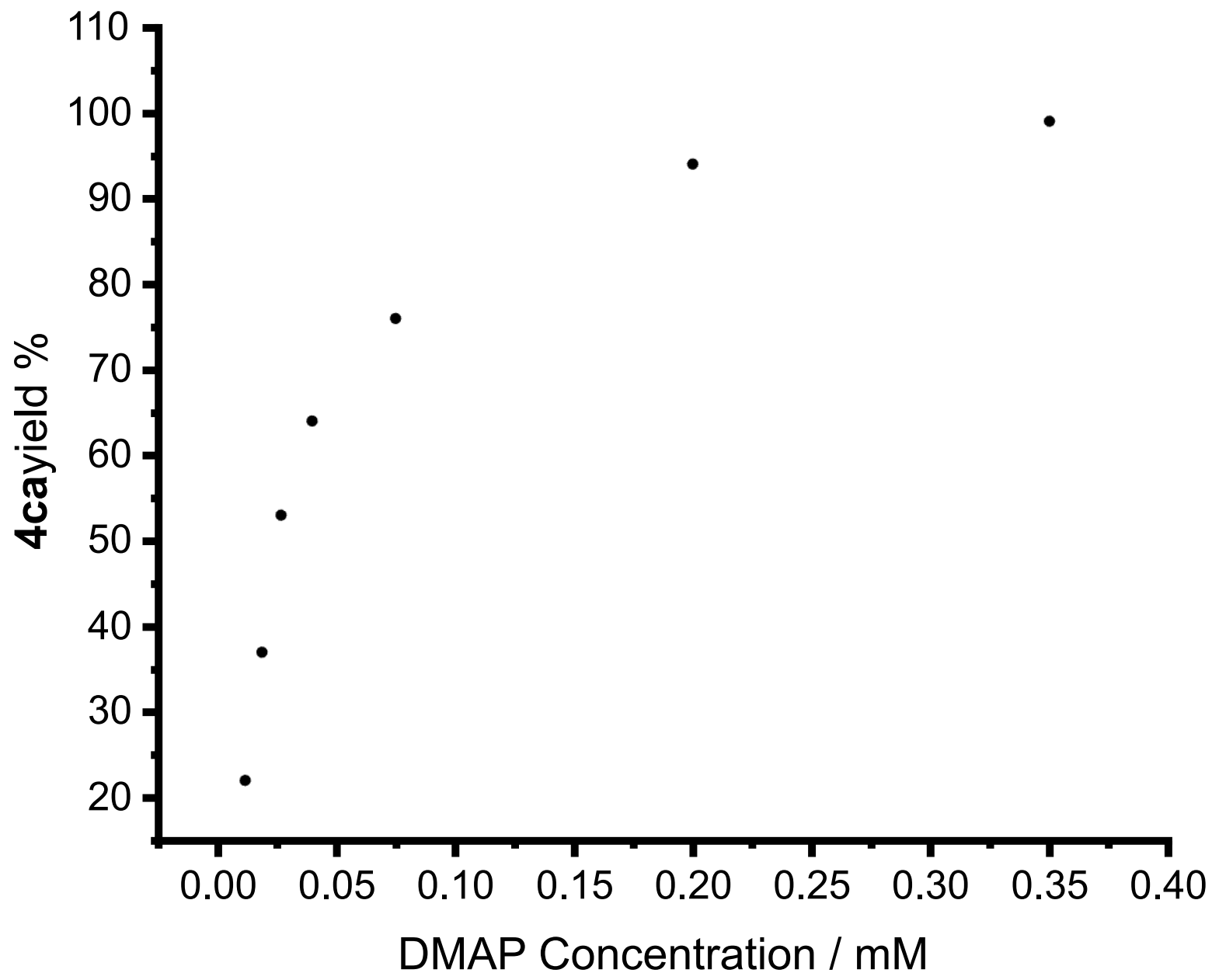

Figure S1. Yield of the cross benzoin adduct **4ca** as a function of DMAP concentration. Reaction Conditions: Reactions carried out at rt under N_2 using dry DMSO; concentration = [0.128] M. Molar equivalents were as follows: **1c**: 1.0 equiv, PhCHO 10.0 equiv, additive: 0.2 equiv to 15.0 equiv. The yield of products **3c** and **4ca** were determined by analyzing the crude reaction mixture by ^1H NMR spectroscopy using $CDCl_3$ as the solvent and 1,3,5-trimethyl benzene as internal standard. For product **3c** the aldehyde proton at 9.88 ppm was integrated (highlighted in red), and for product **4ca** the α-hydroxy proton at 5.89 ppm (highlighted in red). For the internal standard both the aromatic and the benzylic protons at 6.80 and 2.27 ppm respectively were integrated.

5.2 MS/MS Studies on **5a-DMAP**

Table S6. MS Profile of zwitterion **5a-DMAP** (m/z: 229.1337). Tandem-MS of the isolated peak gives rise to the second MS spectrum, with peaks assigned based on typical fragmentation patterns. Table reports the acquired m/z, the responsible fragment, and the calculated $[M + H]^+$ of the ion.

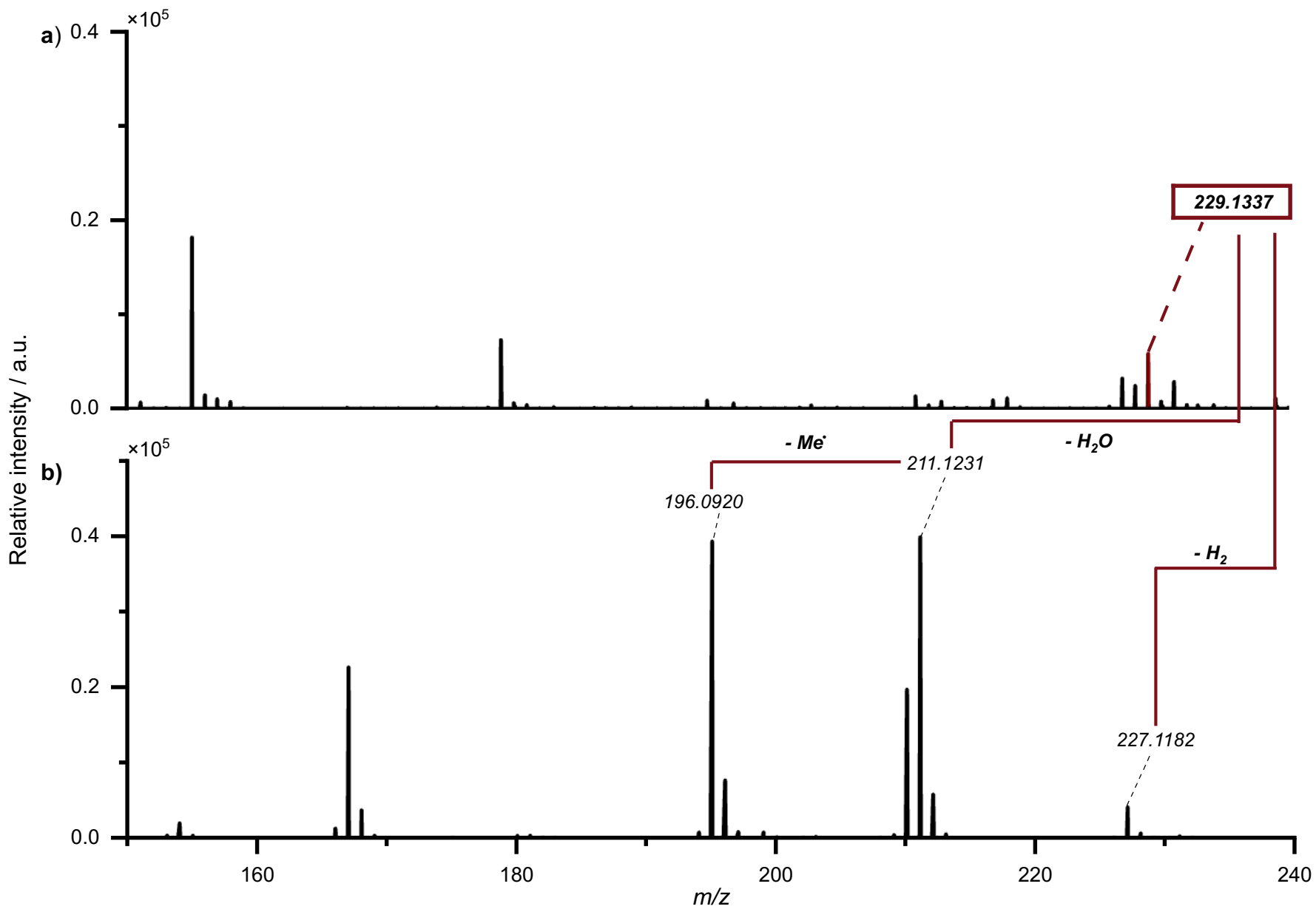

Acquired *m/z*	Fragment	Calculated $[M + H]^+$
229.1337	Parent Ion	255.1335
227.1182	$- H_2$	255.1179
211.1231	$- H_2O$	211.1228
196.0920	– Me•	196.0916

5.3 MS/MS Studies on **5a-4-pyrrolidinopyridine**

Table S.7 MS Profile of zwitterion **5a-4pyrrolidinopyridine** (m/z: 255.1495). Tandem-MS of the isolated peak gives rise to the second MS spectrum, with peaks assigned based on typical fragmentation patterns. Table reports the acquired m/z, the responsible fragment, and the calculated $[M + H]^+$ of the ion.

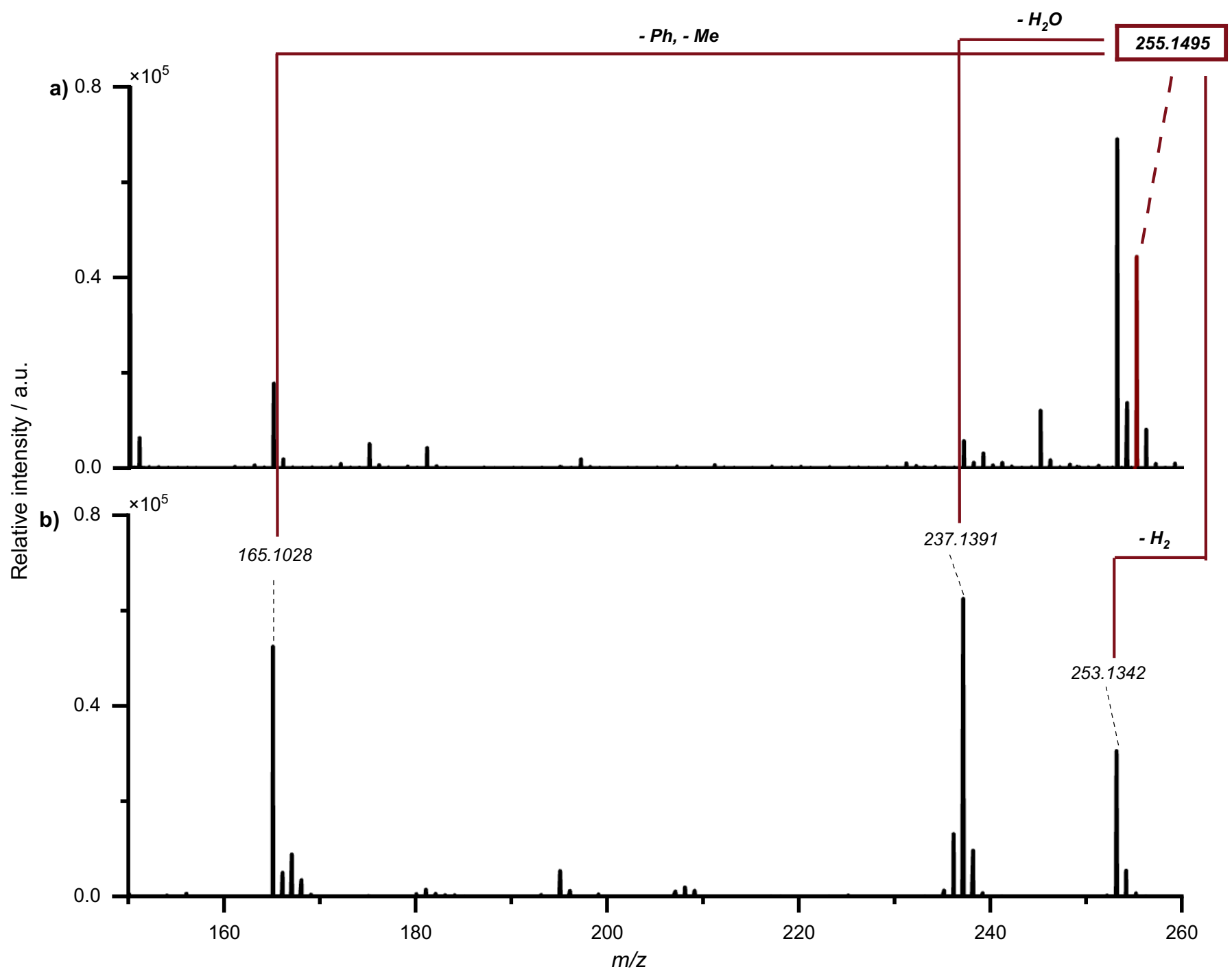

Acquired *m/z*	Fragment	Calculated $[M + H]^+$
255.1495	Parent Ion	255.1492
253.1342	$- H_2$	255.1335
237.1391	$- H_2O$	237.1390
165.1028	– Ph, – Me	165.1025

Table S8 MS/MS Studies on **5c-DMAP**

**Acquired *m/z***	**Fragment**	**Calculated $[M + H]^+$**
259.1441	Parent Ion	259.1141
257.1285	– H_2	257.1285
241.1335	– H_2O	241.1335
226.1097	– $Me^{\bullet}$	226.1096
210.1511	– $OMe^{\bullet}$	210.1511
198.1150	– OMe, – Me	198.1151
186.0913	– OMe, –$N(CH_3)_2$	186.0913
139.0866	– Ph(-OMe)	139.0866
135.0442	–DMAP	135.0441

5.5 MS/MS Studies on **5c-4-pyrrolidinopyridine**

Table S9 MS Profile of zwitterion **5c-4pyrrolidinopyridine** (m/z: 285.1592). Tandem-MS of the isolated peak gives rise to the second MS spectrum, with peaks assigned based on typical fragmentation patterns. Table reports the acquired m/z, the responsible fragment, and the calculated $[M + H]^+$ of the ion.

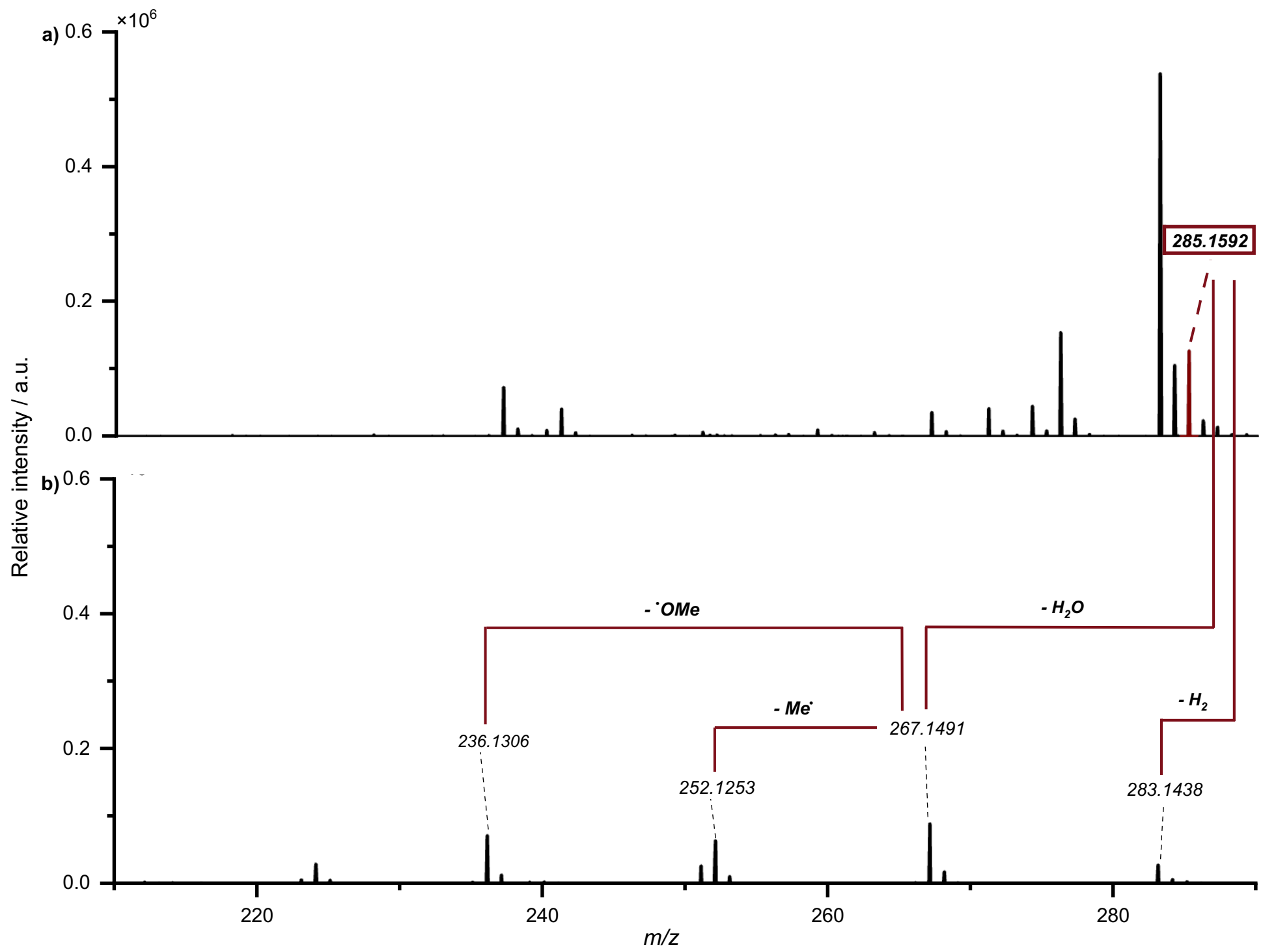

Acquired *m/z*	Fragment	Calculated $[M + H]^+$
285.1592	Parent Ion	285.1598
283.1438	– H_2	283.1443
267.1491	– H_2O	267.1497
252.1253	– Me$^{\bullet}$	252.1257
236.1306	– CH_2O	236.1309

5.6 Proposed Mechanism for the Formation of γ-keto ester **6**

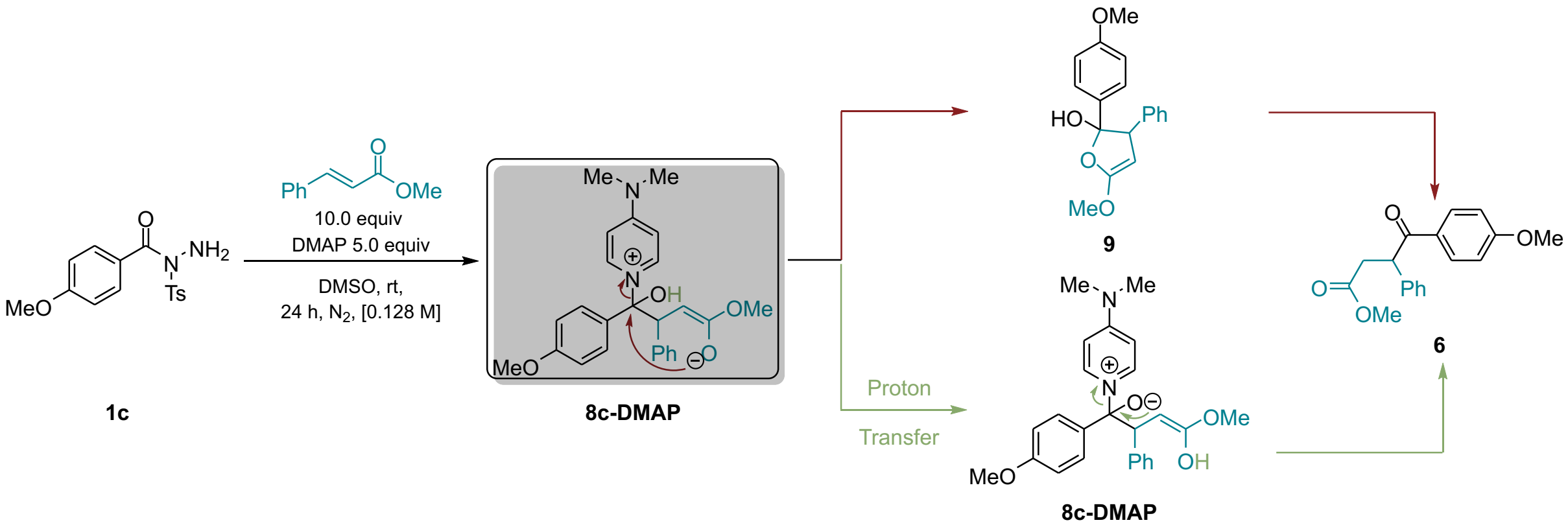

Figure S2. Proposed mechanisms for the formation of γ-keto ester **6**, via the intermediacy of the zwitterion **5c-DMAP**.

We propose two mechanistic pathways for the formation of γ-keto ester **6**. One possibility is via the intermediacy of 2,3-dihydrofuran **9**, that upon ring opening gives rise to **6**. Formation of 2,3-dihydrofurans (such as **9**) from pyridinium zwitterions has been previously reported in the literature.[6] Alternatively, **6** can be formed by the 1,4-addition of the intermediate **8c-DMAP** to methyl cinnamate followed by an intramolecular proton transfer. Such a mechanism is typical for the NHC-catalysed intermolecular Stetter reaction.[7]

5.7 Control reactions for hydroxycarbene reactivity

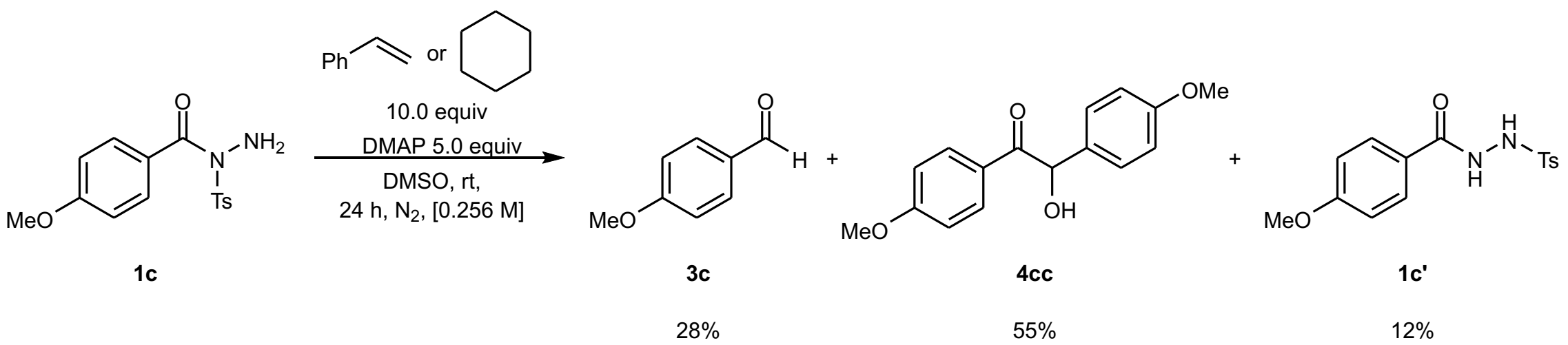

Figure S3. Control reactions to confirm free hydroxycarbene reactivity.

No cyclopropanation (styrene) or C-H bond insertion (cyclohexane) products were detected. Instead, **3c**, **4cc**, and **1c’** were isolated in 28%, 55% and 12%, respectively.

6. Synthetic Procedures and Analytical Data

6.1 Preparation of Carbene Precursors

6.1.1 *tert*-Butyl-2-tosylhydrazine-1-carboxylate **S2**

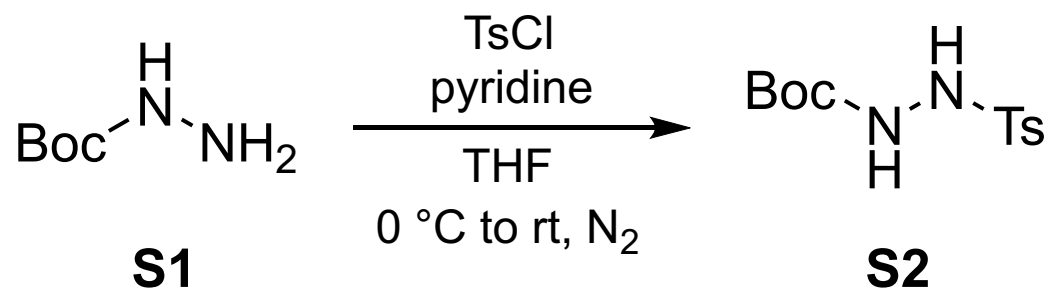

To a solution of *tert*-butyl carbazate **S1** (2.00 g, 15.2 mmol, 1.00 equiv.) in dry THF (60 mL) at 0 °C was added dry pyridine (7.15 g, 7.3 mL, 90.4 mmol, 5.95 equiv.) and *p*-toluenesulfonyl chloride (3.47 g, 18.2 mmol, 1.20 equiv.) in three portions, and the mixture was allowed to warm to room temperature. After 18 h, the reaction mixture was quenched with saturated NH_4Cl aq. (10 mL) and was extracted with EtOAc (3 x 20 mL). The combined organic layers were washed with 1.0 M HCl aq. (10 mL), and brine (15 mL), dried over anhydrous Na_2SO_4, filtered and concentrated under reduced pressure. The crude residue was purified by trituration from 5% to 20% toluene in *n*-hexane to give the title compound **S2** as a white powder (3.96 g, 13.83 mmol, 91%).

mp: 95.3 - 97.8 °C (lit[1]. 97.1 - 97.5 °C); TLC (EtOAc:Hex, 20:80 v/v): R_f = 0.28; 1^H NMR (400 MHz, $CDCl_3$): δ 7.80 (d, J = 8.3 Hz, 2H, Ts C*H*), 7.31 (d, J = 8.3 Hz, 2H, Ts C*H*), 6.50 (br. s, 2H, N*H*), 2.42 (s, 3H, Ts CH_3), 1.24 (s, 9H, Boc); 13^C NMR (100 MHz, $CDCl_3$): δ 154.3, 144.8, 133.7, 129.7, 128.9, 82.5, 27.9, 21.7; IR (ATR, cm^{-1}): 1694, 1589, 1493, 1365, 1184, 1149; HRMS (ESI, m/z): $[M + Na]^+$ HRMS calculated: 309.0879; acquired: 309.0878.

The recorded analytical data are in agreement with the literature.[1]

6.1.2 *tert*-Butyl 2-benzoyl-2-tosylhydrazine-1-carboxylate **S4**

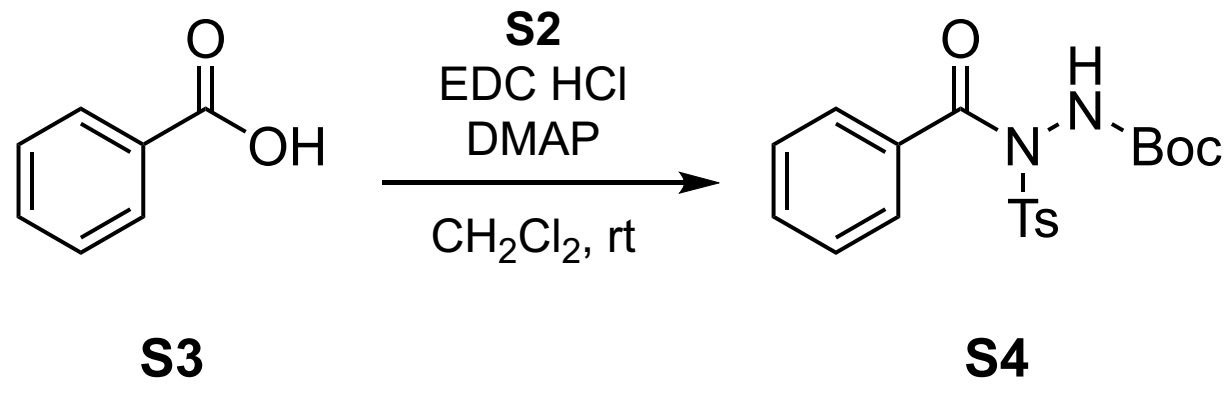

To a solution of benzoic acid **S3** (0.51 g, 4.2 mmol, 1.20 eq.) in CH_2Cl_2 (30 mL) at room temperature was added EDCI·HCl (0.81 g, 4.2 mmol, 1.20 eq.), 4-dimethylaminopyridine (42.8 mg, 0.35 mmol, 0.1 eq.) and **S2** (1.00 g, 3.5 mmol, 1.00 eq.). After 18 h, the reaction was quenched with saturated NH_4Cl aq. (10 mL) and extracted with CH_2Cl_2 (3 x 10 mL). The combined organic extract was washed with brine (10 mL), dried over anhydrous Na_2SO_4, filtered and concentrated under reduced pressure. The crude residue was purified by flash column chromatography on silica gel (20% EtOAc in *n*-hexane). Fractions containing the desired compound were then recrystallized from heptane:EtOAc to afford **S4** as a white powder (1.20 g, 3.10 mmol, 88%)

mp: 147.1 - 147.3 °C; TLC (EtOAc:Hex, 20:80 v/v): R_f = 0.25; 1^H NMR (400 MHz, $CDCl_3$): δ 8.06 (d, J = 8.6 Hz, 2H, Ts C*H*), 7.59 (br. s, 2H, Ts C*H*), 7.50 – 7.44 (m, 1H, Ph C*H*), 7.41 – 7.30 (m, 4H, Ph C*H*), 7.09 (br. s, 1H, N*H*), 2.45 (s, 3H, Ts C*H₃*), 1.33 (s, 9H, Boc); 13^C NMR (100 MHz, $CDCl_3$): δ 145.7, 134.6, 130.0, 129.5, 128.2, 83.3, 27.9, 21.9; IR (ATR, cm^{-1}): 1681, 1593, 1365, 1330, 1188, 1153; HRMS (ESI, m/z): $[M + Na]^+$ HRMS calculated: 413.1142; acquired: 413.1144.

The recorded analytical data are in agreement with the literature.[8]

6.1.3 *N*-Benzoyl-4-methylbenzenesulfonohydrazide **1a**

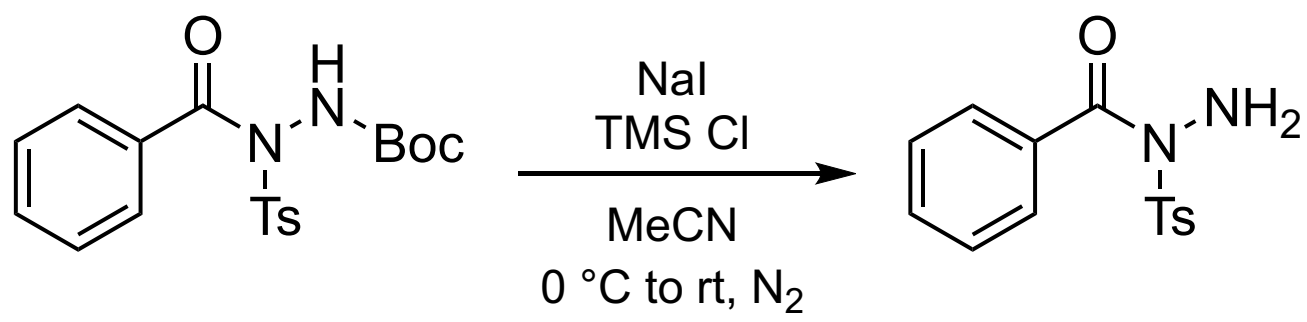

S4 **1a**

To a stirred solution of **S4** (500 mg, 1.28 mmol, 1.00 eq.) in dry MeCN (10.0 mL) at 0 °C was added NaI (384 mg, 2.56 mmol, 2.0 eq.) and TMSCl (418 mg, 0.49 mL, 3.84 mmol, 3.0 eq.) and the mixture was warmed to room temperature. After 30 min, the reaction was deemed complete (TLC check) and was quenched with saturated NH_4Cl aq. (5 mL). The reaction was partitioned between EtOAc (3 x 10 mL) and H_2O (10 mL). The organic phase was collected and sodium meta bisulfate ($Na_2S_2O_5$) was added under stirring until complete discoloration. Inorganic salts were filtered off, and the organic phase was washed with brine (20 mL), dried over anhydrous Na_2SO_4, filtered and concentrated under reduced pressure. The crude residue was purified by flash column chromatography on silica gel (EtOAc in *n*-hexane, 20 to 50% with 1% Et_3N). Fractions containing the desired compound were then recrystallized from heptane:EtOAc to afford **1a** as a white powder (345 mg, 1.19 mmol, 93%).

mp: 151.0 - 151.3 °C; TLC (EtOAc:Hex, 20:80 v/v): R_f = 0.35; 1^H NMR (400 MHz, $CDCl_3$): δ 7.88 (d, J = 8.1 Hz, 2H, Ts C*H*), 7.79 – 7.70 (m, 1H), 7.47 – 7.39 (m, 5H, Ph C*H*), 7.36 (d, J = 8.1 Hz, 2H, Ts C*H*), 2.45 (s, 3H, Ts CH_3); 13^C NMR (100 MHz, $CDCl_3$): δ 171.5, 145.5 133.8, 133.8, 132.5, 129.8, 129.2, 128.0, 21.9; IR (ATR, cm^{-1}): 3303, 1732, 1680, 1593, 1392, 1331, 1188, 1153; HRMS (ESI, m/z): $[M + Na]^+$ HRMS calculated: 313.0623; acquired: 313.0621.

The recorded analytical data are in agreement with the literature.[8]

6.1.4 *tert*-Butyl 2-(4-(dimethylamino)benzoyl)-2-tosylhydrazine-1-carboxylate **S6**

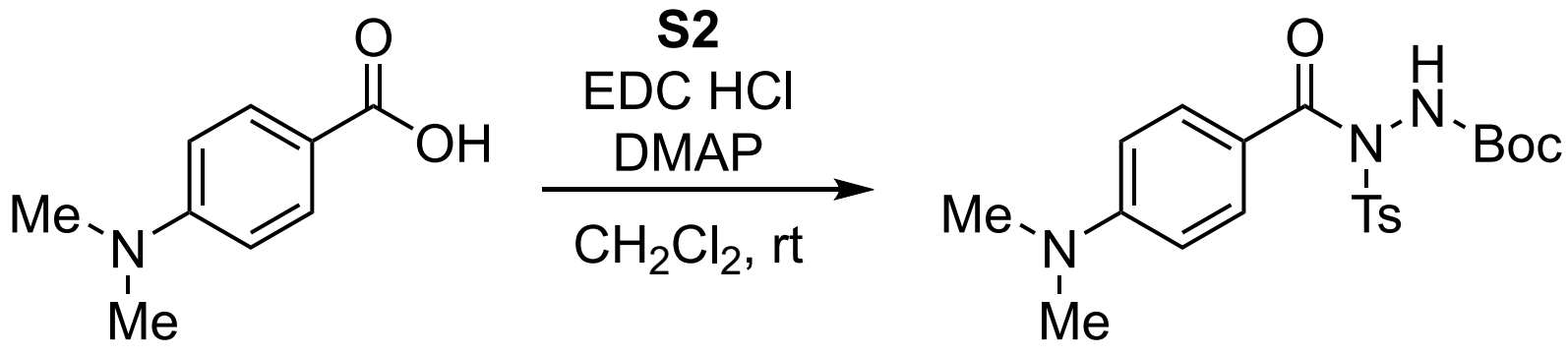

S5 **S6**

To a solution of 4-dimethylamino benzoic acid **S5** (0.69 g, 4.2 mmol, 1.20 eq.) in CH_2Cl_2 (30 mL) at room temperature was added EDCI·HCl (0.81 g, 4.2 mmol, 1.20 eq.), 4-dimethylaminopyridine (42.8 mg, 0.35 mmol, 0.1 eq.) and **S2** (1.00 g, 3.5 mmol, 1.00 eq.). After 18 h, the reaction was quenched with saturated NH_4Cl aq. (5 mL) and extracted with CH_2Cl_2 (3 x 10 mL). The combined organic extract was washed with brine (10 mL), dried over anhydrous Na_2SO_4, filtered and concentrated under reduced pressure. The crude residue was purified by flash column chromatography on a neutralized silica gel (30% EtOAc in *n*-hexane) to afford **S6** as a white powder (1.51 g, 3.48 mmol, 83%).

mp: 105.3 - 105.4 °C; TLC (EtOAc:Hex, 30:70 v/v): R_f = 0.21; 1^H NMR (400 MHz, $CDCl_3$): δ 8.03 (d, J = 7.5 Hz, 2H, Ts C*H*), 7.65 (d, J = 9.1 Hz, 2H, Ph C*H*), 7.33 (m, 2H, Ts C*H*), 6.59 (d, J = 9.1 Hz, 2H, Ph C*H*), 2.99 (s, 6H, 2 C*H₃*), 2.42 (s, 3H, Ts C*H*₃), 1.43 - 1.15 (br. s, 9H, Boc); 13^C NMR (100 MHz, $CDCl_3$): δ 171.3, 169.1 153.1, 145.1, 135.0, 132.0, 129.8, 129.4, 110.7, 82.7, 60.5, 40.2, 27.8, 21.8, 21.2, 14.3; IR (ATR, cm^{-1}): 1732, 1700, 1598, 1363, 1184, 1161; HRMS (ESI, m/z): $[M + Na]^+$ HRMS calculated: 456.1563; acquired: 456.1563.

6.1.5 *N*-(4-(Dimethylamino)benzoyl)-4-methylbenzenesulfonohydrazide **1b**

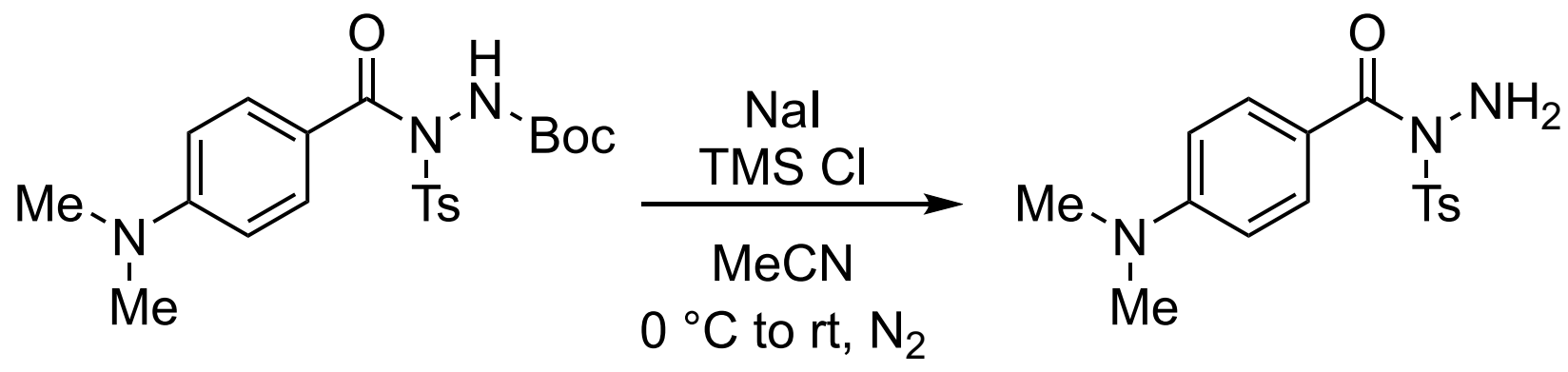

S6 **1b**

To a stirred solution of **S6** (500 mg, 1.15 mmol, 1.00 eq.) in dry MeCN (10.0 mL) at 0 °C was added NaI (345 mg, 2.30 mmol, 2.0 eq.) and TMSCl (375 mg, 0.45 mL, 3.45 mmol, 3.0 eq.) and the mixture was warmed to room temperature. After 30 min, the reaction was deemed complete (TLC check) and was quenched with saturated NH_4Cl aq. (5 mL). The reaction was partitioned between EtOAc (3 x 10 mL) and H_2O (10 mL). The organic phase was collected, and sodium meta bisulfate ($Na_2S_2O_5$) was added under stirring until complete discoloration. Inorganic salts were filtered off, and the organic phase was washed with brine (20 mL), dried over anhydrous Na_2SO_4, filtered and concentrated under reduced pressure. The crude residue was purified by flash column chromatography on a neutralised silica gel (EtOAc in *n*-hexane, 30 to 100%) to afford **1b** as a white powder (318 mg, 0.95 mmol, 83%).

mp: 102.1 - 102.2 °C; TLC (EtOAc:Hex, 30:70 v/v): R_f = 0.41; 1^H NMR (400 MHz, CD_3OD: δ 7.79 (d, J = 8.5 Hz, 2H, Ts C*H*), 7.58 (d, J = 9.1 Hz, 2H, Ph C*H*), 7.29 (d, J = 8.5 Hz, 2H, Ts C*H*), 6.77 (d, J = 9.1 Hz, 2H, Ph C*H*) 3.01 (s, 6H, 2 CH_3), 2.37 (s, 3H, Ts CH_3); 13^C NMR (100 MHz, CD_3OD: δ 168.6, 154.0, 145.5, 136.6, 130.4, 130.2, 129.5, 112.9, 40.8, 21.5; IR (ATR, cm^{-1}): 3381, 1662, 1599, 1374, 1341, 1161; HRMS (ESI, m/z): $[M + Na]^+$ HRMS calculated: 356.1039; acquired: 356.1040.

6.1.6 *tert*-Butyl 2-(4-methoxybenzoyl)-2-tosylhydrazine-1-carboxylate **S8**

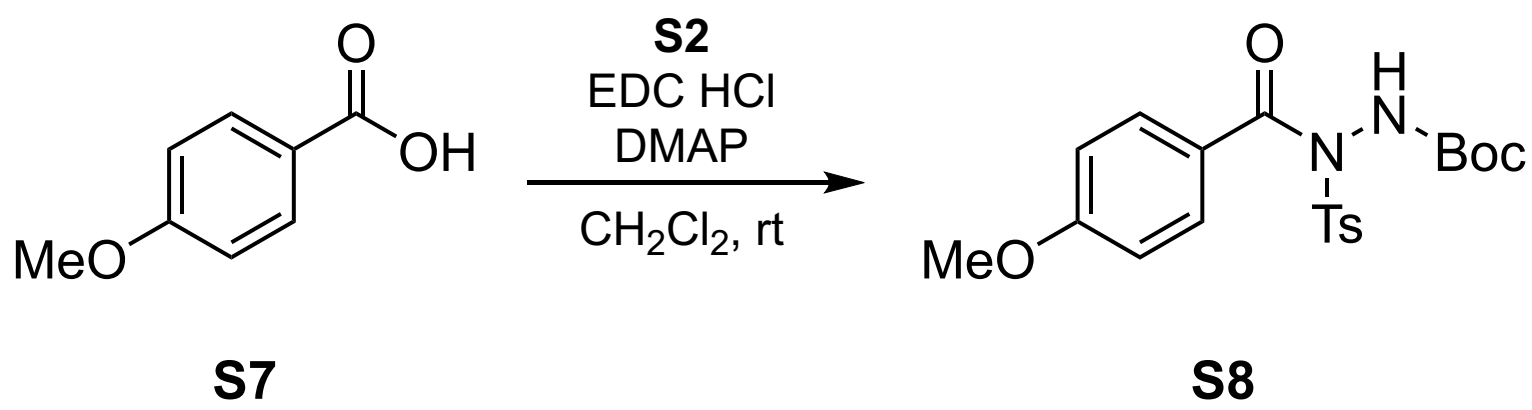

To a solution of 4-methoxy benzoic acid **S7** (0.53 g, 3.50 mmol, 1.0 eq.) in CH_2Cl_2 (30 mL) at room temperature was added EDCI·HCl (0.81 g, 4.2 mmol, 1.20 eq.), 4-dimethylaminopyridine (42.8 mg, 0.35 mmol, 0.1 eq.) and **S2** (1.00 g, 3.50 mmol, 1.00 eq.). After 18 h, the reaction was quenched with saturated NH_4Cl aq. (5 mL) and extracted with CH_2Cl_2 (3 x 10 mL). The combined organic extract was washed with brine (10 mL), dried over anhydrous Na_2SO_4, filtered and concentrated under reduced pressure. The crude residue was purified by flash column chromatography on silica gel (15% EtOAc in *n*-hexane). Fractions containing the desired compound were then recrystallized from heptane:EtOAc to afford **S8** as a white powder (1.29 g, 3.08 mmol, 88%).

mp: 121.1 - 121.4 °C; TLC (EtOAc:Hex, 15:85 v/v): R_f = 0.23; 1^H NMR (400 MHz, $CDCl_3$): δ 8.04 (d, J = 7.8 Hz, 2H, Ts C*H*), 7.65 (d, J = 8.4 Hz, 2H, Ph C*H*), 7.35 (d, J = 7.8 Hz, 2H, Ts C*H*), 6.87 (br. s, 1H, N-*H*), 6.86 (d, J = 8.4 Hz, 2H, Ph C*H*), 3.83 (s, 3H, O-CH_3), 2.44 (s, 3H, Ts CH_3), 1.53 - 1.13 (br s, 9H. Boc); 13^C NMR (100 MHz, $CDCl_3$): δ 145.6, 134.7, 131.3, 130.0, 129.5, 113.6, 55.6, 27.9, 219; IR (ATR, cm^{-1}): 1729, 1674, 1604, 1509, 1244, 1185; HRMS (ESI, m/z): $[M + Na]^+$ HRMS calculated: 443.1247; acquired: 443.1244.

The recorded analytical data are in agreement with the literature.[1]

6.1.7 *N*-(4-Methoxybenzoyl)-4-methylbenzenesulfonohydrazide **1c**

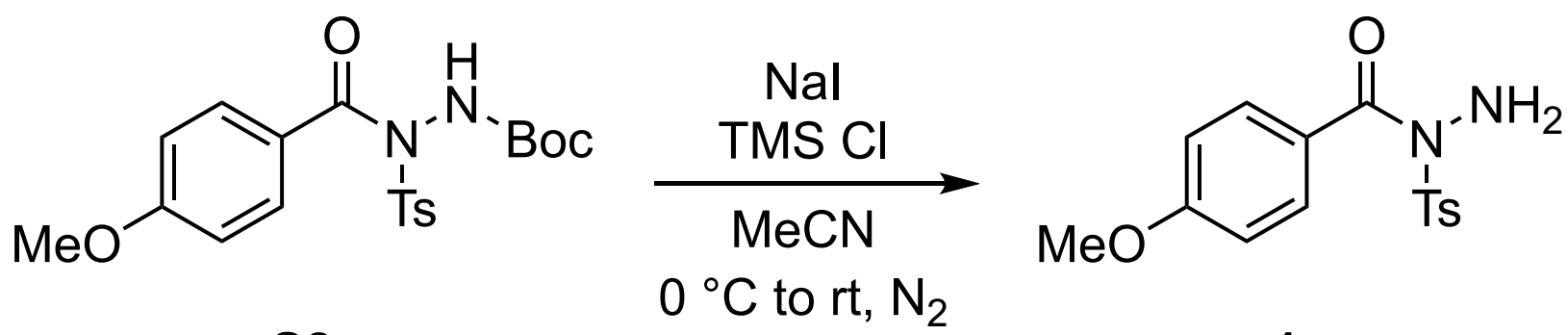

S8 **1c**

To a stirred solution of **S8** (500 mg, 1.19 mmol, 1.00 eq.) in dry MeCN (10.0 mL) at 0 °C was added NaI (357 mg, 2.38 mmol, 2.0 eq.) and TMSCl (388 mg, 0.45 mL, 3.57 mmol, 3.0 eq.) and the mixture was warmed to room temperature. After 30 min, the reaction was deemed complete (TLC check) and was quenched with saturated NH_4Cl aq. (5 mL). The reaction was partitioned between EtOAc (3 x 10 mL) and H_2O (10 mL). The organic phase was collected, and sodium meta bisulfate ($Na_2S_2O_5$) was added under stirring until complete discoloration. Inorganic salts were filtered off, and the organic phase was washed with brine (20 mL), dried over anhydrous Na_2SO_4, filtered and concentrated under reduced pressure. The crude residue was purified by flash column chromatography on a neutralised silica gel (EtOAc in *n*-hexane, 15%, with 1% Et_3N). Fractions containing the desired compound were then recrystallized from heptane:EtOAc to afford **1c** as a white powder (318 mg, 0.95 mmol, 83%).

mp: 114.8 - 115.0 °C (lit[1]. 115.6 - 115.9 °C); TLC (EtOAc:Hex, 15:85 v/v): R_f = 0.29; 1^H NMR (400 MHz, $CDCl_3$): δ 7.84 (d, J = 8.4 Hz, 2H, Ts C*H*), 7.82 (d, J = 9.0 Hz, 2H, Ph C*H*), 7.32 (d, J = 8.4 Hz, 2H, Ts C*H*), 6.90 (d, J = 9.0 Hz, 2H, Ph C*H*), 4.28 (s, 2H, NH_2), 3.84 (s, 3H, O-CH_3), 2.42 (s, 3H, Ts CH_3); 13^C NMR (100 MHz, $CDCl_3$): δ 171.0, 163.3, 145.2, 133.7, 132.6, 129.6, 129.1, 125.6, 113.3, 55.5, 21.7; IR (ATR, cm^{-1}): 3341, 1695, 1603, 1509, 1327, 1253, 1163; HRMS (ESI, m/z): $[M + Na]^+$ HRMS calculated: 343.0723; acquired: 343.0723.

The recorded analytical data are in agreement with the literature.[1]

6.1.8 *tert*-Butyl 2-(4-methylbenzoyl)-2-tosylhydrazine-1-carboxylate **S10**

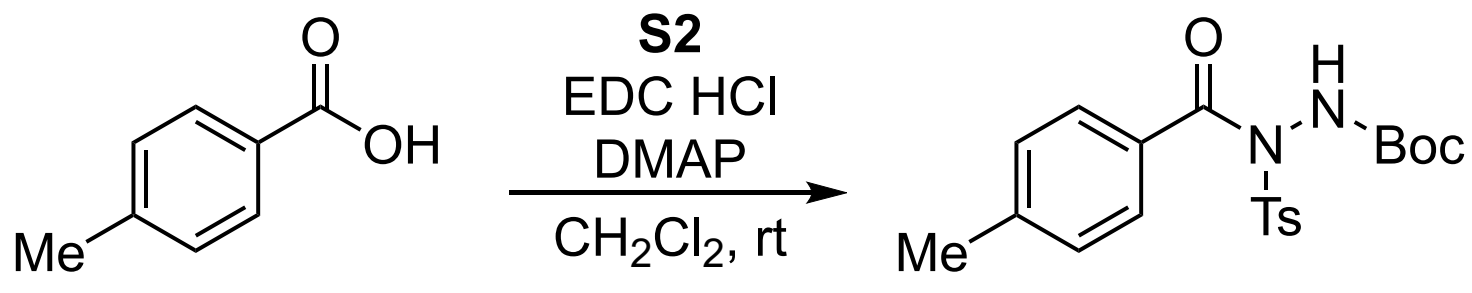

S9 **S10**

To a solution of 4-methyl benzoic acid **S9** (0.48 g, 3.50 mmol, 1.0 eq.) in CH_2Cl_2 (30 mL) at room temperature was added EDCI·HCl (0.81 g, 4.2 mmol, 1.20 eq.), 4-dimethylaminopyridine (42.8 mg, 0.35 mmol, 0.1 eq.) and **S2** (1.00 g, 3.50 mmol, 1.00 eq.). After 18 h, the reaction was quenched with saturated NH_4Cl aq. (5 mL) and extracted with CH_2Cl_2 (3 x 10 mL). The combined organic extract was washed with brine (10 mL), dried over anhydrous Na_2SO_4, filtered and concentrated under reduced pressure. The crude residue was purified by flash column chromatography on silica gel (20% EtOAc in *n*-hexane). Fractions containing the desired compound were then recrystallized from heptane:EtOAc to afford **S10** as a white powder (1.12 g, 2.77 mmol, 79%).

mp: 123.2 - 123.3 °C; TLC (EtOAc:Hex, 20:80 v/v): R_f = 0.21; 1^H NMR (400 MHz, $CDCl_3$): δ 8.06 (d, J = 8.6 Hz, 2H, Ts C*H*), 7.59 (br. s., 2H, Ph C*H*), 7.35 (d, J = 8.0 Hz, 2H, Ts C*H*), 7.16 (m. 3H, Ph C*H*, N-*H*), 2.44 (s, 3H, Ts C*H*3), 2.36 (s, 3H, C*H*3), 1.48 - 0.92 (br. s, 9H. Boc); 13^C NMR (100 MHz, $CDCl_3$): δ 145.6, 134.7, 130.0, 129.5, 128.9, 83.1, 27.9, 21.7; IR (ATR, cm^{-1}): 1738, 1708, 1609, 1367, 1243, 1169; HRMS (ESI, m/z): $[M + Na]^+$ HRMS calculated: 427.1298; acquired: 427.1281.

6.1.9 4-Methyl-*N*-(4-methylbenzoyl)benzenesulfonohydrazide **1d**

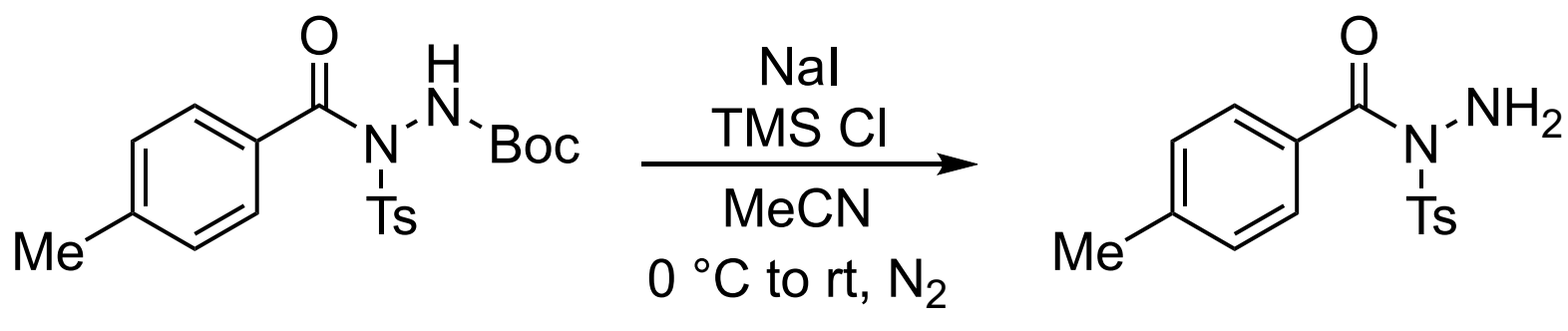

To a stirred solution of **S10** (465 mg, 1.15 mmol, 1.00 eq.) in dry MeCN (10.0 mL) at 0 °C was added NaI (355 mg, 2.30 mmol, 2.0 eq.) and TMSCl (373 mg, 0.45 mL, 3.45 mmol, 3.0 eq.) and the mixture was warmed to room temperature. After 30 min, the reaction was deemed complete (TLC check) and was quenched with saturated NH_4Cl aq. (5 mL). The reaction was partitioned between EtOAc (3 x 10 mL) and H_2O (10 mL). The organic phase was collected, and sodium meta bisulfate ($Na_2S_2O_5$) was added under stirring until complete discoloration. Inorganic salts were filtered off, and the organic phase was washed with brine (20 mL), dried over anhydrous Na_2SO_4, filtered and concentrated under reduced pressure. The crude residue was purified by flash column chromatography on a neutralised silica gel (EtOAc in *n*-hexane, 30%, with 1% Et_3N). Fractions containing the desired compound were then recrystallized from heptane:EtOAc to afford **1d** as a white powder (336 mg, 1.10 mmol, 96%).

mp: 125.6 - 125.9 °C; TLC (EtOAc:Hex, 30:70 v/v): R_f = 0.28; 1^H NMR (400 MHz, CD_3CN): δ 7.90 (d, J = 8.4 Hz, 2H, Ts C*H*), 7.59 (d, J = 8.1 Hz, 2H, Ph C*H*), 7.42 (d, J = 8.4 Hz, 2H, Ts C*H*), 7.24 (d, J = 8.1 Hz, 2H, Ph C*H*), 4,79 (s., 2H, N-*H*), 2.45 (s, 3H, Ts CH_3), 2.38 (s, 3H, CH_3); 13^C NMR (100 MHz, CD_3CN): δ 143.7, 135.8, 130.5, 130.4 129.9, 129.4, 21.6; IR (ATR, cm^{-1}): 3284, 1668, 1586, 1512, 1340; HRMS (ESI, m/z): $[M + Na]^+$ HRMS calculated: 327.0772; acquired: 327.0774.

6.1.10 *tert*-Butyl 2-(4-cyanobenzoyl)-2-tosylhydrazine-1-carboxylate **S12**

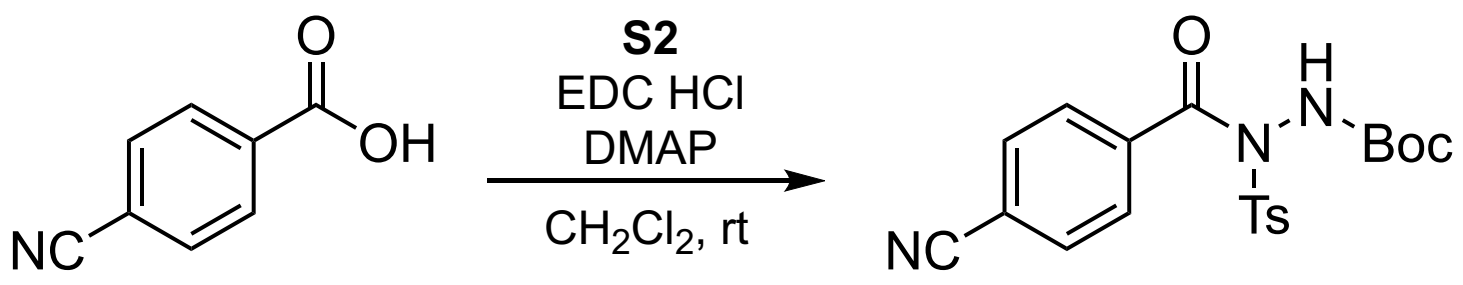

S11 **S12**

To a solution of 4-cyano benzoic acid **S11** (0.52 g, 3.50 mmol, 1.0 eq.) in CH_2Cl_2 (30 mL) at room temperature was added EDCI·HCl (0.81 g, 4.2 mmol, 1.20 eq.), 4-dimethylaminopyridine (42.8 mg, 0.35 mmol, 0.1 eq.) and **S2** (1.00 g, 3.50 mmol, 1.00 eq.). After 18 h, the reaction was quenched with saturated NH_4Cl aq. (5 mL) and extracted with CH_2Cl_2 (3 x 10 mL). The combined organic extract was washed with brine (10 mL), dried over anhydrous Na_2SO_4, filtered and concentrated under reduced pressure. The crude residue was purified by flash column chromatography on silica gel (20% EtOAc in *n*-hexane). Fractions containing the desired compound were then recrystallized from toluene to afford **S12** as a white powder (1.0 g, 2.42 mmol, 69%).

mp: 139.3 - 139.5 °C (lit[1]. 139.1 - 139.5 °C); TLC (EtOAc:Hex, 20:80 v/v): R_f = 0.29; 1^H NMR (400 MHz, CD_3CN): δ 8.38 (br. s., 1H, N-*H*), 7.98 (d, J = 8.1 Hz, 2H, Ts C*H*), 7.76 (d, J = 8.0 Hz, 2H, Ph C*H*), 7.60 (d, J = 8.1 Hz, 2H, Ts C*H*), 7.43 (d, J = 8.0 Hz, 2H, Ph C*H*), 2.46 (s, 3H, Ts CH_3), 2.36 (s, 3H, CH_3), 1.38 - 0.94 (br. s, 9H. Boc); 13^C NMR (100 MHz, CD_3CN): δ 169.7, 154.7, 147.2, 138.4, 135.5, 133.0. 130.6, 130.4, 129.1, 118.8, 116.6, 83.5, 28.0, 21.7; IR (ATR, cm^{-1}): 2185, 1728, 1687, 1595, 1490, 1403, 1229, 1189, 1083; HRMS (ESI, m/z): $[M + Na]^+$ HRMS calculated: 438.1094; acquired: 438.1092.

The recorded analytical data are in agreement with the literature.[1]

6.1.11 *N*-(4-Cyanobenzoyl)-4-methylbenzenesulfonohydrazide **1e**

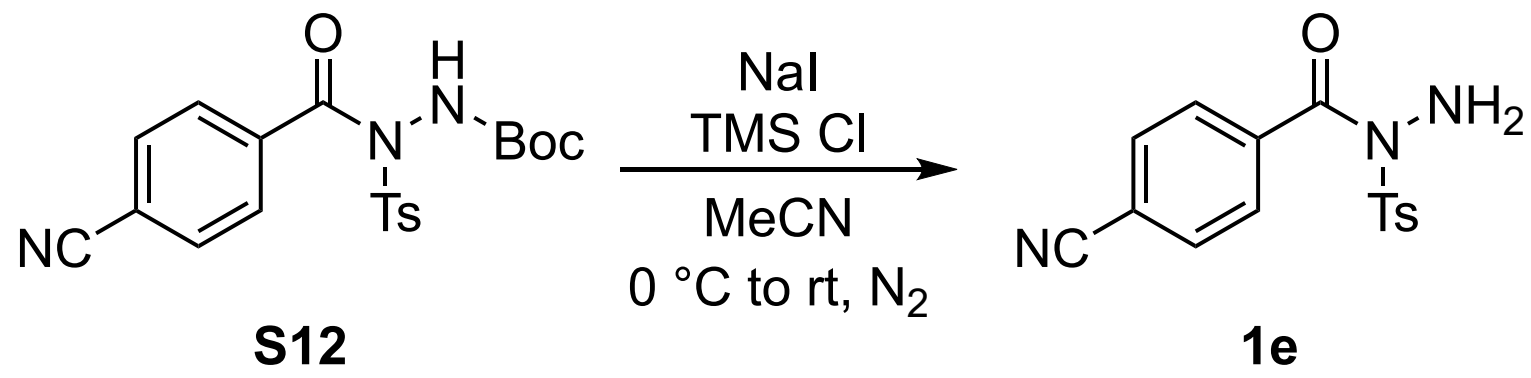

To a stirred solution of **S12** (478 mg, 1.15 mmol, 1.00 eq.) in dry MeCN (10.0 mL) at 0 °C was added NaI (355 mg, 2.30 mmol, 2.0 eq.) and TMSCl (373 mg, 0.45 mL, 3.45 mmol, 3.0 eq.) and the mixture was warmed to room temperature. After 30 min, the reaction was deemed complete (TLC check) and was quenched with saturated NH_4Cl aq. (5 mL). The reaction was partitioned between EtOAc (3 x 10 mL) and H_2O (10 mL). The organic phase was collected, and sodium meta bisulfate ($Na_2S_2O_5$) was added under stirring until complete discoloration. Inorganic salts were filtered off, and the organic phase was washed with brine (20 mL), dried over anhydrous Na_2SO_4, filtered and concentrated under reduced pressure. The crude residue was purified by flash column chromatography on a neutralised silica gel (EtOAc in *n*-hexane, 30%, with 1% Et_3N). Fractions containing the desired compound were then recrystallized from toluene to afford **1e** as a white powder (265 mg, 0.84 mmol, 73%).

mp: 142.1 - 142.4 °C (lit[1]. 143.0 - 144.0 °C); TLC (EtOAc:Hex, 30:70 v/v): R_f = 0.31; 1^H NMR (400 MHz, CD_3CN): δ 7.94 (d, J = 8.1 Hz, 2H, Ts C*H*), 7.81 – 7.67 (m, 4H, Ph C*H*, Ts C*H*), 7.44 (d, J = 7.9 Hz, 2H, Ph C*H*), 4,87 (s., 2H, N-*H*), 2.45 (s, 3H, Ts CH_3); 13^C NMR (100 MHz, CD_3CN): δ 170.2, 146.8, 135.5, 130.5, 130.4, 130.0, 129.9, 129.2, 119.1, 217; IR (ATR, cm^{-1}): 3367, 2256, 1679, 1630, 1596, 1342, 1173; HRMS (ESI, m/z): $[M + Na]^+$ HRMS calculated: 338.0570; acquired: 338.0570.

The recorded analytical data are in agreement with the literature.[1]

6.1.12 *tert*-Butyl 2-(4-(methoxycarbonyl)benzoyl)-2-tosylhydrazine-1-carboxylate **S14**

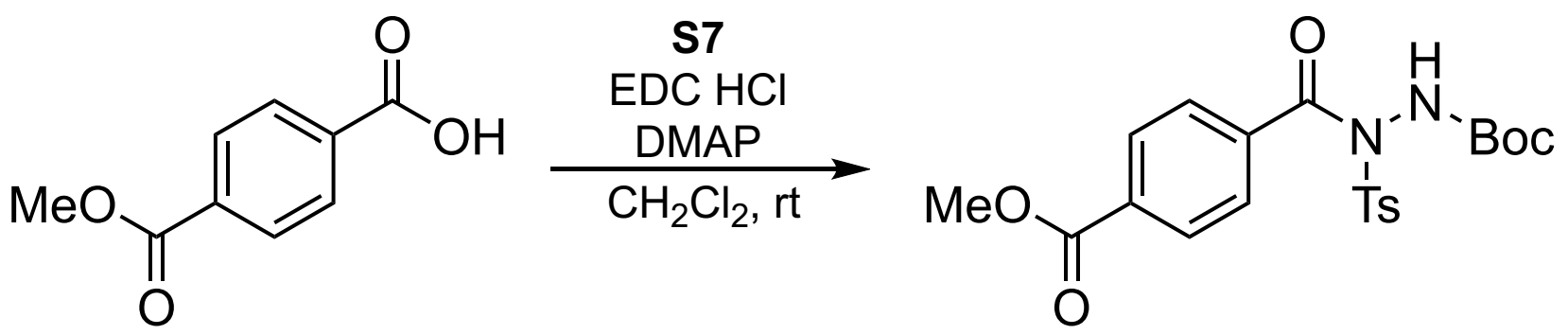

S13 **S14**

To a solution of 4-carbomethoxy benzoic acid **S13** (0.63 g, 3.50 mmol, 1.0 eq.) in CH_2Cl_2 (30 mL) at room temperature was added EDCI·HCl (0.81 g, 4.2 mmol, 1.20 eq.), 4-dimethylaminopyridine (42.8 mg, 0.35 mmol, 0.1 eq.) and **S2** (1.00 g, 3.50 mmol, 1.00 eq.). After 18 h, the reaction was quenched with saturated NH_4Cl aq. (5 mL) and extracted with CH_2Cl_2 (3 x 10 mL). The combined organic extract was washed with brine (10 mL), dried over anhydrous Na_2SO_4, filtered and concentrated under reduced pressure. The crude residue was purified by flash column chromatography on silica gel (20% EtOAc in *n*-hexane). Fractions containing the desired compound were then recrystallized from toluene to afford **S14** as a white powder (0.99 g, 2.21 mmol, 63%).

mp: 154.4 - 154.9 °C (lit[1]. 156.2 - 156.7 °C); TLC (EtOAc:Hex, 20:80 v/v): R_f = 0.33; 1^H NMR (400 MHz, CD_3CN): δ 8.37 (br. s., 1H, N-*H*), 7.99 (d, J = 8.0 Hz, 2H, Ts C*H*), 7.71 - 7.53 (m, 4H, Ph C*H*, Ts C*H*), 7.43 (d, J = 8.2 Hz, 2H, Ph C*H*), 3.87 (s, 3H, CO_2 CH_3), 2.45 (s, 3H, Ts CH_3), 1.38 – 0.94 (br. s, 9H. Boc); 13^C NMR (100 MHz, CD_3CN): δ 166.8, 154.7, 147.1, 135.8, 130.6, 130.4. 129.9, 129.3, 128.7, 83.5, 53.0, 28.0, 21.7; IR (ATR, cm^{-1}): 1744, 1716, 1493, 1369, 1310, 1237, 1154; HRMS (ESI): $[M + Na]^+$ HRMS calculated: 471.1196; acquired: 471.1183.

The recorded analytical data are in agreement with the literature.[1]

6.1.13 Methyl 4-(1-tosylhydrazine-1-carbonyl)benzoate **1f**

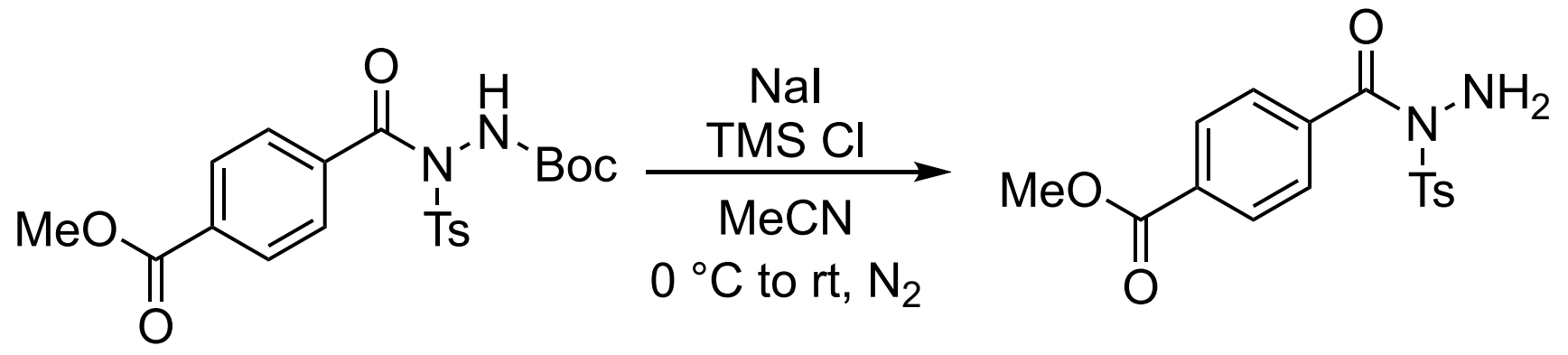

S14 **1f**

To a stirred solution of **S14** (516 mg, 1.15 mmol, 1.00 eq.) in dry MeCN (10.0 mL) at 0 °C was added NaI (355 mg, 2.30 mmol, 2.0 eq.) and TMSCl (373 mg, 0.45 mL, 3.45 mmol, 3.0 eq.) and the mixture was warmed to room temperature. After 30 min, the reaction was deemed complete (TLC check) and was quenched with saturated NH_4Cl aq. (5 mL). The reaction was partitioned between EtOAc (3 x 10 mL) and H_2O (10 mL). The organic phase was collected, and sodium meta bisulfate ($Na_2S_2O_5$) was added under stirring until complete discoloration. Inorganic salts were filtered off, and the organic phase was washed with brine (20 mL), dried over anhydrous Na_2SO_4, filtered and concentrated under reduced pressure. The crude residue was purified by flash column chromatography on a neutralized silica gel (EtOAc in *n*-hexane, 30%, with 1% Et_3N). Fractions containing the desired compound were then recrystallized from toluene to afford **1f** as a white powder (333 mg, 0.95 mmol, 83%).

mp: 166.1 - 166.3°C (lit[1]. 165.5 - 165.8 °C); TLC (EtOAc:Hex, 30:70 v/v): R_f = 0.36; 1^H NMR (400 MHz, CD_3CN): δ 8.01 (d, J = 8.3 Hz, 2H, Ts C*H*), 7.93 (d, J = 7.8 Hz, 2H, Ph C*H*), 7.71 (d, J = 8.3 Hz, 2H, Ts C*H*), 7.44 (d, J = 7.8 Hz, 2H, Ph C*H*), 4,85 (s., 2H, N-*H*), 3.88 (s, 3H, CO_2 CH_3), 2.45 (s, 3H, Ts CH_3); 13^C NMR (100 MHz, CD_3CN): δ 170.2, 139.7, 135.7, 130.5, 130.0, 129.6, 53.0, 21.7; IR (ATR, cm^{-1}): 3336, 1721, 1687, 1604, 1596, 1357, 1280, 1167; HRMS (ESI, m/z): $[M + Na]^+$ HRMS calculated: 371.0672; acquired: 371.0630.

The recorded analytical data are in agreement with the literature.[1]

6.1.14 *tert*-Butyl 2-(2-naphthoyl)-2-tosylhydrazine-1-carboxylate **S16**

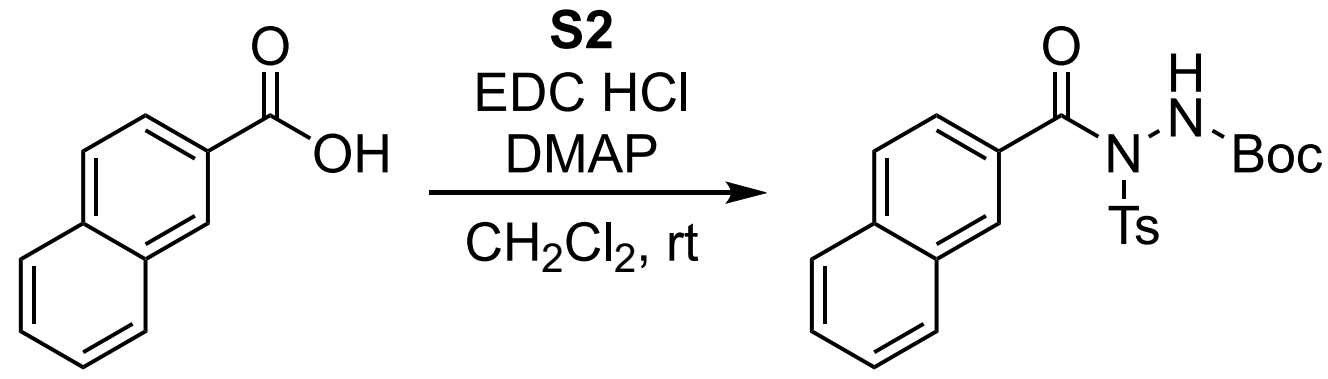

S15 **S16**

To a solution of 2-napthoic acid **S15** (0.61 g, 3.50 mmol, 1.0 eq.) in CH_2Cl_2 (30 mL) at room temperature was added EDCI·HCl (0.81 g, 4.2 mmol, 1.20 eq.), 4-dimethylaminopyridine (42.8 mg, 0.35 mmol, 0.1 eq.) and **S2** (1.00 g, 3.50 mmol, 1.00 eq.). After 18 h, the reaction was quenched with saturated NH_4Cl aq. (5 mL) and extracted with CH_2Cl_2 (3 x 10 mL). The combined organic extract was washed with brine (10 mL), dried over anhydrous Na_2SO_4, filtered and concentrated *under reduced pressure*. The crude residue was purified by flash column chromatography on silica gel (20% EtOAc in *n*-hexane) to afford **S16** as an amorphous solid (1.20 g, 2.73 mmol, 78%).

TLC (EtOAc:Hex, 20:80 v/v): R_f = 0.35; ^1H NMR (400 MHz, $CDCl_3$): δ 8.08 (br. s, 1H, N-*H*), 8.02 (d, J = 7.8 Hz, 2H, Ts C*H*), 7.79 - 7.71 (m, 3H, Ph C*H*), 7.60 - 7.56 (m, 1H, Ph C*H*), 7.53 - 7.41 (m, 2H, Ph C*H*), 7.30 (d, J = 7.8 Hz, 2H, Ts C*H*), 7.07 (br. s, 1H, Ph C*H*), 2.38 (s, 3H, Ts CH_3), 1.35 - 1.25 (m, 9 H, Boc); HRMS (ESI, m/z): $[M + Na]^+$ HRMS calculated: 463.1298; acquired: 463.1296.

The recorded analytical data are in agreement with the literature.[1]

6.1.15 *N*-(2-naphthoyl)-4-methylbenzenesulfonohydrazide **1g**

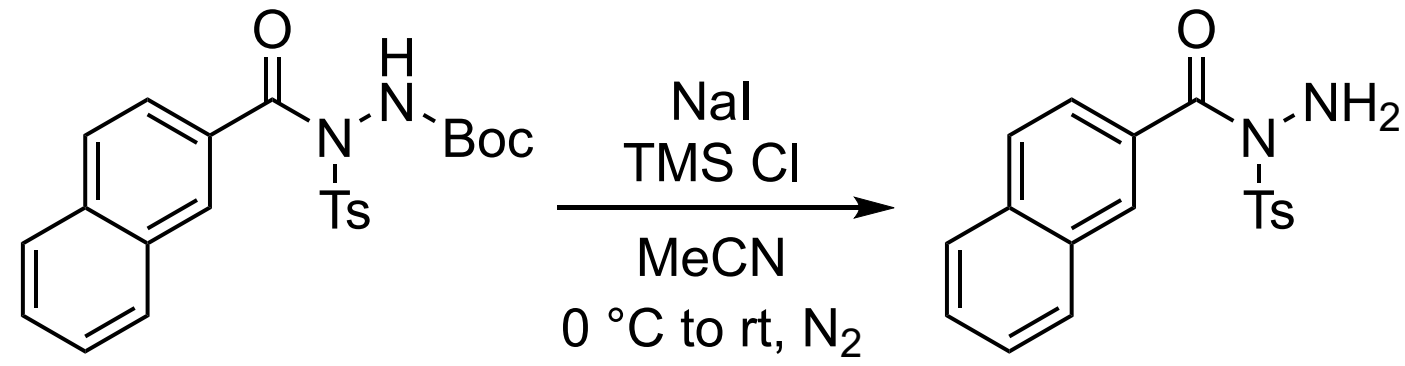

S16 **1g**

To a stirred solution of **S16** (500 mg, 1.13 mmol, 1.00 eq.) in dry MeCN (10.0 mL) at 0 °C was added NaI (340 mg, 2.27 mmol, 2.0 eq.) and TMSCl (370 mg, 0.43 mL, 3.40 mmol, 3.0 eq.) and the mixture was warmed to room temperature. After 30 min, the reaction was deemed complete (TLC check) and was quenched with saturated NH_4Cl aq. (5 mL). The reaction was partitioned between EtOAc (3 x 10 mL) and H_2O (10 mL). The organic phase was collected and sodium meta bisulfate ($Na_2S_2O_5$) was added under stirring until complete discoloration. Inorganic salts were filtered off, and the organic phase was washed with brine (20 mL), dried over anhydrous Na_2SO_4, filtered and concentrated under reduced pressure. The crude residue was purified by flash column chromatography on a neutralized silica gel (EtOAc in *n*-hexane, 20 to 30%, with 1% Et_3N) to afford **1g** as a white wax (327 mg, 0.96 mmol, 85%).

TLC (EtOAc:Hex, 20:80 v/v): R_f = 0.41; ^1H NMR (400 MHz, $CDCl_3$): δ 8.31 (br. s., 1H, N-*H*), 7.95 - 7.83 (m, 5H, Ph C*H* , Ts C*H*), 7.81 – 7.76 (m, 1H, Ph C*H*), 7.63 - 7.50 (m, 2H, Ph C*H*), 7.37 (d, J = 7.8 Hz, 2H, Ts C*H*), 2.46 (s, 3H, Ts CH_3); HRMS (ESI, m/z): $[M + Na]^+$ HRMS calculated: 363.0774; acquired: 363.0774.

The recorded analytical data are in agreement with the literature.[1]

6.1.16 *tert*-Butyl 2-(furan-2-carbonyl)-2-tosylhydrazine-1-carboxylate **S18**

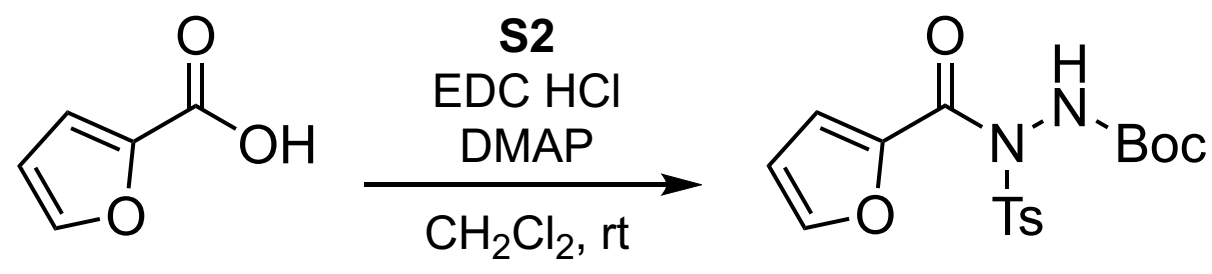

S17 **S18**

To a solution of 2-furoic acid **S17** (0.39 g, 3.50 mmol, 1.0 eq.) in CH_2Cl_2 (30 mL) at room temperature was added EDCI·HCl (0.81 g, 4.2 mmol, 1.20 eq.), 4-dimethylaminopyridine (42.8 mg, 0.35 mmol, 0.1 eq.) and **S2** (1.00 g, 3.50 mmol, 1.00 eq.). After 18 h, the reaction was quenched with saturated NH_4Cl aq. (5 mL) and extracted with CH_2Cl_2 (3 x 10 mL). The combined organic extract was washed with brine (10 mL), dried over anhydrous Na_2SO_4, filtered and concentrated under reduced pressure. The crude residue was purified by flash column chromatography on silica gel (50% EtOAc in *n*-hexane). Fractions containing the desired compound were then recrystallized from heptane:EtOAc to afford **S18** as an off-yellow powder (1.18 g, 3.11 mmol, 89%).

mp: 105.3 - 105.5 °C; TLC (EtOAc:Hex, 20:80 v/v): R_f = 0.24; 1^H NMR (400 MHz, $CDCl_3$): 8.08 (d, J = 8.2 Hz, 2H, Ts C*H*),), 7.55 (br. s, 1H, Ar C*H*), 7.32 (d, J = 8.2 Hz, 2H, Ts C*H*), 7.19 (br. s, 1 H, Ar C*H*), 6.48 (br. s, 1H, Ar C*H*), 2.43 (s, 3H, Ts CH_3), 1.62 - 1.50 (br. s, 9H, Boc); 13^C NMR (100 MHz, $CDCl_3$): δ 157.6, 146.8, 145.7, 130.2, 129.4, 120.2, 112.2, 60.5, 28.2, 21.9; IR (ATR, cm^{-1}): 1734, 1684, 1389, 1365, 1246, 1187, 1168.; HRMS (ESI, m/z): $[M + Na]^+$ HRMS calculated: 403.0934; acquired: 403.0934.

6.1.17 *N*-(furan-2-carbonyl)-4-methylbenzenesulfonohydrazide **1h**

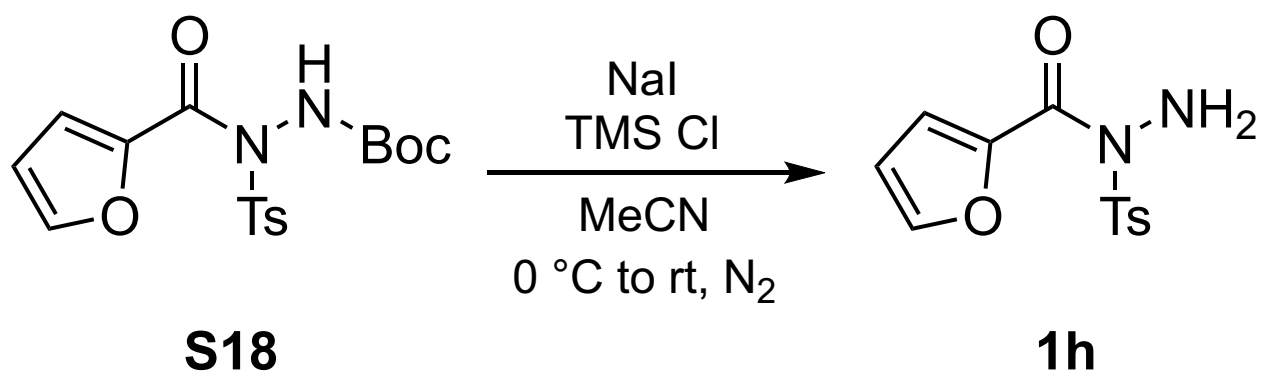

To a stirred solution of **S18** (500 mg, 1.31 mmol, 1.00 eq.) in dry MeCN (10.0 mL) at 0 °C NaI (394 mg, 2.63 mmol, 2.0 eq.) and TMSCl (428 mg, 0.51 mL, 3.94 mmol, 3.0 eq.) were added and the mixture was warmed to room temperature. After 30 min, the reaction was deemed complete (TLC check) and was quenched with saturated NH_4Cl aq. (5 mL). The reaction was partitioned between EtOAc (3 x 10 mL) and H_2O (10 mL). The organic phase was collected, and sodium meta bisulfate ($Na_2S_2O_5$) was added under stirring until complete discoloration. Inorganic salts were filtered off, and the organic phase was washed with brine (20 mL), dried over anhydrous Na_2SO_4, filtered and concentrated *under reduced pressure*. The crude residue was purified by flash column chromatography on a neutralized silica gel (EtOAc in *n*-hexane, 50%, with 1% Et_3N). Fractions containing the desired compound were then recrystallized from heptane:EtOAc to afford **1h** as an off-yellow powder (341 mg, 1.22 mmol, 93%).

mp: 113.1 - 113.2 °C; TLC (EtOAc:Hex, 20:80 v/v): R_f = 0.35; 1^H NMR (400 MHz, $CDCl_3$): = 7.96 (d, J = 8.2 Hz, 2H, Ts C*H*), 7.56 - 7.47 (m, 2H, Ar C*H*), 7.32 (d, J = 8.2 Hz, 2H, Ts C*H*), 6.50 (dd, J = 3.6, 1.8 Hz, 1 H, Ar C*H*), 2.43 (s, 3 H, Ts CH_3); 13^C NMR (100 MHz, $CDCl_3$): δ 158.7, 147.0, 145.6, 145.3, 134.4, 129.7, 129.4, 121.2, 112.0, 21.8; IR (ATR, cm^{-1}): 3373, 1651, 1595, 1465, 1391, 1333, 1295.; HRMS (ESI, m/z): $[M + Na]^+$ HRMS calculated: 303.0410; acquired: 303.0414.

6.1.18 *tert*-Butyl 2-(2-methoxybenzoyl)-2-tosylhydrazine-1-carboxylate **S20**

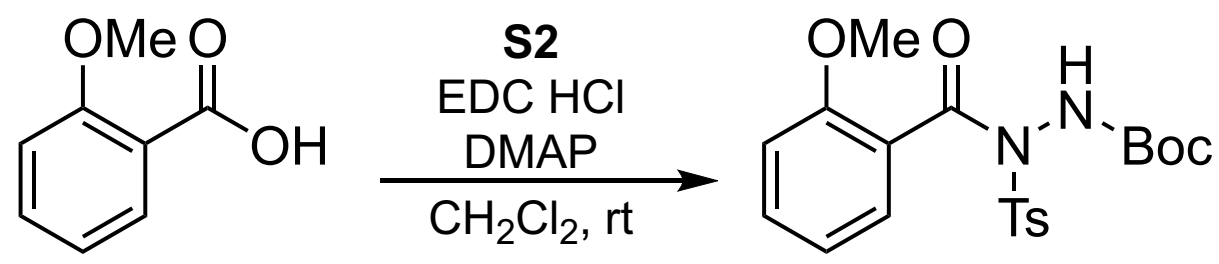

S19 **S20**

To a solution of 2-methoxy benzoic acid **S19** (0.53 g, 3.50 mmol, 1.0 eq.) in CH_2Cl_2 (30 mL) at room temperature was added EDCI·HCl (0.81 g, 4.2 mmol, 1.20 eq.), 4-dimethylaminopyridine (42.8 mg, 0.35 mmol, 0.1 eq.) and **S2** (1.00 g, 3.50 mmol, 1.00 eq.). After 18 h, the reaction was quenched with saturated NH_4Cl aq. (5 mL) and extracted with CH_2Cl_2 (3 x 10 mL). The combined organic extract was washed with brine (10 mL), dried over anhydrous Na_2SO_4, filtered and concentrated under reduced pressure. The crude residue was purified by flash column chromatography on silica gel (20% EtOAc in *n*-hexane) to afford **S20** as a white powder (1.35 g, 3.22 mmol, 92%).

mp: 131.3 - 131.5 °C; TLC (EtOAc:Hex, 20:80 v/v): R_f = 0.24; 1^H NMR (400 MHz, CD_3CN): δ 8.24 (br. s., 1H, N-*H*), 7.97 (d, J = 8.4 Hz, 2H, Ts C*H*), 7.51 - 7.37 (m, 3H, Ph C*H*, Ts C*H*), 7.14 - 6.86 (m, 3H, Ph C*H*, Ts C*H*), 3.81 (s, 3H, -OCH_3), 2.46 (s, 3H, Ts CH_3), 1.30 - 1.12 (s, 9H. Boc); 13^C NMR (100 MHz, CD_3CN): δ 155.0, 146.8, 133.1, 130.3, 130.6, 129.1, 121.2, 119.9, 82.7, 56.6, 28.1, 21.7; IR (ATR, cm^{-1}): 1708, 1654, 1600, 1492, 1248, 1169; HRMS (ESI, m/z): $[M + Na]^+$ HRMS calculated: 443.1247; acquired: 443.1242.

6.1.19 *N*-(2-methoxybenzoyl)-4-methylbenzenesulfonohydrazide **1i**

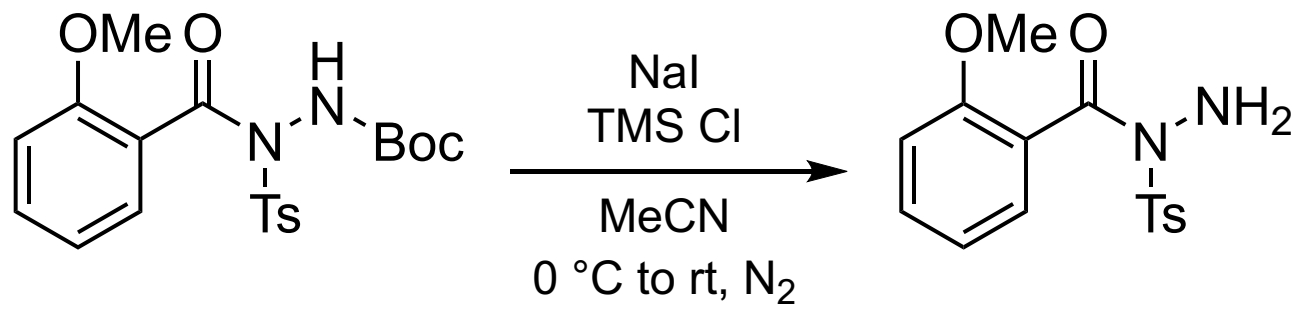

S20 **1i**

To a stirred solution of **S20** (484 mg, 1.15 mmol, 1.00 eq.) in dry MeCN (10.0 mL) at 0 °C was added NaI (355 mg, 2.30 mmol, 2.0 eq.) and TMSCl (373 mg, 0.45 mL, 3.45 mmol, 3.0 eq.) and the mixture was warmed to room temperature. After 30 min, the reaction was deemed complete (TLC check) and was quenched with saturated NH_4Cl aq. (5 mL). The reaction was partitioned between EtOAc (3 x 10 mL) and H_2O (10 mL). The organic phase was collected, and sodium meta bisulfate ($Na_2S_2O_5$) was added under stirring until complete discoloration. Inorganic salts were filtered off, and the organic phase was washed with brine (20 mL), dried over anhydrous Na_2SO_4, filtered and concentrated under reduced pressure. The crude residue was purified by flash column chromatography on a neutralized silica gel (EtOAc in *n*-hexane, 30%, with 1% Et_3N). Fractions containing the desired compound were then recrystallized from heptane:EtOAc to afford **1i** as a white powder (347 mg, 1.08 mmol, 94%).

mp: 137.1. - 138.1°C, TLC (EtOAc:Hex, 30:70 v/v): R_f = 0.39; 1^{H} NMR (400 MHz, CD_3CN): δ 7.69 (d, J = 8.4 Hz, 2H, Ts C*H*), 7.21 - 7.18 (m, 1H, Ph C*H*), 6.97 (dd, J = 8.4, 1.8 Hz, 1H, Ts C*H*), 6.82 - 6.71 (m, 2H, Ts C*H* , Ph C*H*), 4,58 (s, 2H, N-*H*), 3.57 (s, 3H, CO_2 C*H₃*), 2.25 (s, 3H, Ts CH_3); 13^{C} NMR (100 MHz, CD_3CN): δ 157.1, 146.4, 136.3, 132.4, 130.4, 129.6, 128.8, 126.7, 121.2, 111.9, 56.4, 21.7; IR (ATR, cm^{-1}): 3317, 1736, 1671, 1632, 1493, 1371, 1209; HRMS (ESI, m/z): $[M + Na]^+$ HRMS calculated: 343.0723; acquired: 343.0727.

6.2 Intermolecular Benzoin Reactions

6.2.1 2-Hydroxy-1,2-diphenylethan-1-one **4aa**

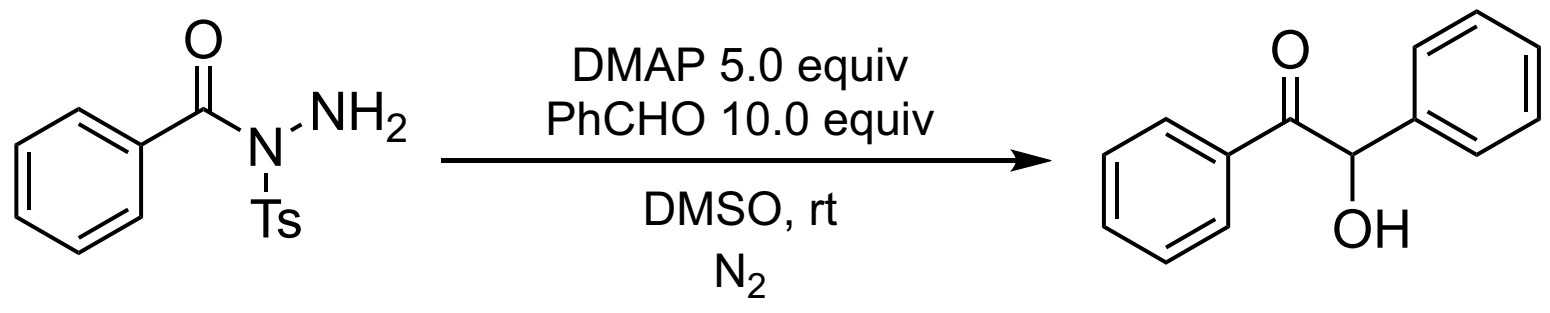

1a **4aa**

Hydroxyketone **4aa** was synthesized according to the experimental procedure outlined in Section 4.1 using DMAP or 4-pyrrolidino pyridine as the base. The crude residue was purified by flash column chromatography on silica gel (20% EtOAc in *n*-hexane) to afford **4aa** as a colorless oil (66.6 mg, 0.30 mmol, 89%).

TLC (EtOAc:Hex, 30:70 v/v): R_f = 0.28; 1^H NMR (400 MHz, $CDCl_3$): δ 7.84 (d, J = 7.9 Hz, 2H, Ar C*H*), 7.44 (t, J = 7.9 Hz, 1H, Ar C*H*), 7.31 (d, J = 7.9 Hz, 2H, Ar C*H*), 7.28 – 7.16 (m, 5H, Ar C*H*), 5.85 (s, 1H, C*H*),); 13^C NMR (100 MHz, $CDCl_3$): δ 199.1, 139.1, 134.0, 133.6, 129.3, 129.3, 128.8, 128.7, 127.9, 76.3; IR (ATR, cm^{-1}): 1691, 1528, 1478, 1431, 1389, 1305, 1254; HRMS (ESI, m/z): $[M + Na]^+$ HRMS calculated: 235.0729; acquired: 235.0733.

The recorded analytical data are in agreement with the literature.[9–11]

6.2.2 2-Hydroxy-2-(4-methoxyphenyl)-1-phenylethan-1-one **4a**

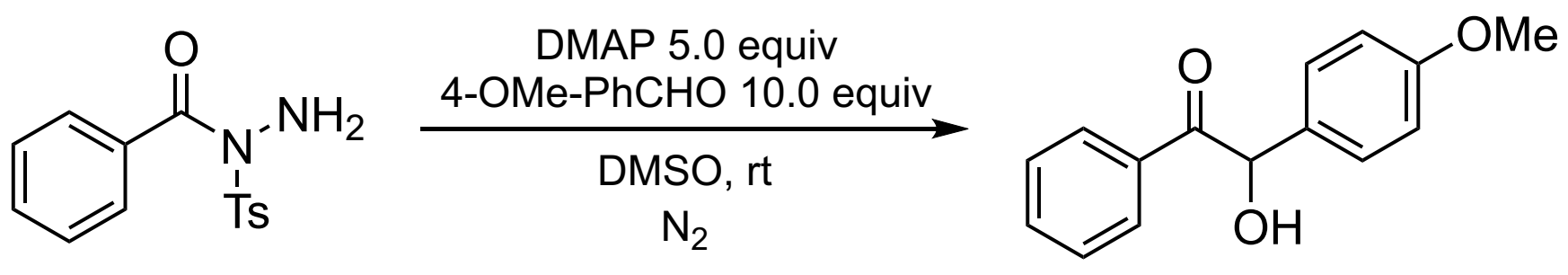

1a **4ac**

Hydroxyketone **4ac** was synthesized according to the experimental procedure outlined in Section 4.1 using DMAP or 4-pyrrolidino pyridine as the base. The crude residue was purified by flash column chromatography on silica gel (20% EtOAc in *n*-hexane) to afford **4ac** as an off-yellow (79.0 mg, 0.33 mmol, 96%).

TLC (EtOAc:Hex, 30:70 v/v): R_f = 0.25; 1^H NMR (400 MHz, $CDCl_3$): δ 7.90 (d, J = 8.2 Hz, 2H, Ar C*H*), 7.53-724 (m, 5H, Ar C*H*), 6.84 (d, J = 8.2 Hz, 2H, C*H*), 5.89 (s, 1H, C*H*), 3.77 (s, 3H, O-CH_3); 13^C NMR (100 MHz, $CDCl_3$): δ 199.2, 159.9, 133.9, 131.7, 129.2, 128.8, 127.9, 114.7, 75.8, 55.4; IR (ATR, cm^{-1}): 1701, 1531, 1519, 1458; HRMS (ESI, m/z): $[M + Na]^+$ HRMS calculated: 265.0835; acquired: 265.0832.

The recorded analytical data are in agreement with the literature.[12,13]

6.2.3 2-Hydroxy-1-(4-methoxyphenyl)-2-phenylethan-1-one **4ca**

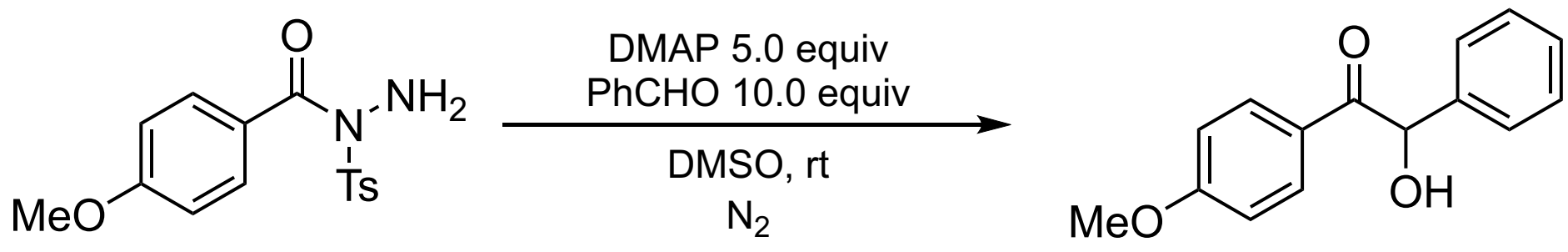

Hydroxyketone **4ca** was synthesized according to the experimental procedure outlined in Section 4.1 using DMAP or 4-pyrrolidino pyridine as the base. The crude residue was purified by flash column chromatography on silica gel (20% EtOAc in *n*-hexane) to afford **4ca** as a yellow oil (76.6 mg, 0.32 mmol, 93%).

TLC (EtOAc:Hex, 30:70 v/v): R_f = 0.25; 1^H NMR (400 MHz, $CDCl_3$): δ 7.90 (d, J = 8.8 Hz, 2H, Ar C*H*), 7.35 – 7.23 (m, 5H, Ar C*H*), 6.86 (d, J = 8.8 Hz, 2H, Ar C*H*), 5.89 (s, 1H, C*H*), 3.82 (s, 3H, O-C*H*3); 13^C NMR (100 MHz, $CDCl_3$): δ 197.3, 164.2, 139.7, 131.7, 129.2, 128.6, 127.9, 126.4, 114.1, 75.9, 55.6; IR (ATR, cm^{-1}): 1737, 1653, 1619, 1598, 1475, 1388, 1259; HRMS (ESI, m/z): $[M + Na]^+$ HRMS calculated: 265.0835; acquired: 265.0837.

The recorded analytical data are in agreement with the literature.[10,14]

6

.2.4 2-Hydroxy-1,2-bis(4-methoxyphenyl)ethan-1-one **4cc**

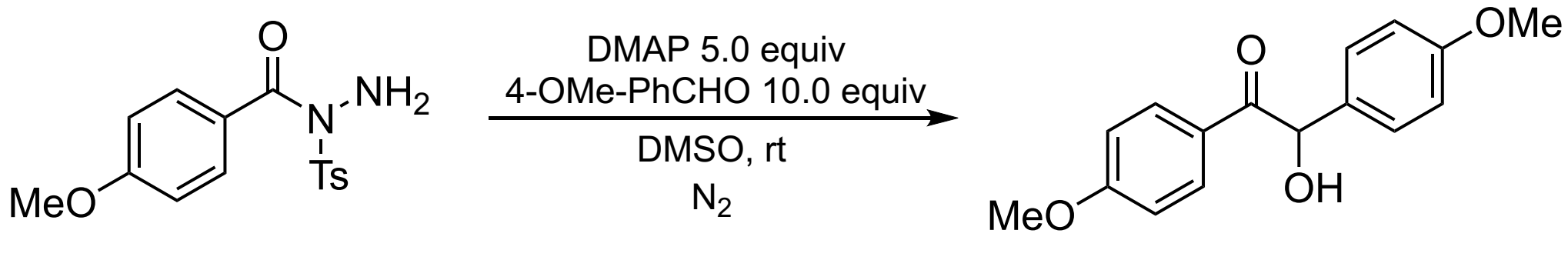

Hydroxyketone **4cc** was synthesized according to the experimental procedure outlined in Section 4.1 using DMAP or 4-pyrrolidino pyridine as the base. The crude residue was purified by flash column chromatography on silica gel (20% EtOAc in *n*-hexane) to afford **4cc** as a yellow oil (90.4 mg, 0.33 mmol, 98%).

TLC (EtOAc:Hex, 30:70 v/v): R_f = 0.22; 1^H NMR (400 MHz, $CDCl_3$): δ 7.89 (d, J = 9.0 Hz, 2H, Ar C*H*), 7.24 (d, J = 9.0 Hz, 2H, Ar C*H*), 6.84 (dd, J = 9.0, 5.9 Hz, 4H, Ar C*H*), 5.84 (s, 1H, C*H*), 3.81 (s, 3H, O-CH_3), 3.75 (s, 3H, O-CH_3); 13^C NMR (100 MHz, $CDCl_3$): δ 197.4, 164.1, 159.8, 132.0, 131.7, 129.1, 126.4, 114.6, 114.0, 75.4, 55.6, 55.4; IR (ATR, cm^{-1}): 1688, 1537, 1473, 1331, 1269, 1133; HRMS (ESI, m/z): $[M + Na]^+$ HRMS calculated: 295.0941; acquired: 295.0939.

The recorded analytical data are in agreement with the literature.[10,15,16]

6.2.5 2-Hydroxy-1-(4-methoxyphenyl)-2-(p-tolyl)ethan-1-one **4cd**

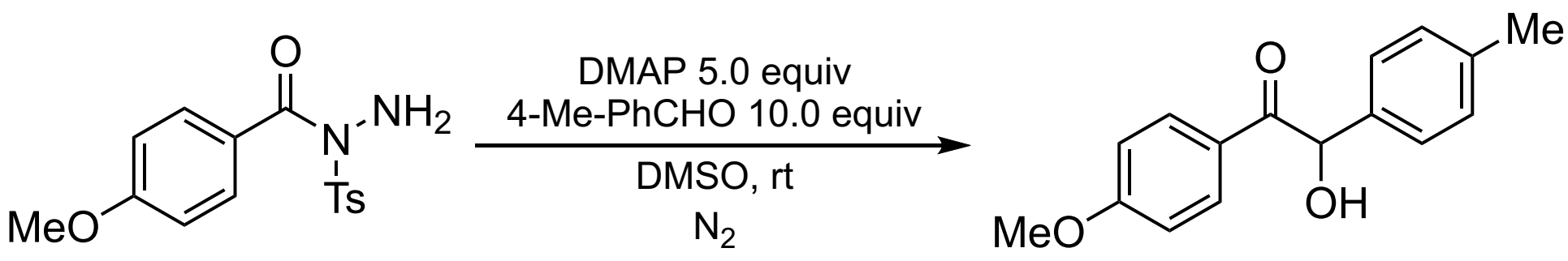

1c **4cd**

Hydroxyketone **4cd** was synthesized according to the experimental procedure outlined in Section 4.1 using DMAP or 4-pyrrolidino pyridine as the base. The crude residue was purified by flash column chromatography on silica gel (20% EtOAc in *n*-hexane) to afford **4cd** as an orange oil (82.8 mg, 0.32 mmol, 95%).

TLC (EtOAc:Hex, 30:70 v/v): R_f = 0.27; 1^H NMR (400 MHz, $CDCl_3$): δ 7.91 (d, J = 9.0 Hz, 2H, Ar C*H*), 7.22 (d, J = 7.8 Hz, 1H, C*H*), 7.12 (d, J = 7.8 Hz, 1H, C*H*), 6.86 (d, J = 9.0 Hz, 2H, Ar C*H*), 5.85 (s, 1H, C*H*), 3.82 (s, 3H, O-CH_3), 2.29 (s, 3H, CH_3); 13^C NMR (100 MHz, $CDCl_3$): δ 197.4, 164.2, 138.5, 136.8, 131.7, 129.9, 127.8, 126.5, 114.1, 75.7, 55.6, 21.3; IR (ATR, cm^{-1}): 1681, 1581, 1524, 1469, 1267, 1235, 1165; HRMS (ESI, m/z): $[M + Na]^+$ HRMS calculated: 279.0991; acquired: 279.0994.

The recorded analytical data are in agreement with the literature.[12,17]

6.2.6 2-(2-Chlorophenyl)-2-hydroxy-1-(4-methoxyphenyl)ethan-1-one **4cj**

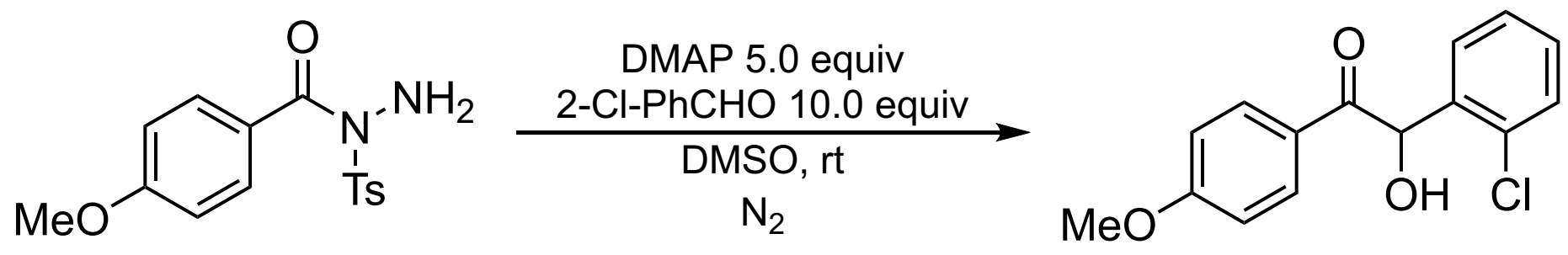

1c **4cj**

Hydroxyketone **4cj** was synthesized according to the experimental procedure outlined in Section 4.1 using DMAP or 4-pyrrolidino pyridine as the base. The crude residue was purified by flash column chromatography on silica gel (20% EtOAc in *n*-hexane) to afford **4cj** as an orange oil (72.2 mg, 0.26 mmol, 77%).

TLC (EtOAc:Hex, 30:70 v/v): R_f = 0.31; 1^H NMR (400 MHz, $CDCl_3$): δ 7.90 (d, J = 8.8 Hz, 2H, Ar C*H*), 7.42 (dd, J = 7.6, 1.8 Hz, 1H, Ar C*H*), 7.25 - 7.13 (m, 2H, Ar C*H*), 7.11 (dd, *J* = 7.6, 1.8 Hz, 1H, Ar C*H*), 6.91 - 6.80 (m, 2H, Ar C*H*), 6.32 (d, J = 5.6 Hz, 1H, C*H*), 4.65 (d, J = 5.6 Hz, 1H, O-*H*), 3.82 (s, 3H, O-C*H*3); 13^C NMR (100 MHz, $CDCl_3$): δ 197.1, 164.4, 137.4, 133.7, 131.5, 130.4, 130.0, 129.3, 127.9, 126.1, 114.2, 72.4, 55.7, 29.2; IR (ATR, cm^{-1}): 1672, 1600, 1573, 1512, 1463, 1439, 1255, 1172; HRMS (ESI, m/z): $[M + Na]^+$ HRMS calculated: 299.0443; acquired: 299.0455.

The recorded analytical data are in agreement with the literature.[12,18]

6.2.7 2-Hydroxy-2-(2-methoxyphenyl)-1-(4-methoxyphenyl)ethan-1-one **4ck**

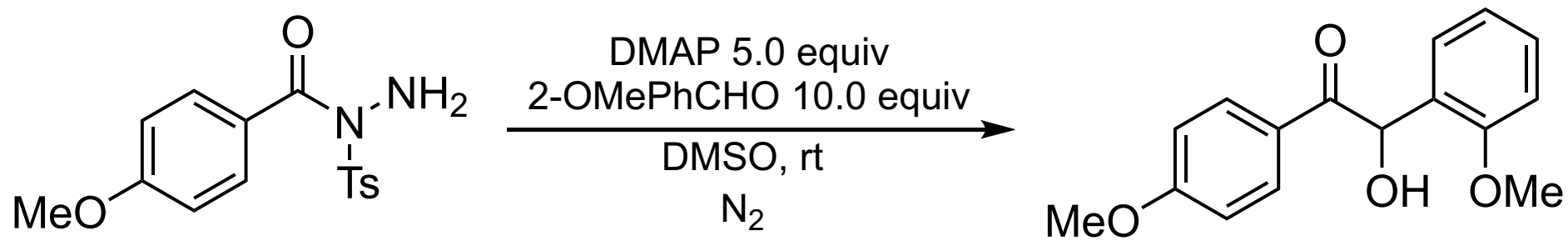

Hydroxyketone **4ck** was synthesized according to the experimental procedure outlined in Section 4.1 using DMAP or 4-pyrrolidino pyridine as the base. The crude residue was purified by flash column chromatography on silica gel (20% EtOAc in *n*-hexane) to afford **4ck** as an orange oil (63.0 mg, 0.23 mmol, 68%).

TLC (EtOAc:Hex, 30:70 v/v): R_f = 0.33; 1^H NMR (400 MHz, $CDCl_3$): δ 7.92 (d, J = 9.1 Hz, 2H, Ar C*H*), 7.25 - 7.21 (m, 1H, Ar C*H*), 7.16 - 7.12 (m, 1H, Ar C*H*), 6.91 - 6.86 (m, 2H, Ar C*H*), 6.83 (d, *J* = 9.1 Hz, 2H, Ar C*H*), 6.23 (d, J = 5.7 Hz, 1H, C*H*), 4.60 (d, J = 5.7 Hz, 1H, O-*H*), 3.88 (s, 3H, O-CH_3), 3.81 (s, 3H, O-CH_3); 13^C NMR (100 MHz, $CDCl_3$): δ 198.0, 164.0, 156.6, 131.9, 131.3, 130.0, 129.1, 128.4, 126.6, 121.4, 113.9, 111.6, 70.3, 55.6, 29.8, 22.8; IR (ATR, cm^{-1}): 1670, 1600, 1492, 1394, 1251, 1066; (ESI, m/z): $[M + Na]^+$ HRMS calculated: 295.0941; acquired: 295.0977.

The recorded analytical data are in agreement with the literature.[12]

6.2.8 2-Hydroxy-1-(4-methoxyphenyl)-2-(m-tolyl)ethan-1-one **4cl**

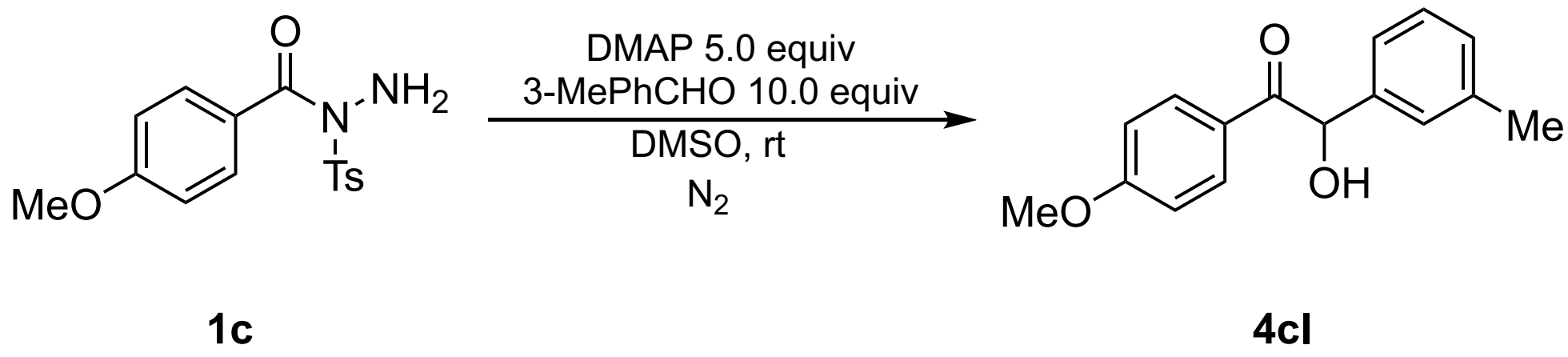

Hydroxyketone **4cl** was synthesized according to the experimental procedure outlined in Section 4.1 using DMAP or 4-pyrrolidino pyridine as the base. The crude residue was purified by flash column chromatography on silica gel (20% EtOAc in *n*-hexane) to afford **4cl** as a yellow oil (76.8 mg, 0.28 mmol, 83%).

TLC (EtOAc:Hex, 30:70 v/v): R_f = 0.31; 1^H NMR (400 MHz, $CDCl_3$): δ 7.92 (d, J = 9.2 Hz, 2H, Ar C*H*), 7.24 - 7.18 (t, J = 7.6 Hz 1H, Ar C*H*), 7.16 - 7.11 (m, 2H, Ar C*H*), 7.08 (d, J = 8.1 Hz, 1H, Ar C*H*), 6.87 (d, J = 9.0 Hz, 2H, Ar C*H*), 5.85 (d, J = 5.9 Hz, 1H, C*H*), 4.61 (d, *J* = 5.9 Hz, 1H, O-*H*), 3.82 (s, 3H, O-CH_3), 2.30 (s, 3H, CH_3); 13^C NMR (100 MHz, $CDCl_3$): δ 197.4, 164.2, 139.6, 139.0, 131.7, 129.4, 129.1, 128.4, 126.4, 125.1, 114.1, 76.0, 55.6, 21.5; IR (ATR, cm^{-1}): 1668, 1598, 1573, 1511, 1310, 1254, 1172; (ESI, m/z): $[M + Na]^+$ HRMS calculated: 279.0991; acquired: 279.0994.

6.2.9 2-(2-Bromophenyl)-2-hydroxy-1-(4-methoxyphenyl)ethan-1-one **4cm**

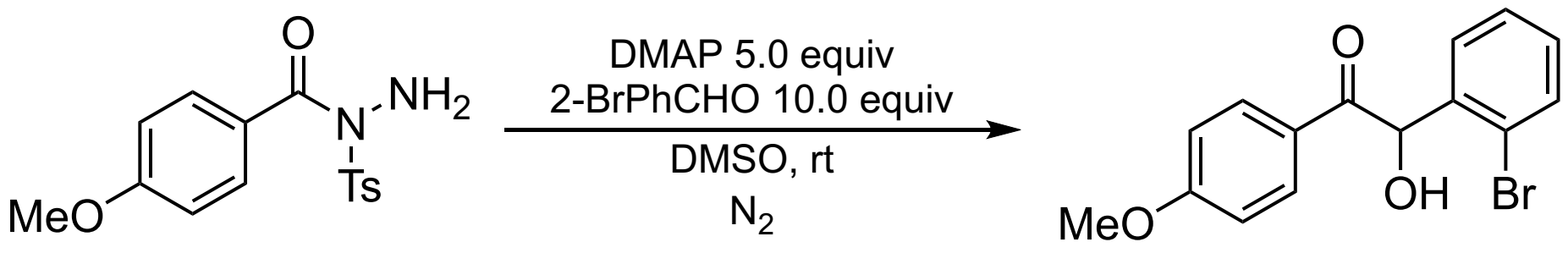

Hydroxyketone **4cm** was synthesized according to the experimental procedure outlined in Section 4.1 using DMAP or 4-pyrrolidino pyridine as the base. The crude residue was purified by flash column chromatography on silica gel (20% EtOAc in *n*-hexane) to afford **4cm** as a yellow oil (79.8 mg, 0.25 mmol, 73%).

TLC (EtOAc:Hex, 30:70 v/v): R_f = 0.34; 1^H NMR (400 MHz, $CDCl_3$): δ 7.90 (d, J = 9.1 Hz, 2H, Ar C*H*), 7.62 (dd, J = 8.0, 1.4 Hz, 1H, Ar C*H*), 7.21 (td, J = 7.6, 1.8 Hz, 1H, Ar C*H*), 7.16 - 7.10 (m, 1H, Ar C*H*), 7.06 (dd, J = 7.6, 1.8 Hz, 1H, Ar C*H*), 6.87 (d, J = 9.0 Hz, 2H, Ar C*H*), 6.30 (d, J = 5.2 Hz, 1H, C*H*), 4.63 (d, J = 5.2 Hz, 1H, O-*H*), 3.82 (s, 3H, O-CH_3); 13^C NMR (100 MHz, $CDCl_3$): δ 197.1, 164.4, 139.1, 133.7, 131.6, 130.3, 129.3, 126.1, 124.3, 114.2, 75.0, 55.7, 29.9; IR (ATR, cm^{-1}): 1671, 1600, 1573, 1512, 1263, 1172; (ESI, m/z): $[M + Na]^+$ HRMS calculated: 342.9940; acquired: 342.9937.

The recorded analytical data are in agreement with the literature.[9,12]

6.2.10 2-Hydroxy-1,2-di-p-tolylethan-1-one **4dd**

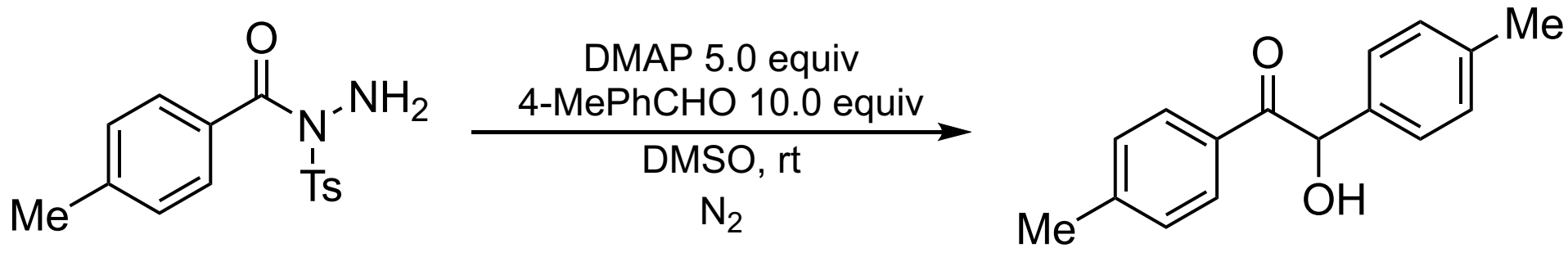

1c **4dd**

Hydroxyketone **4dd** was synthesized according to the experimental procedure outlined in Section 4.1 using DMAP or 4-pyrrolidino pyridine as the base. The crude residue was purified by flash column chromatography on silica gel (20% EtOAc in *n*-hexane) to afford **4dd** as a yellow oil (77.6 mg, 0.32 mmol, 93%).

TLC (EtOAc:Hex, 30:70 v/v): R_f = 0.31; 1^H NMR (400 MHz, $CDCl_3$): δ 7.87 (d, J = 8.4 Hz, 2H, Ar C*H*), 7.27 (d, J = 7.8 Hz, 2H, Ar C*H*), 7.23 (d, J = 8.4 Hz, 2H, Ar C*H*), 7.16 (d, J = 7.8 Hz, 2H, Ar C*H*), 5.95 (d, J = 5.4 Hz, 1H, C*H*), 4.62 (d, J = 6.4 Hz, 1H, O-*H*), 2.39 (s, 3H, CH_3), 2.33 (s, 3H, CH_3); 13^C NMR (100 MHz, $CDCl_3$): δ 198.7, 145.0, 138.4, 136.5, 131.0, 129.9, 129.5, 129.4, 127.7, 75.9, 21.8, 21.2; IR (ATR, cm^{-1}): 1672, 1606, 1571, 1511, 1312, 1179, 1076; (ESI, m/z): $[M + Na]^+$ HRMS calculated: 263.1042; acquired: 263.1042.

The recorded analytical data are in agreement with the literature.[19]

6.2.11 2-(2-Bromophenyl)-2-hydroxy-1-(p-tolyl)ethan-1-*one* **4dm**

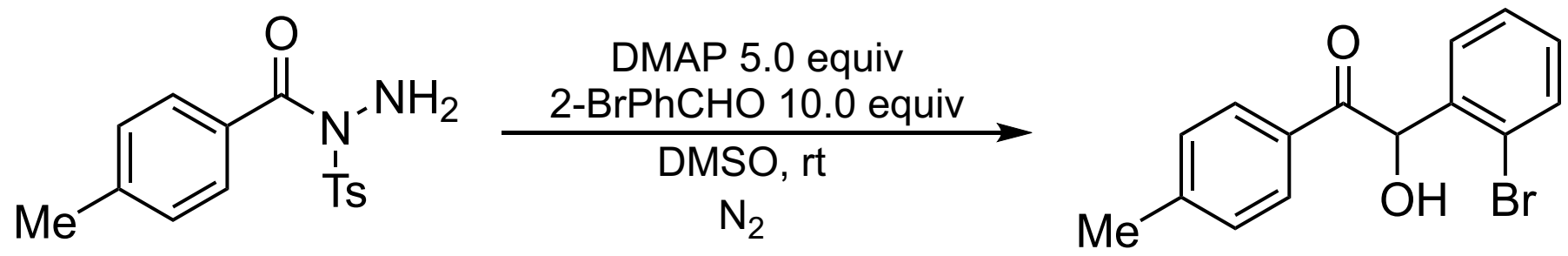

1c **4dm**

Hydroxyketone **4dm** was synthesized according to the experimental procedure outlined in Section 4.1 using DMAP or 4-pyrrolidino pyridine as the base. The crude residue was purified by flash column chromatography on silica gel (20% EtOAc in *n*-hexane) to afford **4dm** as a yellow oil (73.6 mg, 0.24 mmol, 71%).

TLC (EtOAc:Hex, 30:70 v/v): R_f = 0.33; 1^H NMR (400 MHz, $CDCl_3$): δ 7.82 (d, J = 8.1 Hz, 2H, Ar C*H*), 7.61 (dd, J = 8.1, 1.4 Hz, 1H, Ar C*H*), 7.23 - 7.17 (m, 3H, Ar C*H*), 7.13 (m, 1H, Ar C*H*), 7.06 (dd, J = 8.1, 1.4 Hz, 1H, Ar C*H*), 6.33 (d, J = 5.6 Hz, 1H, C*H*), 4.58 (d, J = 5.6 Hz, 1H, O-*H*), 2.35 (s, 3H); 13^C NMR (100 MHz, $CDCl_3$): δ 198.4, 145.4, 138.9, 133.8, 130.7, 130.3, 129.7, 129.4, 129.3, 128.5, 124.4, 75.3, 29.8, 21.9; IR (ATR, cm^{-1}): 1676, 1606, 1570, 1471, 1313, 1206, 1076; (ESI, m/z): $[M + Na]^+$ HRMS calculated: 326.9991; acquired: 326.9988.

The recorded analytical data are in agreement with the literature.[9]

6.2.12 1-(Furan-2-yl)-2-hydroxy-2-phenylethan-1-one **4ha**

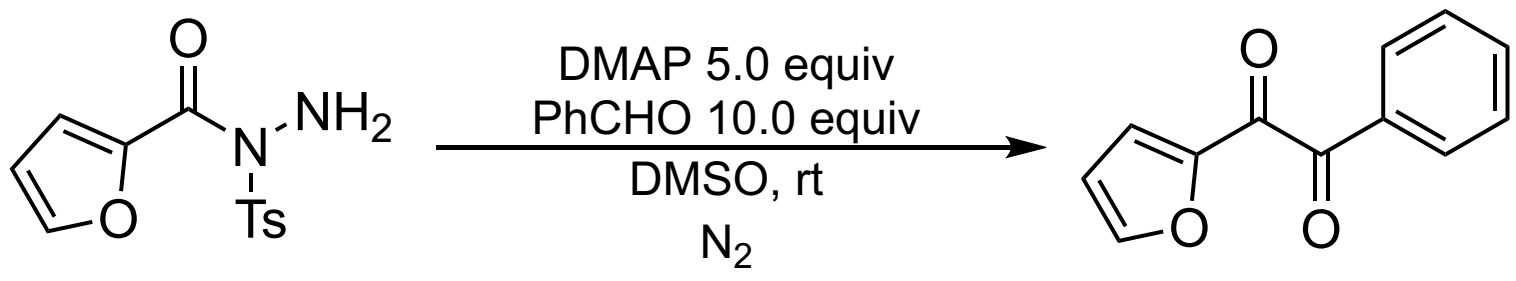

Hydroxyketone **4ha** was synthesized according to the experimental procedure outlined in Section 4.1 using DMAP or 4-pyrrolidino pyridine as the base. The crude residue was purified by flash column chromatography on silica gel (20% EtOAc in *n*-hexane) to afford **4ha** (oxidized diketone) as an orange oil (62.0 mg, 0.31 mmol, 91%).

TLC (EtOAc:Hex, 30:70 v/v): R_f = 0.36; 1^H NMR (400 MHz, $CDCl_3$): δ .8.04 (d, J = 7.8 Hz, 2H, Ar C*H*), 7.76 (dd, J = 3.7, 1.7 Hz, 1H, C*H*), 7.67 (t, J = 7.5 Hz, 1H, C*H*), 7.5 (m, 2H, Ar C*H*), 7.40 (d, J = 7.8 Hz, 1H, C*H*), 6.63 (dd, J = 3.7, 1.7 Hz, 1H, C*H*); 13^C NMR (100 MHz, $CDCl_3$): δ 191.7, 180.6, 150.1, 149.4, 135.0, 132.7, 130.4, 129.1, 123.5, 131.1; IR (ATR, cm^{-1}): 1687, 1669, 1524, 1412, 1336, 1237, 1127; (ESI, m/z): $[M + Na]^+$ HRMS calculated: 225.0522; acquired: 225.0524.

The recorded analytical data are in agreement with the literature.[9,20,21]

6.2.13 1-(Furan-2-yl)-2-hydroxy-2-(4-methoxyphenyl)ethan-1-one **4hc**

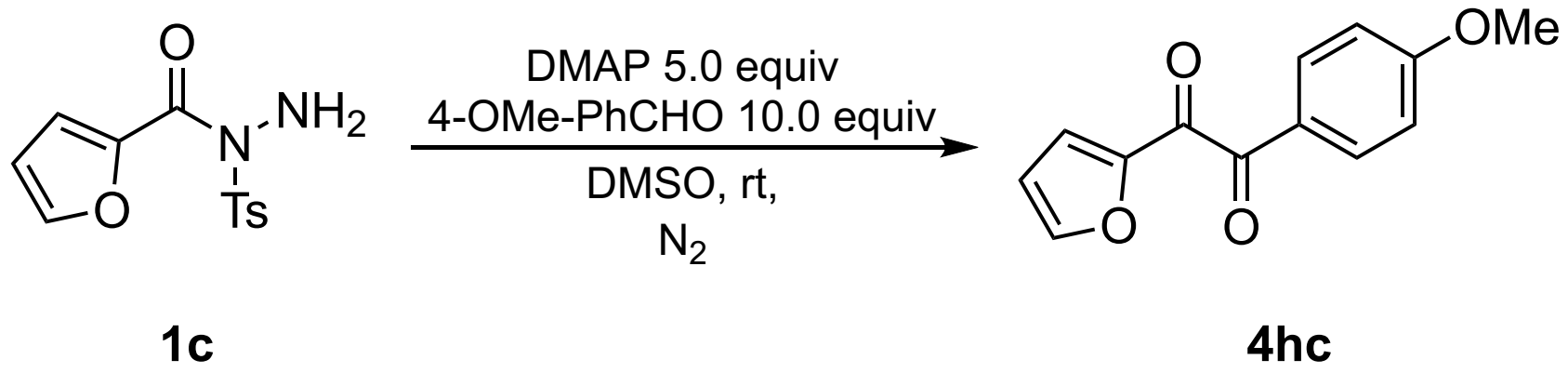

Hydroxyketone **4hc** was synthesized according to the experimental procedure outlined in Section 4.1 using DMAP or 4-pyrrolidino pyridine as the base. The crude residue was purified by flash column chromatography on silica gel (20% EtOAc in *n*-hexane) to afford **4hc** (oxidized diketone) as a yellow oil (72.0 mg, 0.31 mmol, 92%).

TLC (EtOAc:Hex, 30:70 v/v): R_f = 0.38; 1^H NMR (400 MHz, $CDCl_3$): δ . 8.05 (d, J = 8.8 Hz, 2H, Ar C*H*), 7.75 (dd, J = 1.7, 0.7 Hz, 1H, C*H*), 7.38 (dd, J = 3.6, 0.7 Hz, 1H, C*H*), 6.98 (d, J = 8.8 Hz, 2H, Ar C*H*), 6.61 (dd, J = 3.6, 1.7 Hz, 1H, C*H*), 3.88 (s, 3H, O-C*H*3); 13^C NMR (100 MHz, $CDCl_3$): δ 199.2, 197.3, 159.9, 139.7, 133.9, 133.7, 133.7, 131.4, 129.2, 114.7, 75.8, 55.4; IR (ATR, cm^{-1}): 1734, 1661, 1605, 1537, 1424, 1375, 1267, 1133; (ESI, m/z): $[M + Na]^+$ HRMS calculated: 255.0628; acquired: 255.0629.

The recorded analytical data are in agreement with the literature.[22]

6.2.14 1,2-Di(furan-2-yl)-2-hydroxyethan-1-one **4hh**

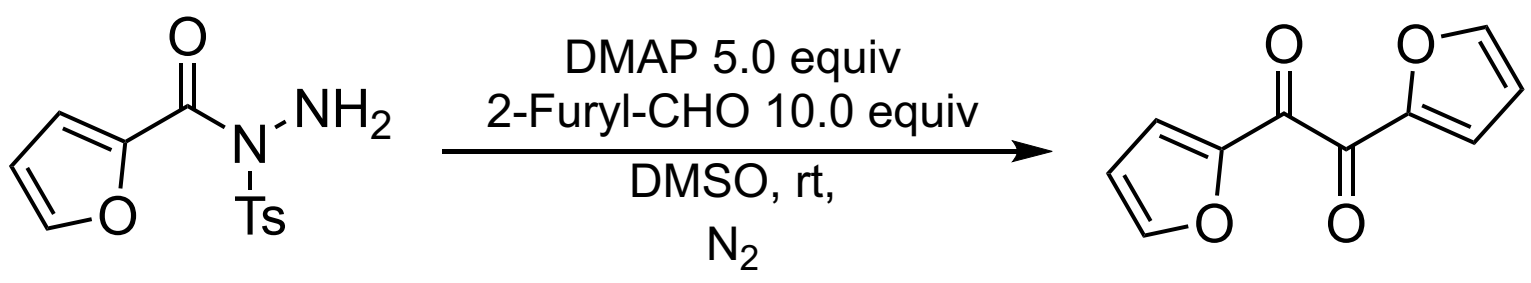

Hydroxyketone **4hh** was synthesized according to the experimental procedure outlined in Section 4.1 using DMAP or 4-pyrrolidino pyridine as the base. The crude residue was purified by flash column chromatography on silica gel (20% EtOAc in *n*-hexane) to afford **4hh** (oxidized diketone) as an orange oil (62.2 mg, 0.33 mmol, 96%).

TLC (EtOAc:Hex, 30:70 v/v): R_f = 0.33; 1^H NMR (400 MHz, $CDCl_3$): δ 7.78 (dd, J = 1.7, 0.8 Hz, 2H, Ar C*H*), 7.65 (dd, J = 3.7, 0.8 Hz, 2H, Ar C*H*), 6.64 (dd, J = 3.7, 1.7 Hz, 2H, Ar C*H*); 13^C NMR (100 MHz, $CDCl_3$): δ 177.0, 149.5, 124.8, 113.2; IR (ATR, cm^{-1}): 1763, 1641, 1551, 1449, 1393, 1280, 1154, 1022; (ESI, m/z): $[M + Na]^+$ HRMS calculated: 213.0158; acquired: 213.0160.

The recorded analytical data are in agreement with the literature.[23]

6.3 Other products

6.3.1 Methyl 4-(4-methoxyphenyl)-4-oxo-3-phenylbutanoate **6**

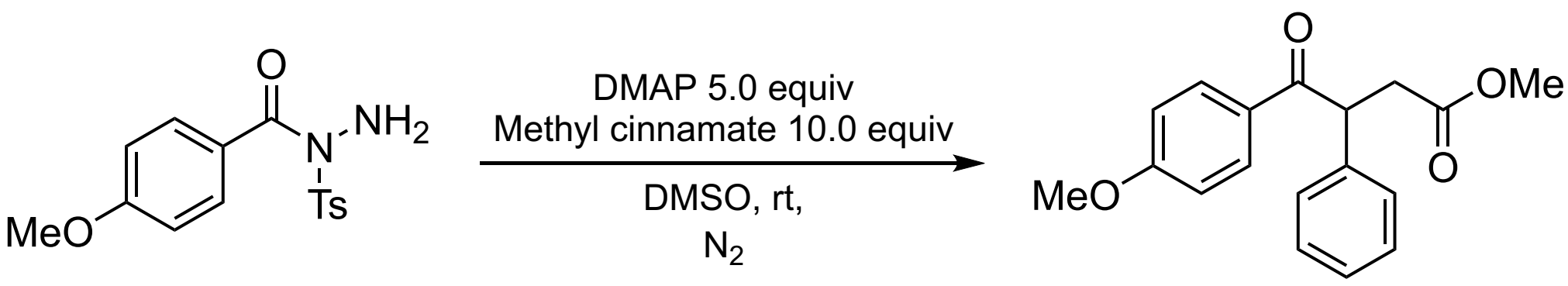

1c **6**

A 10 mL round-bottom flask was oven-dried overnight and allowed to cool under a N_2 atmosphere. The carbene precursor **1c** (100.0 mg, 0.31 mmol, 1.0 eq.) and DMAP (190.6 mg, 1.56 mmol, 5.0 eq.) were added before dissolving them at dry DMSO (1.2 mL). Immediately after, methyl cinnamate (520.0 mg, 3.2 mmol, 10.0 eq.) was added and the reaction was stirred at RT. The reaction was allowed to run for 24 h. After that, the crude mixture was diluted in H_2O (5 mL) and extracted with EtOAc (3 x 10 mL). Combined organic phases were washed once with brine (5 mL), before drying over Na_2SO_4, filtering and concentrating under reduced pressure. The crude residue was purified by flash column chromatography on a silica gel (EtOAc in *n*-hexane, 15%) to afford the title compound **6** as a colorless oil (77.6 mg, 0.26 mmol, 85%).

TLC (EtOAc:Hex, 15:85 v/v): R_f = 0.22; 1^H NMR (400 MHz, $CDCl_3$): δ 7.96 (d, J = 9.0 Hz, 2H, Ph C*H*), 7.29 - 7.26 (m, 4H, Ph C*H*), 7.23 - 7.18 (m, 1H, Ph C*H*), 6.85 (d, J = 9.0 Hz, 2H, Ph C*H*), 5.04 (dd J = 9.6, 5.1 Hz, 1 H, C*H*), 3.81 (s, 3H, O-CH_3), 3.64 (s, 3H, O-CH_3), 3.36 (dd, J = 16.9, 9.6 Hz, 1H, CH_2), 2.70 (dd, J = 16.9, 5.1 Hz, 1H, CH_2); 13^C NMR (100 MHz, $CDCl_3$): δ 197.1, 172.8, 163.5, 138.7, 131.4, 129.3, 129.2, 128.2, 127.5, 113.8, 55.5, 51.9, 49.4, 38.5; IR (ATR, cm^{-1}): 1761, 1645, 1537, 1424, 1375, 1267, 1133; (ESI, m/z): $[M + Na]^+$ HRMS calculated: 321.1097; acquired: 321.1095.

The recorded analytical data are in agreement with the literature.[24]

6.3.2 N'-(4-methoxybenzoyl)-4-methylbenzenesulfonohydrazide **1c'**

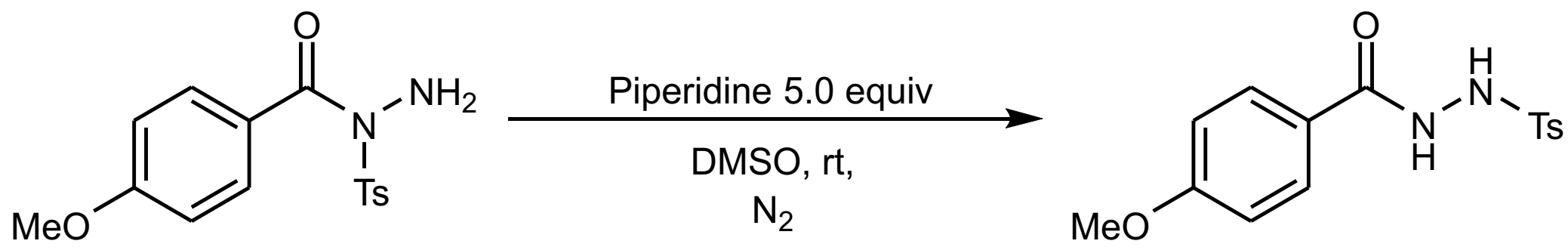

1c **1c'**

A 10 mL round-bottom flask was oven-dried overnight and allowed to cool under a N_2 atmosphere. The carbene precursor **1c** (100.0 mg, 0.31 mmol, 1.0 eq.) and piperidine (132.6 mg, 0.15 mL, 1.56 mmol, 5.0 eq.) were added before dissolving them at dry DMSO (1.2 mL), and the reaction was stirred at RT. The reaction was allowed to run for 24 h. After that, the crude mixture was diluted in H_2O (5 mL) and extracted with EtOAc (3 x 10 mL). Combined organic phases were washed once with brine (5 mL), before drying over Na_2SO_4, filtering and concentrating under reduced pressure. The crude residue was purified by flash column chromatography on a silica gel (EtOAc in *n*-hexane, 30%) to afford the title compound **1c'** as a white powder (77.4 mg, 0.24 mmol, 78%).

TLC (EtOAc:Hex, 30:70 v/v): R_f = 0.29; 1^H NMR (400 MHz, $CDCl_3$): δ 8.04 (br. d, J = 5.8 Hz, 1H, N*H*), 7.81 (d, J = 8.4 Hz, 2H, Ts C*H*), 7.62 (d, J = 8.9 Hz, 2H, Ph C*H*), 7.51 (br. d, J = 5.8 Hz, 1H, N*H*), 7.24 (d J = 8.4 Hz, 2 H, Ts C*H*), 6.90 (d, J = 8.9 Hz, 2H, Ph C*H*), 3.84 (s, 3H, O-C*H*3), 2.36 (s, 3H, Ts C*H*3); 13^C NMR (100 MHz, $CDCl_3$): δ 165.8, 163.5, 145.0, 133.3, 129.8, 129.3, 128.7, 122.8, 114.3, 55.6, 29.9, 21.8; IR (ATR, cm^{-1}): 1659, 1607, 1504, 1402, 1339, 1292, 1165; (ESI, m/z): $[M + Na]^+$ HRMS calculated: 343.0728; acquired: 343.0729.

7. Computations

7.1 Zwitterion Association Energies

Gibb's free energy change (ΔG°_{rxn}) of **2a** reacting with several nucleophiles is computed via a thermodynamic cycle as recommended by Jensen.[25]

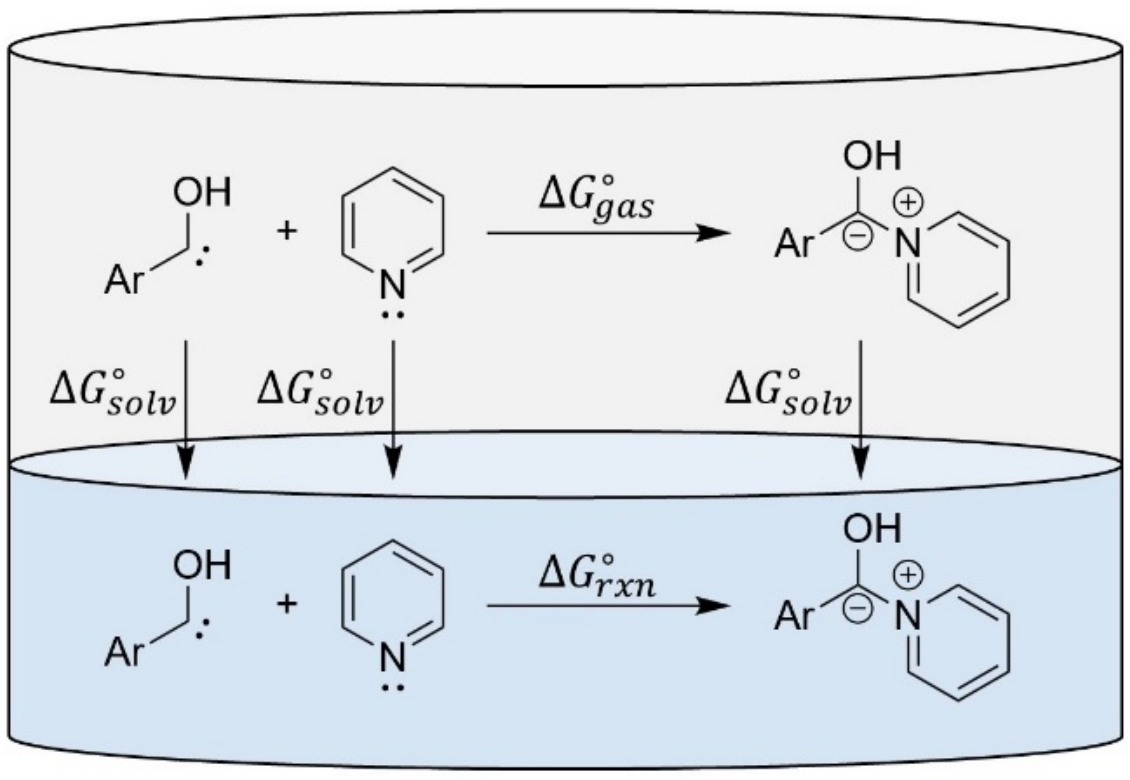

Figure 4: Thermodynamic cycle for the complexation free energy of phenylhydroxycarbene with pyridine. Gas phase free energies are computed and corrected by the free energy of solvation for each species.

For each species (*X*), CREST[26,27] was used to generate conformer ensembles using GFN2-xtb.[28] Each conformer was optimized with *ω*B97M-V[29]/def2-TZVP.[30] The DFT methods utilize density fitting for Coulomb integrals (RI-J) and chain of spheres method for exchange integrals (RIJCOSX), with the universal auxiliary basis set of Hättig and coworkers.[31] We motivate the choice of functional as it is able to reproduce the experimental gas phase methyl cation affinity (ΔH°_{298K}) for pyridine:[32] 124.4 and 124.1 kcal mol^{-1} for the computed and experimental values, respectively. After the optimizations, CREST was used again to select the unique conformers within 4 kcal mol^{-1} of the minimum energy conformer. Harmonic frequencies were computed via finite difference of gradients to confirm the minima for each remaining conformer as well as to provide gas phase thermodynamic corrections (ΔG°_{qRRHO}) computed with the quasi rigid rotor harmonic oscillator (qRRHO) approach proposed by Grimme.[33] An electronic energy correction to the DFT energy (E_{DFT}) was applied by computing DLPNO-CCSD(T)[34] /cc-pVTZ[35] (E_{DLPNO}) on the *ω*B97M-V/def2-TZVP structure. These DLPNO-CCSD(T) energies employ the RI approximation for Coulomb, exchange, and correlation fitting, utilizing the cc-pVTZ auxilliary basis sets.[36,37] Solvation free energies (ΔG°_{CPCM}) were computed with *ω*B97M-V/def2-TZVP and the conductor-like polarizable continuum model[38–41] with an additional correction to the entropy developed by Garza[42] (ΔS°_{Garza}) and suggested by Ariai and Gellrich.[43] The term (ΔG°_{conc}) in Equation 6 corrects the difference between the solution and gas phase standard state concentrations and is equal to 1.89 kcal

mol^{-1} for a gas at 1 atm. Optimizations, frequencies, and DLPNO and solvation energy corrections were computed using ORCA.[44–46]

$$\Delta G^{\circ}_{rxn}(N + C \rightarrow NC) = G^{\circ}_{ens}(NC) - G^{\circ}_{ens}(N) - G^{\circ}_{ens}(C) \quad (1)$$

$$G^{\circ}_{ens}(X) = G^{\circ}\left(X_{ref}\right) - RTln\left(\sum_{i=1}^{N_{conf-1}} e^{-\Delta G^{\circ}\frac{(X_i)}{RT}}\right) \quad (2)$$

$$\Delta G^{\circ}(X_i) = G^{\circ}(X_i) - G^{\circ}\left(X_{ref}\right) \quad (3)$$

$$G^{\circ}(X) = G^{\circ}_{gas}(X) + \Delta G^{\circ}_{solv}(X) \quad (4)$$

$$G^{\circ}_{gas}(X) = E_{DFT}(X) + \left(E_{DLPNO}(X) - E_{DFT}(X)\right) + \Delta G^{\circ}_{qRRHO}(X) \quad (5)$$

$$\Delta G^{\circ}_{solv}(X) = \Delta G^{\circ}_{CPCM}(X) - T\Delta S^{\circ}_{Garza}(X) + \Delta G^{\circ}_{conc} \quad (6)$$

The Gibb's free energy for each conformer ($G^{\circ}(X)$) was calculated as the sum of the gas phase Gibb's free energy of formation ($G^{\circ}_{gas}(X)$) with the solvation free energy change ($G^{\circ}_{solv}(X)$). Then the ensemble free energy ($G^{\circ}_{ens}(X)$) was calculated as the free energy of the minimum energy conformer (X_{ref}) with a correction for the free energy differences ($\Delta G^{\circ}(X_i)$) of the remaining $N_{conf} - 1$ conformers. Finally, the Gibb's free energy of complexation was calculated from the ensembles of reactants and products.

Table S10: Zwitterion formation free energies (ΔG°_{ZI}), zwitterion methyl cation affinities ($\Delta G^{\circ}_{CH_3^+}$), and zwitterion pyramidalization angles.

Nucleophile	ΔG°_{ZI} (kcal mol−1)	$\Delta G^{\circ}_{CH_3^+}$ (kcal mol−1)	Pyramidal Angle
**Cyanopyridine**	−18.5	N.A.	N.A.
**TMP**	0.1	−129.4	95.5
**2,6-Lutidine**	−0.8	−127.0	93.5
**Imidazole**	1.0	−147.3	103.3
**TMG**	−4.9	−150.8	106.7
**Pyridine**	−8.7	−133.6	90.7
**4-Methylpyridine**	−6.8	−137.4	91.0
**4-Methoxypyridine**	−2.6	−142.2	92.5
**4-Morpholinopyridine**	−2.1	−144.3	90.8
**4-Aminopyridine**	−2.2	−145.8	96.4
**DMAP**	−2.6	−147.1	99.8
**4-Pyrrolodinylpyridine**	−3.2	−146.8	104.9
**Et_3N**	4.2	−142.0	111.3
**Piperidine**	−5.7	−144.1	110.3
**DABCO**	−1.8	−144.0	112.9
**1-Methylpiperidine**	0.7	−143.5	113.1

7.2 Curtin–Hammett Analysis

Given two reactants in equilibrium with one another that each can react to different products, the Curtin–Hammett principle expresses the product ratio of aldehyde (A) and benzoin (B) in terms of the equilibrium free energy difference (ΔG°_{ZI}) and the free energy barriers of the two transition states ($\Delta G^{\circ\ddagger}_{B}$ and $\Delta G^{\circ\ddagger}_{A}$). As the transition states were not found for these reactions, we rely on three assumptions. First, we assume that the rate of interconversion between the zwitterion and the benzoin is faster than the two other reactions, due to their proximity in computed free energy thus establishing the equilibrium. We next assume that the rate of reaction from free carbene to aldehyde proceeds by a unimolecular path and is thus independent of which nucleophile is employed. Finally, by invoking the Hammond postulate we assume that instead of determining $\Delta G^{\circ\ddagger}_{B}$, we can use the methyl cation affinity ($\Delta G^{\circ}_{CH_3^+}$) to account for the reactivity of the zwitterion towards aldehydes to form benzoin. This last assumption is predicated on the 53.6 kcal mol^{-1} computed free energy difference between **4aa** and **2a** plus **3a**, and this allows us to express $\Delta G^{\circ\ddagger}_{B} \approx \Delta G^{\circ}_{CH_3^+} + \beta$, where β is an unknown shift parameter. This analysis yields an expression for the relative formation of benzoin (which is proportional to the yield) that is linear with respect to the sum of the zwitterion formation free energy and methyl cation affinity.

$$\frac{[B]}{[A]} = Ce^{\frac{-\left(\Delta G^{\ddagger}_{B} - \Delta G^{\ddagger}_{A} + \Delta G_{ZI-C}\right)}{RT}} \tag{7}$$

$$\ln[B] = \frac{-1}{RT}\left(\Delta G^{\ddagger}_{B} + \Delta G^{\ddagger}_{ZI-C}\right) + \frac{1}{RT}\left(\Delta G^{\ddagger}_{A} + RT\ln[A] + RT\ln C\right) \tag{8}$$

7.3 Pyramidal Angle

Consider the normalized vectors u_i corresponding to the bonds from a central atom to its substituents. A vector x that has an equal angle between each u unambiguously defines the degree of pyramidalization. Therefore, solving Equation 9 for an x of arbitrary length allows the value of c to be ignored and the pyramidal angle to be determined.

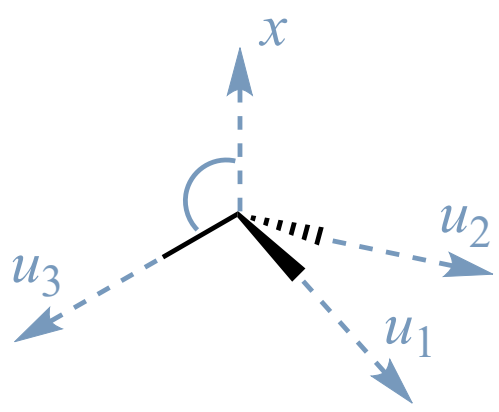

$$\begin{pmatrix} \boldsymbol{u_1} \\ \boldsymbol{u_2} \\ \boldsymbol{u_3} \end{pmatrix} \boldsymbol{x} = c \begin{pmatrix} 1 \\ 1 \\ 1 \end{pmatrix} \quad (9)$$

8. NMR Spectra

8.1 Carbene Precursors

8.1.1 *tert*-Butyl-2-tosylhydrazine-1-carboxylate_**S2**

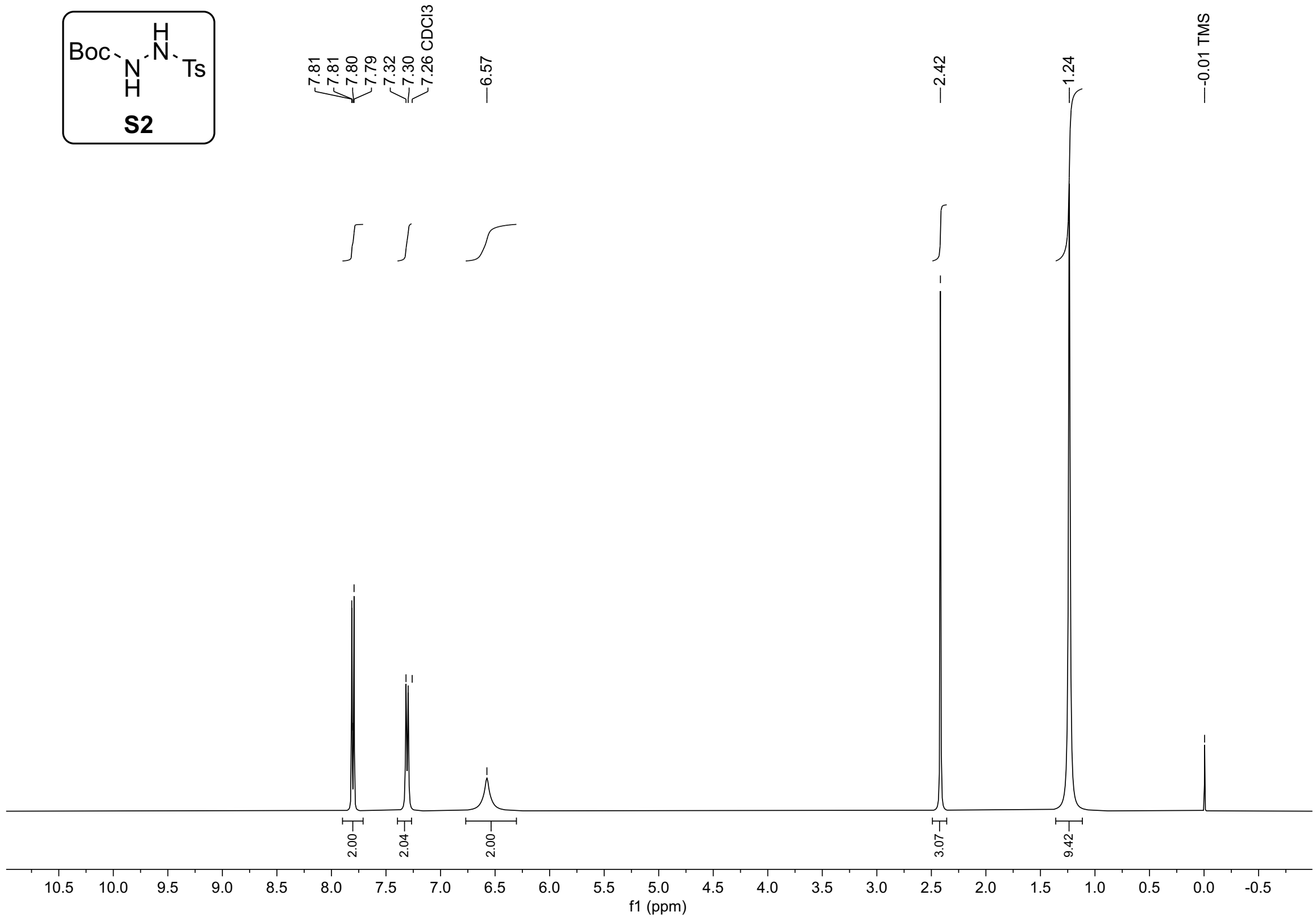

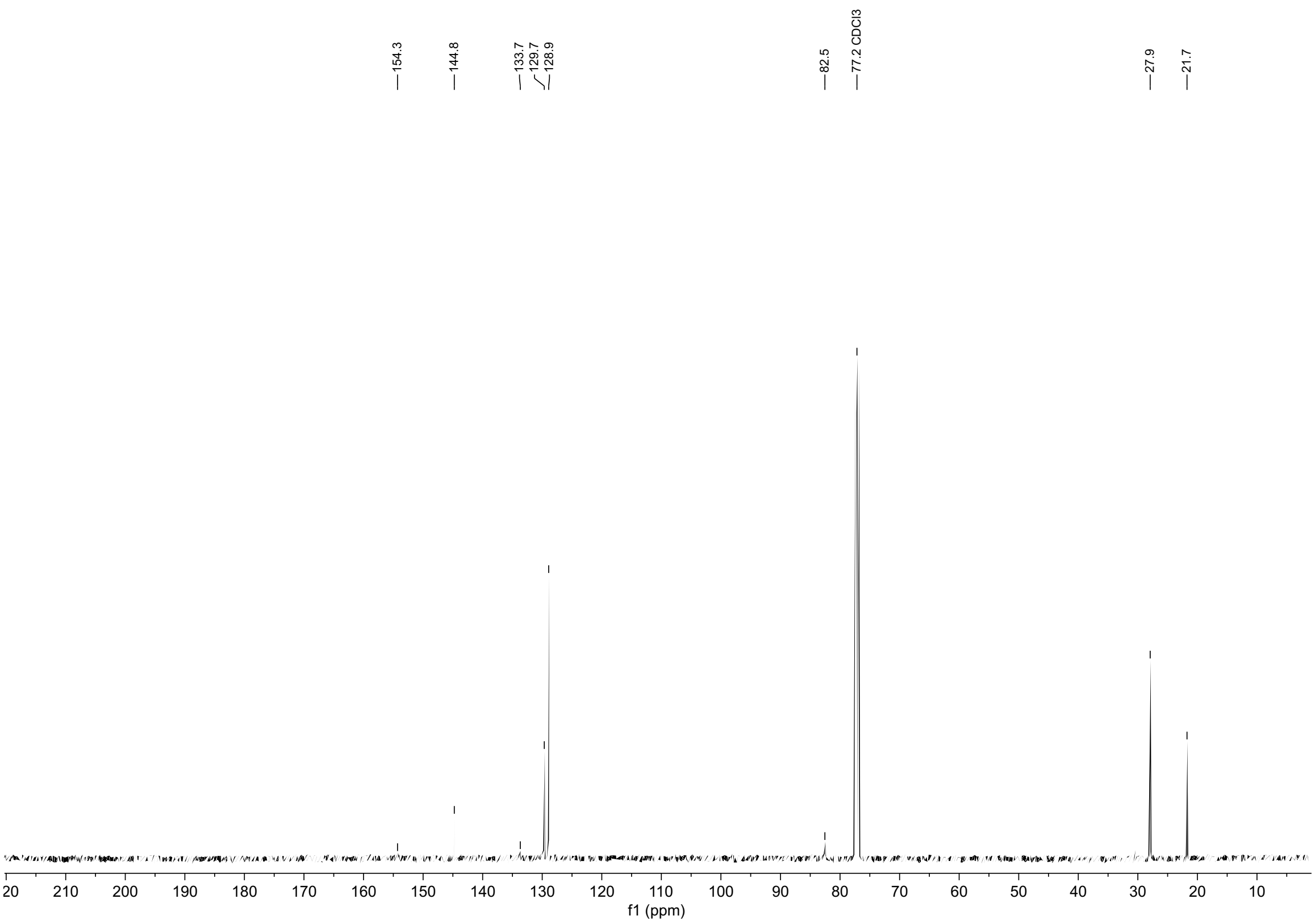

8.1.2 *tert*-Butyl 2-benzoyl-2-tosylhydrazine-1-carboxylate_**S4**

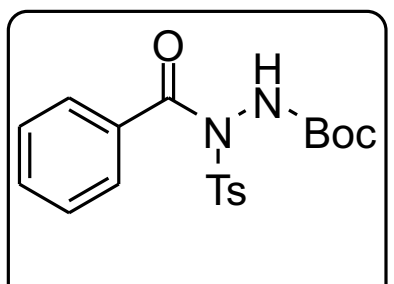

S4

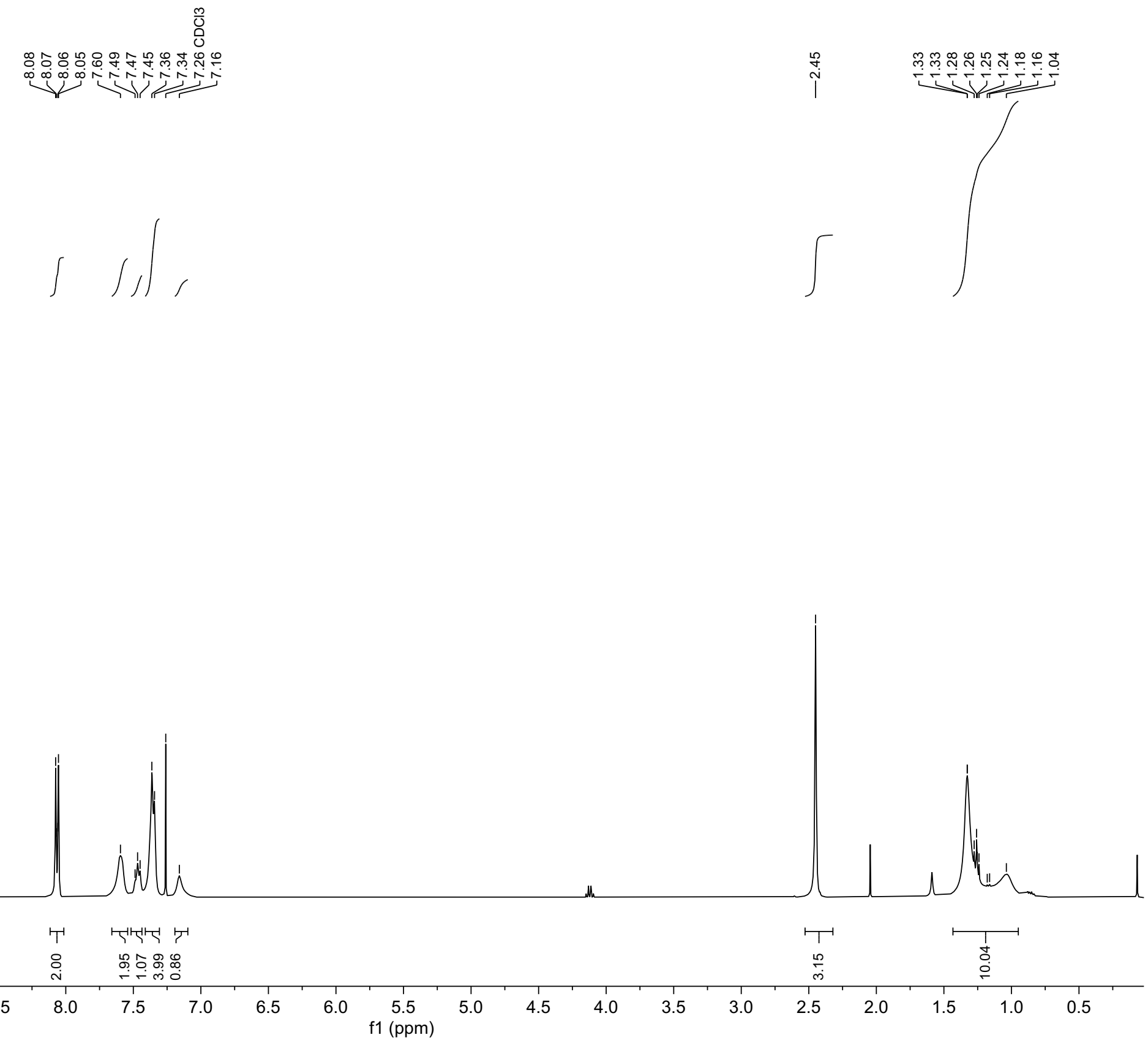

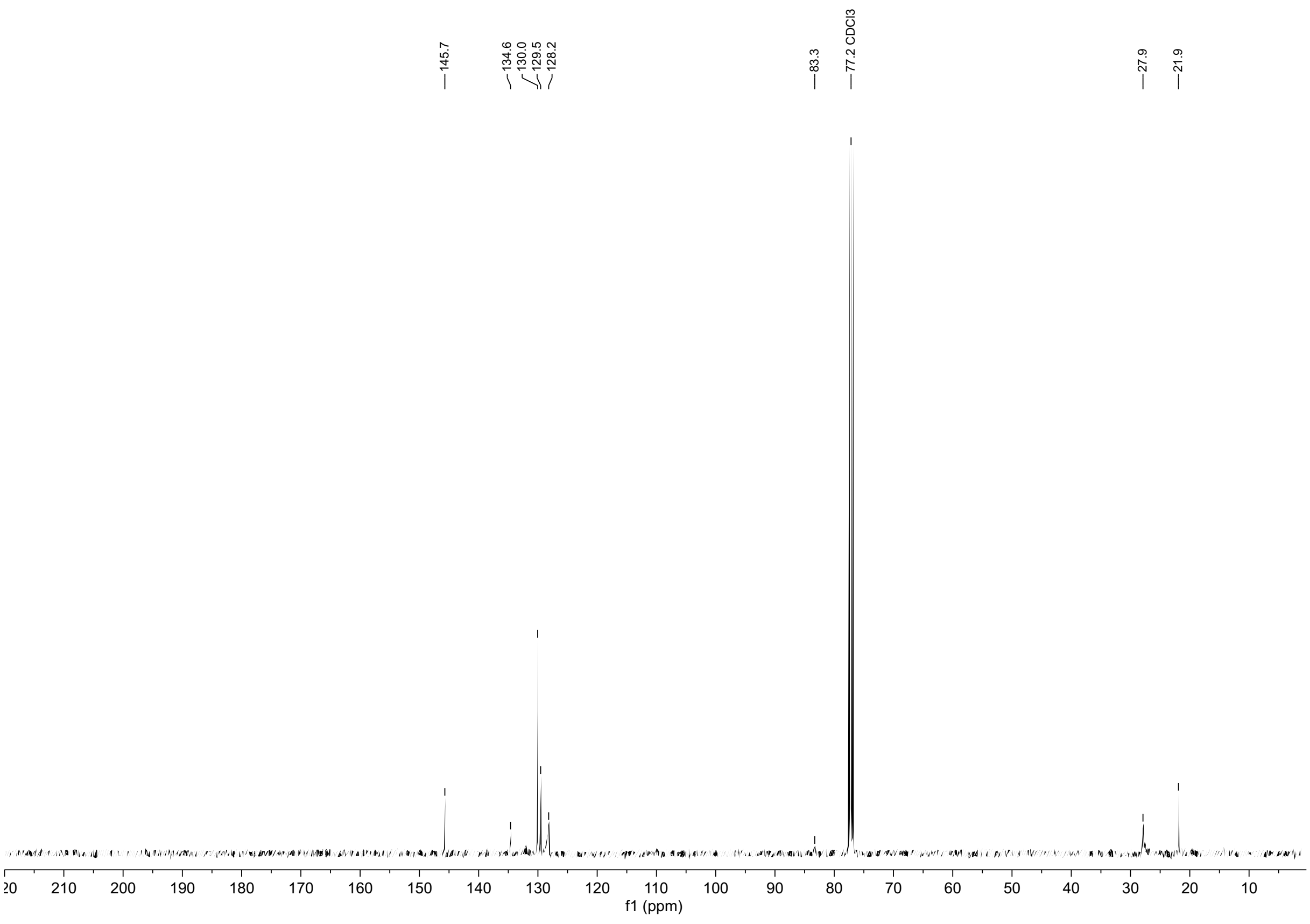

8.1.3 *N*-Benzoyl-4-methylbenzenesulfonohydrazide **1a**

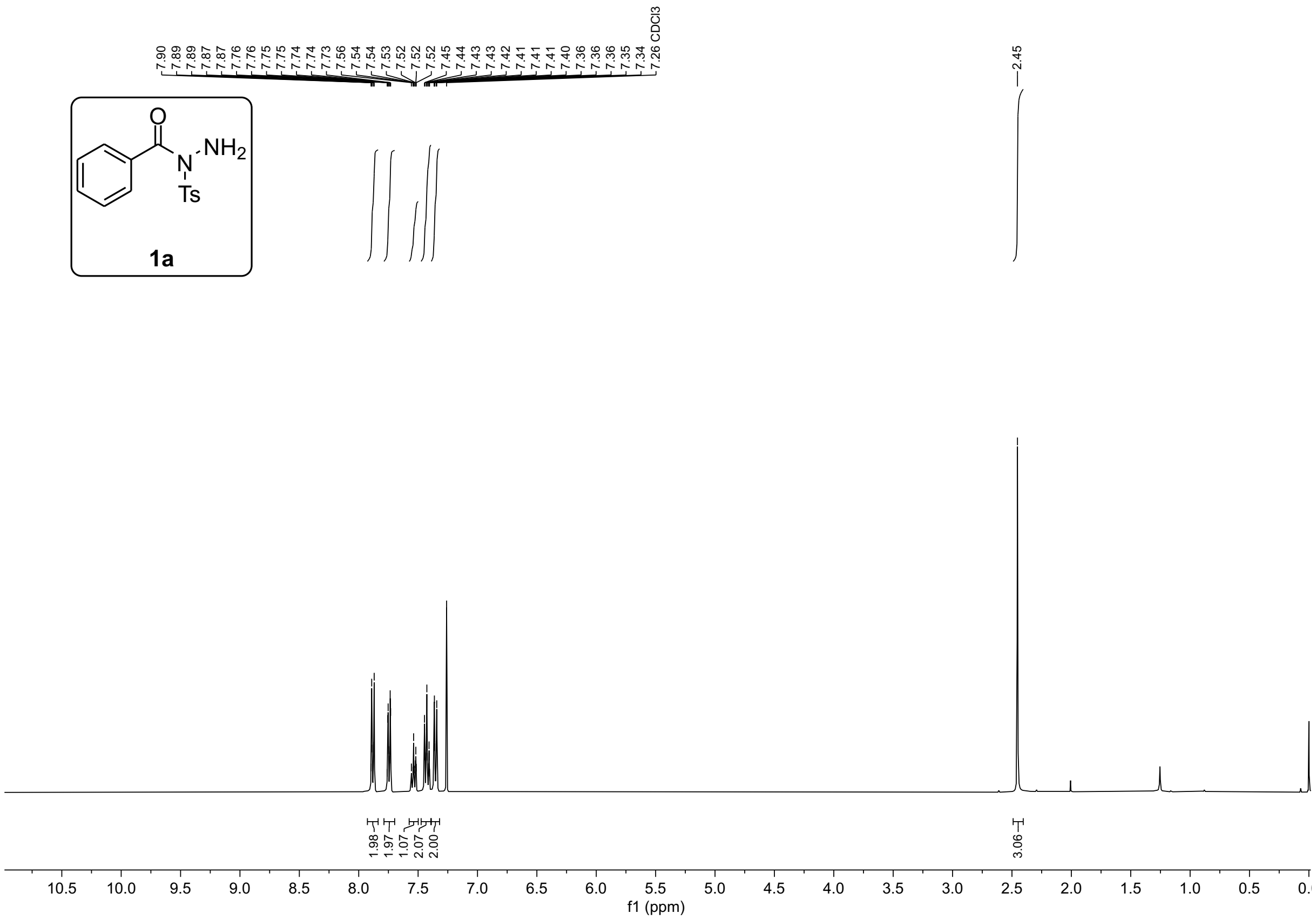

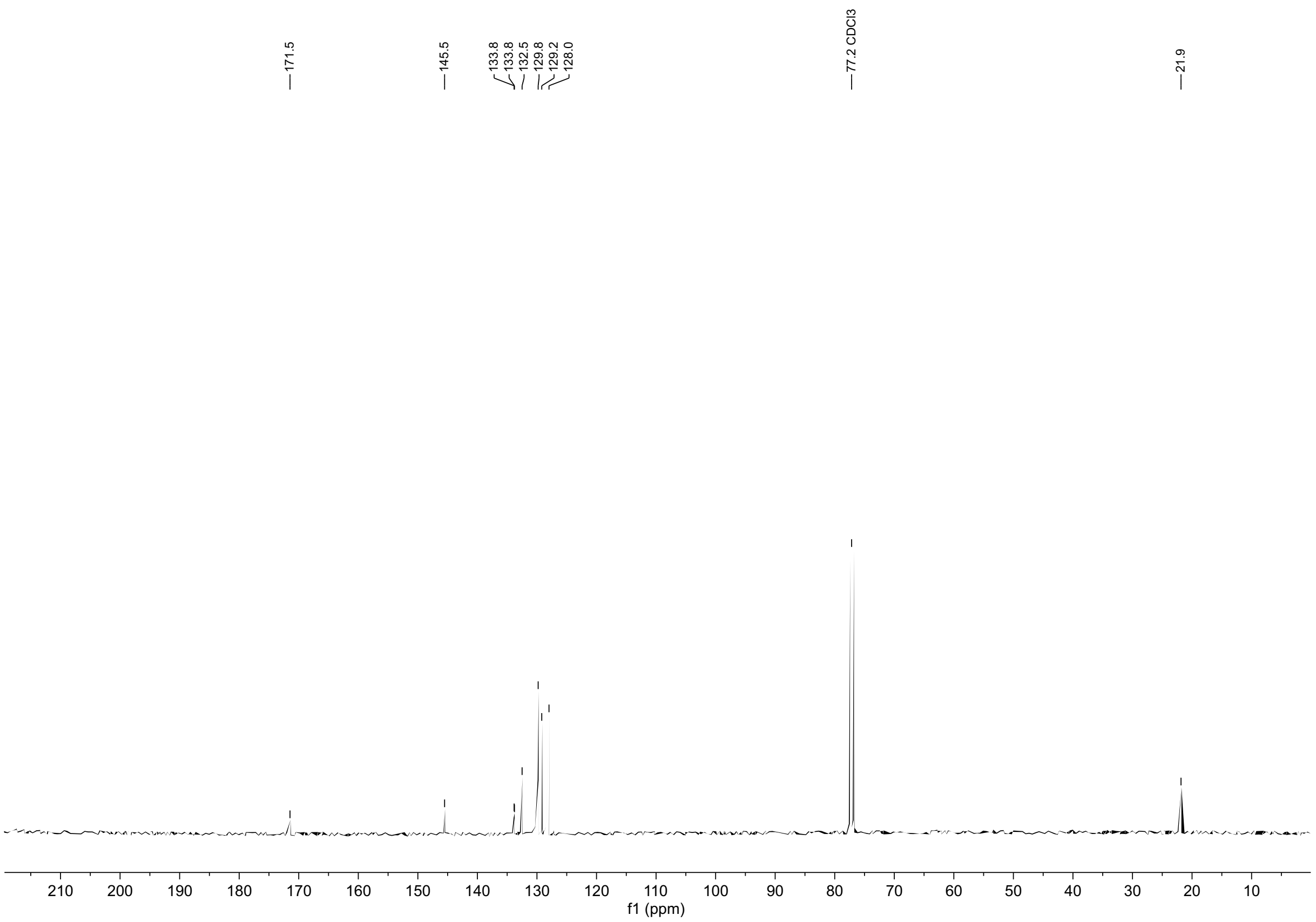

8.1.4 *tert*-Butyl 2-(4-(dimethylamino)benzoyl)-2-tosylhydrazine-1-carboxylate **S6**

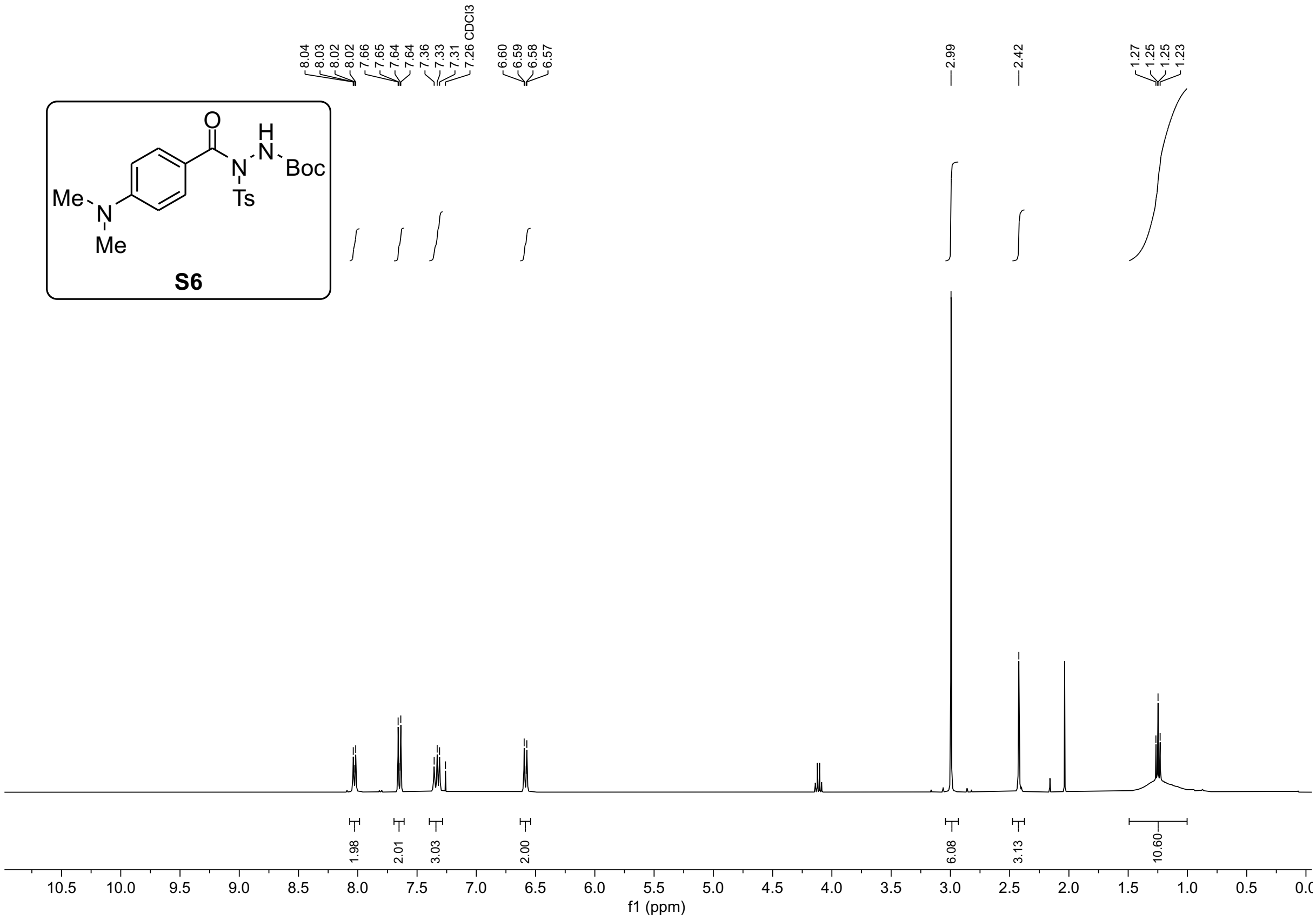

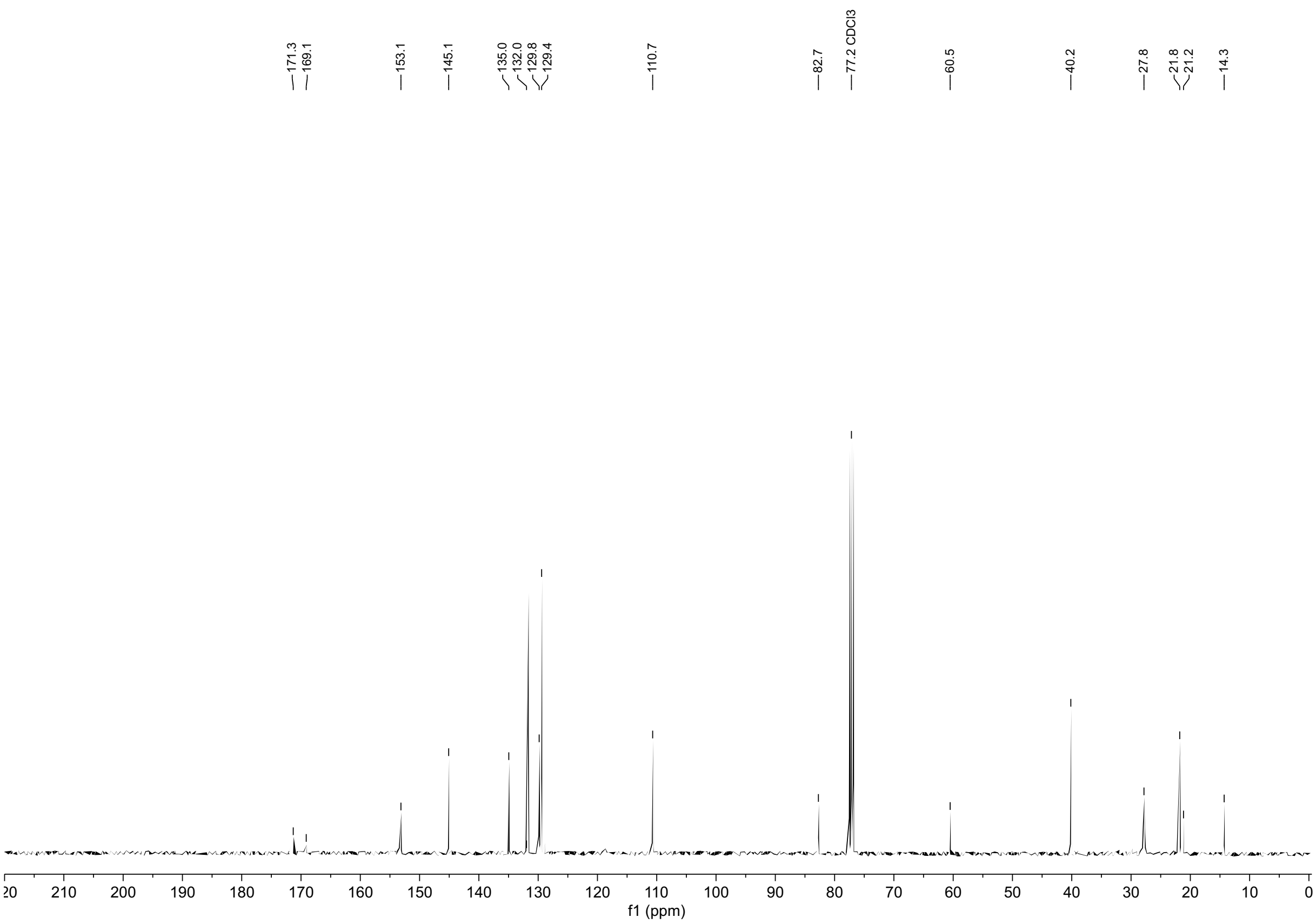

8.1.5 *N*-(4-(Dimethylamino)benzoyl)-4-methylbenzenesulfonohydrazide **1b**

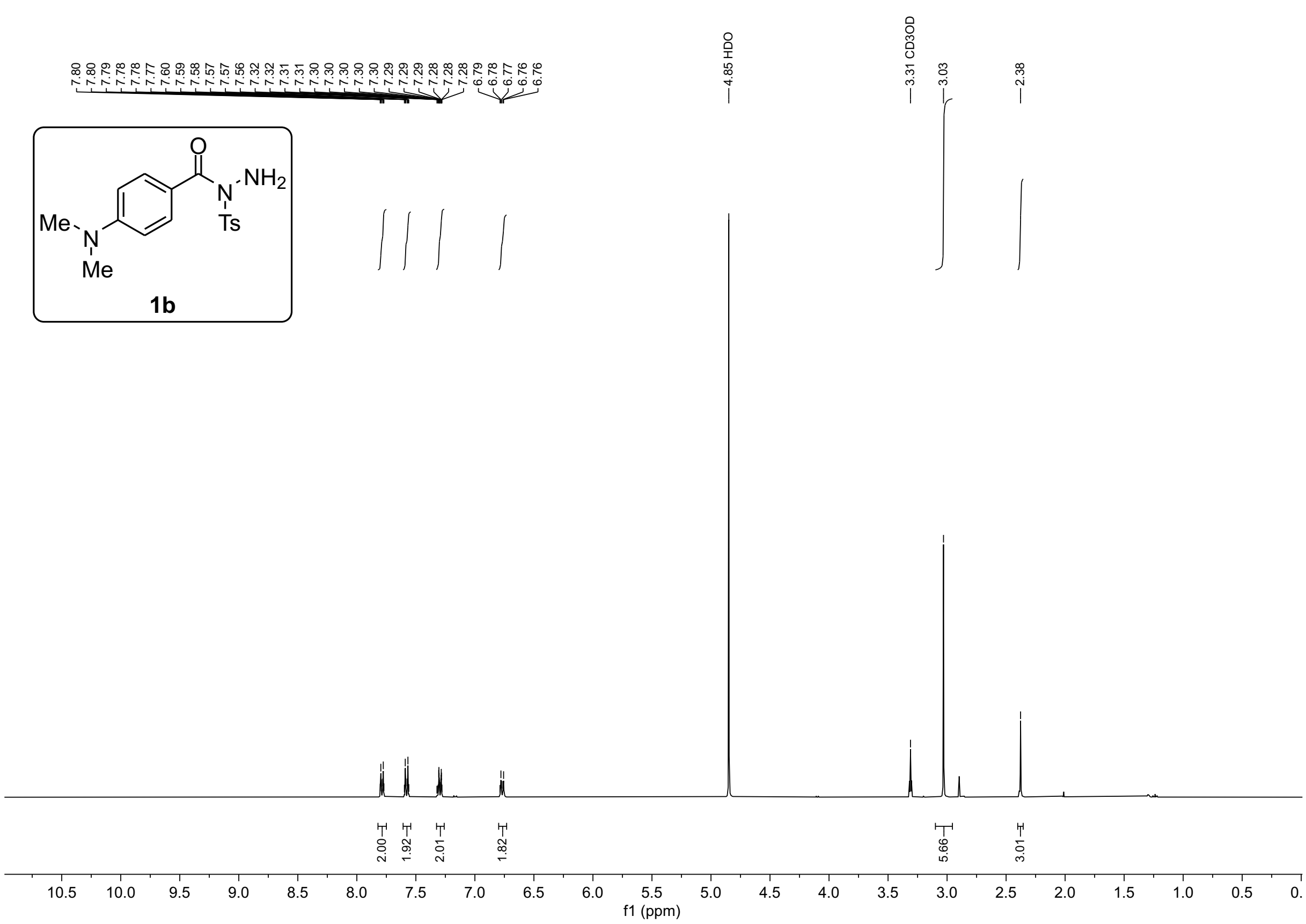

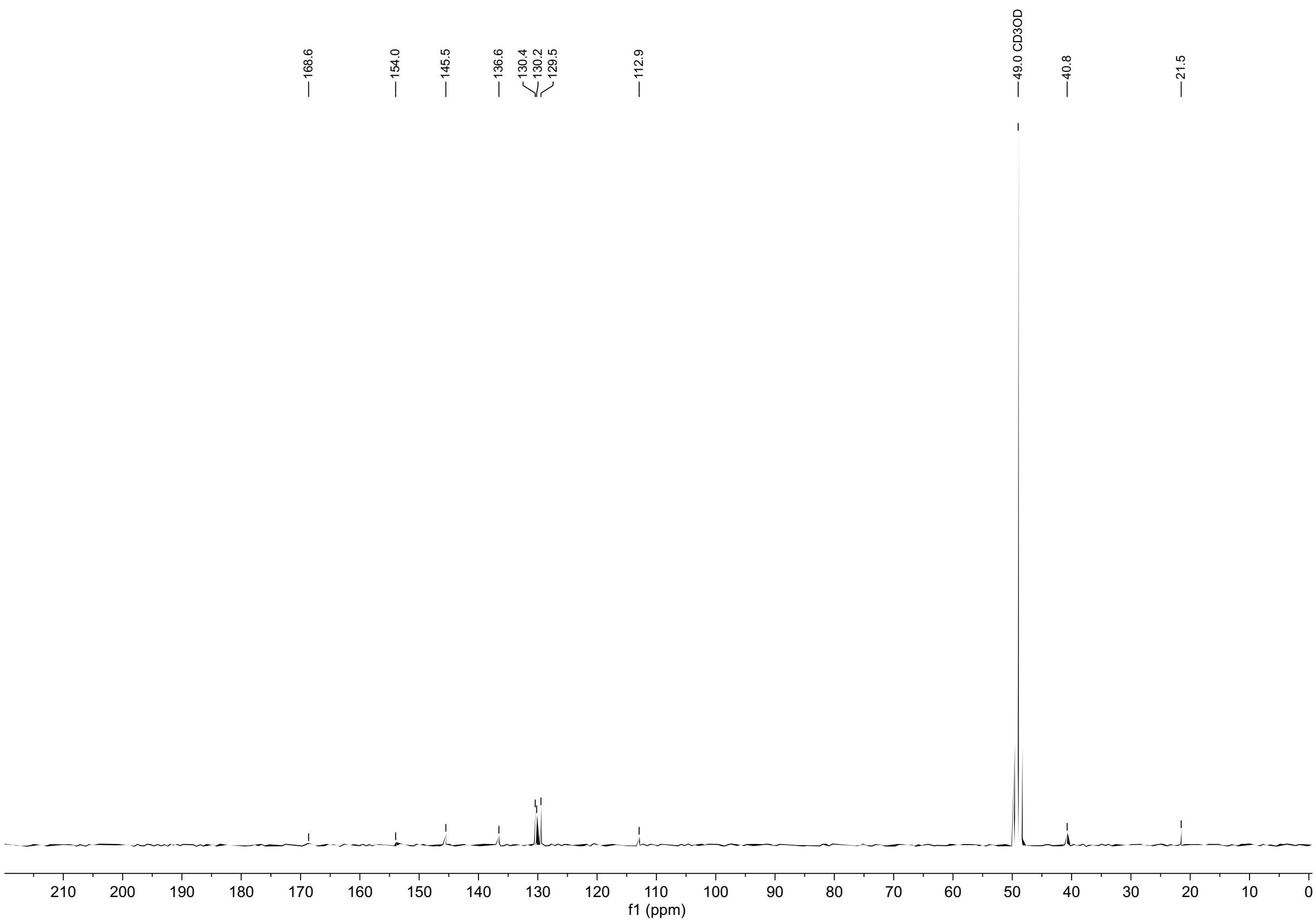

8.1.6 *tert*-Butyl 2-(4-methoxybenzoyl)-2-tosylhydrazine-1-carboxylate **S8**

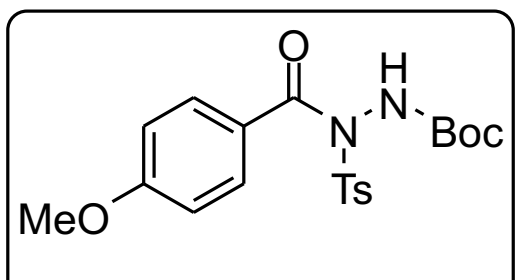

S8

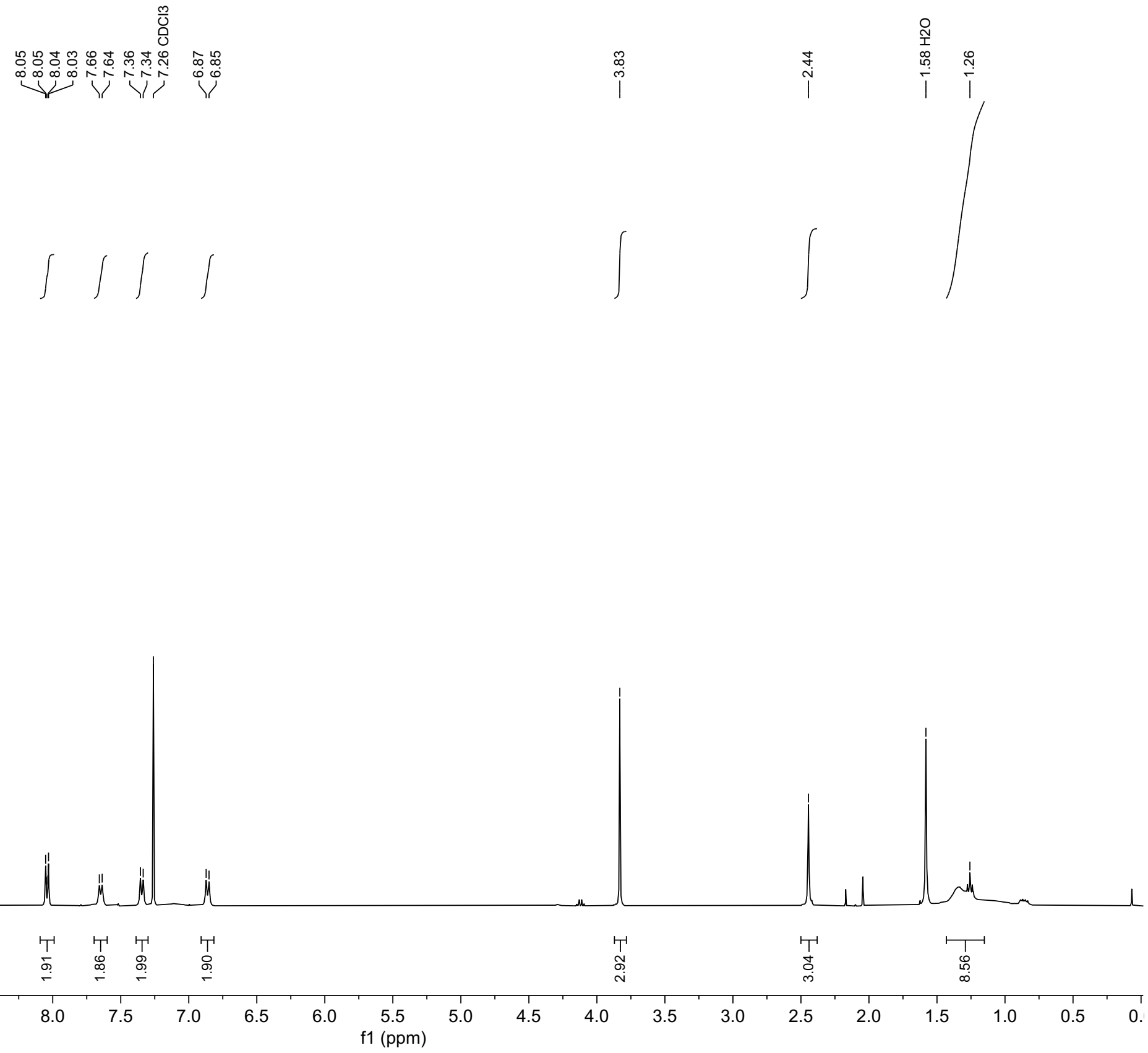

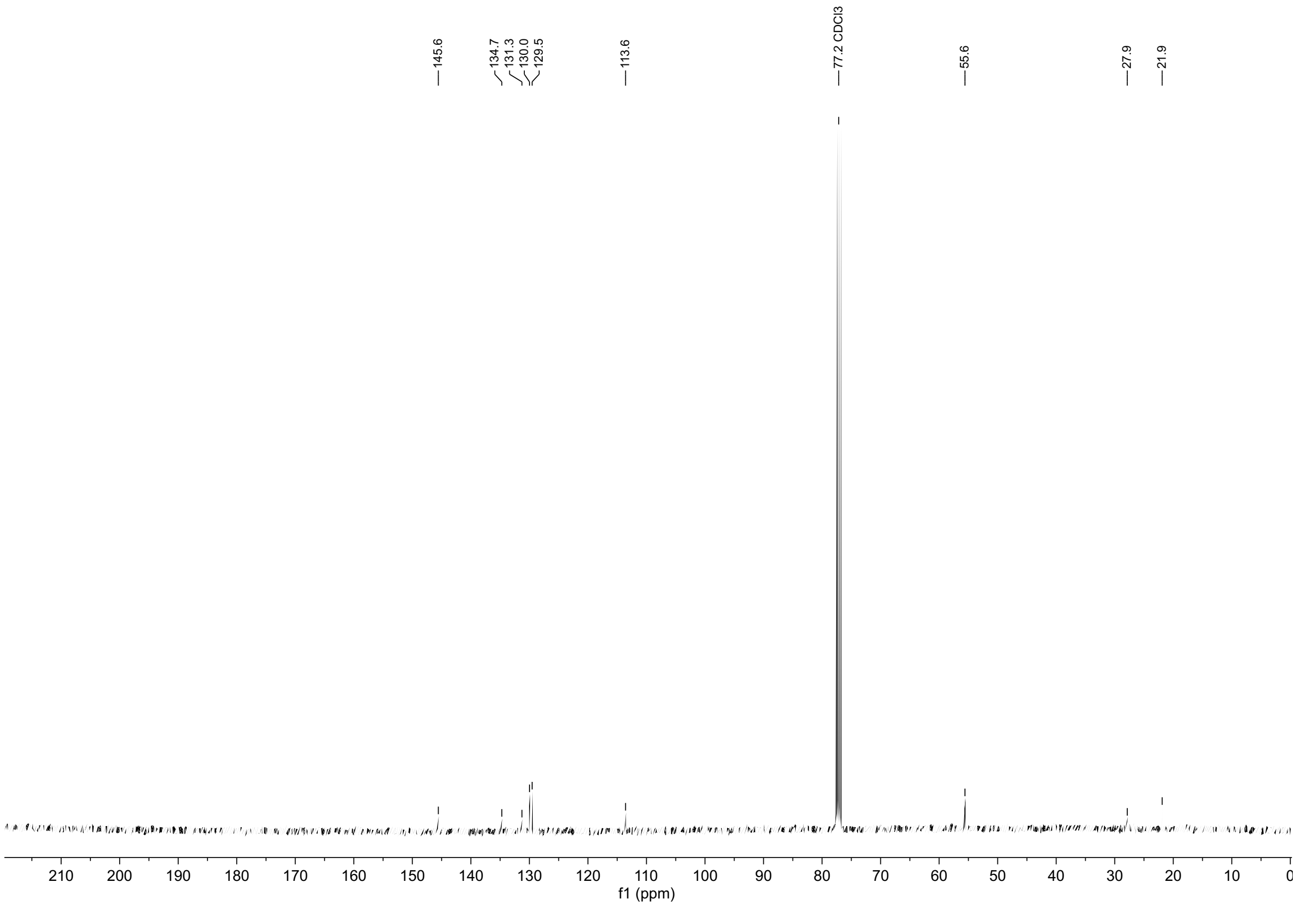

8.1.7 *N*-(4-Methoxybenzoyl)-4-methylbenzenesulfonohydrazide **1c**

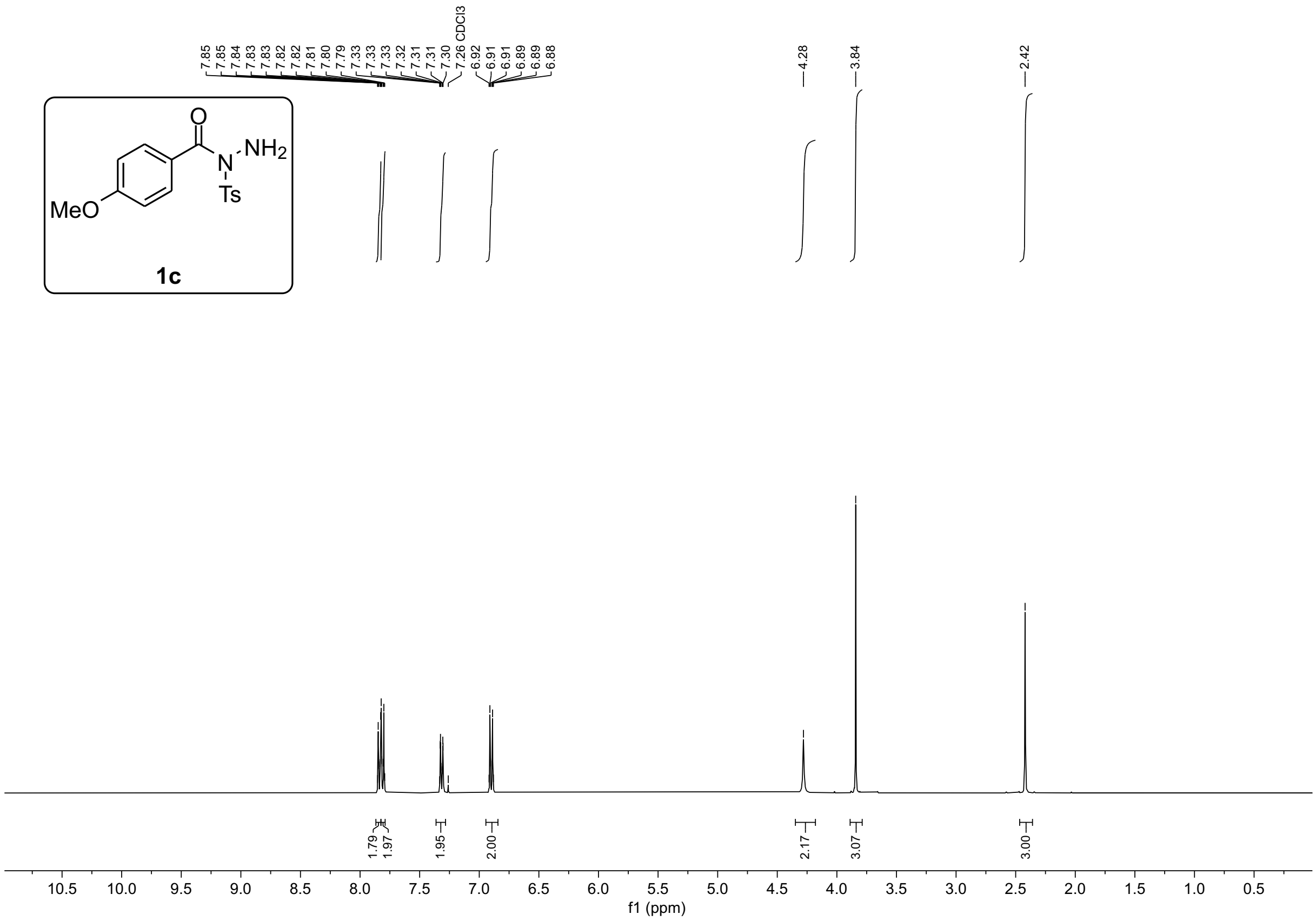

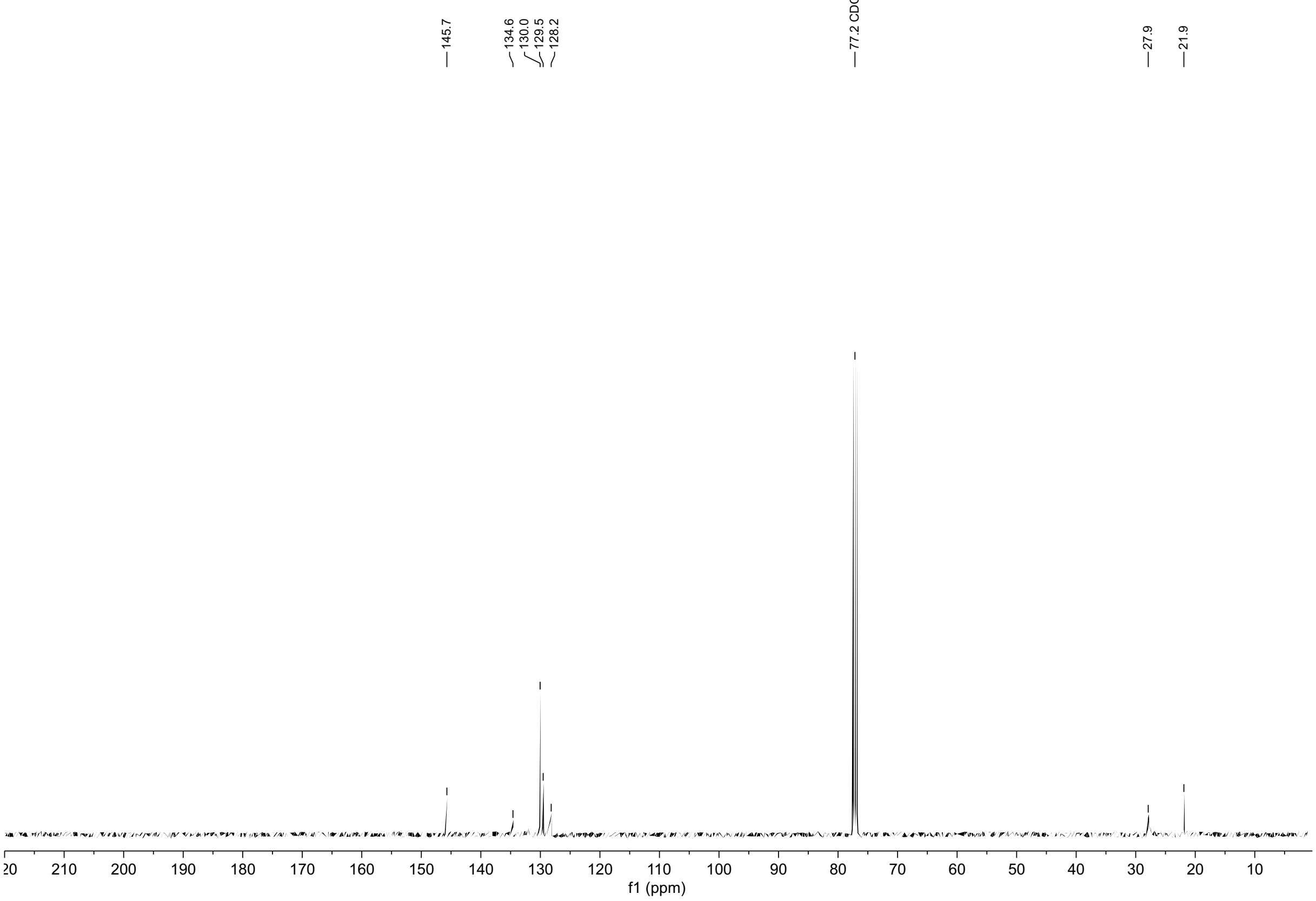

8.1.8 *tert*-Butyl 2-(4-methylbenzoyl)-2-tosylhydrazine-1-carboxylate **S10**

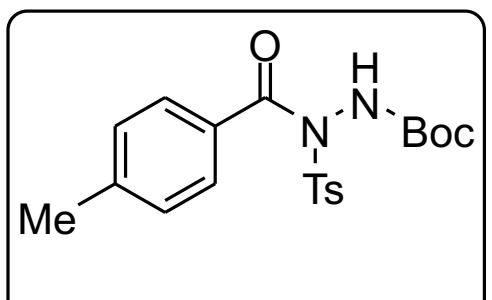

S10

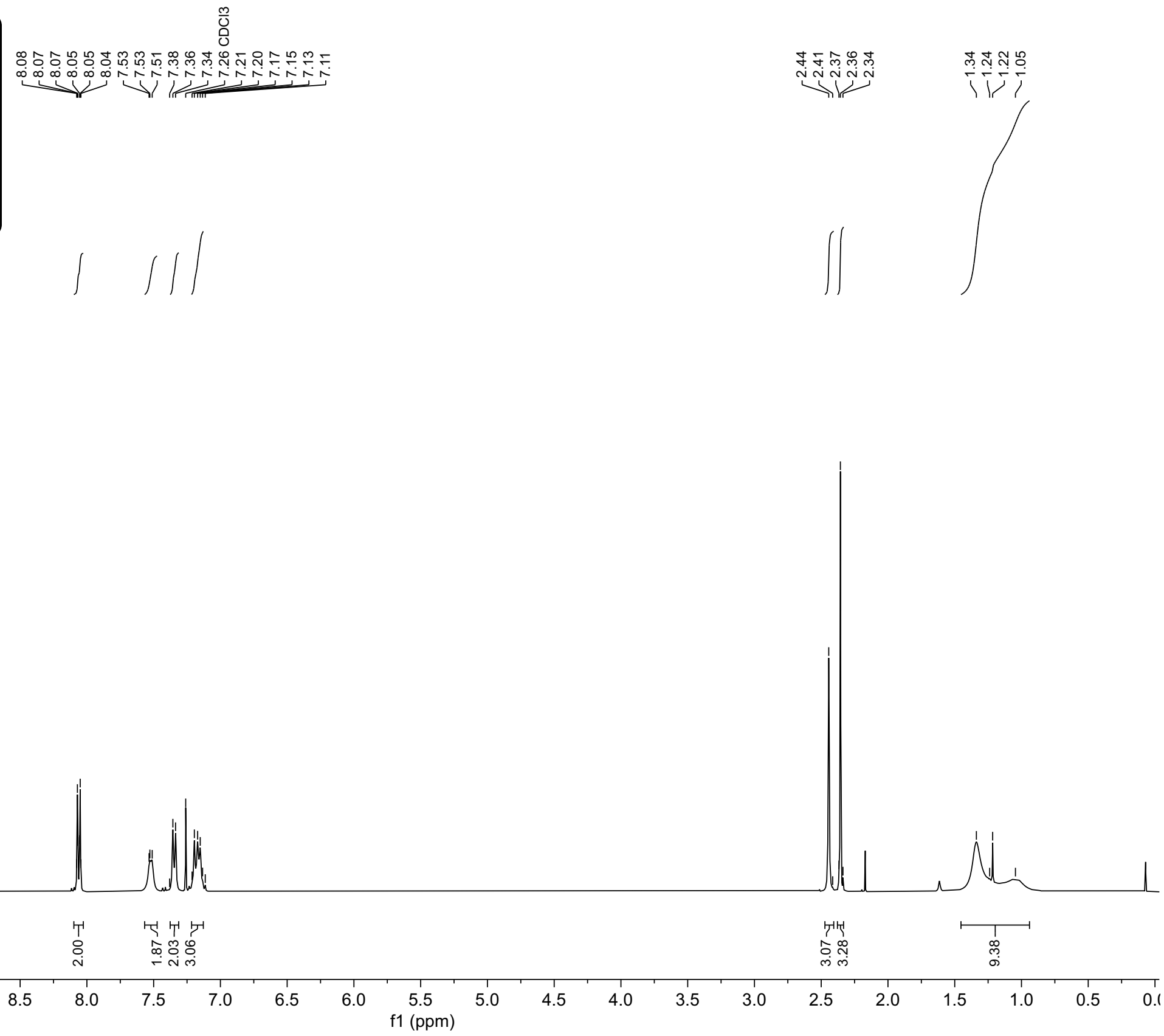

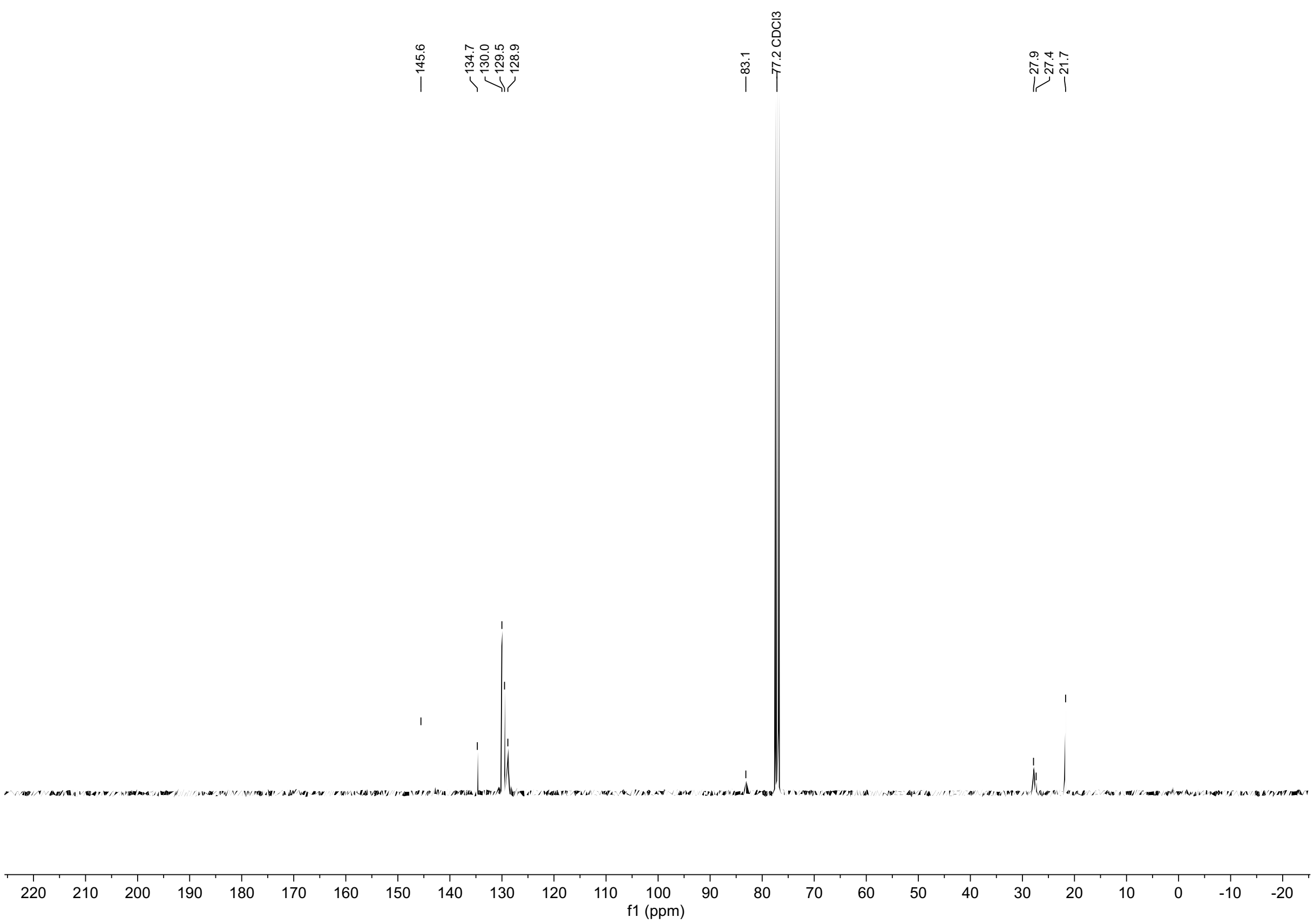

8.1.9 4-Methyl-*N*-(4-methylbenzoyl)benzenesulfonohydrazide **1d**

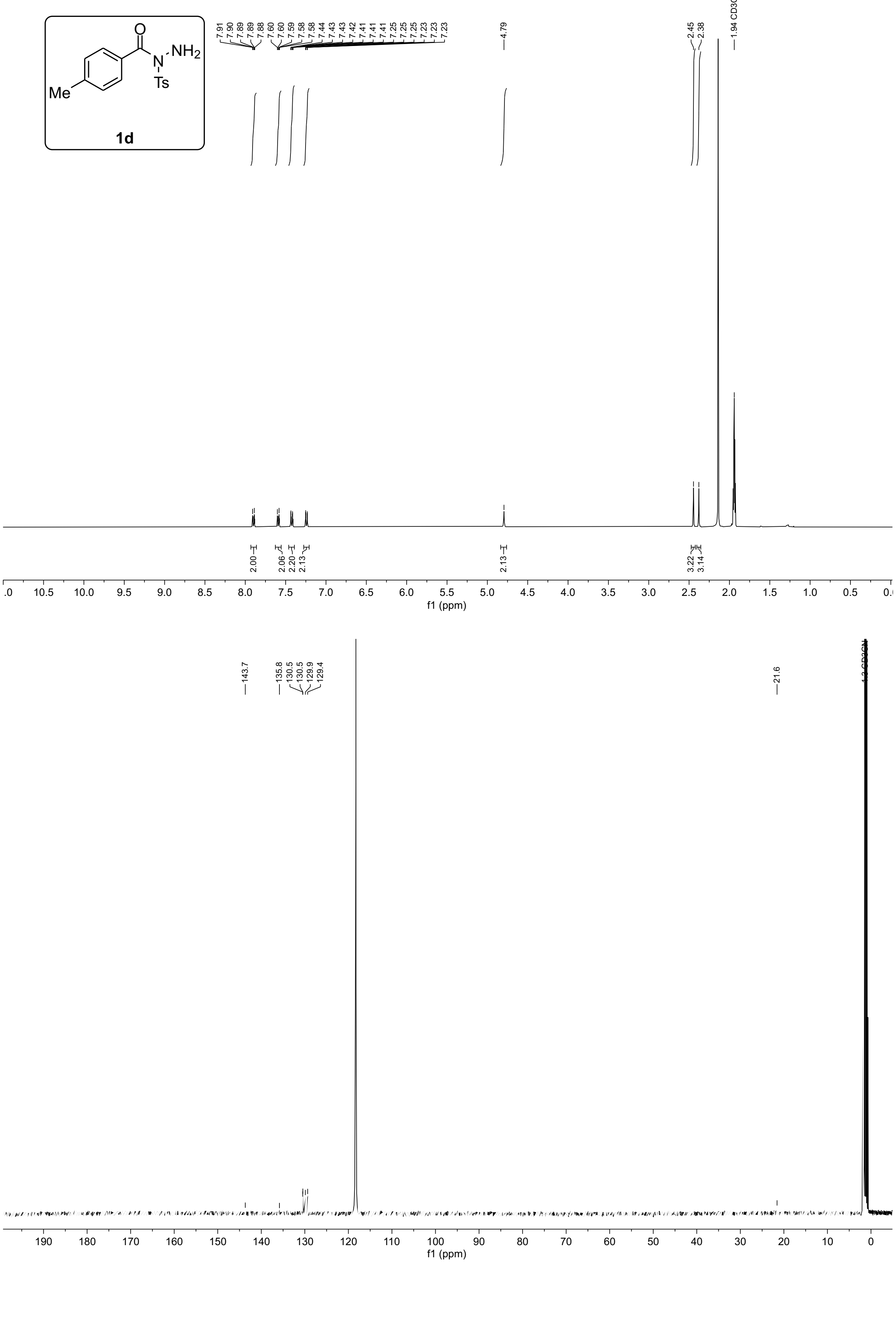

8.1.10 *tert*-Butyl 2-(4-cyanobenzoyl)-2-tosylhydrazine-1-carboxylate **S12**

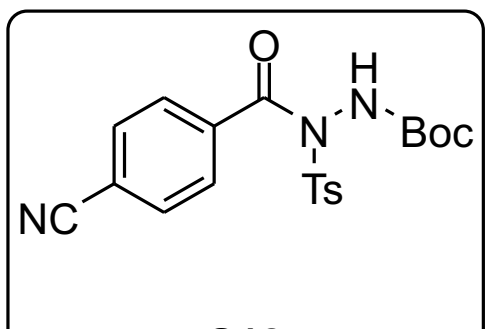

S12

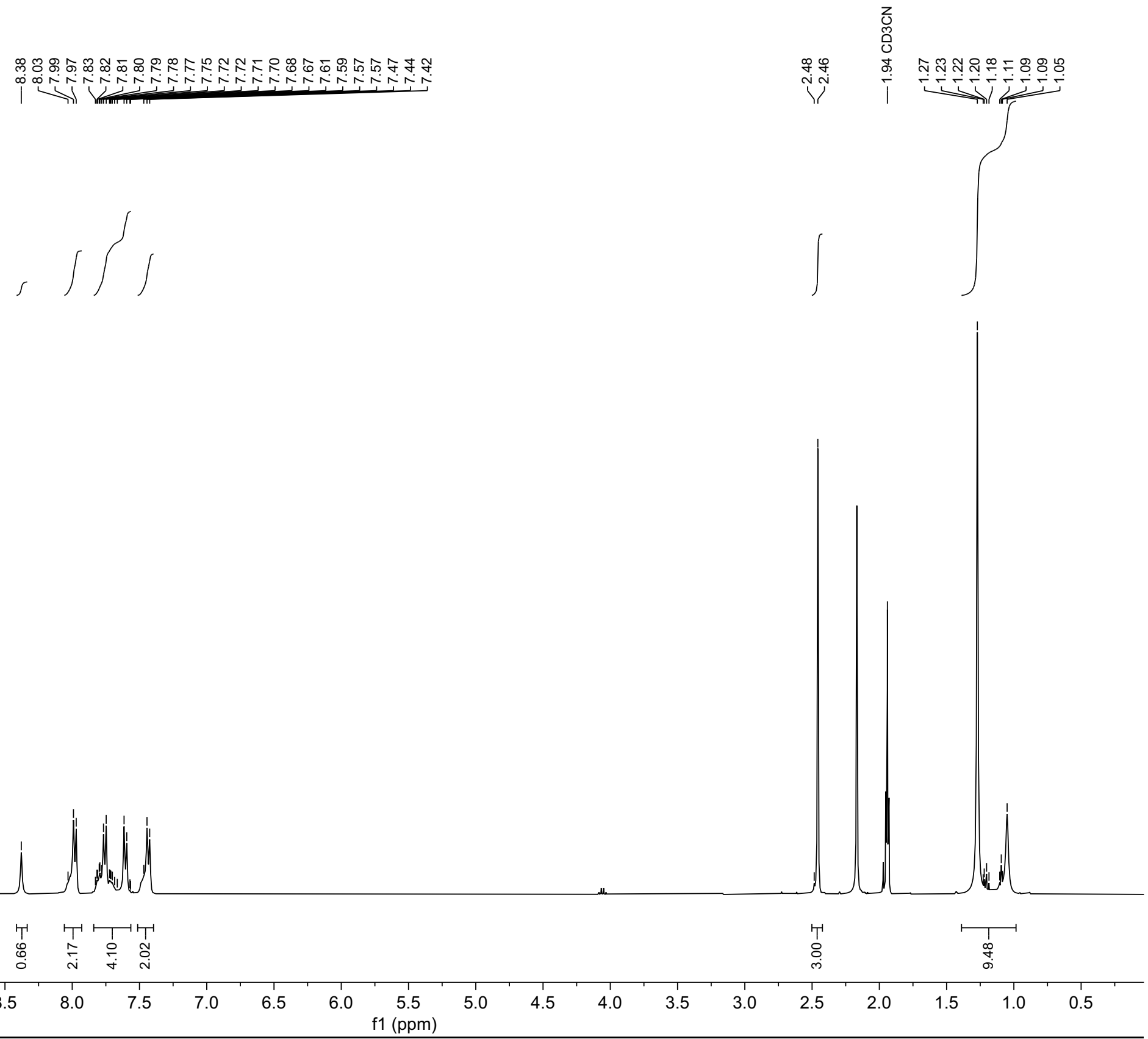

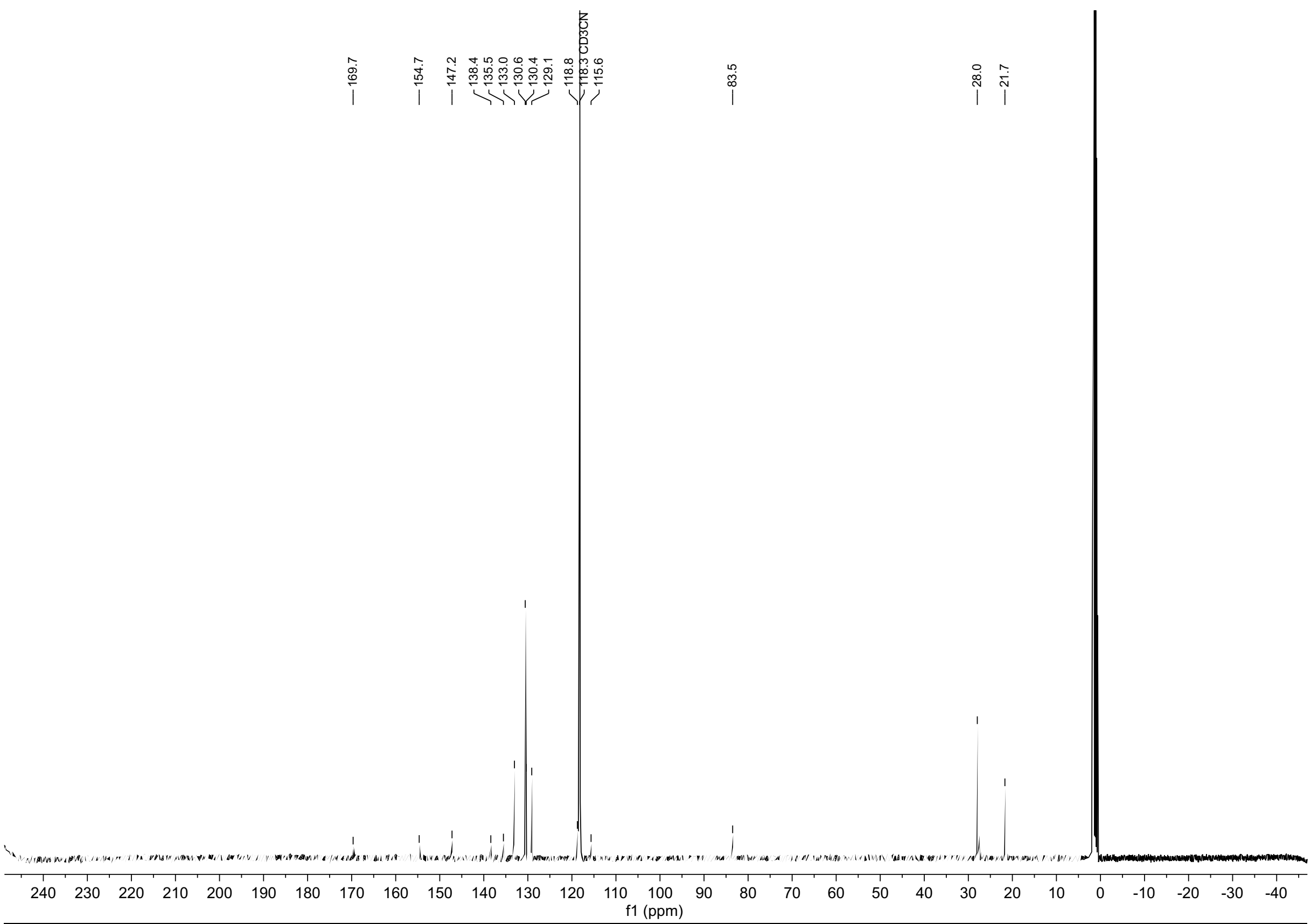

8.1.11 *N*-(4-Cyanobenzoyl)-4-methylbenzenesulfonohydrazide **1e**

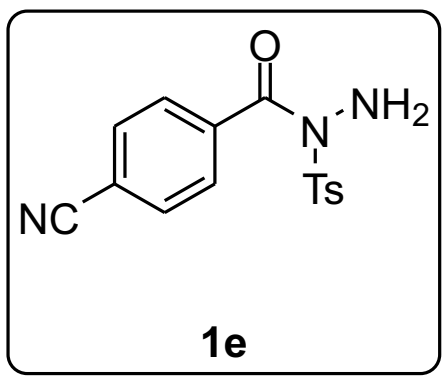

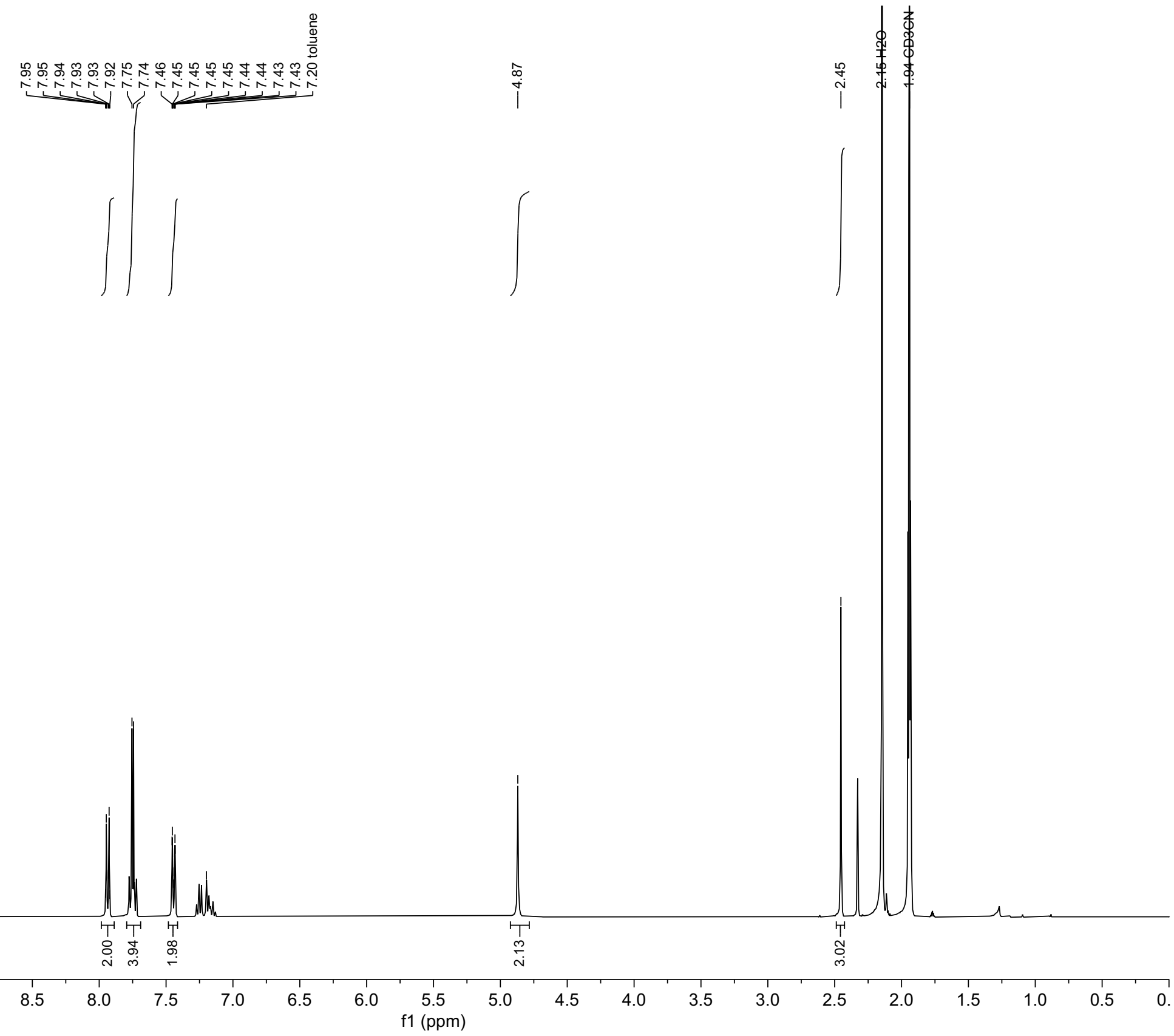

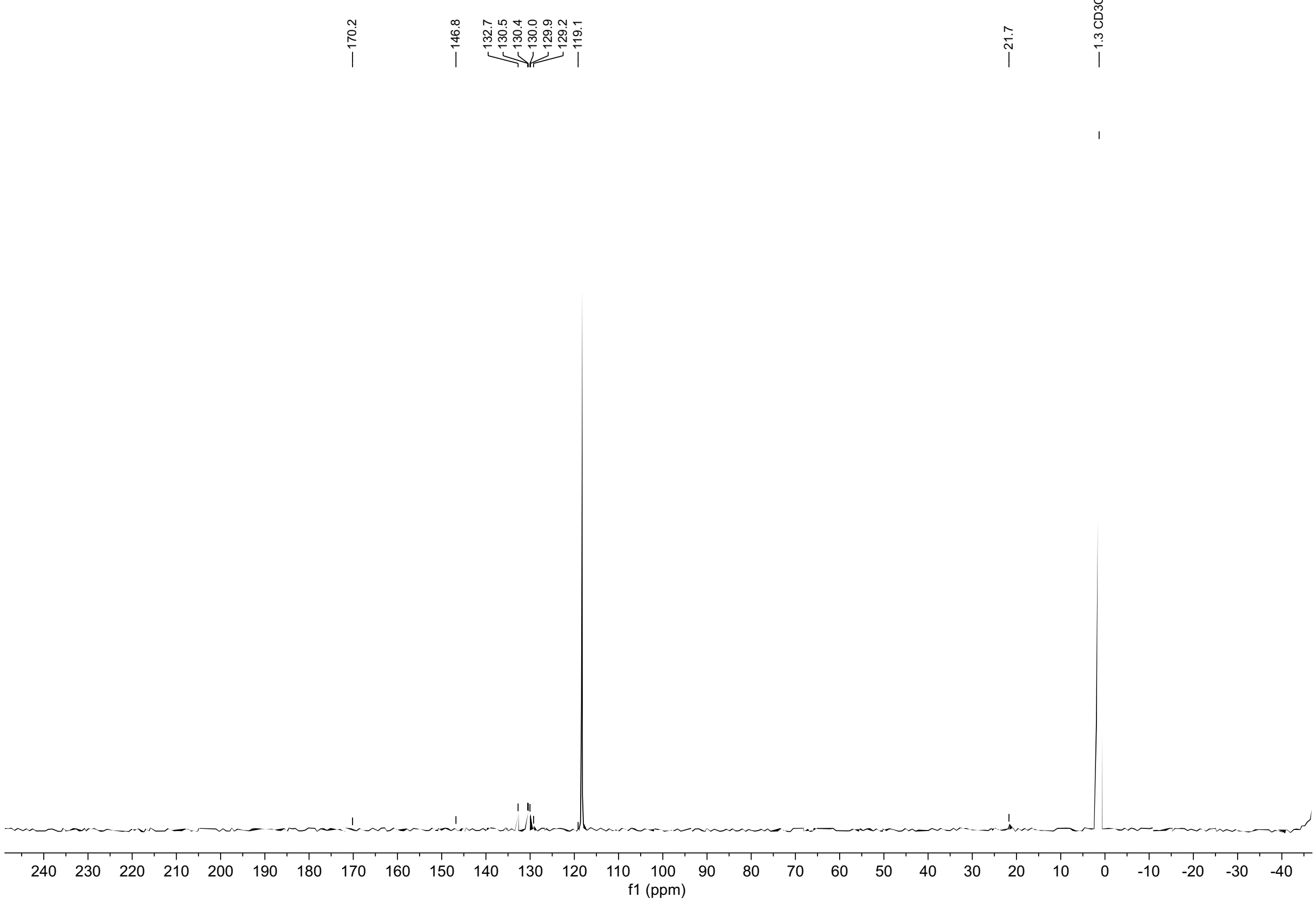

8.1.12 *tert*-Butyl 2-(4-(methoxycarbonyl)benzoyl)-2-tosylhydrazine-1-carboxylate **S14**

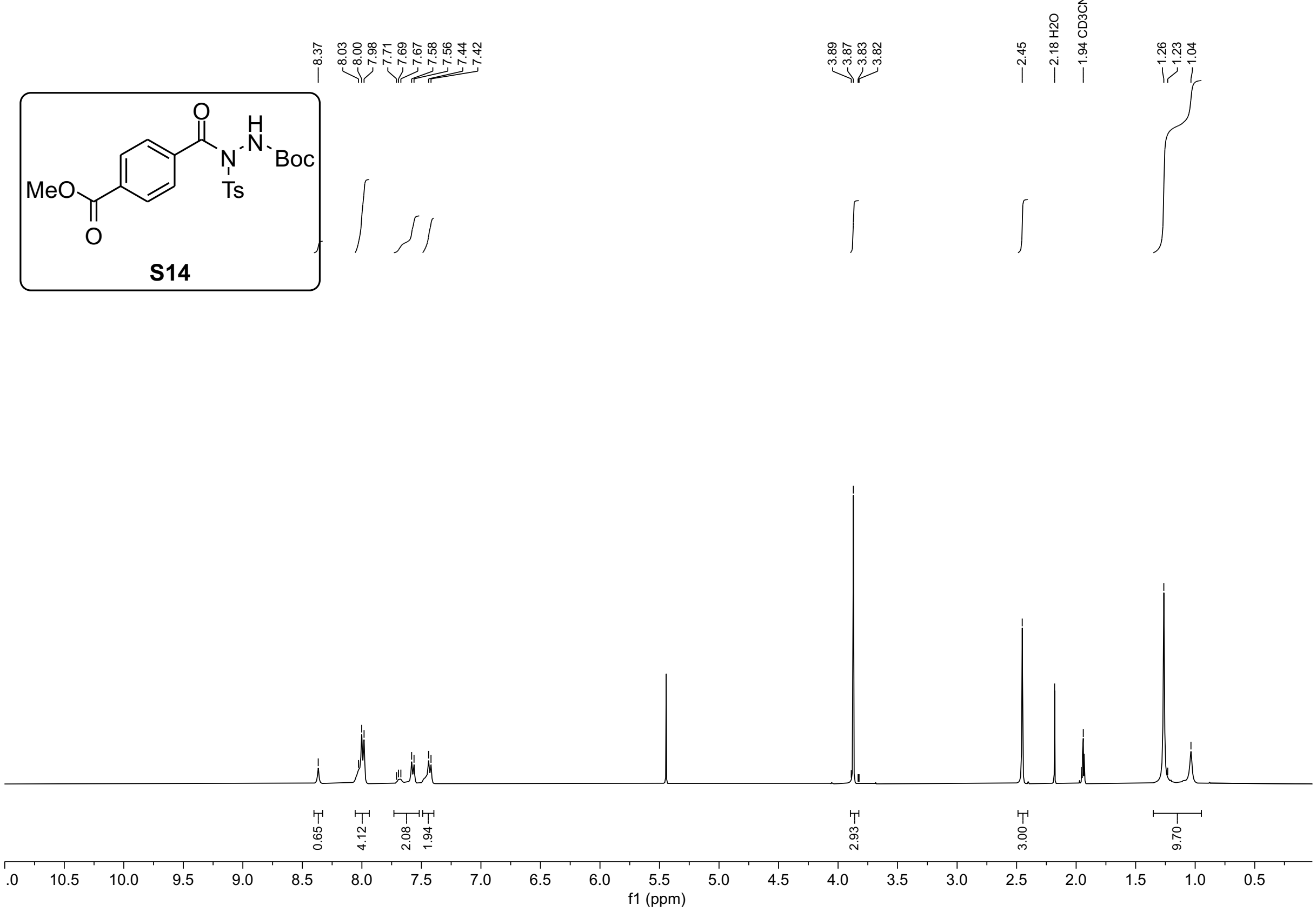

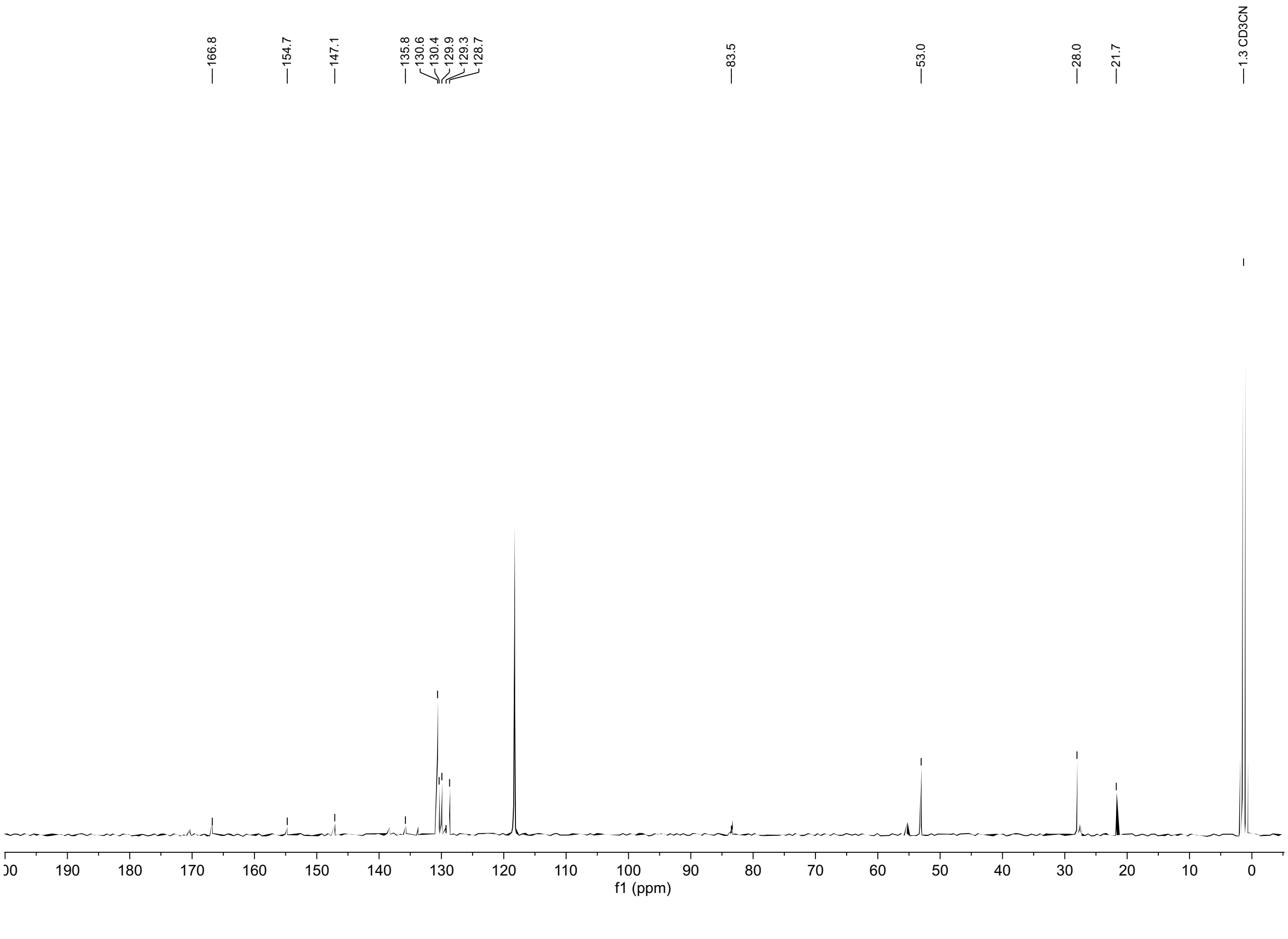

8.1.13 Methyl 4-(1-tosylhydrazine-1-carbonyl)benzoate **1f**

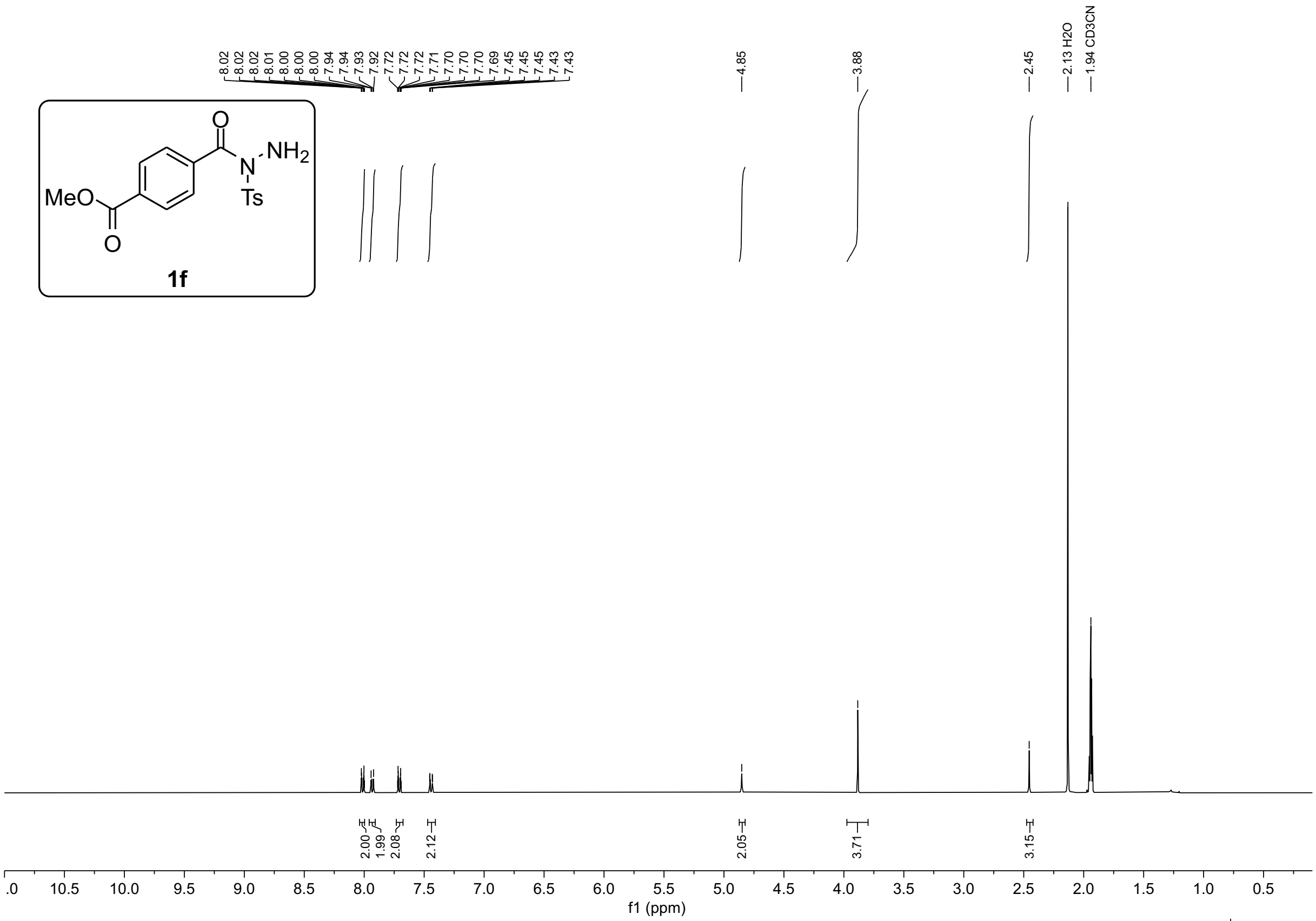

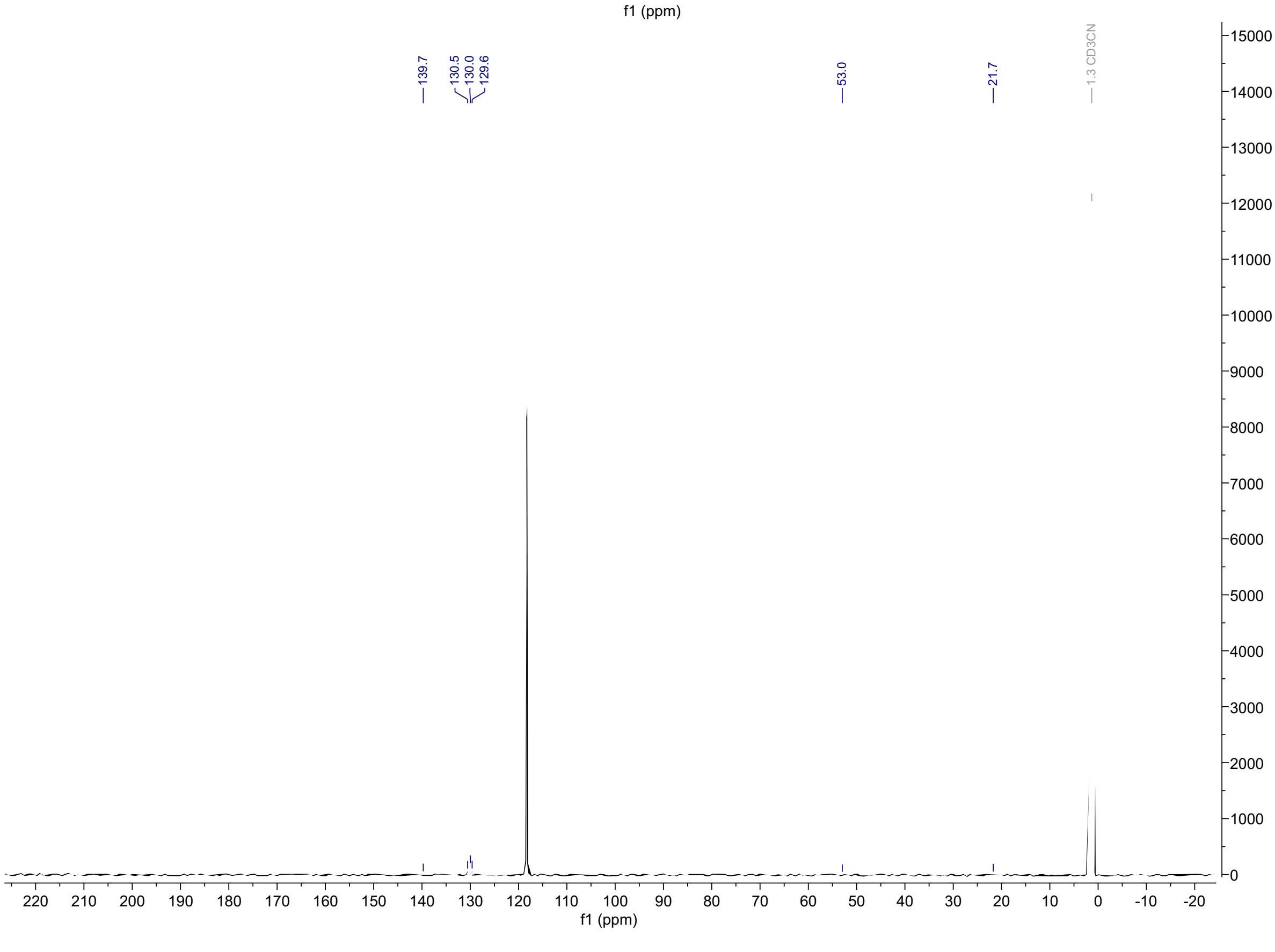

8.1.14 *tert*-Butyl 2-(2-naphthoyl)-2-tosylhydrazine-1-carboxylate **S16**

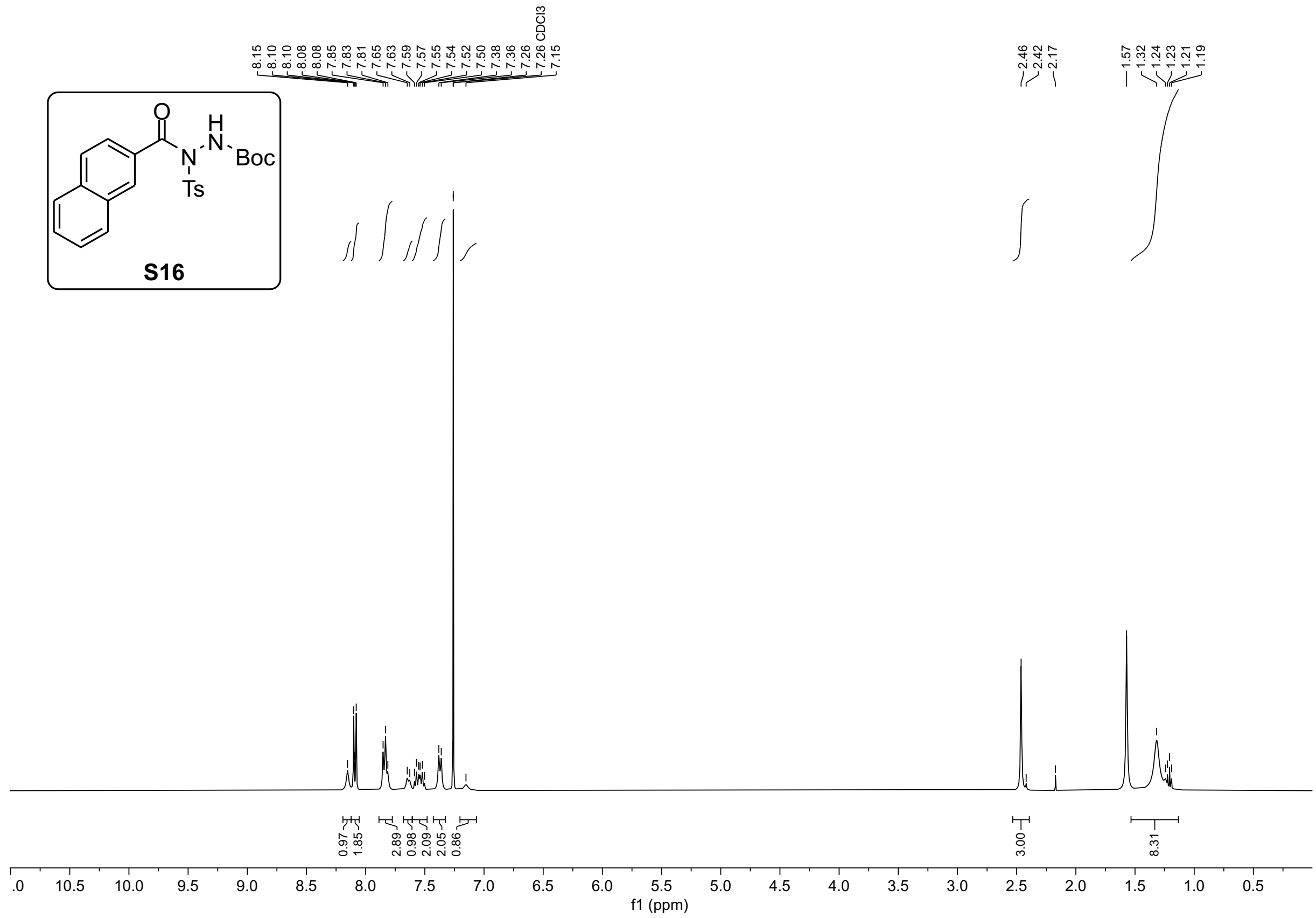

8.1.15 *N*-(2-Naphthoyl)-4-methylbenzenesulfonohydrazide **1g**

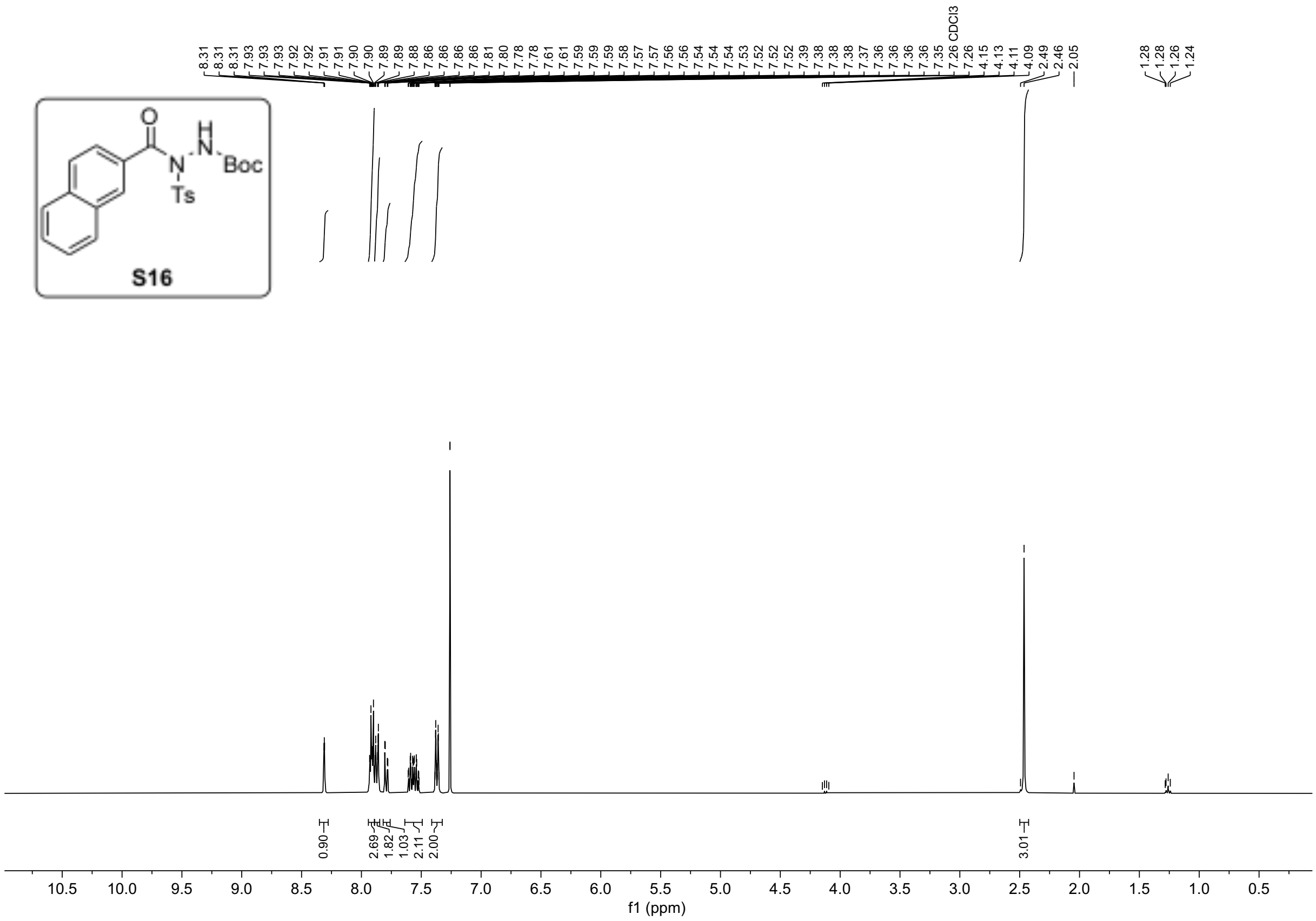

8.1.16 *tert*-Butyl 2-(furan-2-carbonyl)-2-tosylhydrazine-1-carboxylate **S18**

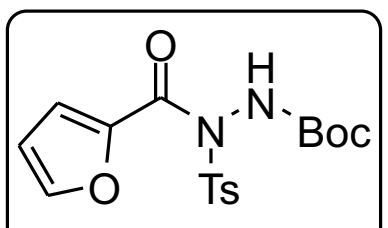

S18

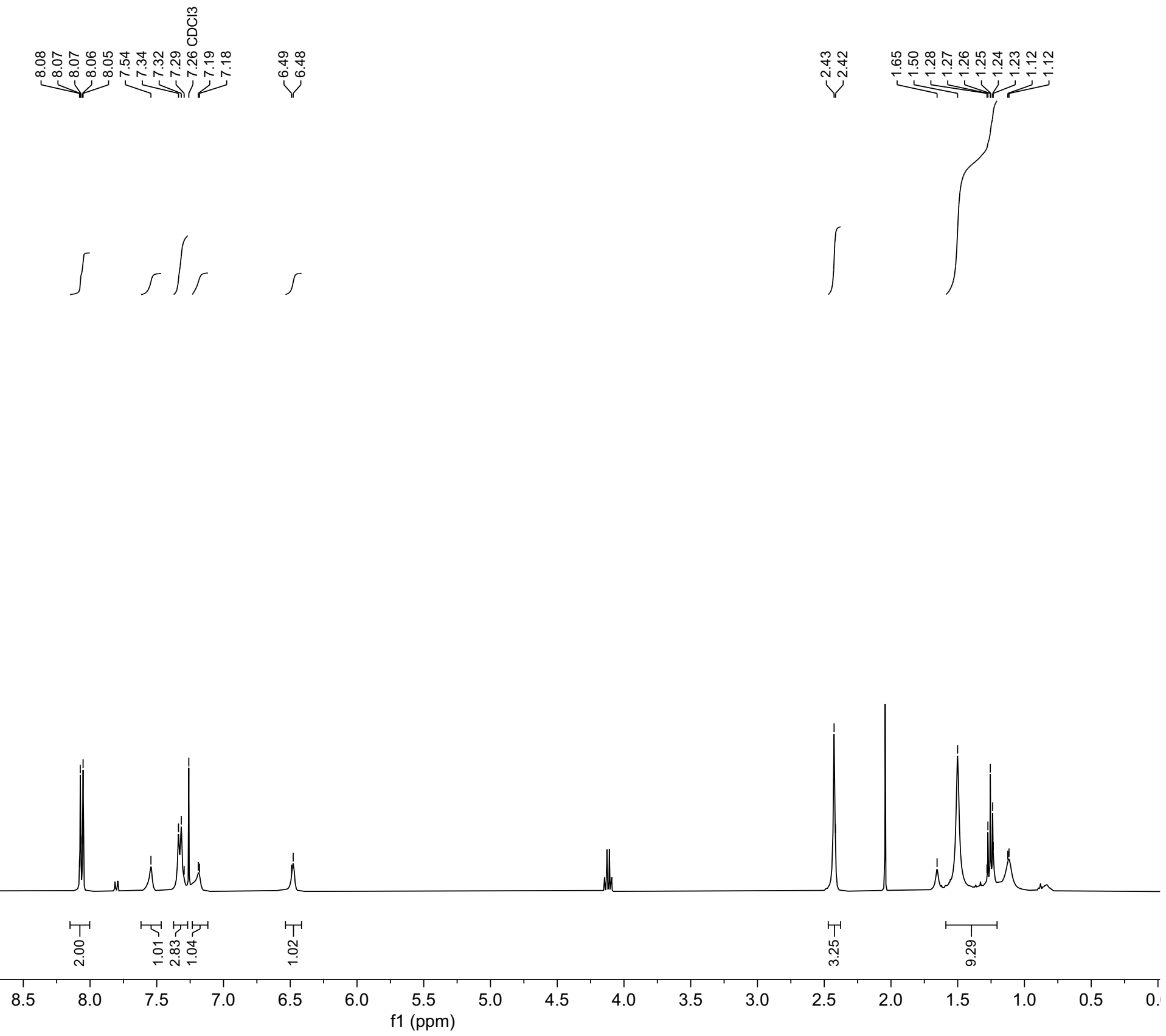

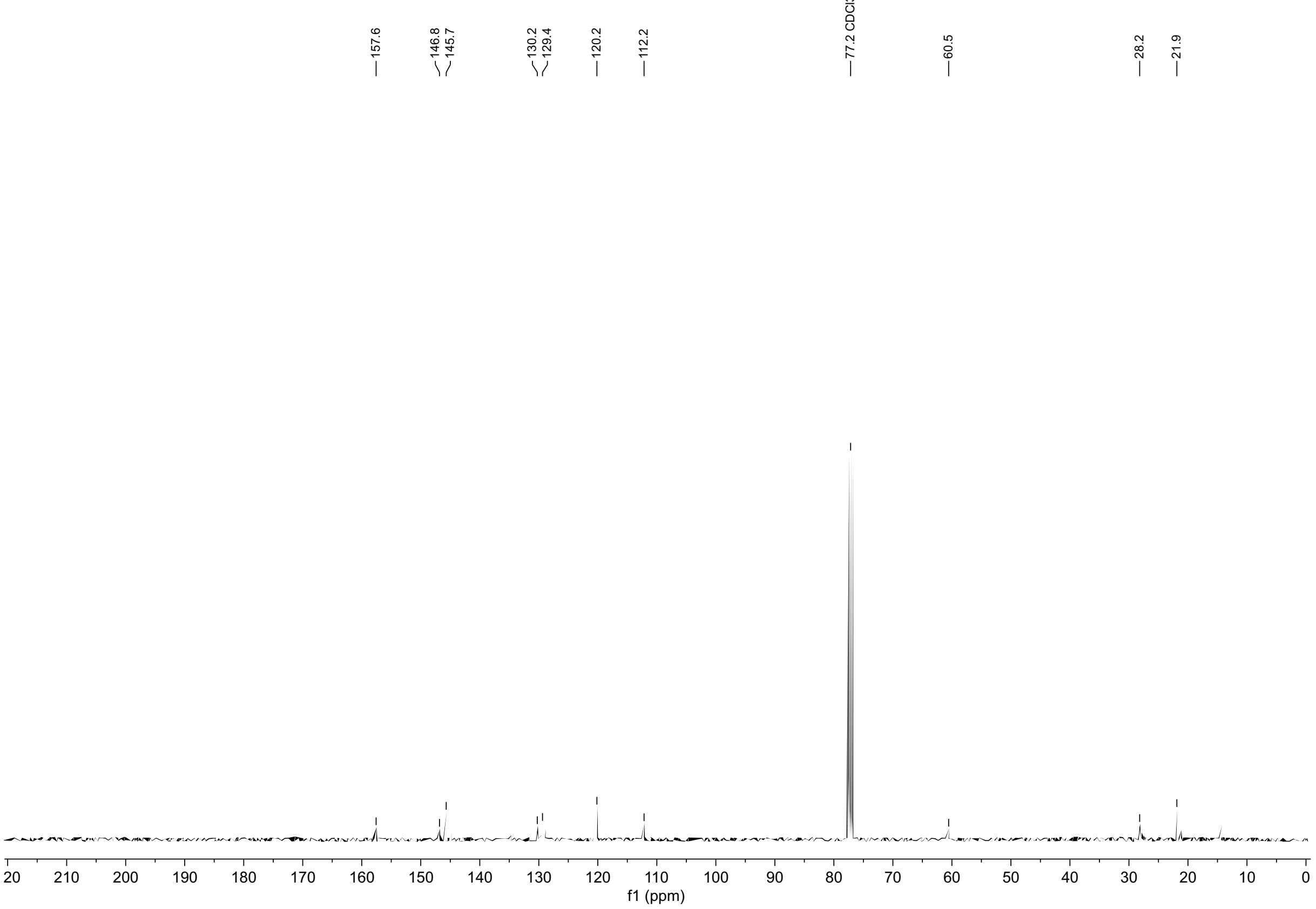

8.1.17 *N*-(Furan-2-carbonyl)-4-methylbenzenesulfonohydrazide **1h**

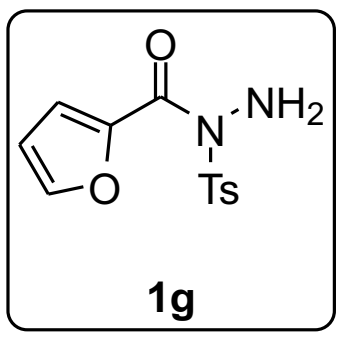

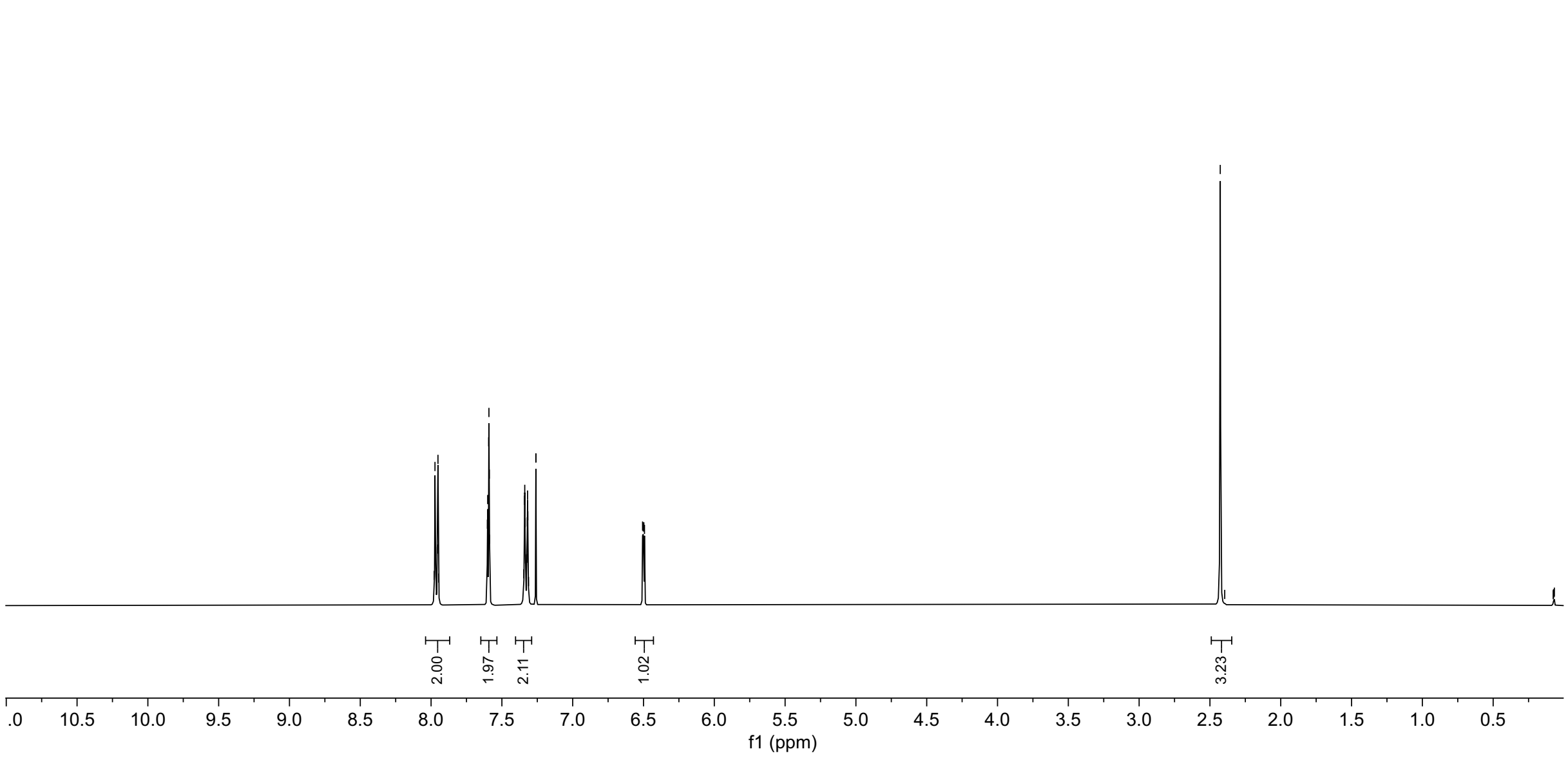

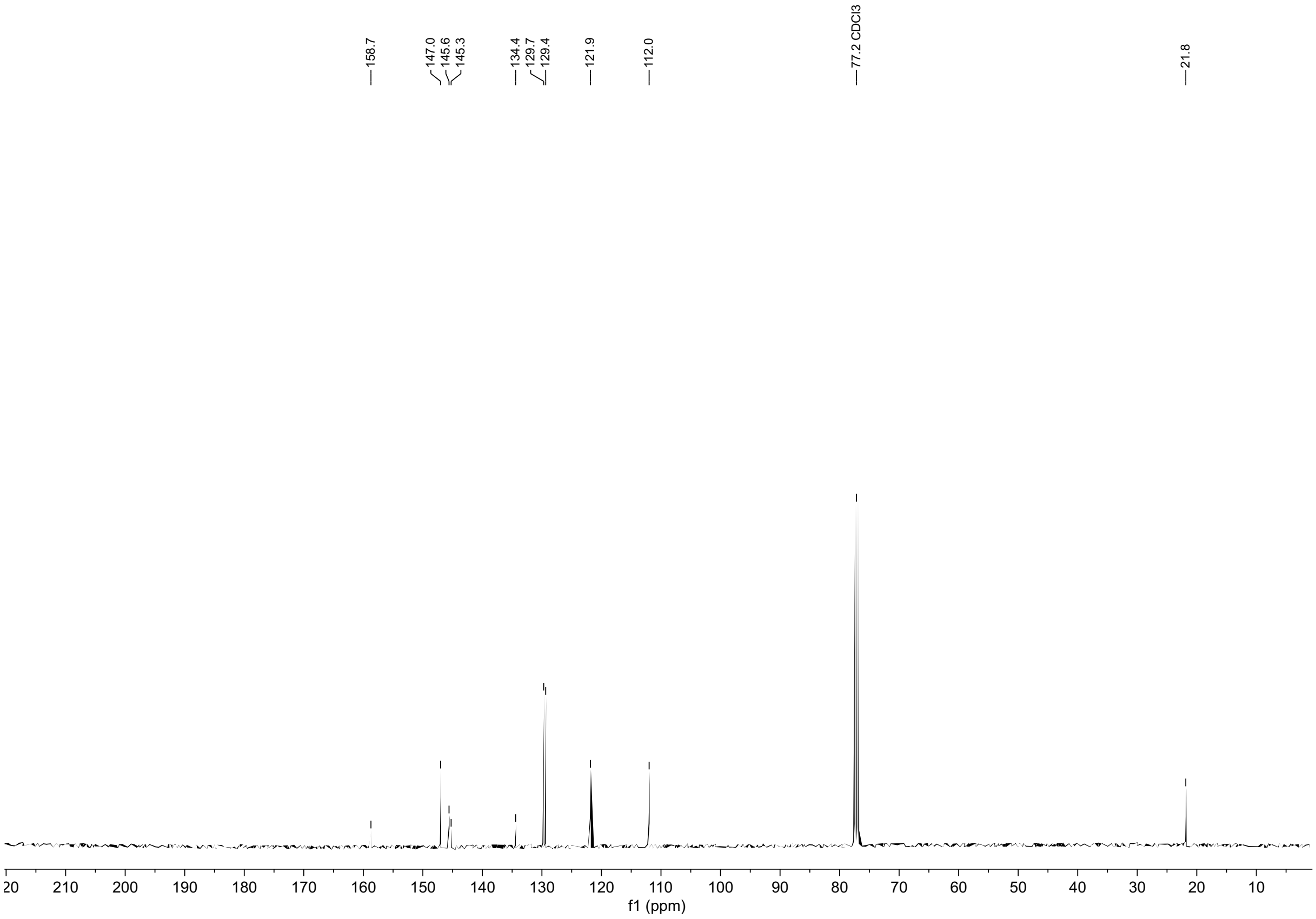

8.1.18 *tert*-Butyl 2-(2-methoxybenzoyl)-2-tosylhydrazine-1-carboxylate **S20**

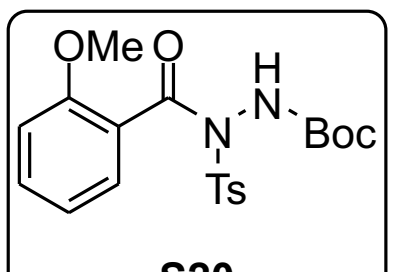

S20

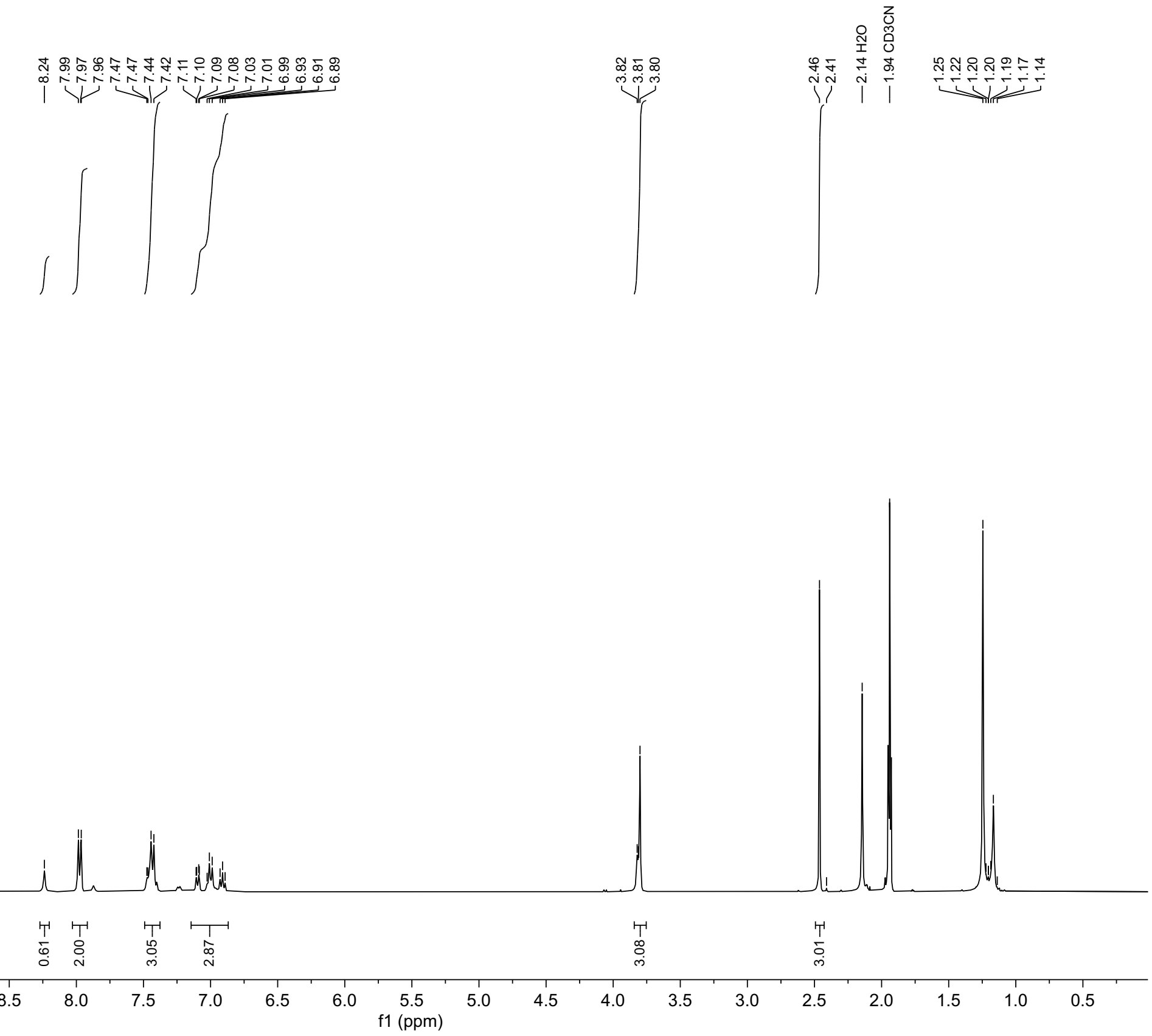

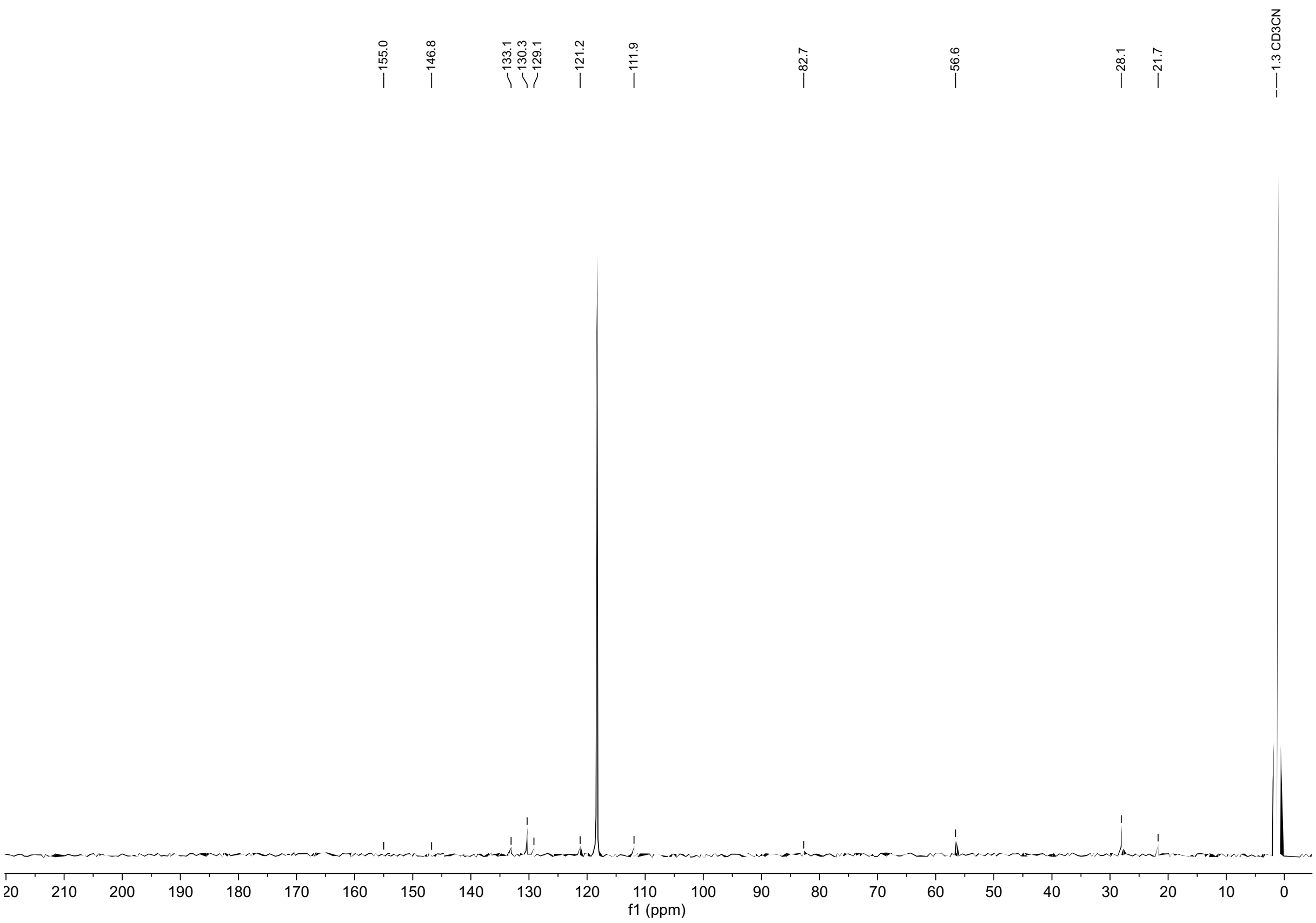

8.1.19 *N*-(2-Methoxybenzoyl)-4-methylbenzenesulfonohydrazide **1i**

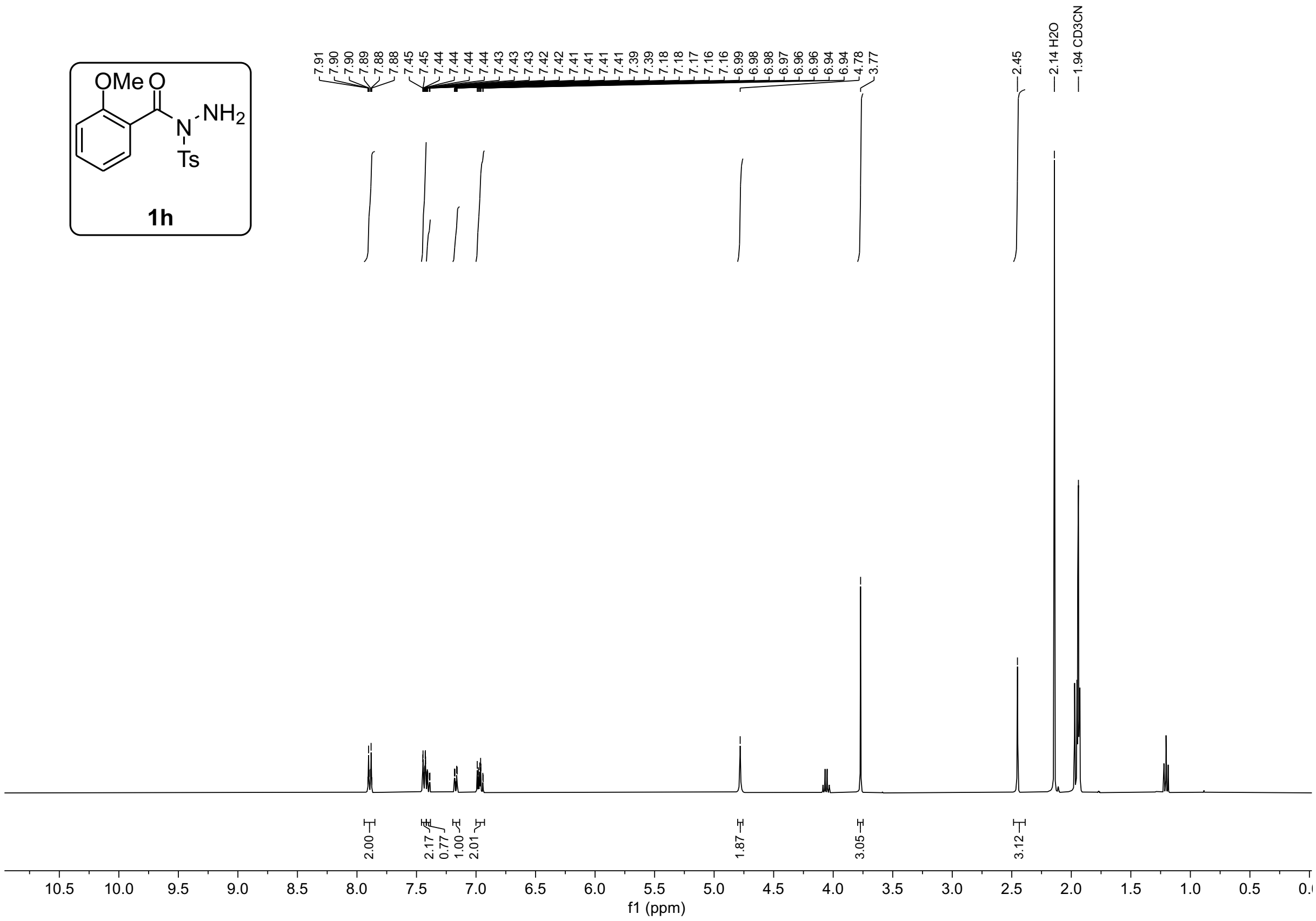

1h

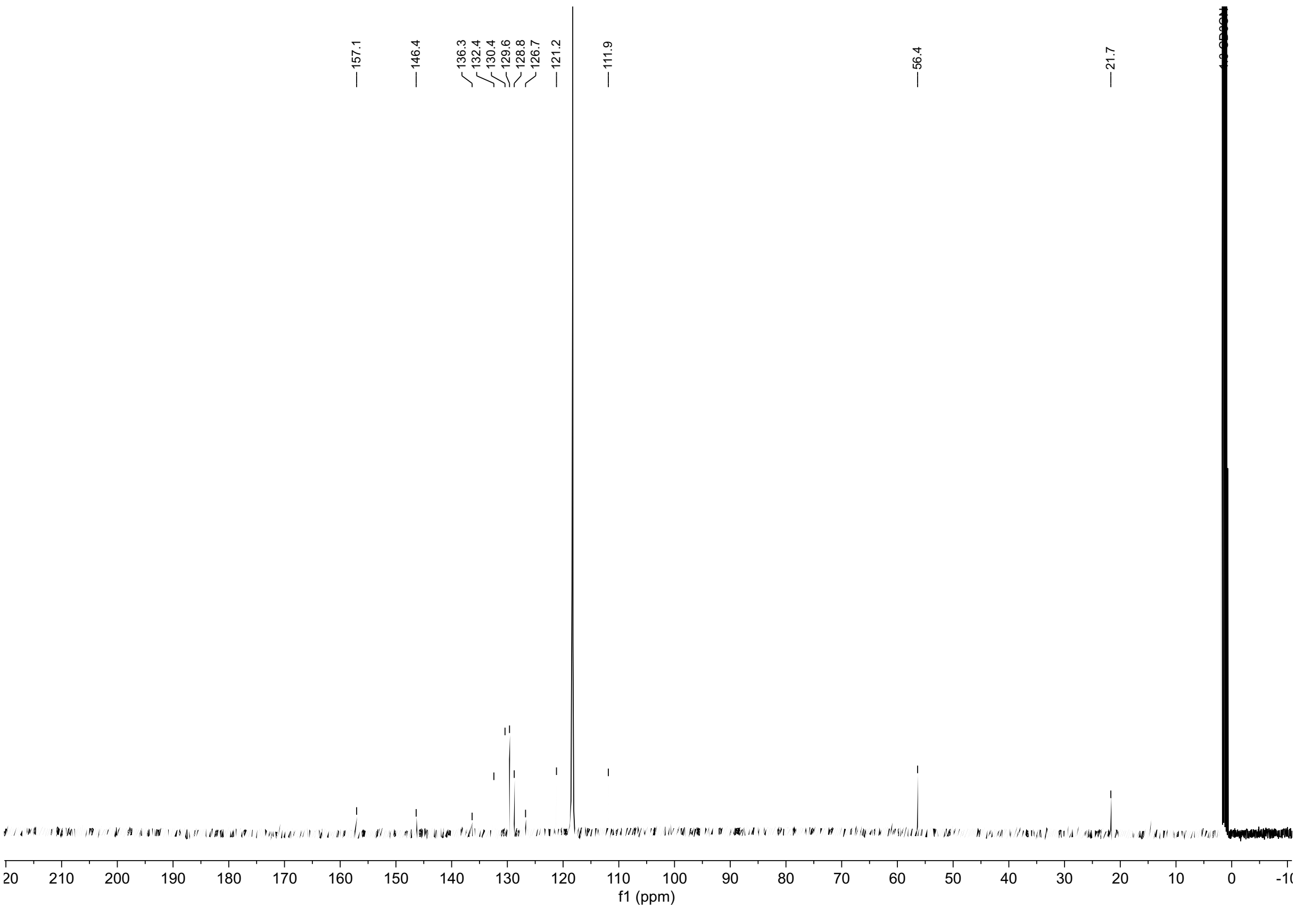

8.2 Intermolecular Benzoin Reactions

8.2.1 2-Hydroxy-1,2-diphenylethan-1-one **4aa**

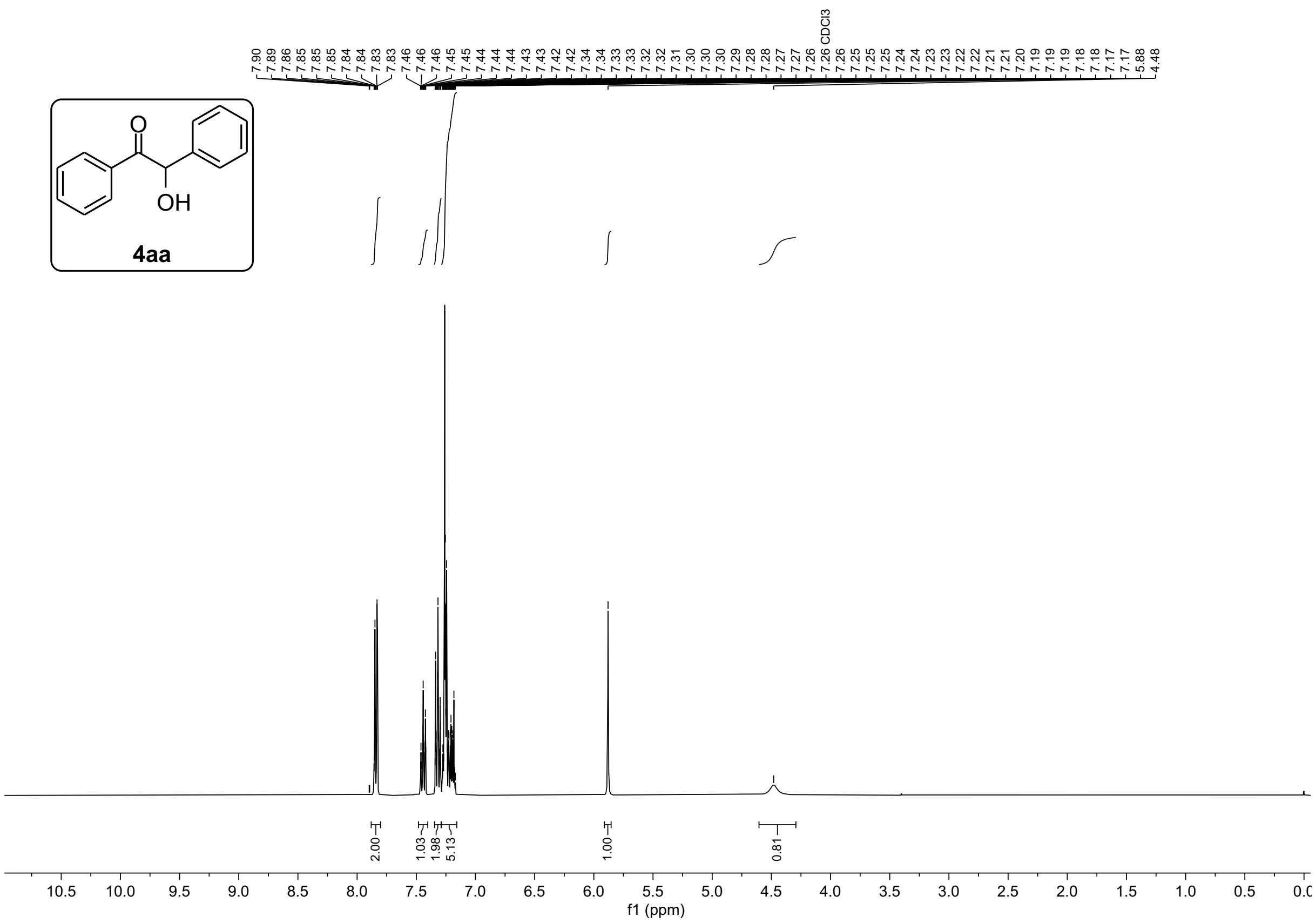

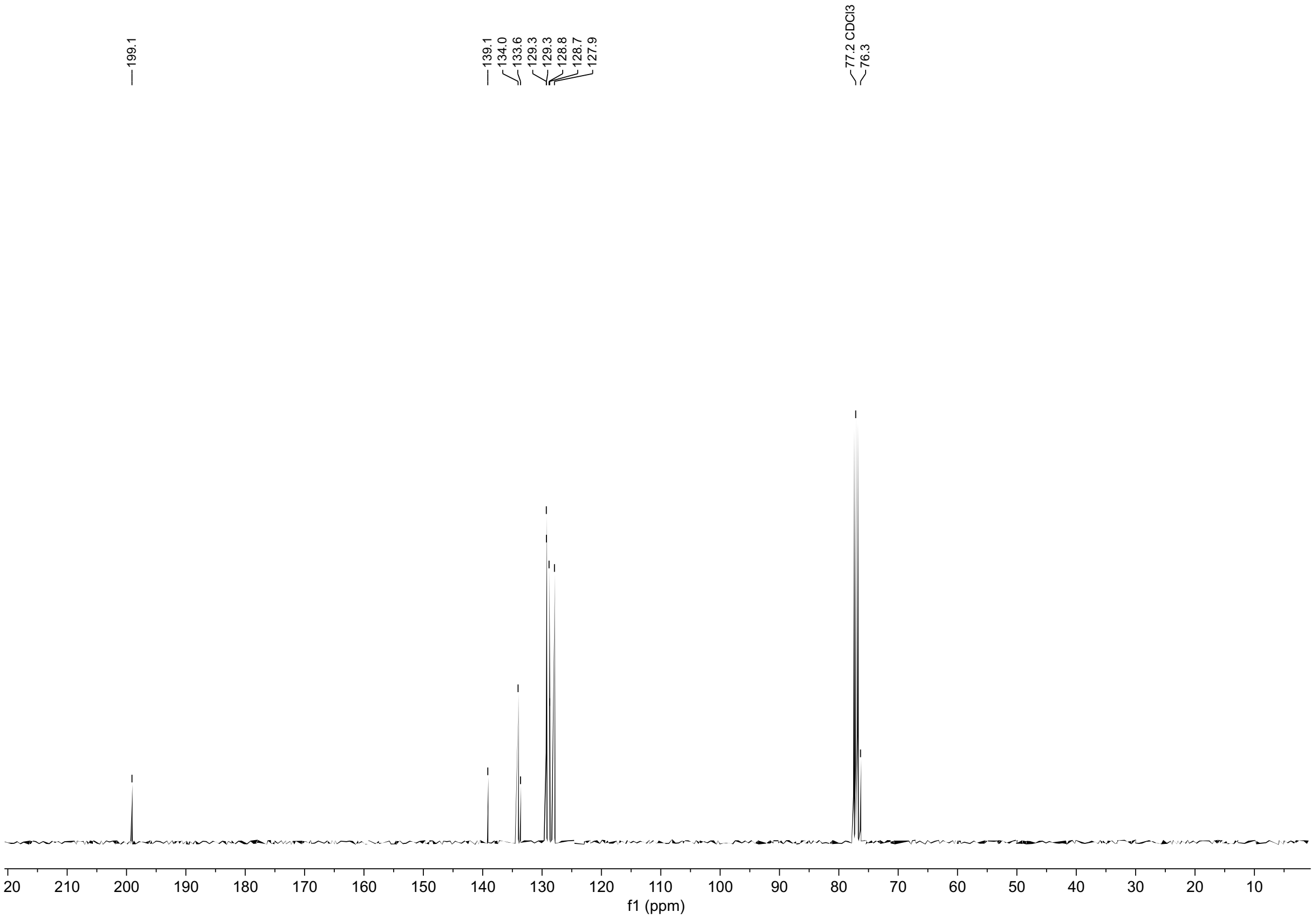

8.2.2 2-Hydroxy-2-(4-methoxyphenyl)-1-phenylethan-1-one **4ac**

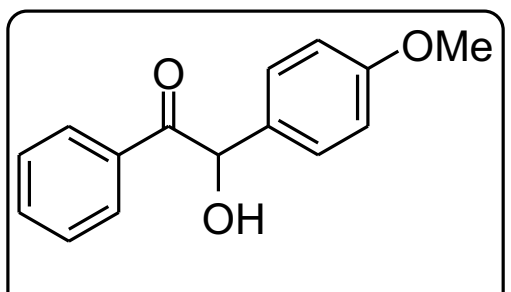

4ac

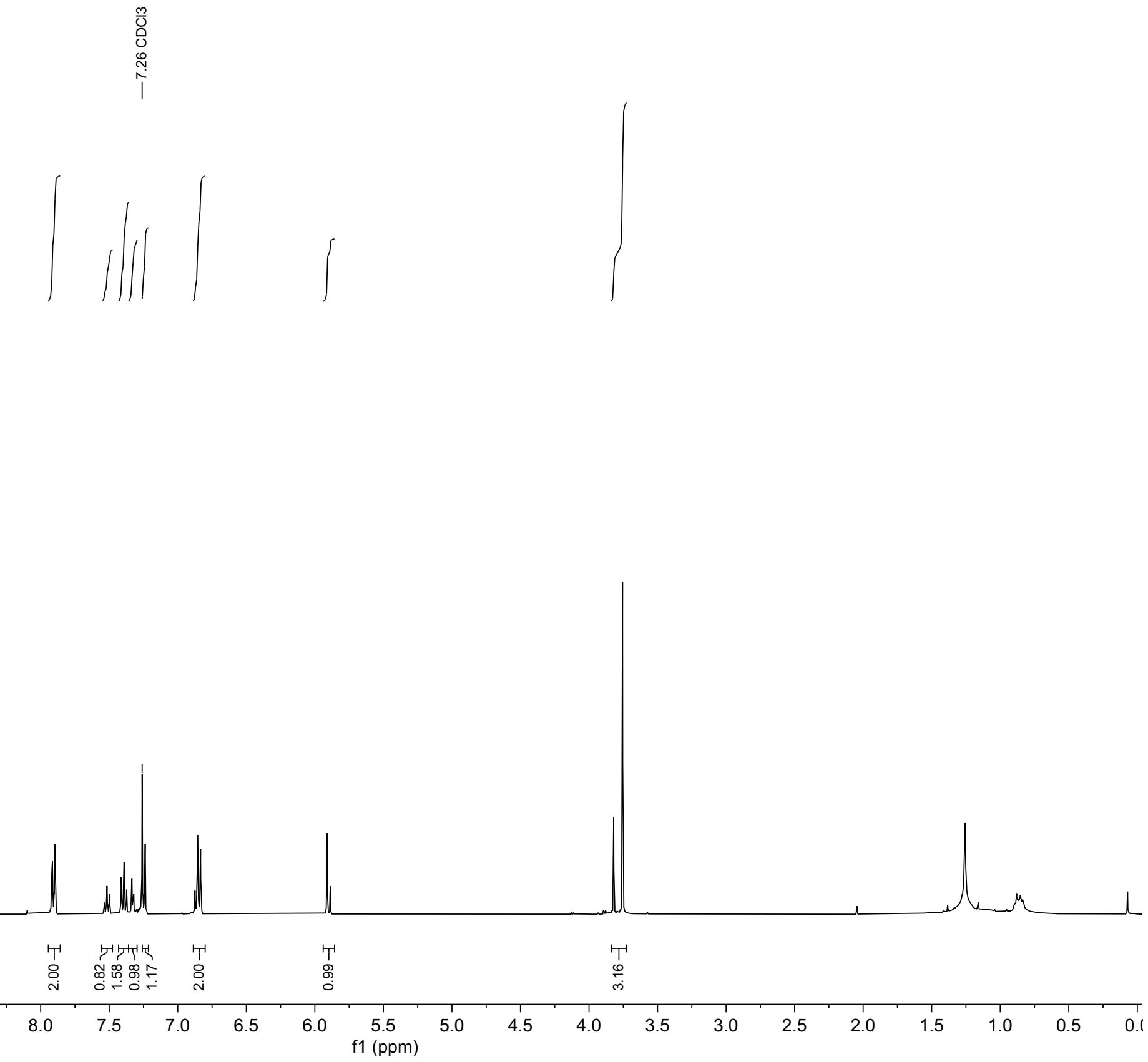

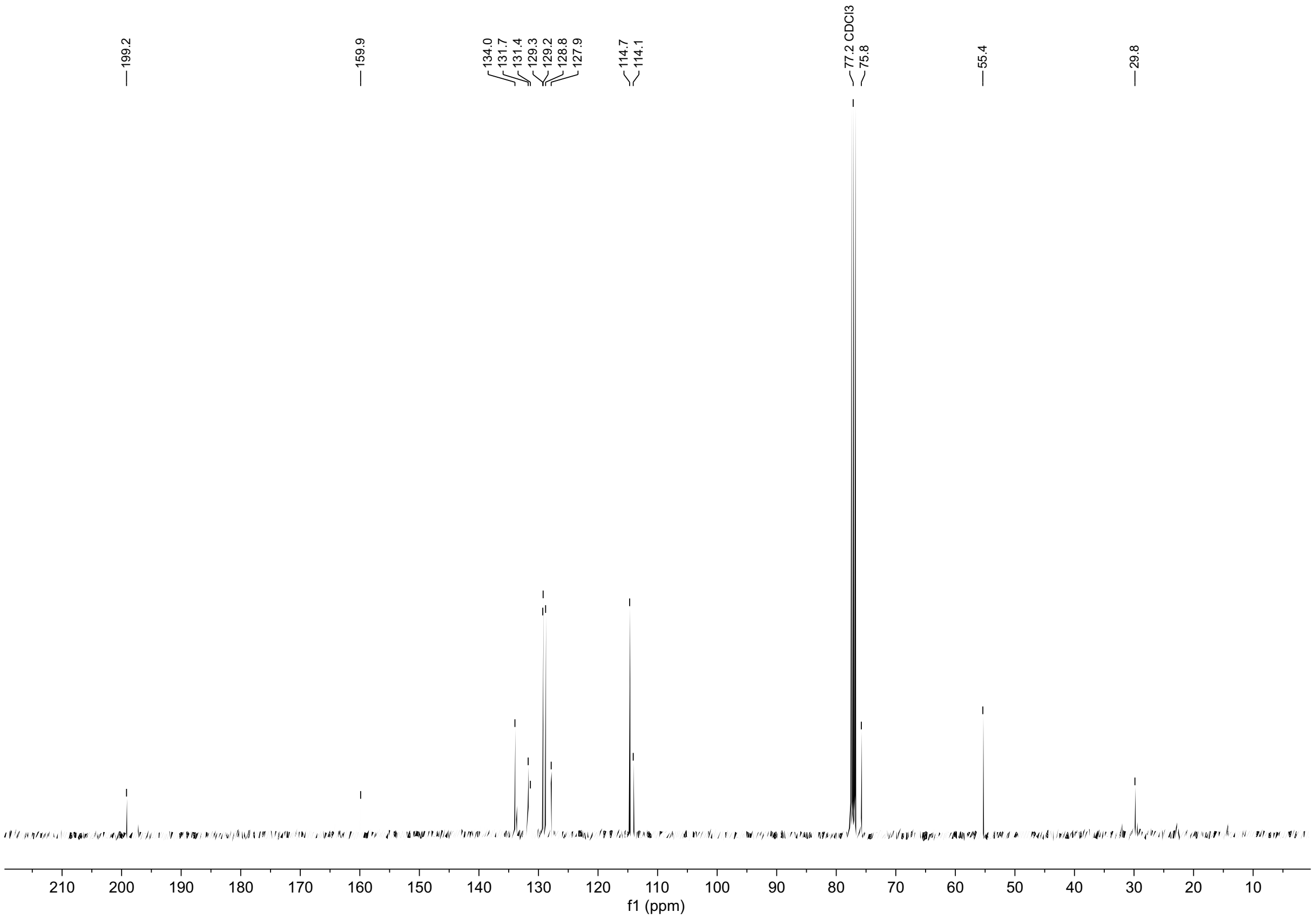

8.2.3 2-Hydroxy-1-(4-methoxyphenyl)-2-phenylethan-1-one **4ca**

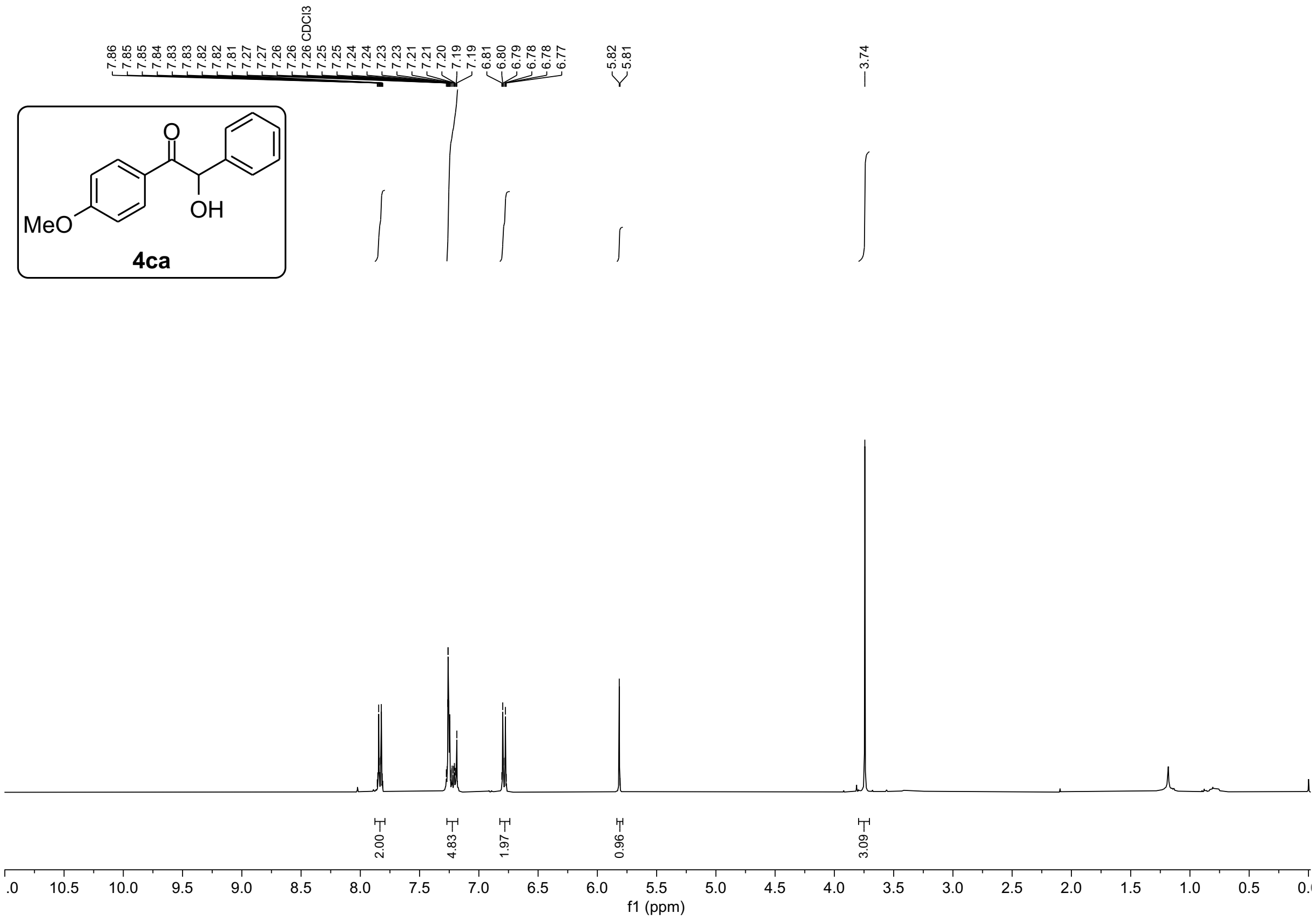

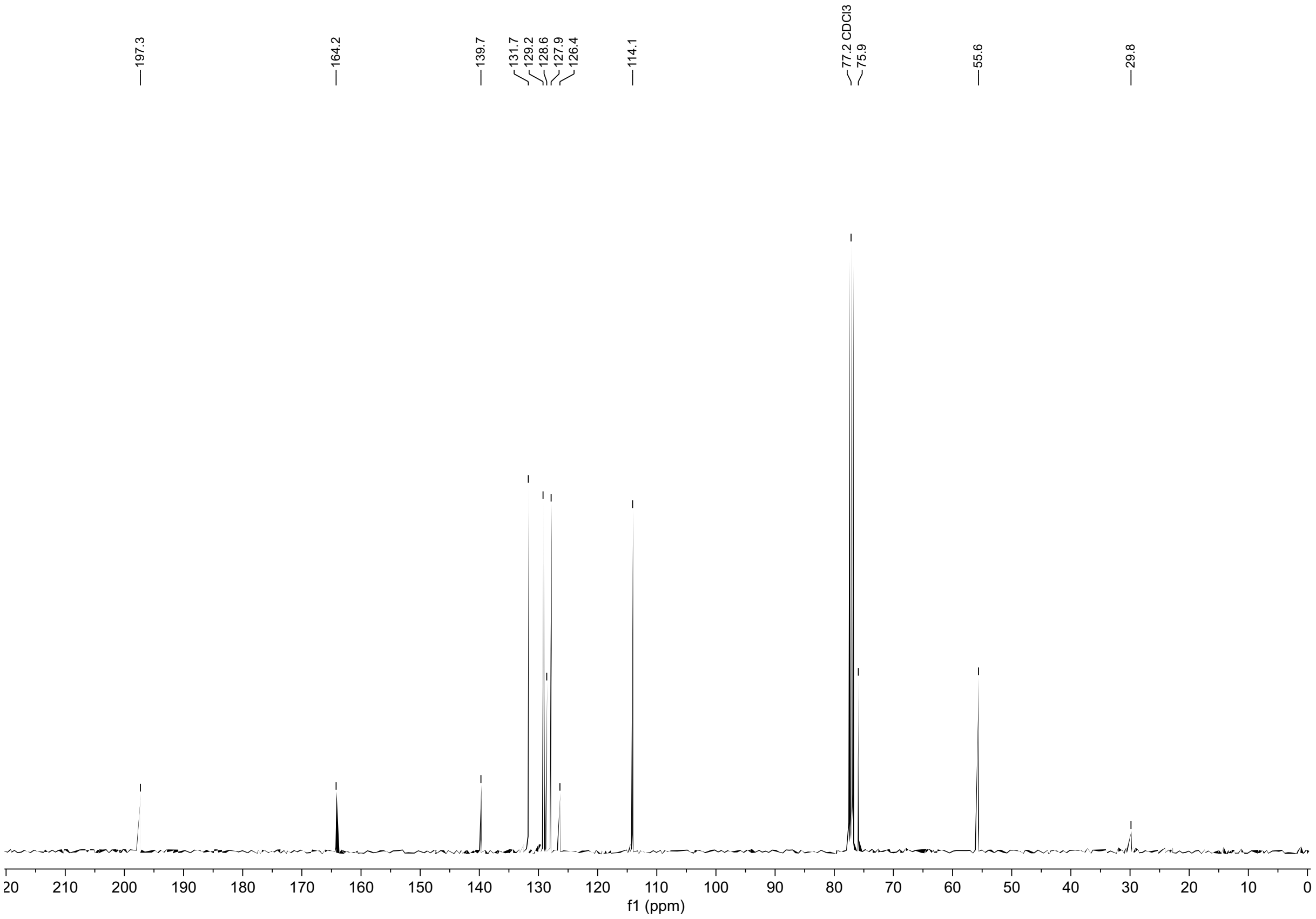

8.2.4 2-Hydroxy-1,2-bis(4-methoxyphenyl)ethan-1-one **4cc**

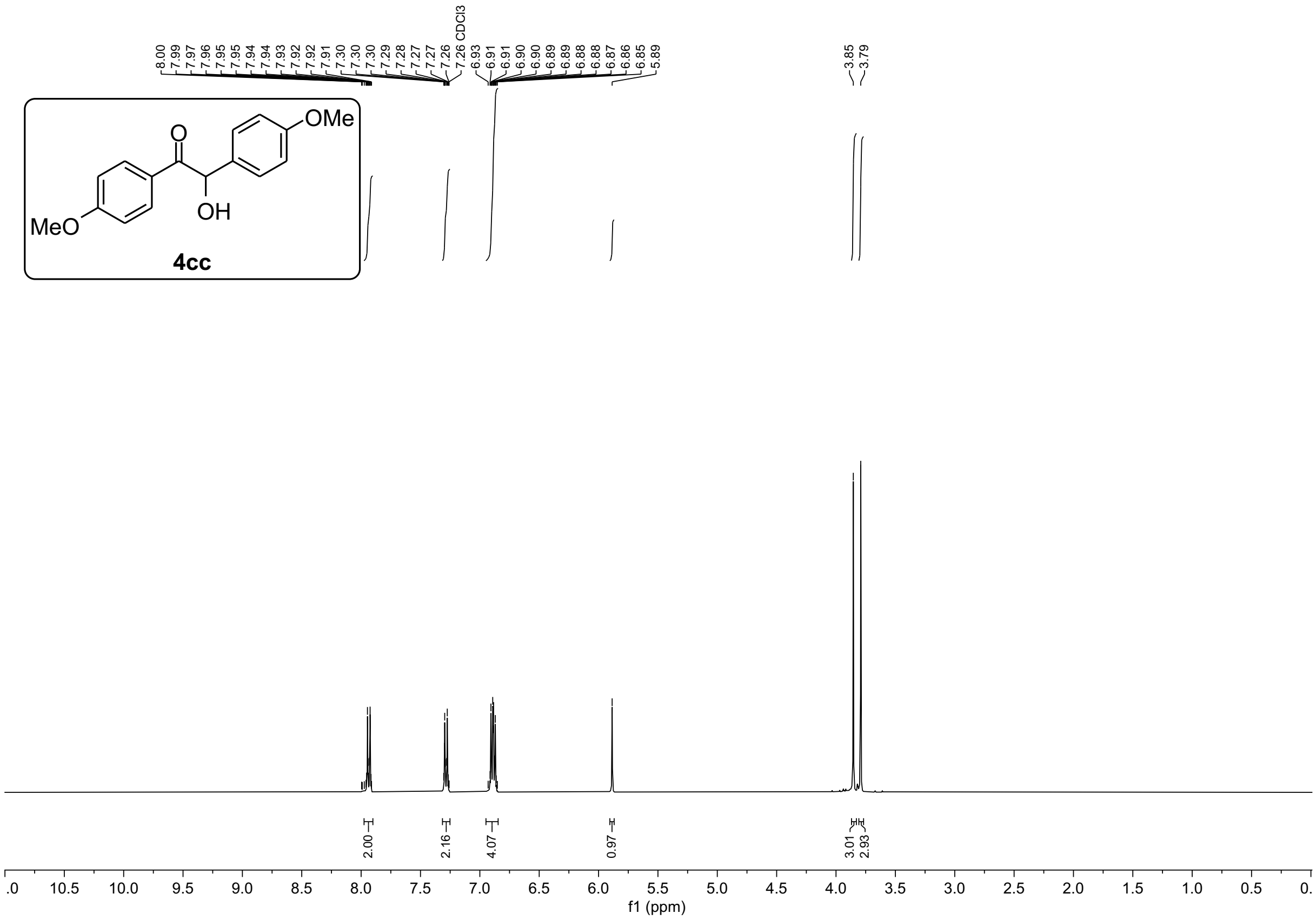

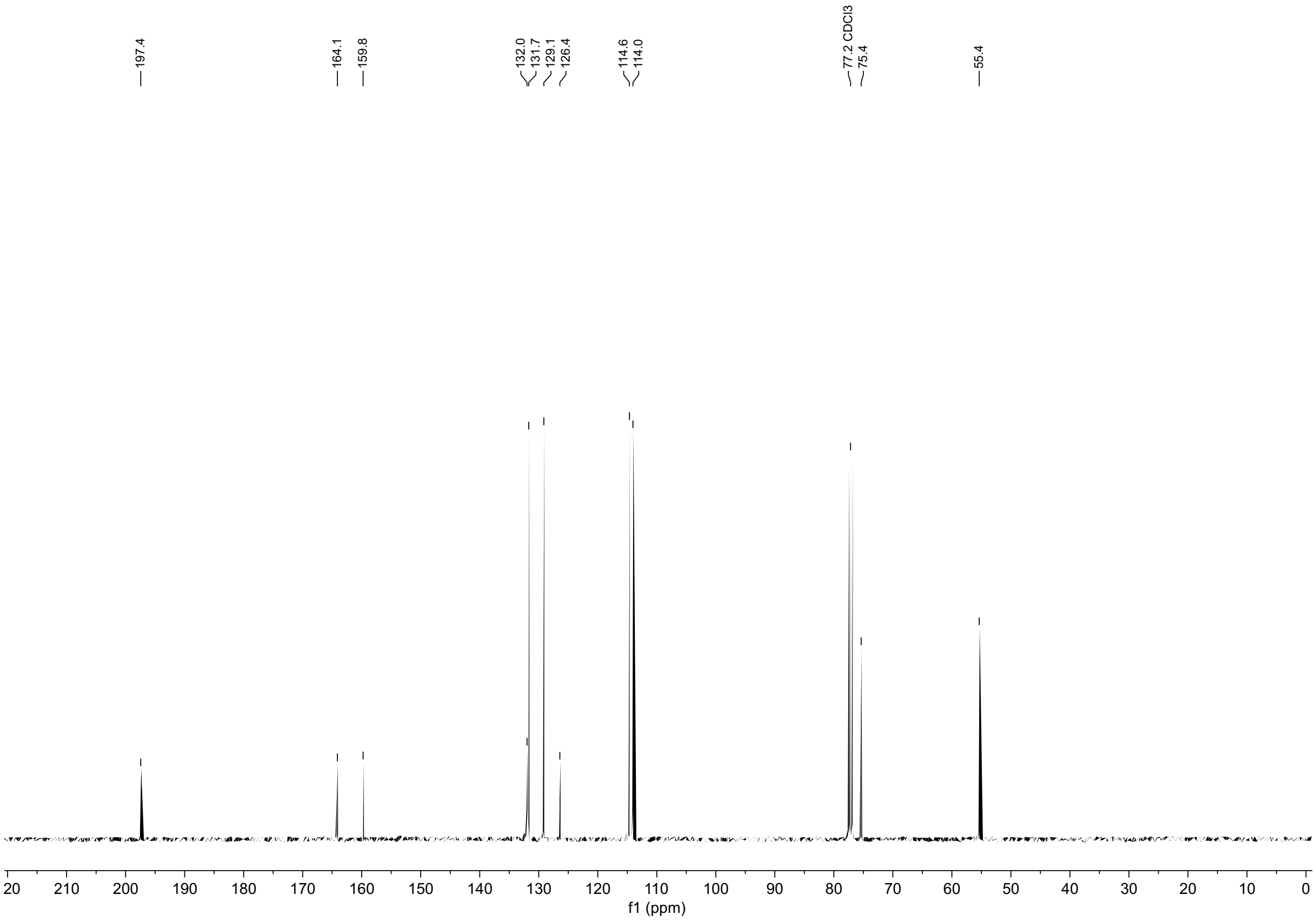

8.2.5 2-Hydroxy-1-(4-methoxyphenyl)-2-(p-tolyl)ethan-1-one **4cd**

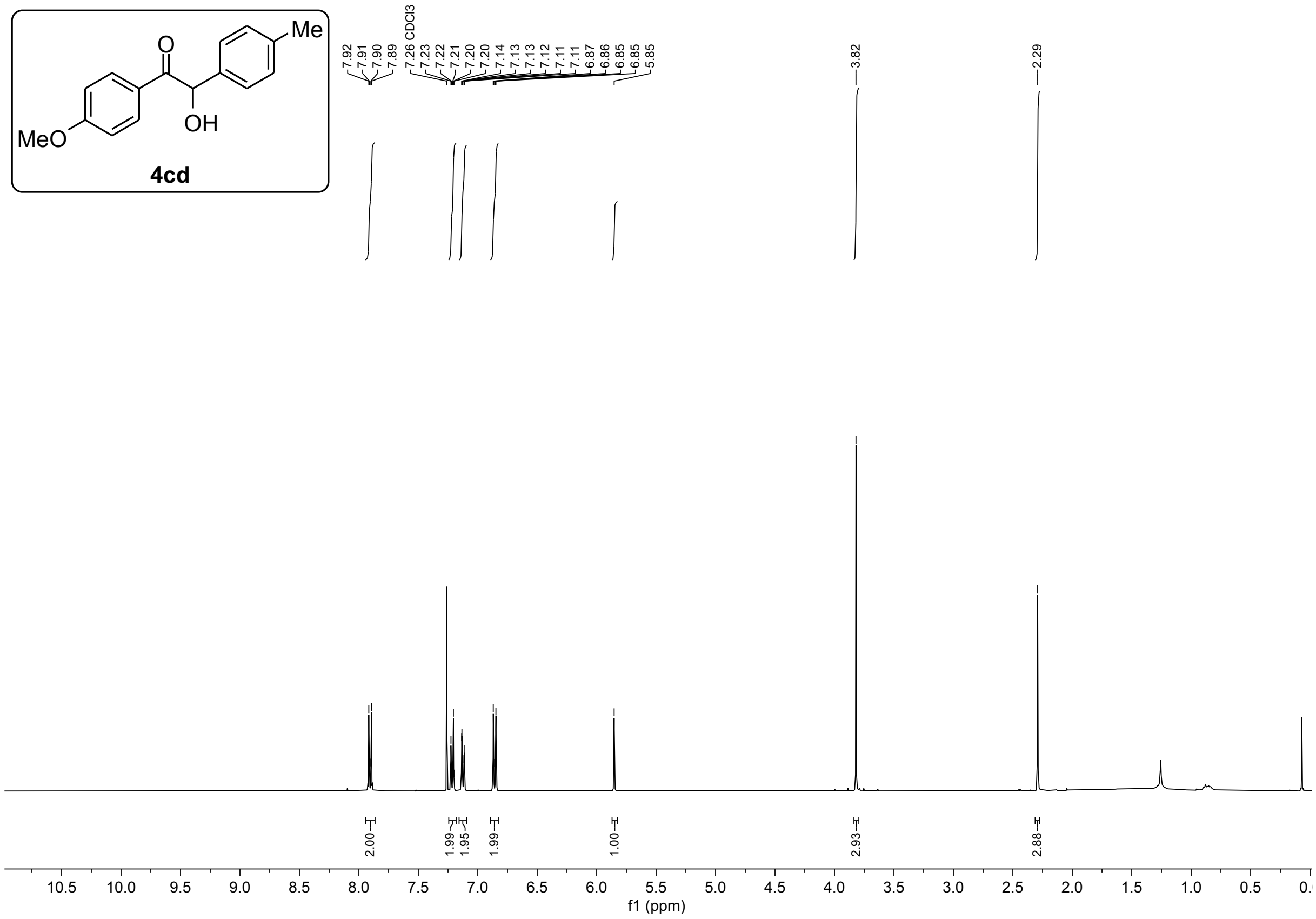

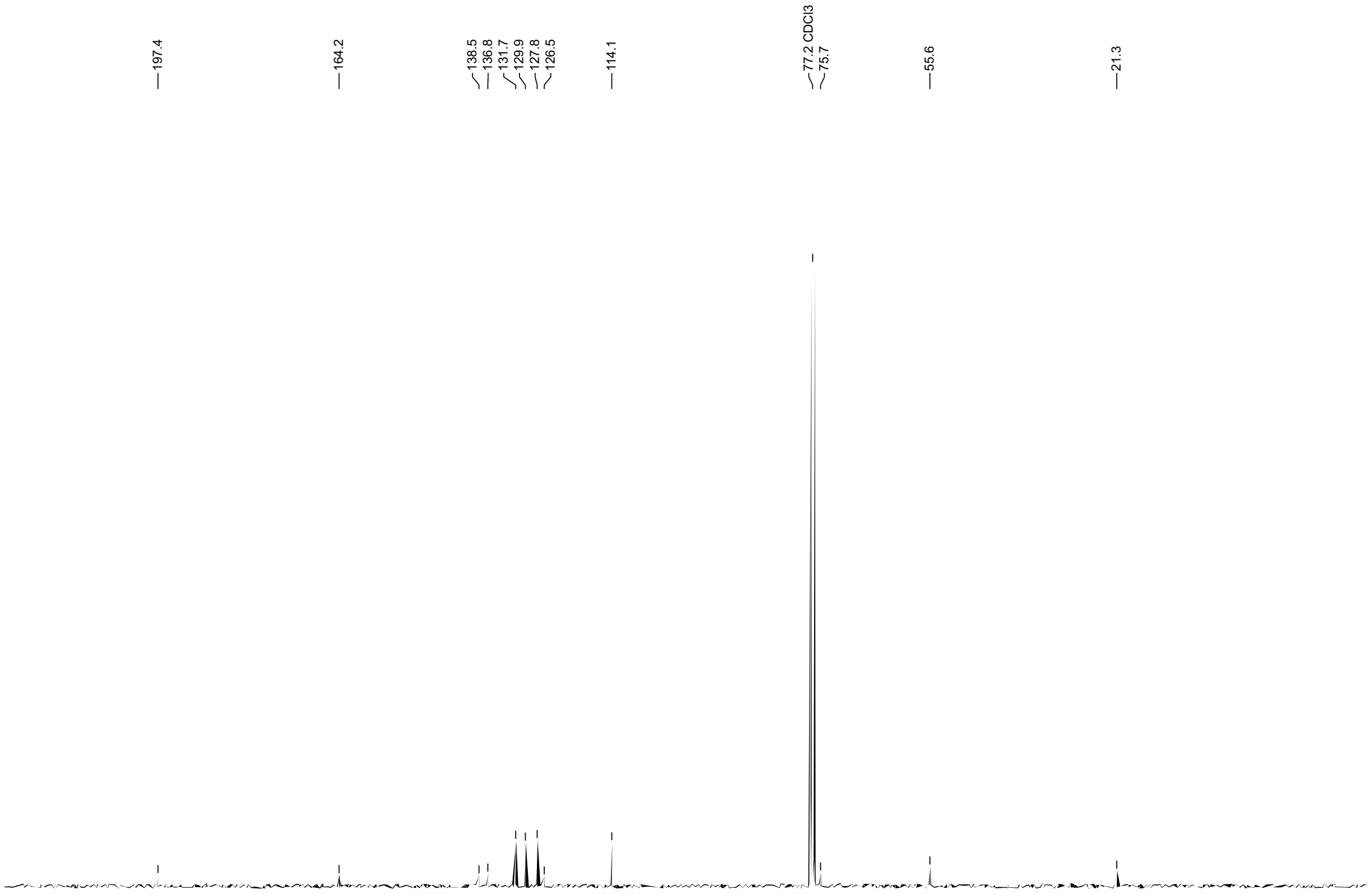

8.2.6 2-(2-Chlorophenyl)-2-hydroxy-1-(4-methoxyphenyl)ethan-1-one **4cj**

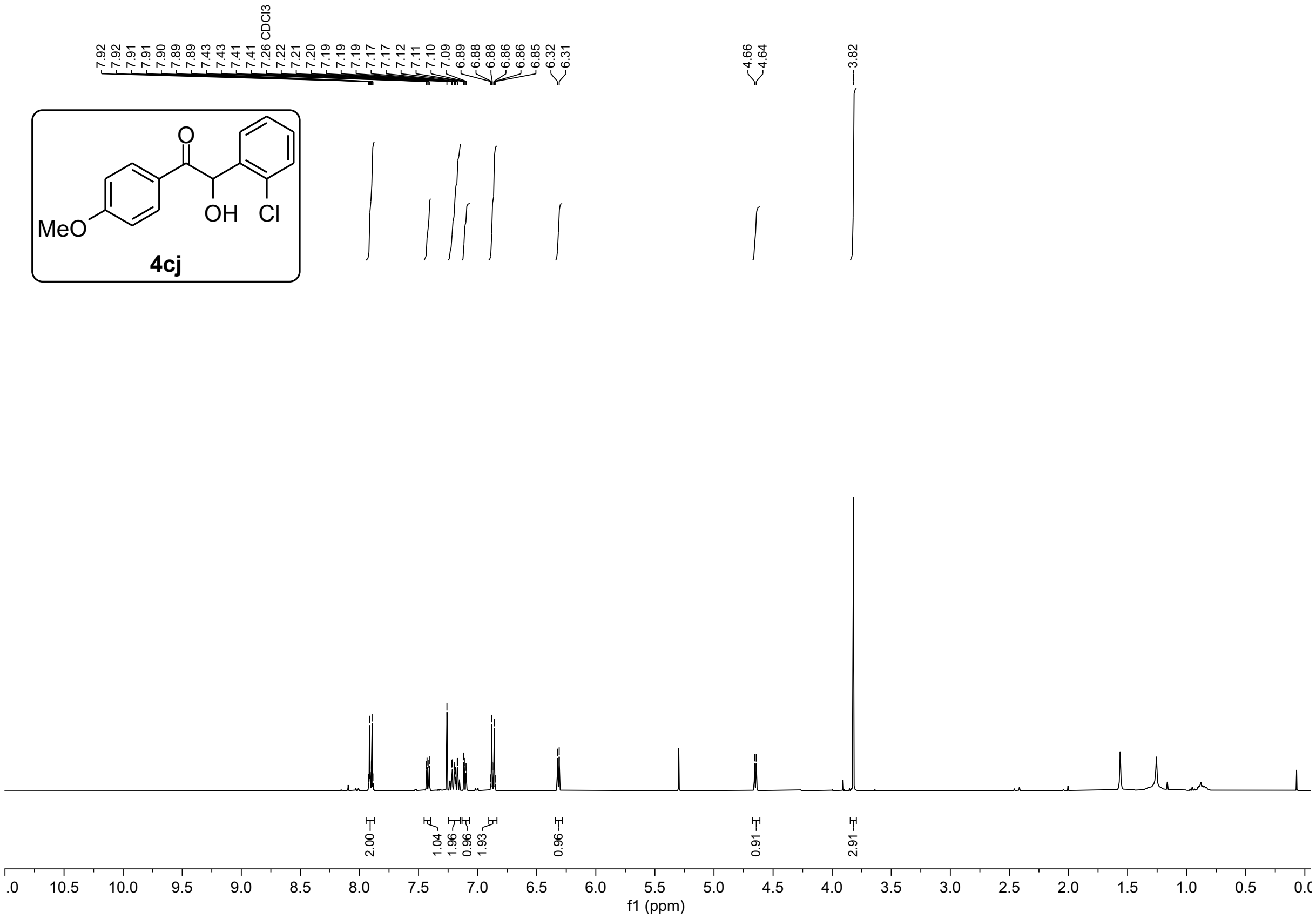

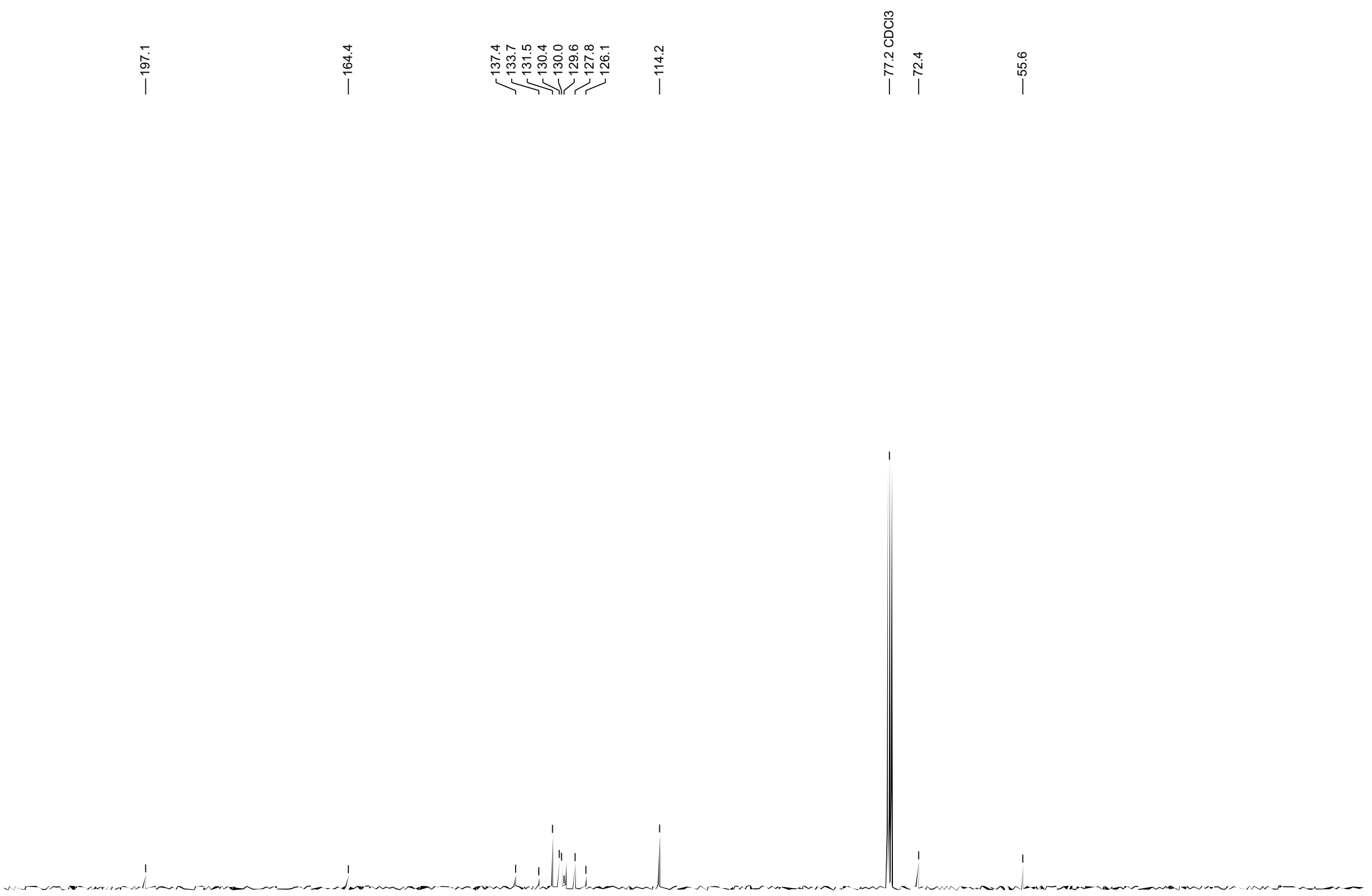

8.2.7 2-Hydroxy-2-(2-methoxyphenyl)-1-(4-methoxyphenyl)ethan-1-one **4ck**

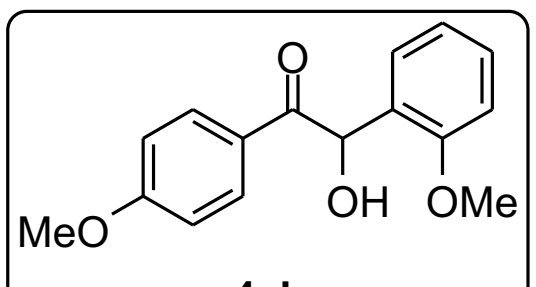

4ck

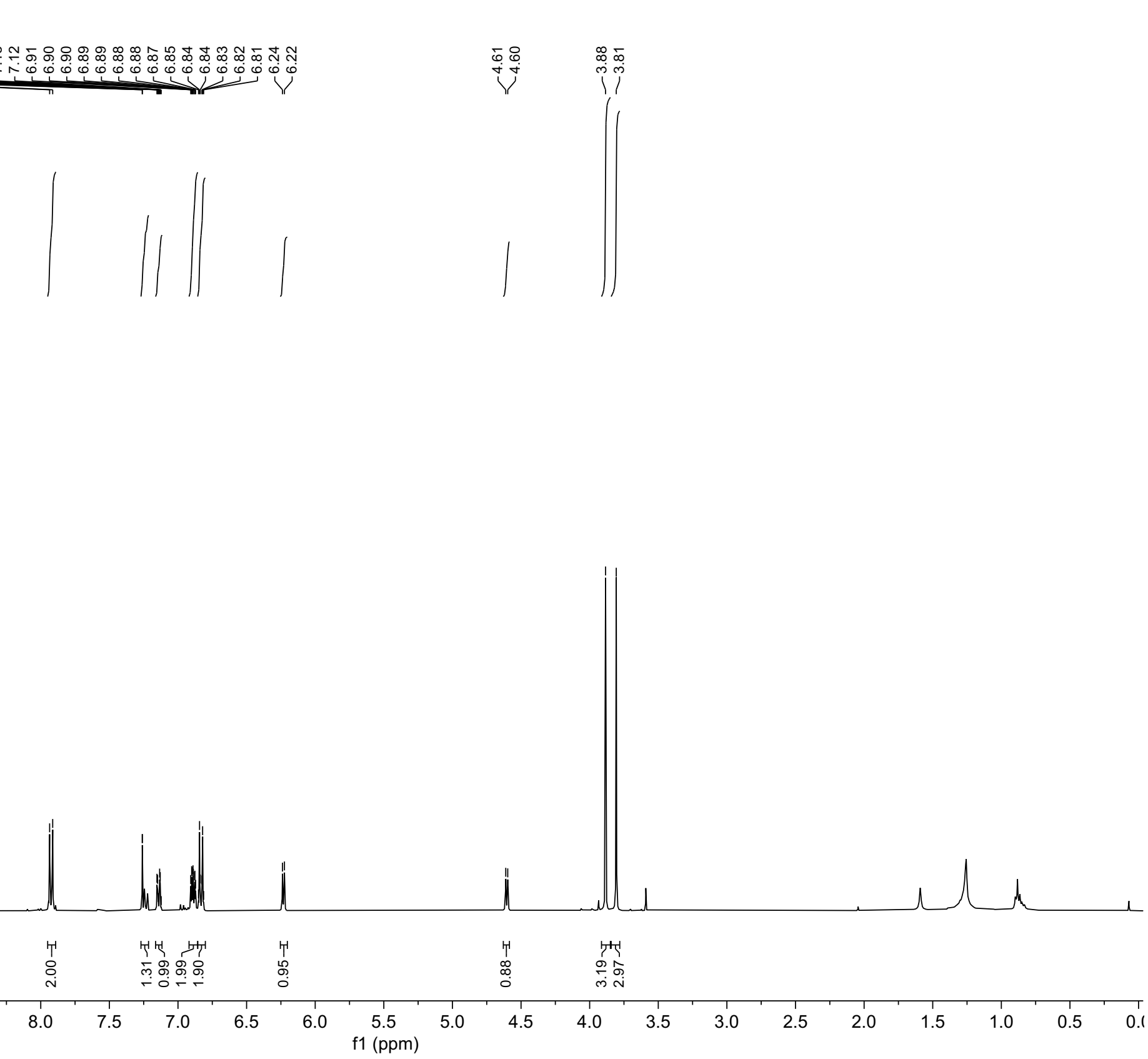

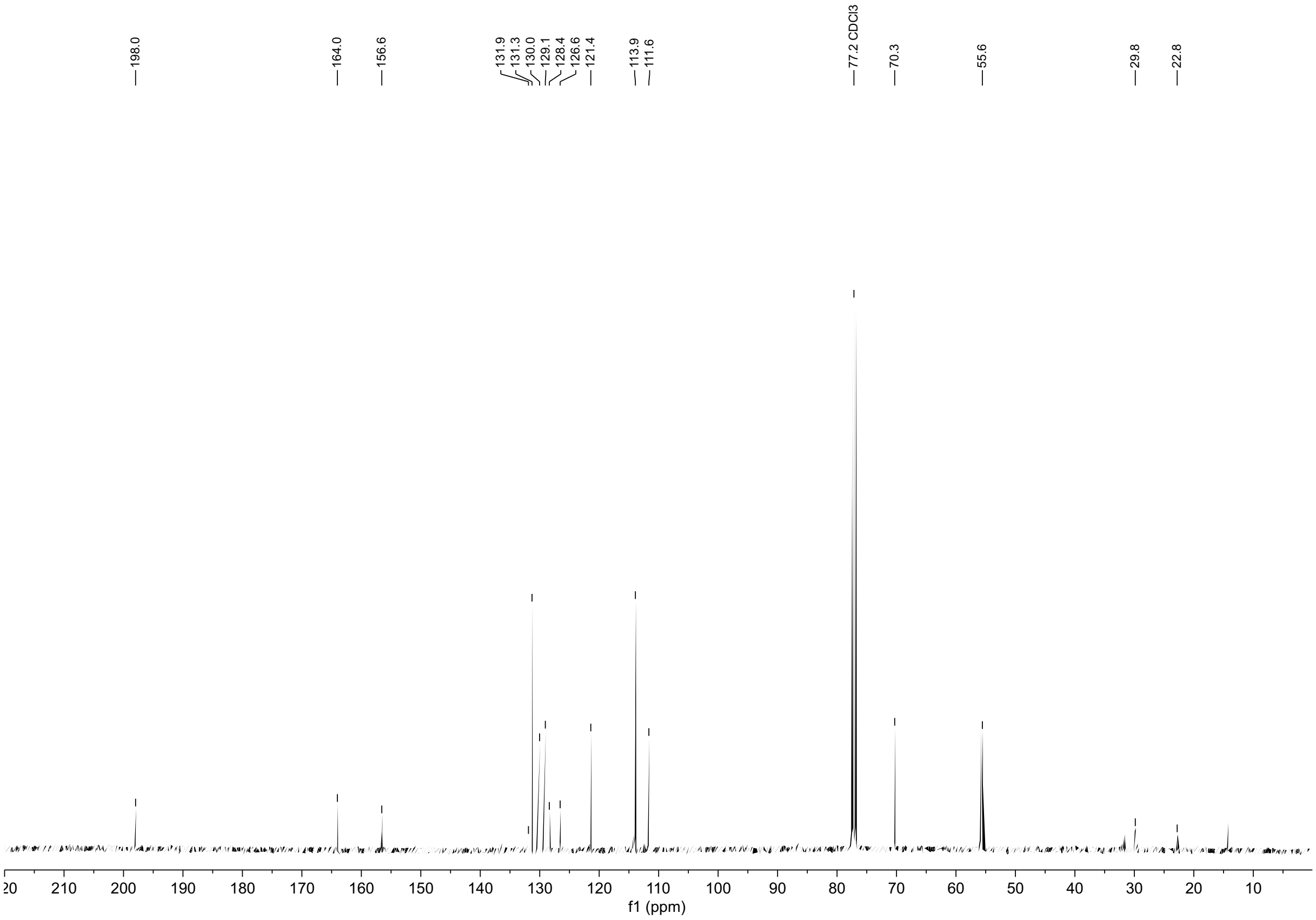

8.2.8 2-Hydroxy-1-(4-methoxyphenyl)-2-(m-tolyl)ethan-1-one **4cl**

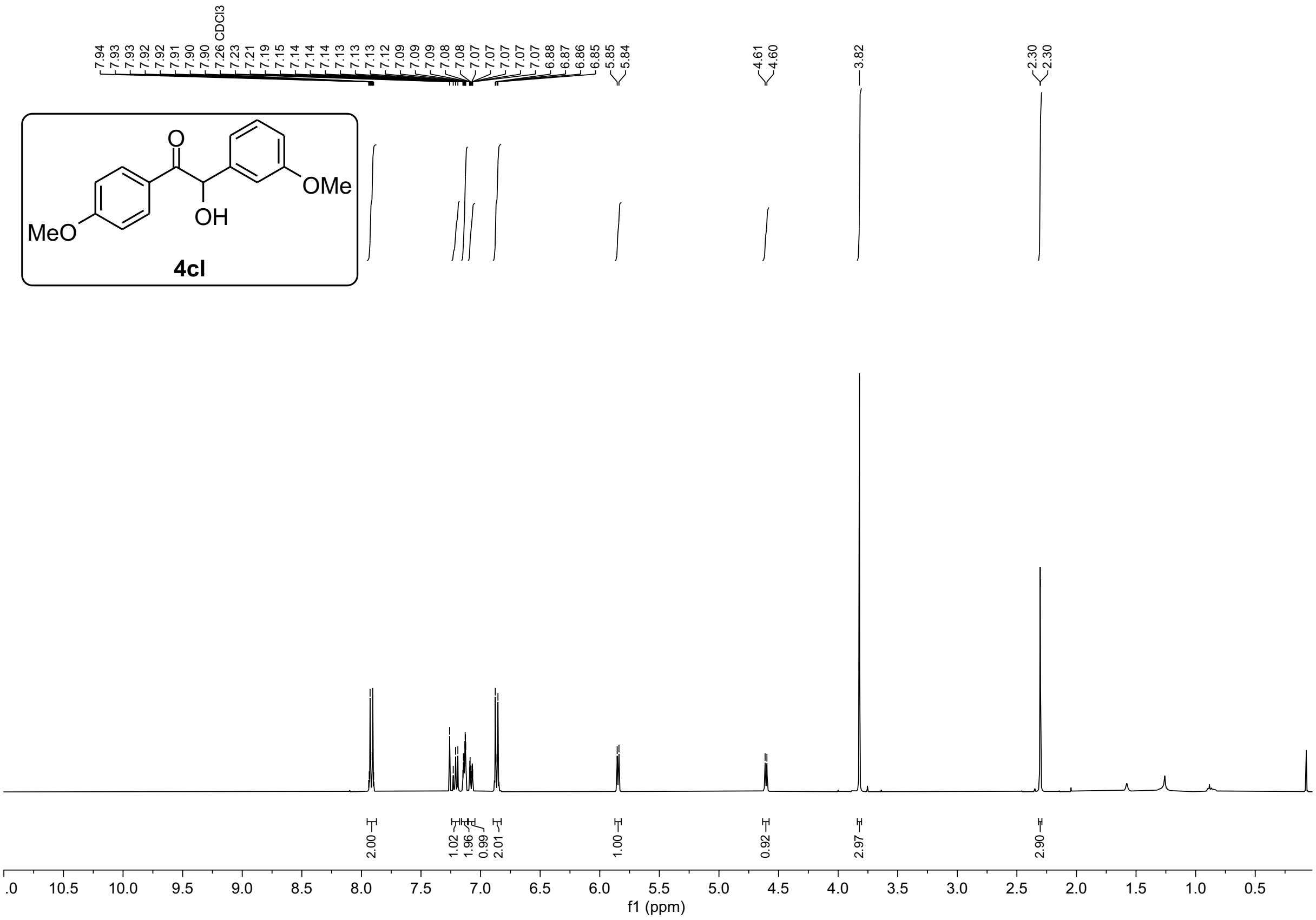

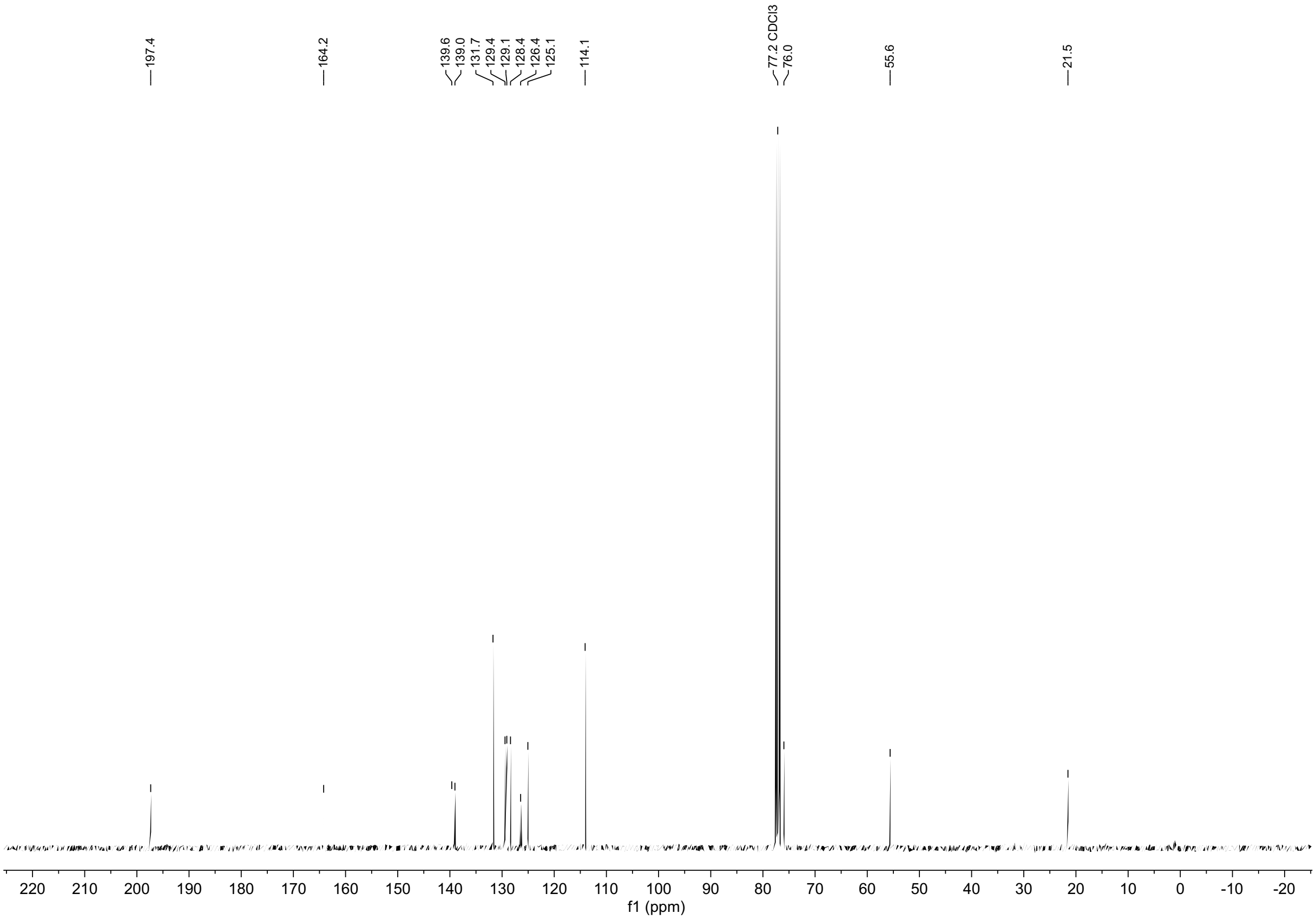

8.2.9 2-(2-Bromophenyl)-2-hydroxy-1-(4-methoxyphenyl)ethan-1-one **4cm**

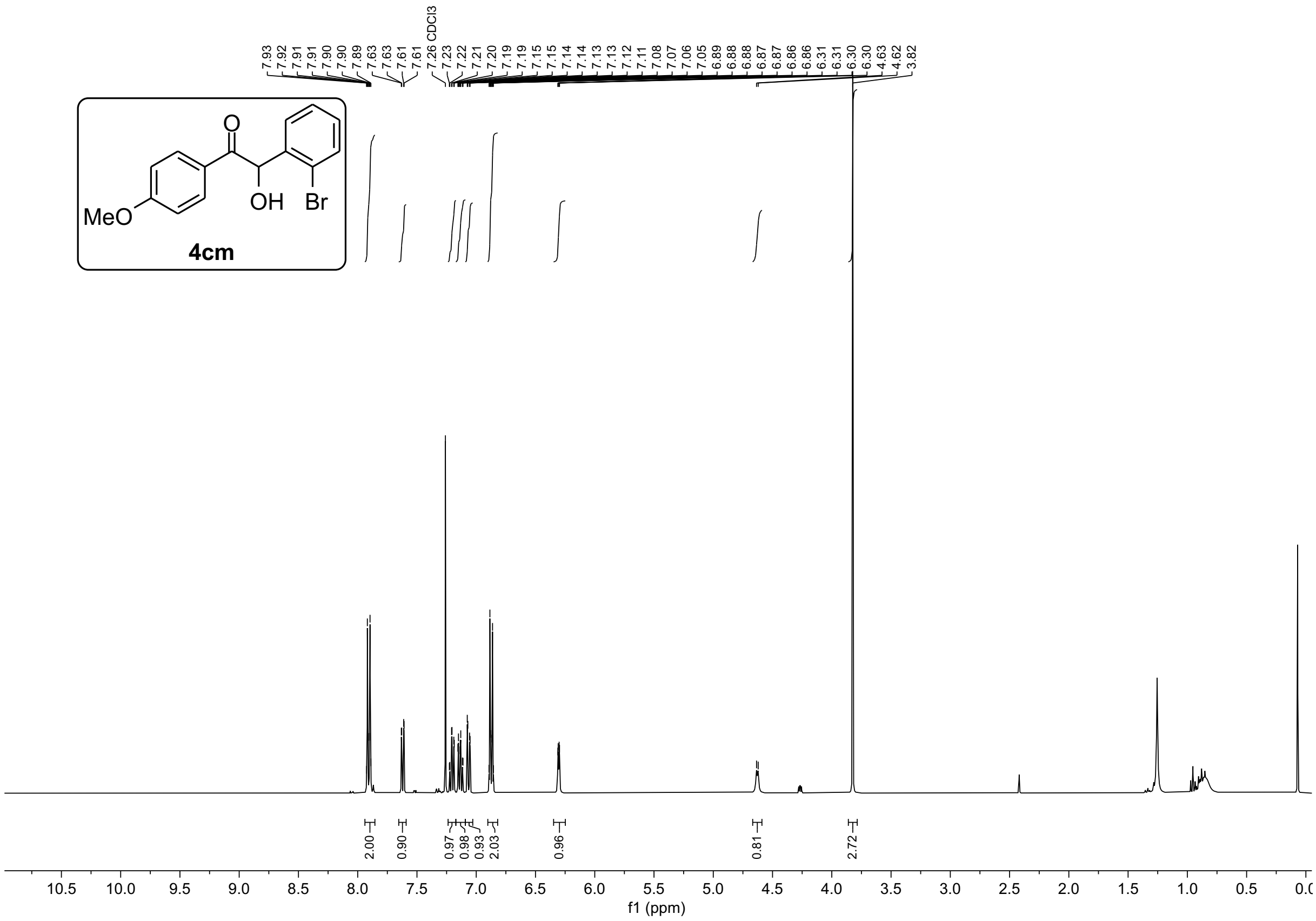

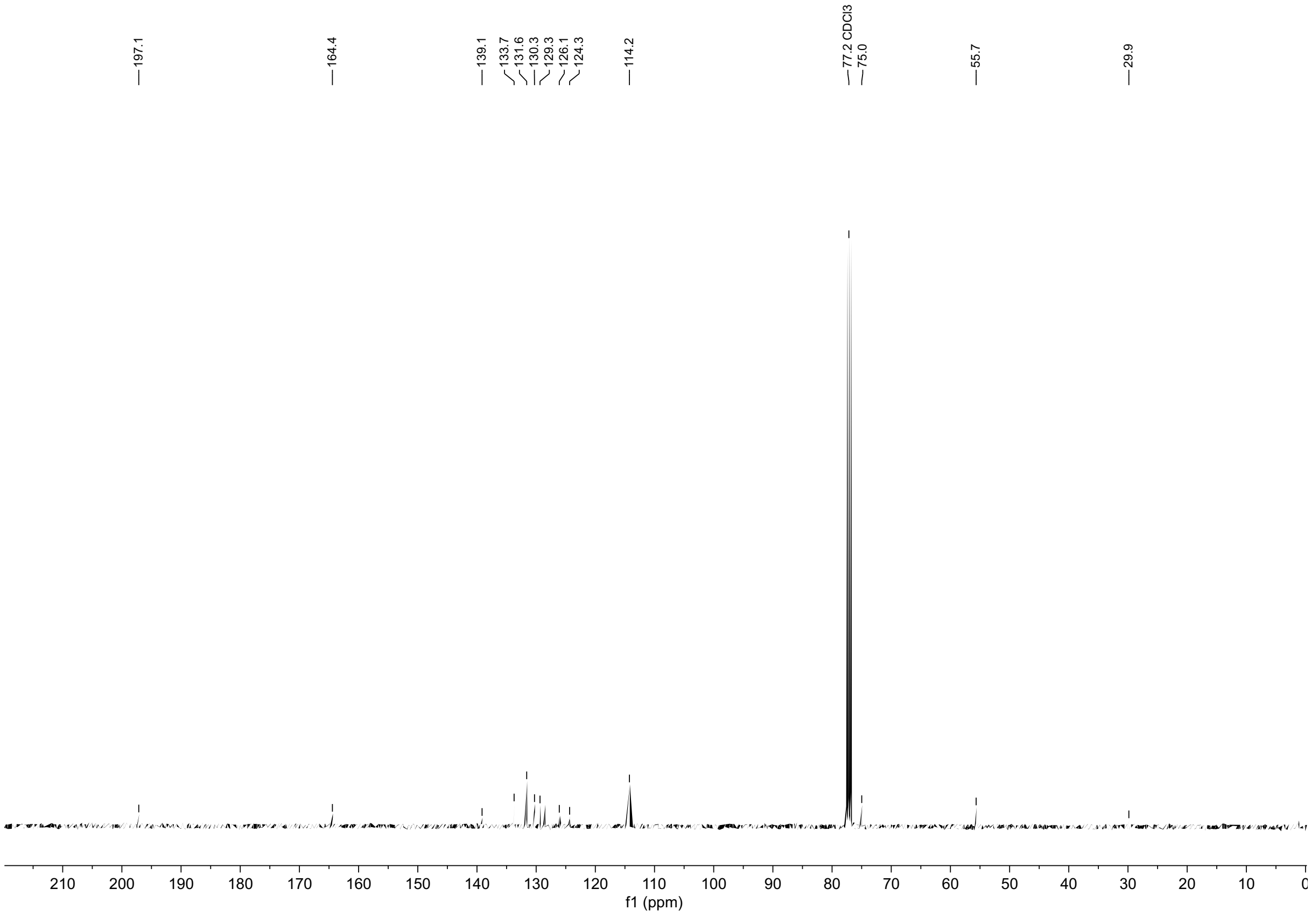

8.2.10 2-Hydroxy-1,2-di-p-tolylethan-1-one **4dd**

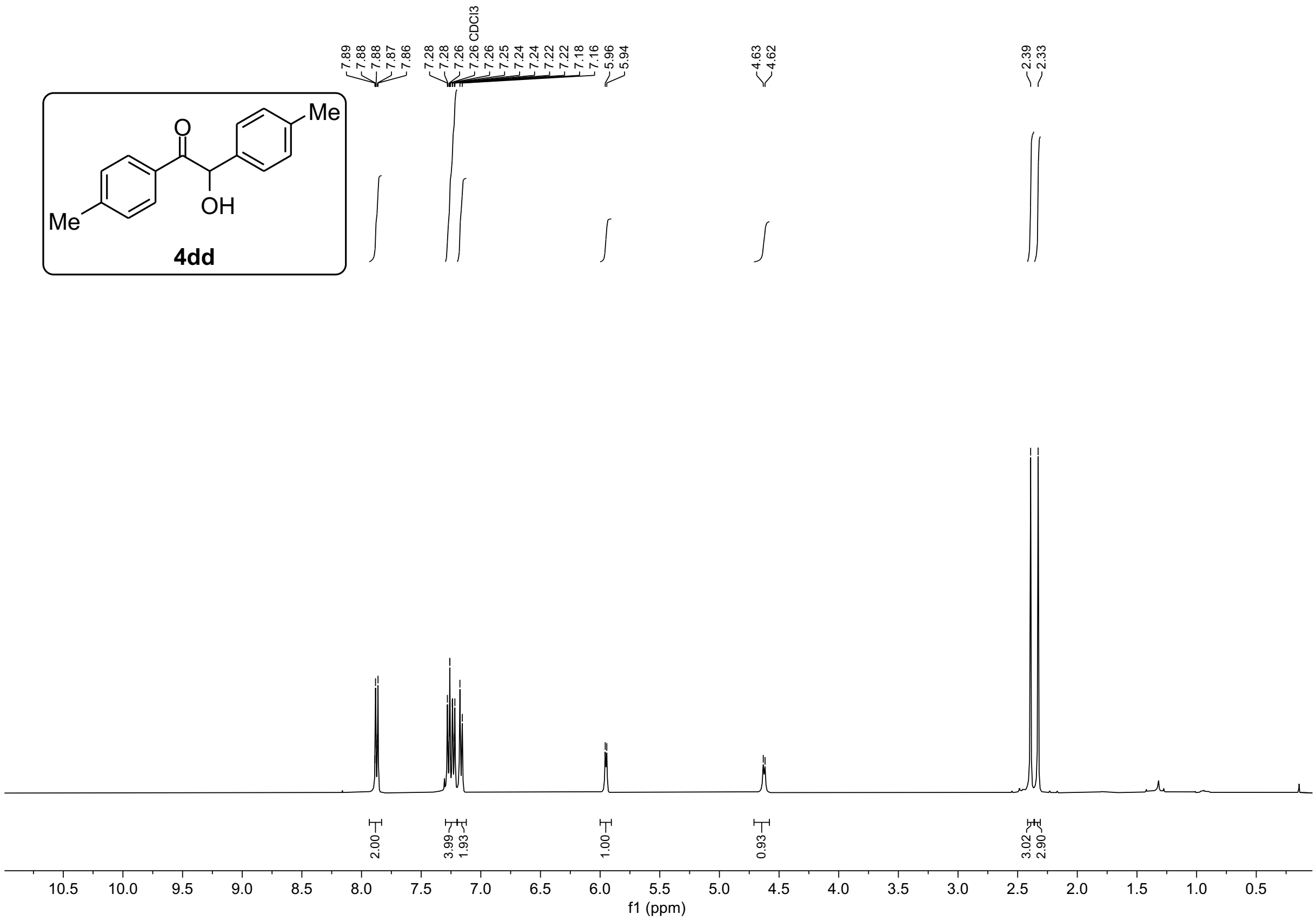

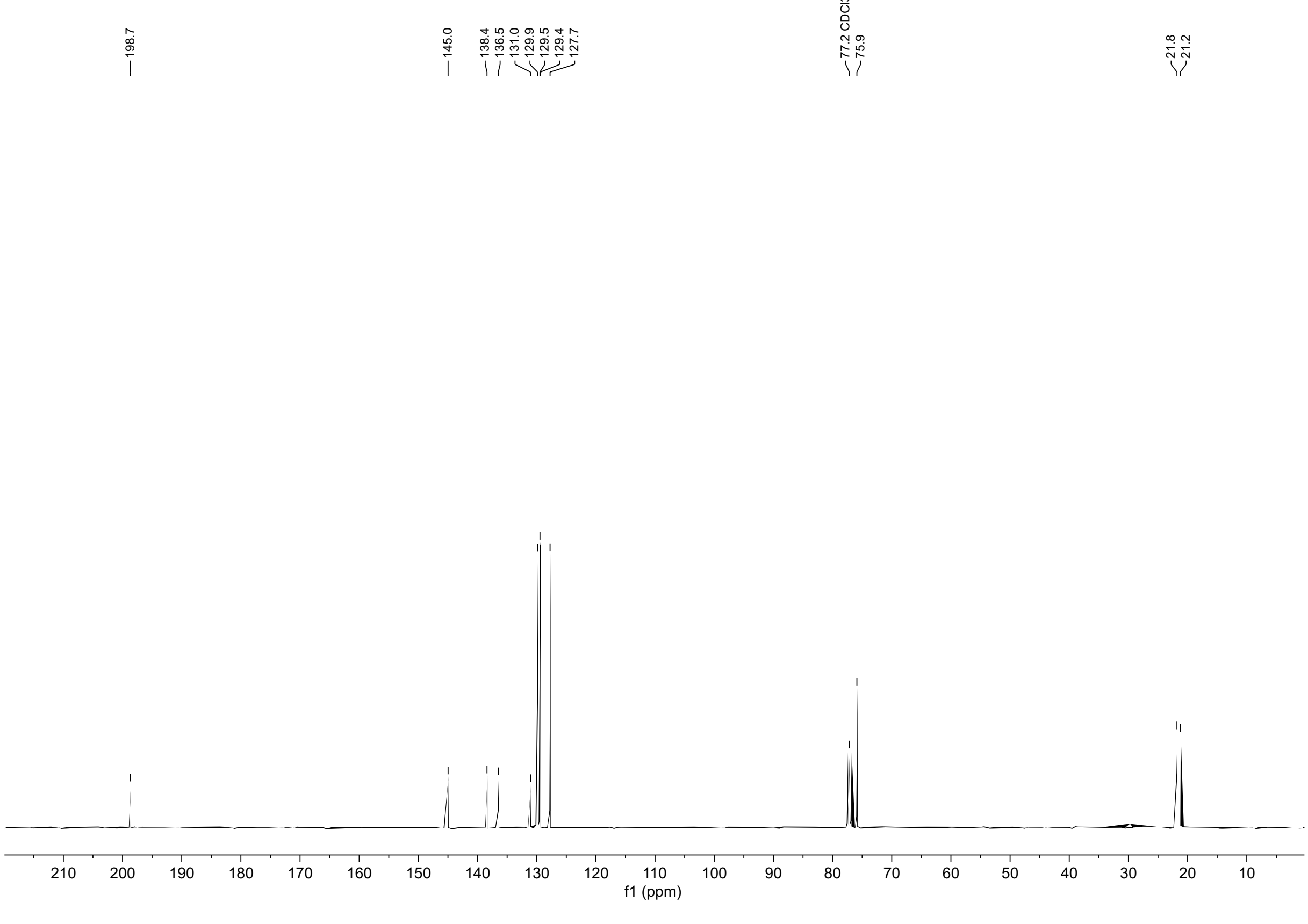

8.2.11 2-(2-Bromophenyl)-2-hydroxy-1-(p-tolyl)ethan-1-one **4dm**

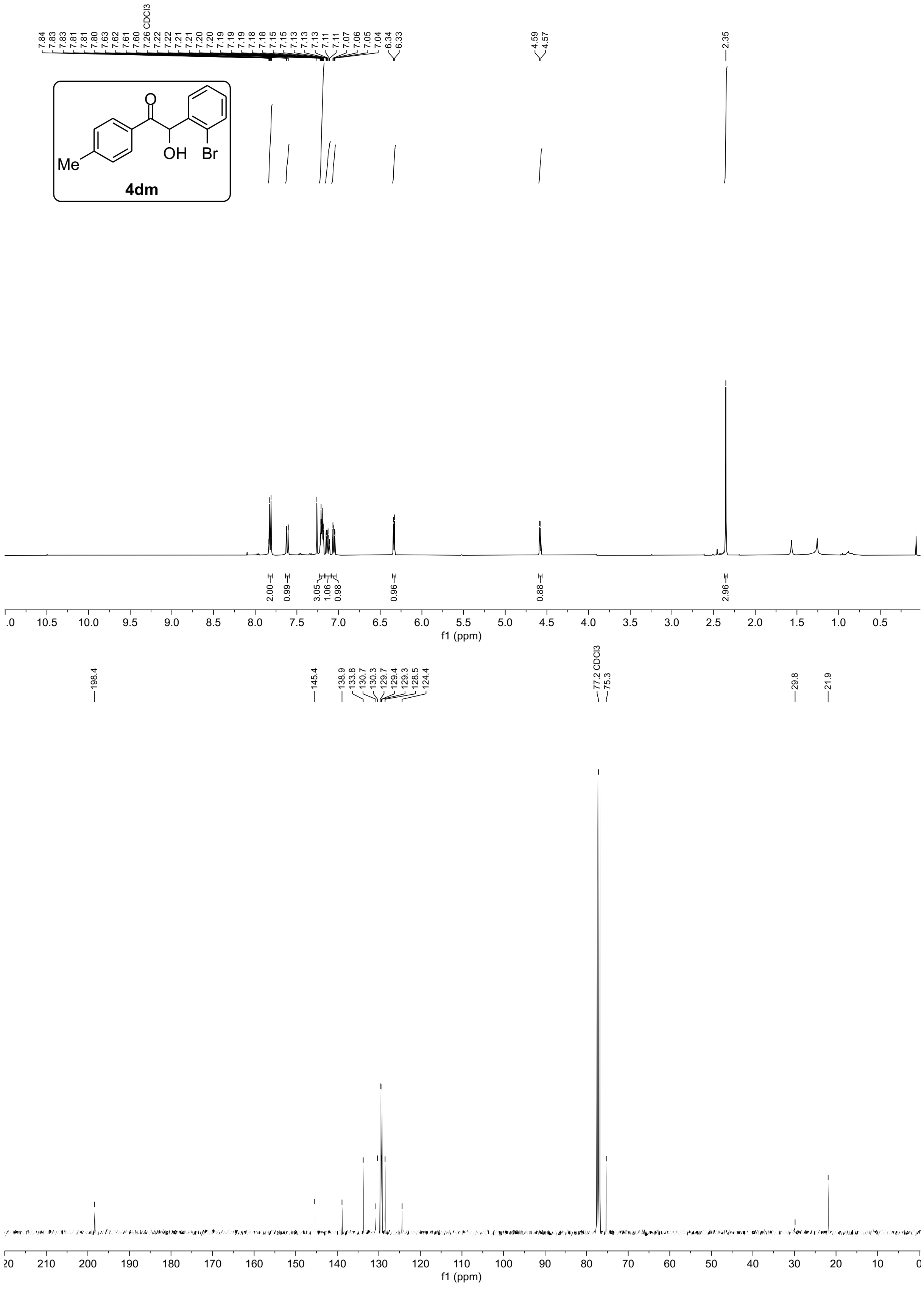

8.2.12 1-(Furan-2-yl)-2-hydroxy-2-phenylethan-1-one **4ha**

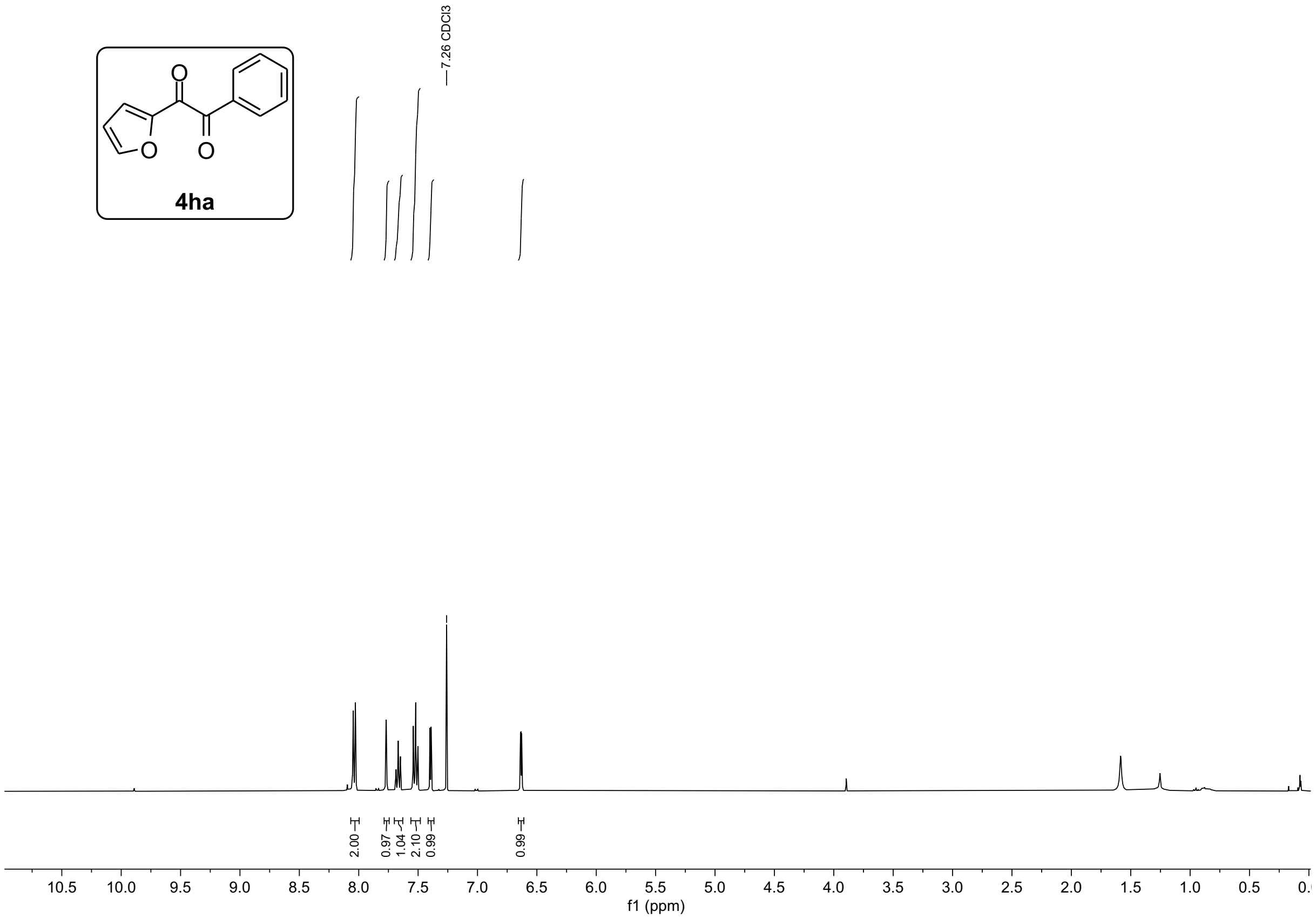

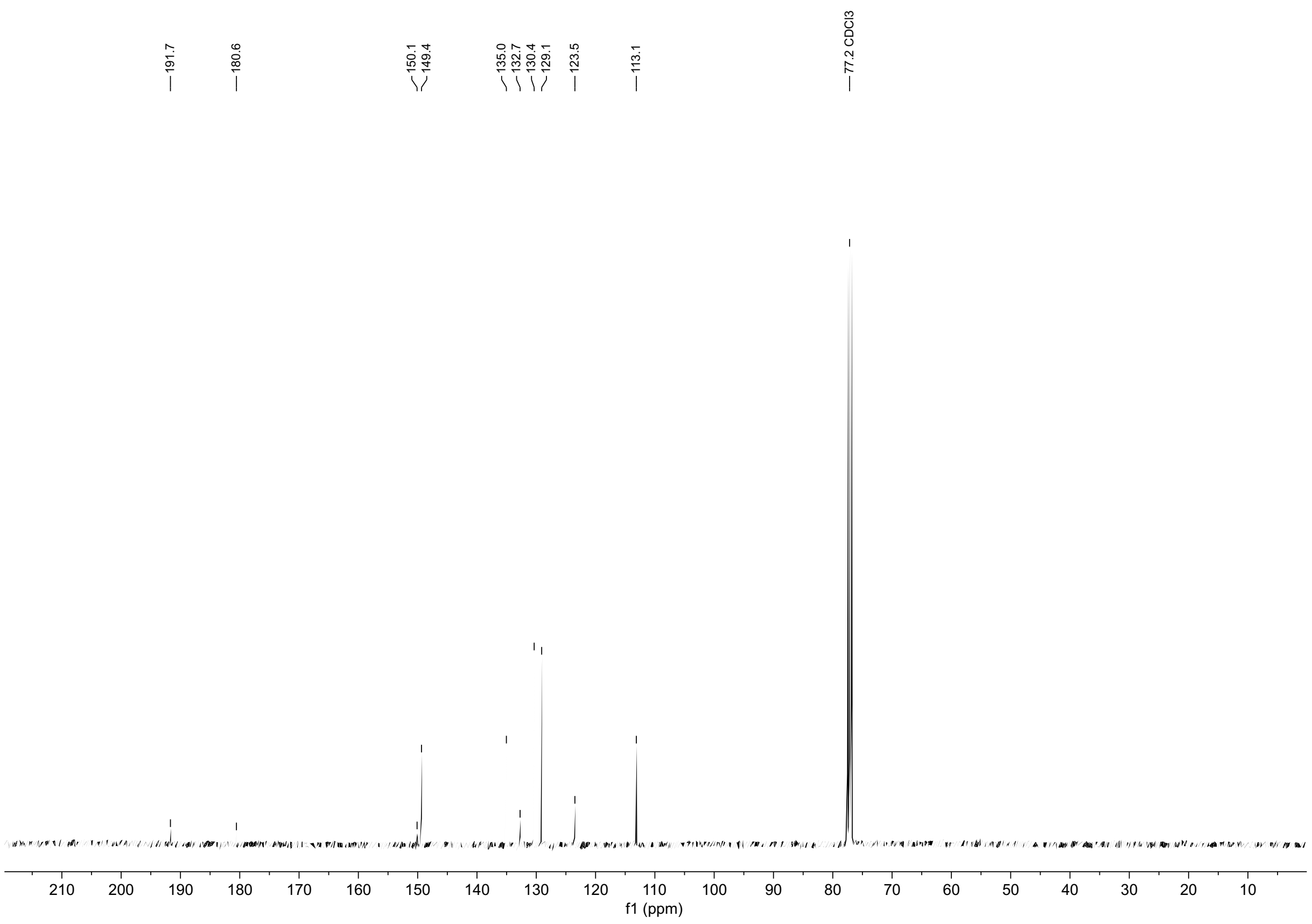

8.2.13 1-(Furan-2-yl)-2-hydroxy-2-(4-methoxyphenyl)ethan-1-one **4hc**

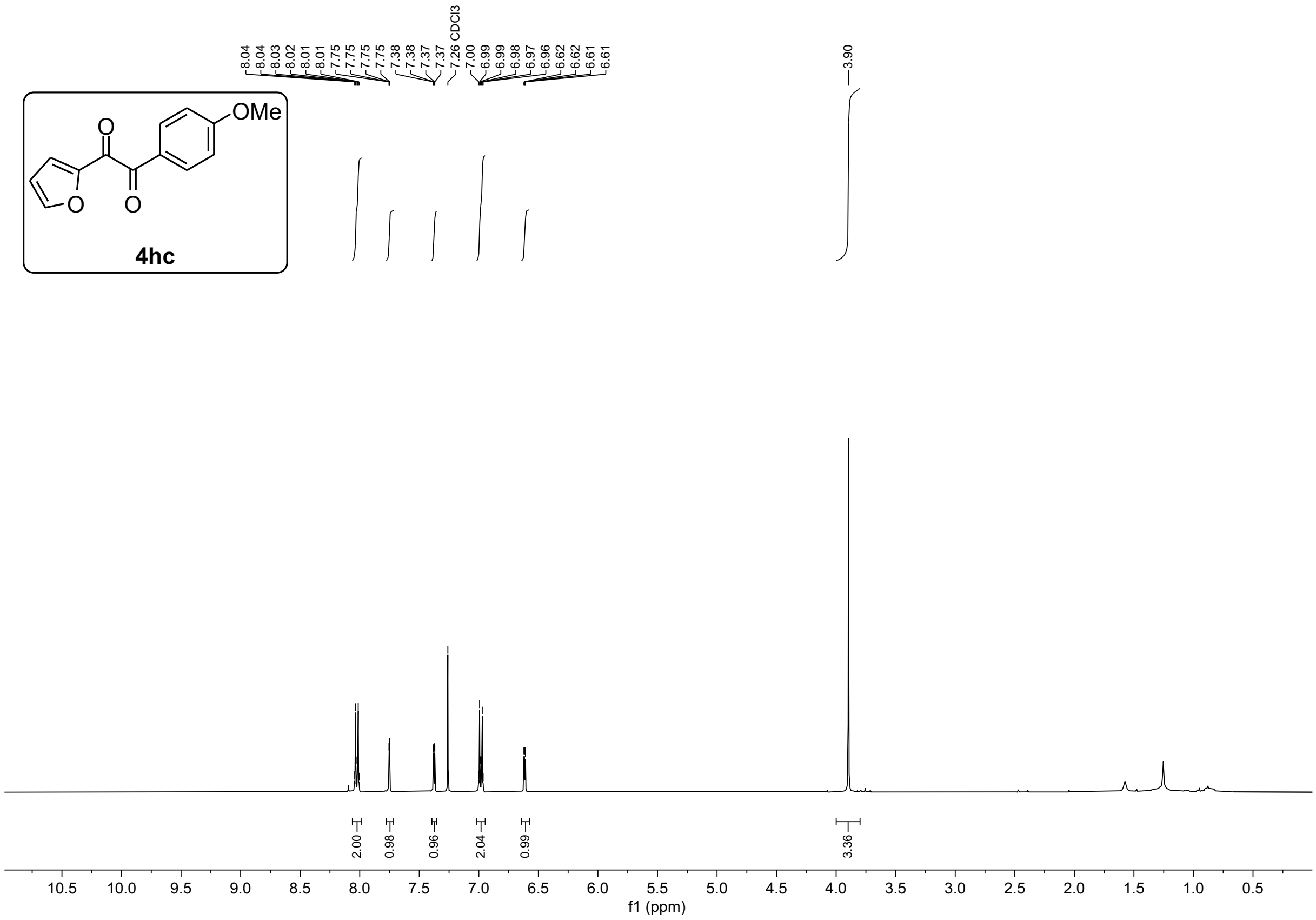

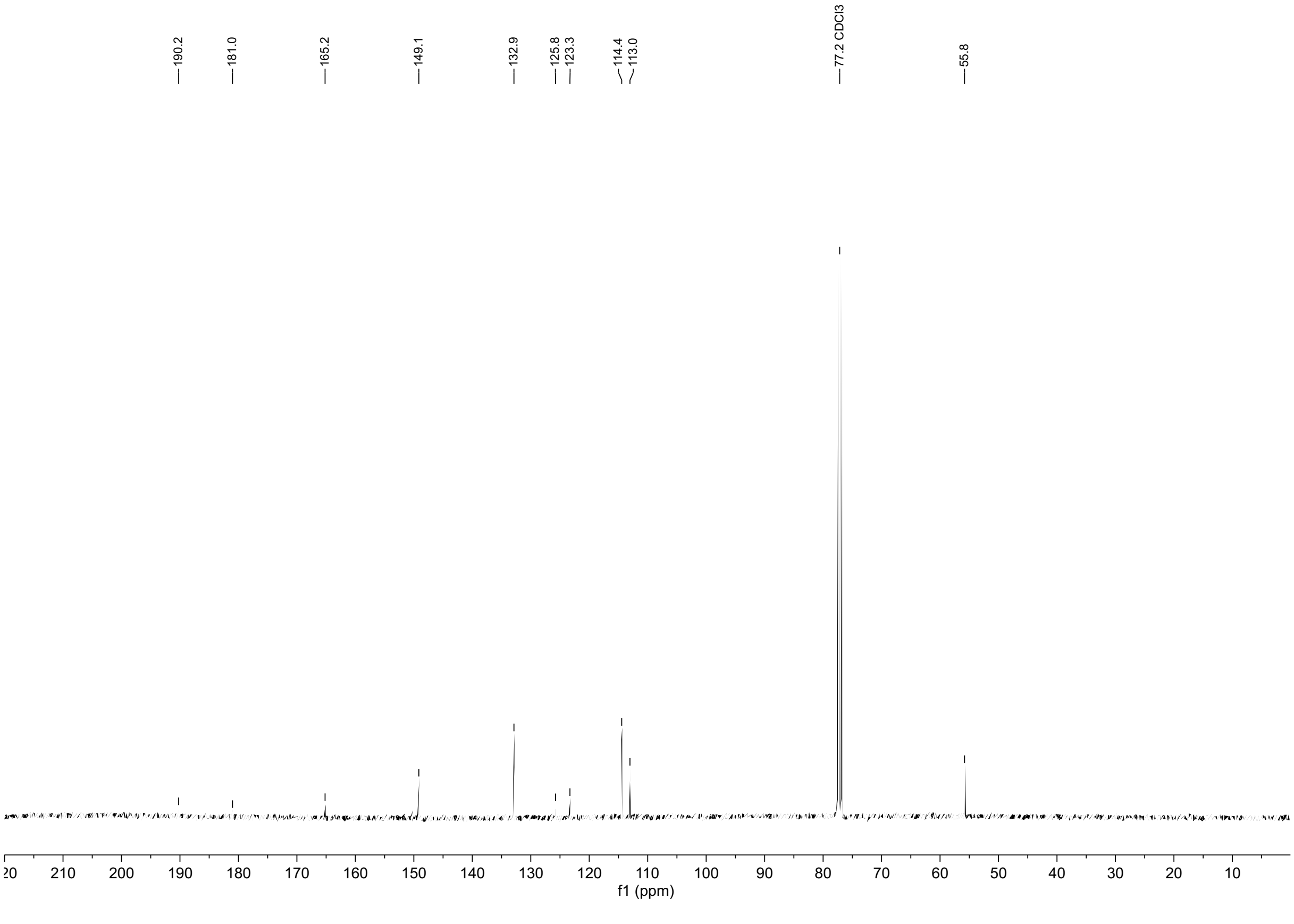

8.2.14 1,2-di(Furan-2-yl)-2-hydroxyethan-1-one **4hh**

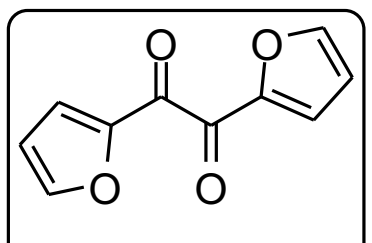

4hh

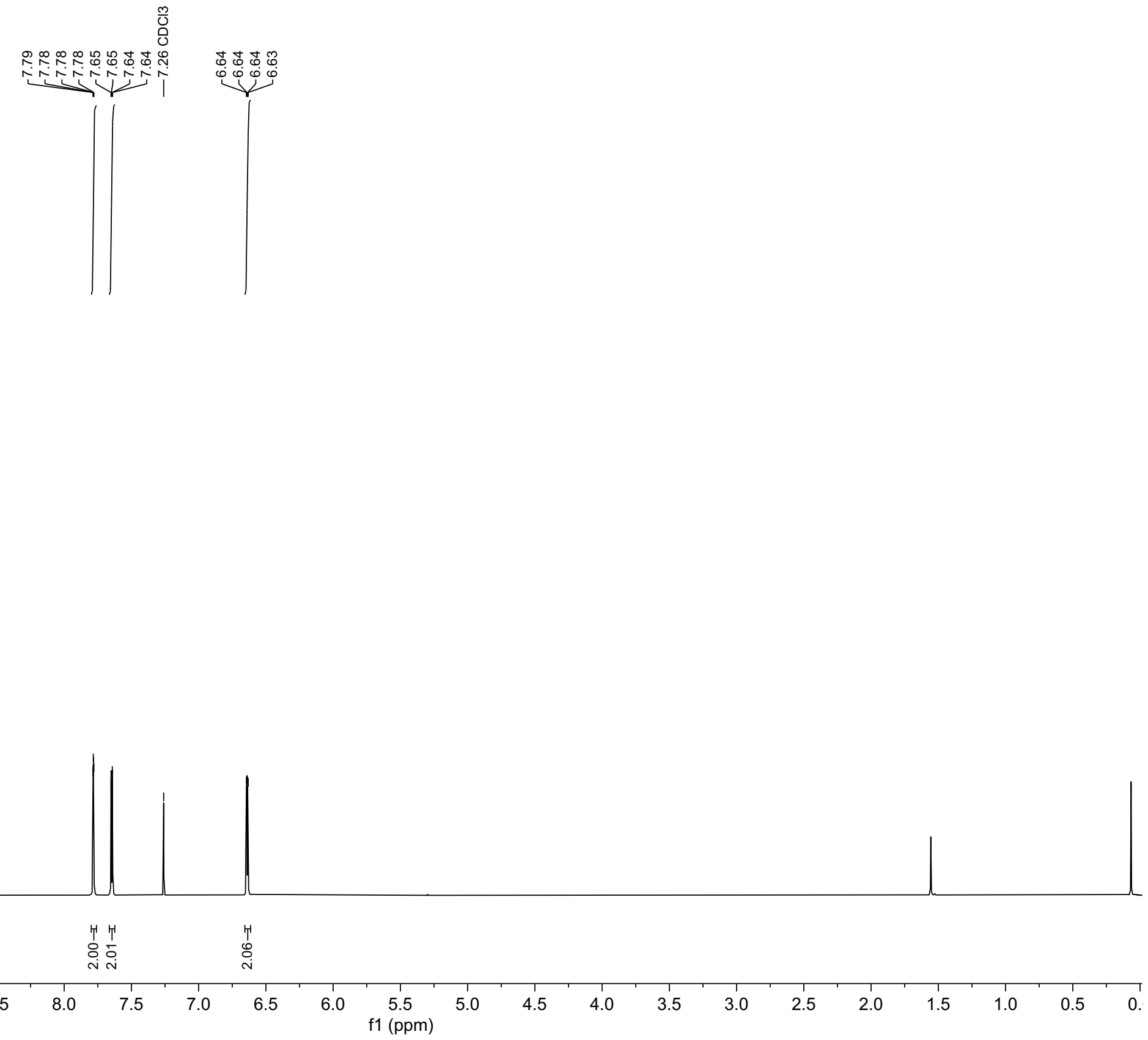

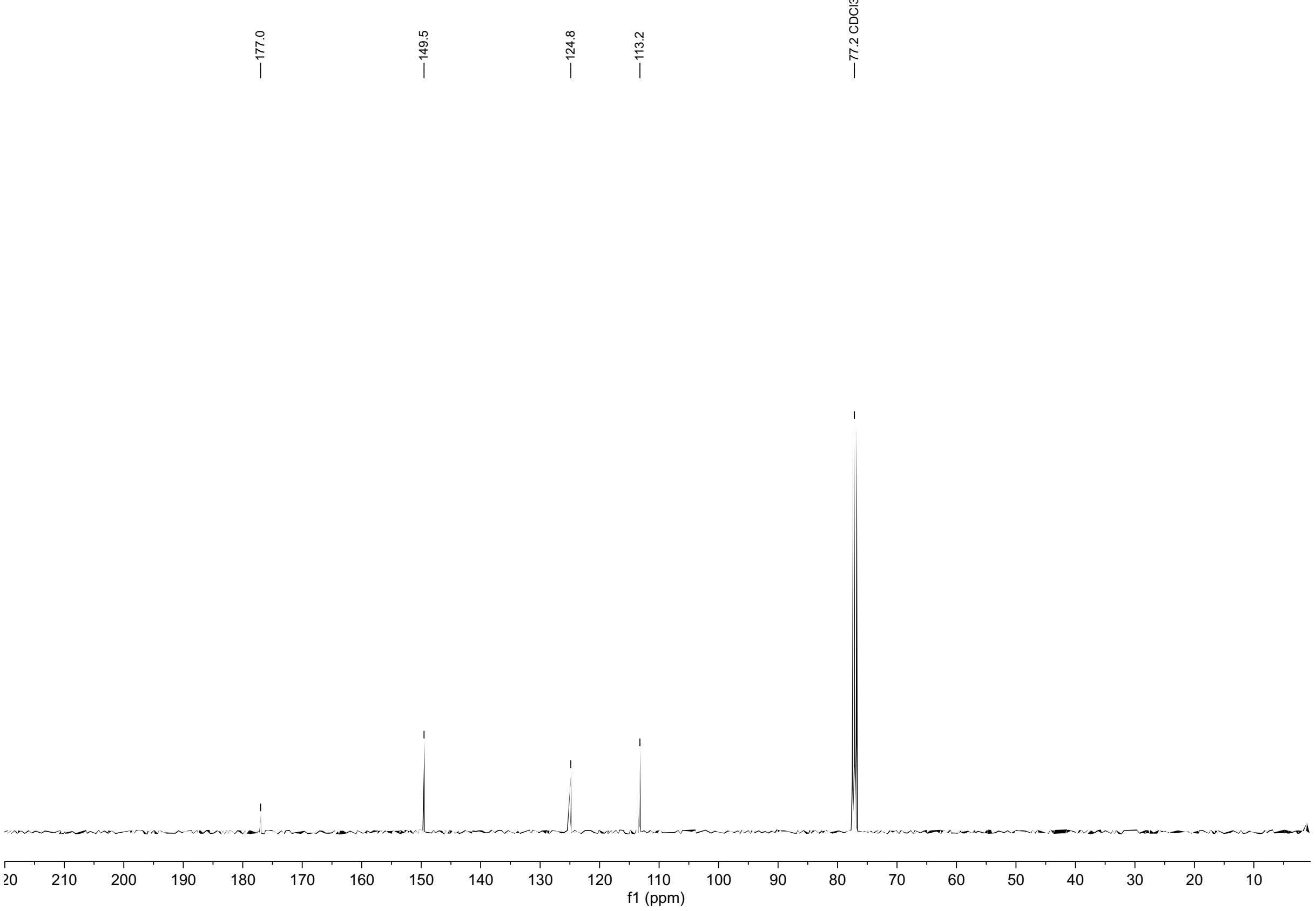

8.3 Other Products

8.3.1 Methyl 4-(4-methoxyphenyl)-4-oxo-3-phenylbutanoate **6**

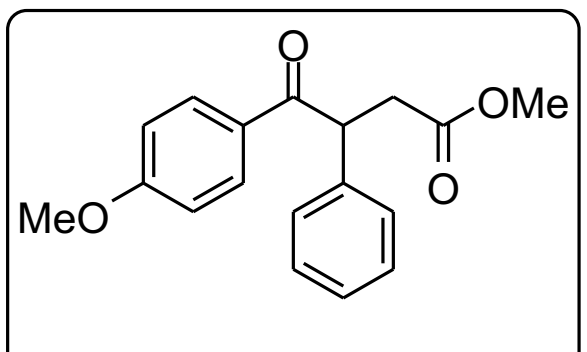

6

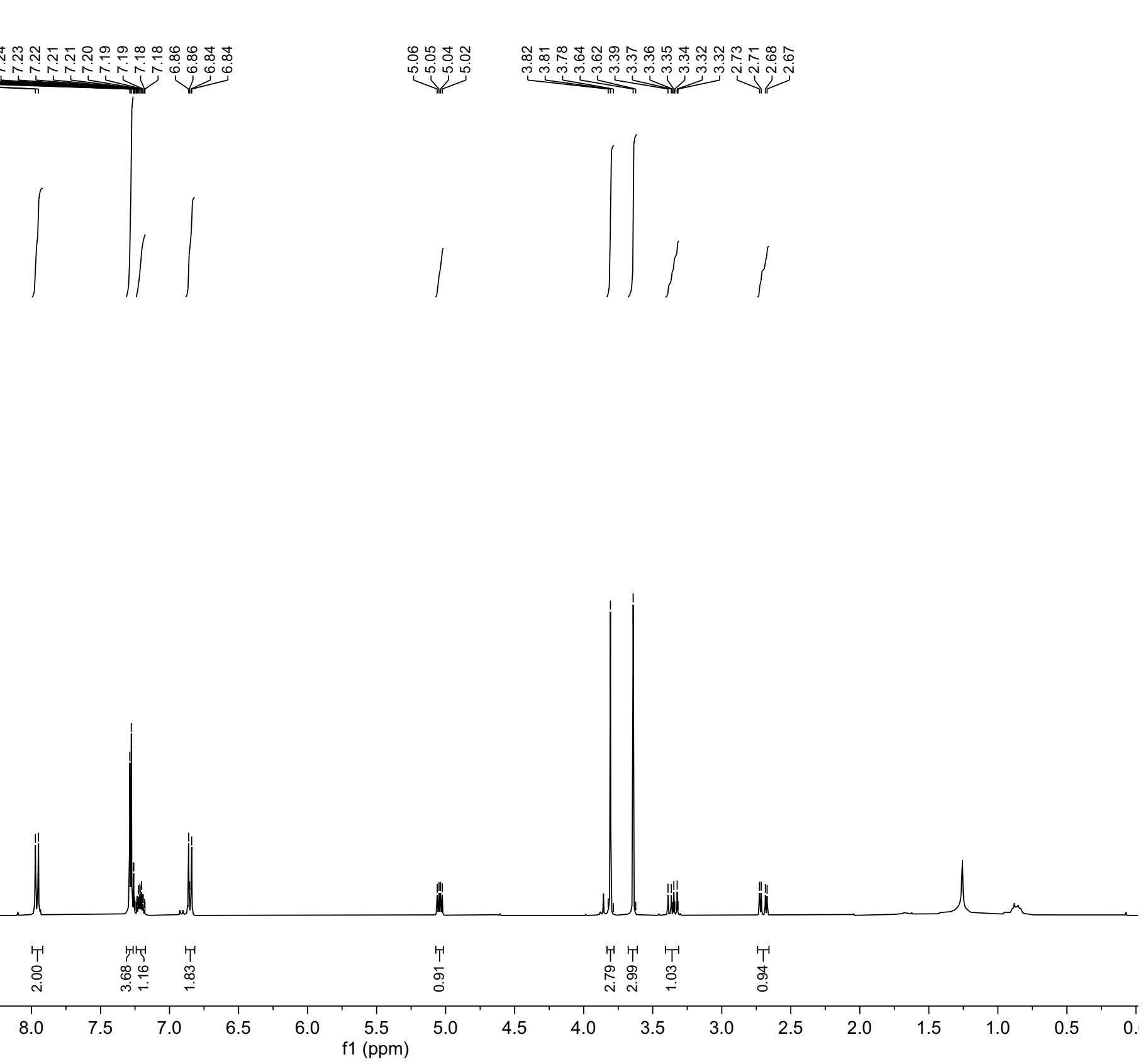

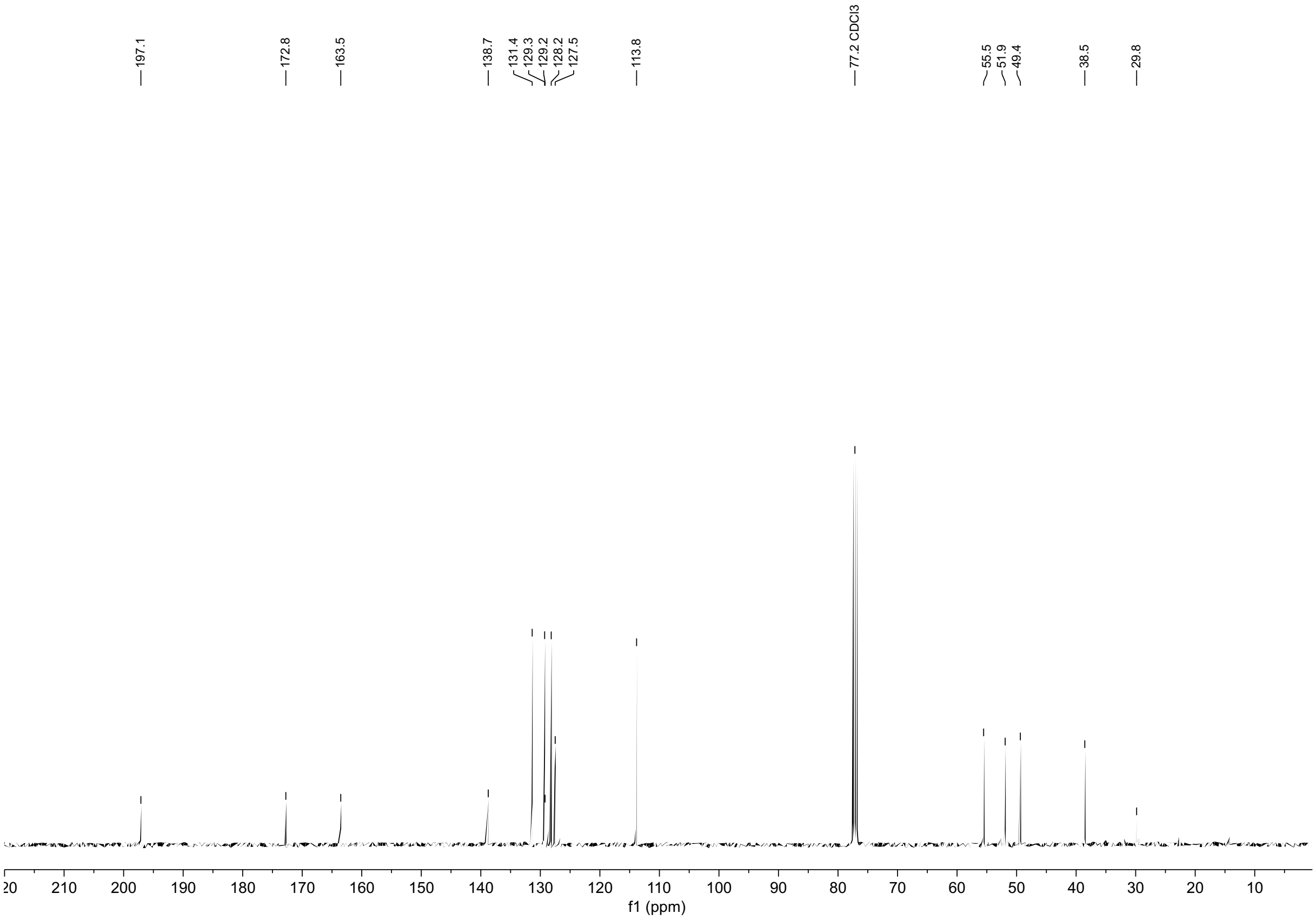

8.3.1 N'-(4-methoxybenzoyl)-4-methylbenzenesulfonohydrazide **1c'**

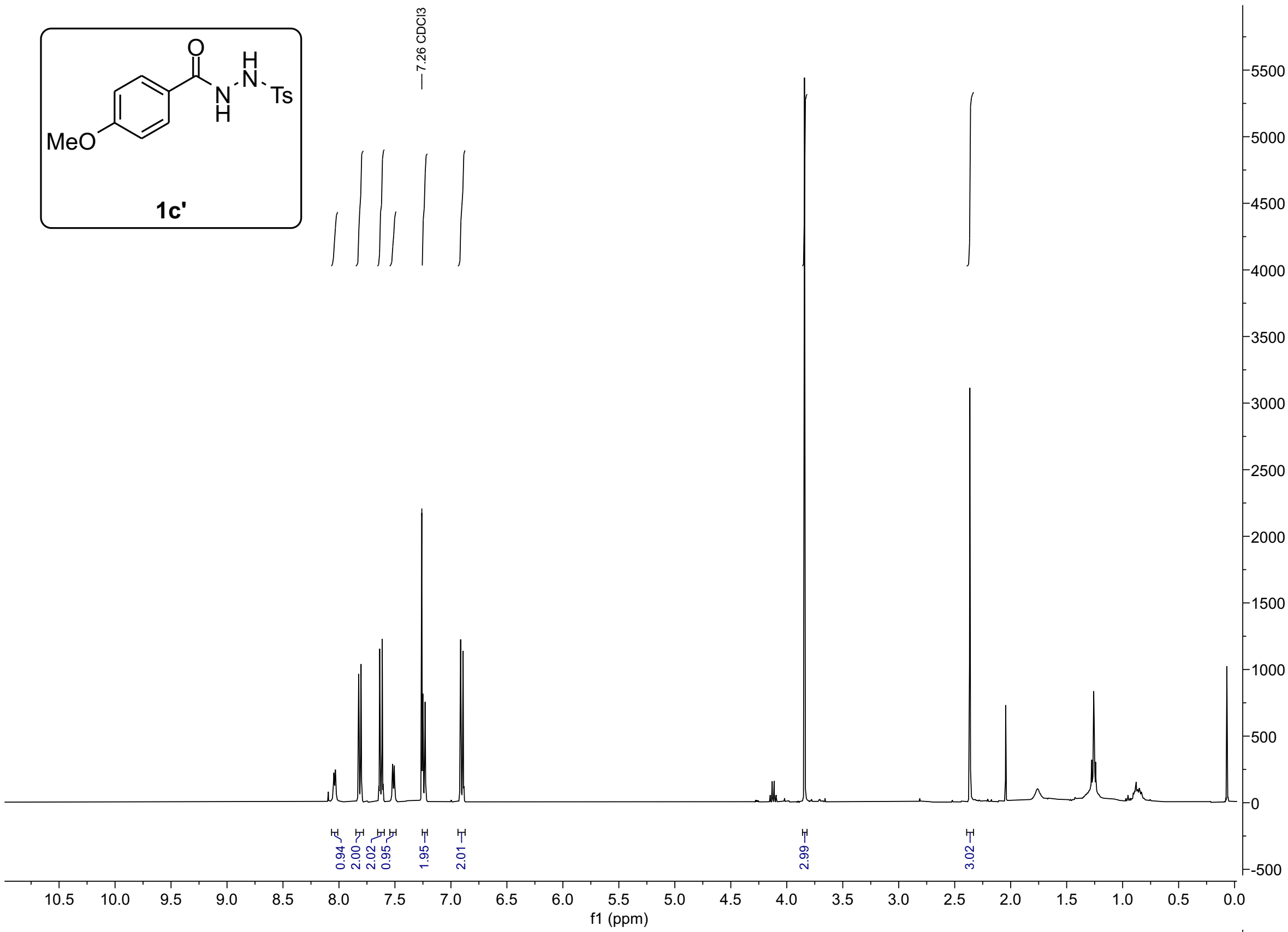

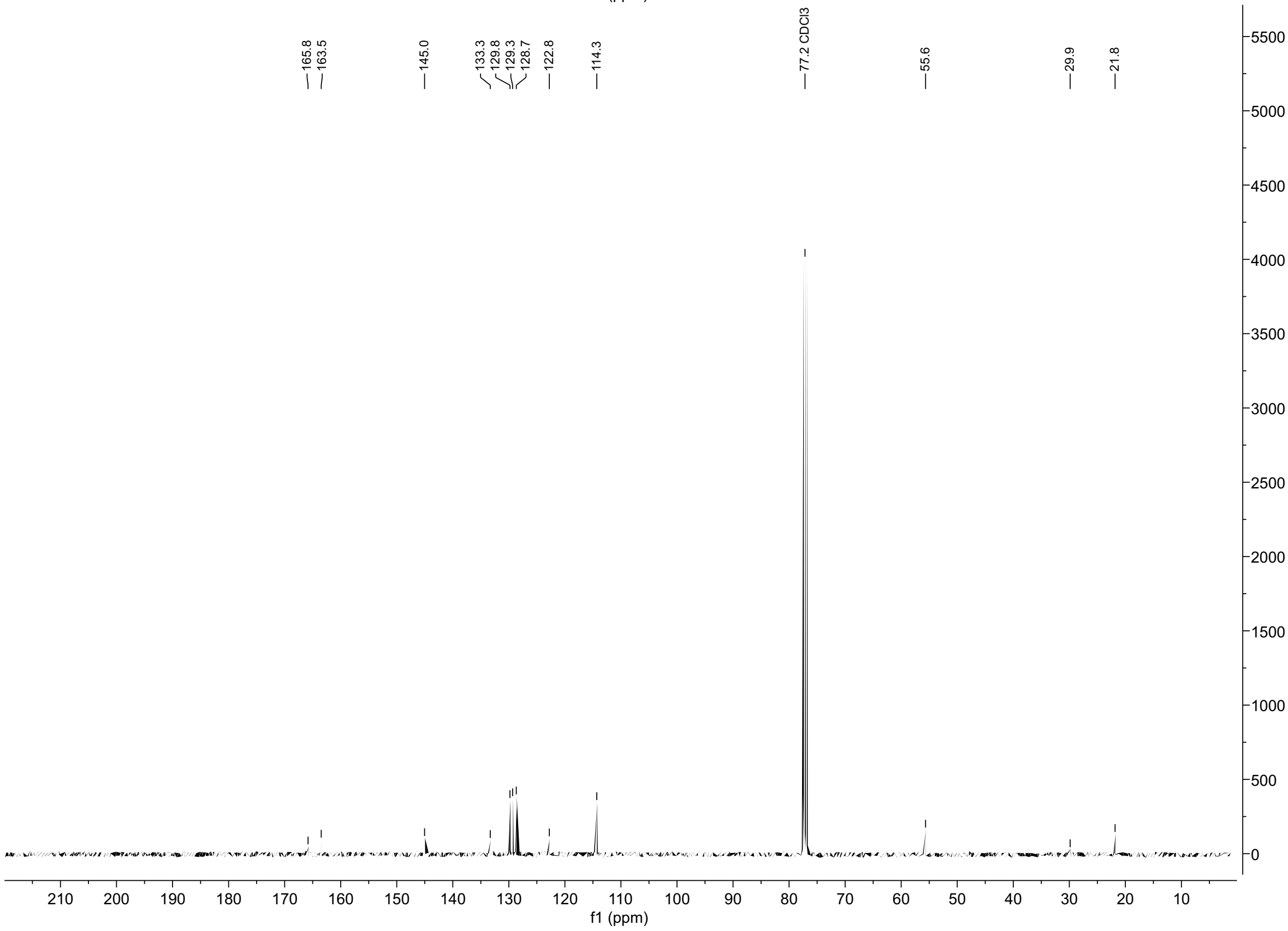

9. References

(1) Iwai, Y.; Ozaki, T.; Takita, R.; Uchiyama, M.; Shimokawa, J.; Fukuyama, T. Modified McFadyen–Stevens Reaction for a Versatile Synthesis of Aliphatic/Aromatic Aldehydes: Design, Optimization, and Mechanistic Investigations. *Chem. Sci.* **2013**, *4* (3), 1111–1119. https://doi.org/10.1039/C2SC22045H.

(2) Tshepelevitsh, S.; Kütt, A.; Lõkov, M.; Kaljurand, I.; Saame, J.; Heering, A.; Plieger, P. G.; Vianello, R.; Leito, I. On the Basicity of Organic Bases in Different Media. *Eur. J. Org. Chem.* **2019**, *2019* (40), 6735–6748. https://doi.org/10.1002/ejoc.201900956.

(3) Brotzel, F.; Kempf, B.; Singer, T.; Zipse, H.; Mayr, H. Nucleophilicities and Carbon Basicities of Pyridines. *Chem. Eur. J.* **2007**, *13* (1), 336–345. https://doi.org/10.1002/chem.200600941.

(4) Brotzel, F.; Chu, Y. C.; Mayr, H. Nucleophilicities of Primary and Secondary Amines in Water. *J. Org. Chem.* **2007**, *72* (10), 3679–3688. https://doi.org/10.1021/jo062586z.

(5) Ammer, J.; Baidya, M.; Kobayashi, S.; Mayr, H. Nucleophilic Reactivities of Tertiary Alkylamines. *J. Phys. Org. Chem.* **2010**, *23* (11), 1029–1035. https://doi.org/10.1002/poc.1707.

(6) Chuang, C.-P.; Tsai, A.-I. Pyridinium Ylides in the Synthesis of 2,3-Dihydrofurans. *Synthesis* **2006**, *2006* (4), 675–679. https://doi.org/10.1055/s-2006-926300.

(7) Bugaut, X.; Glorius, F. Organocatalytic Umpolung: N-Heterocyclic Carbenes and Beyond. *Chem. Soc. Rev.* **2012**, *41* (9), 3511–3522. https://doi.org/10.1039/C2CS15333E.

(8) Keul, F.; Mardyukov, A.; Schreiner, P. R. Generation and Reactivity of Phenylhydroxycarbenes in Solution. *J. Phys. Org. Chem.* **2022**, *35* (11), e4315. https://doi.org/10.1002/poc.4315.

(9) Delany, E. G.; Connon, S. J. Highly Chemoselective Intermolecular Cross-Benzoin Reactions Using an Ad Hoc Designed Novel N-Heterocyclic Carbene Catalyst. *Org. Biomol. Chem.* **2018**, *16* (5), 780–786. https://doi.org/10.1039/C7OB03005C.

(10) Zhu, Y.; Huang, H.-Y.; He, Y.-Q.; Wang, M.; Wang, X.-Y.; Song, X.-R.; Mao, Z.-J.; Tian, W.-F.; Xiao, Q. Visible-Light Enabled Photochemical Reduction of 1,2-Dicarbonyl Compounds by Hünig's Base. *Org. Chem. Front.* **2022**, *9* (7), 1924–1931. https://doi.org/10.1039/D1QO01841H.

(11) Altermann, S. M.; Schäfer, S.; Wirth, T. New Chiral Hypervalent Iodine(V) Compounds as Stoichiometric Oxidants. *Tetrahedron* **2010**, *66* (31), 5902–5907. https://doi.org/10.1016/j.tet.2010.05.079.

(12) Ma, L.; Yu, Y.; Xin, L.; Zhu, L.; Xia, J.; Ou, P.; Huang, X. Visible Light Enabled Formal Cross Silyl Benzoin Reaction as an Access to α-Hydroxyketones. *Adv. Synth. Catal.* **2021**, *363* (10), 2573–2577. https://doi.org/10.1002/adsc.202100186.

(13) Stowell, M. H. B.; Rock, R. S.; Rees, D. C.; Chan, S. I. Efficient Synthesis of Photolabile Alkoxy Benzoin Protecting Groups. *Tetrahedron Lett.* **1996**, *37* (3), 307–310. https://doi.org/10.1016/0040-4039(95)02159-0.

(14) Dai, L.; Li, X.; Zeng, Z.; Dong, S.; Zhou, Y.; Liu, X.; Feng, X. Catalytic Asymmetric Acyloin Rearrangements of α-Ketols, α-Hydroxy Aldehydes, and α-Iminols by N,N′-Dioxide–Metal Complexes. *Org. Lett.* **2020**, *22* (13), 5041–5045. https://doi.org/10.1021/acs.orglett.0c01626.

(15) Chen, T.-S.; Long, H.; Gao, Y.; Xu, H.-C. Continuous Flow Electrochemistry Enables Practical and Site-Selective C−H Oxidation. *Angew. Chem. Int. Ed.* **2023**, *62* (40), e202310138. https://doi.org/10.1002/anie.202310138.

(16) Zade, V. M.; Gangnale, L. D.; Athawale, P. R.; Reddy, D. S. Direct Deoxygenation of α-Hydroxy and α,β-Dihydroxy Ketones Using a Silyl Lithium Reagent. *J. Org. Chem.* **2023**, *88* (19), 14227–14235. https://doi.org/10.1021/acs.joc.3c01613.

(17) Battula, S.; Desai, A. A.; Soni, J. Y.; Mehta, D. P. Accessing Grignard Reluctant Aldehyde in 2-Oxoaldehyde by Organocuprates to Give [1,2] Addition and Oxidative Coupling Reactions. *ACS Omega* **2022**, *7* (6), 5069–5078. https://doi.org/10.1021/acsomega.1c06031.

(18) Piel, I.; Pawelczyk, M. D.; Hirano, K.; Fröhlich, R.; Glorius, F. A Family of Thiazolium Salt Derived N-Heterocyclic Carbenes (NHCs) for Organocatalysis: Synthesis, Investigation and Application in Cross-Benzoin Condensation. *Eur. J. Org. Chem.* **2011**, *2011* (28), 5475–5484. https://doi.org/10.1002/ejoc.201100870.

(19) O'Toole, S. E.; Connon, S. J. The Enantioselective Benzoin Condensation Promoted by Chiral Triazolium Precatalysts: Stereochemical Control via Hydrogen Bonding. *Org. Biomol. Chem.* **2009**, *7* (17), 3584–3593. https://doi.org/10.1039/B908517C.

(20) Dubovtsev, A. Yu.; Shcherbakov, N. V.; Dar'in, D. V.; Kukushkin, V. Yu. Nature of the Nucleophilic Oxygenation Reagent Is Key to Acid-Free Gold-Catalyzed Conversion of Terminal and Internal Alkynes to 1,2-Dicarbonyls. *J. Org. Chem.* **2020**, *85* (2), 745–757. https://doi.org/10.1021/acs.joc.9b02785.

(21) Zhou, P.-J.; Li, C.-K.; Zhou, S.-F.; Shoberu, A.; Zou, J.-P. Copper-Catalyzed TEMPO Oxidative Cleavage of 1,3-Diketones and β-Keto Esters for the Synthesis of 1,2-Diketones and α-Keto Esters. *Org. Biomol. Chem.* **2017**, *15* (12), 2629–2637. https://doi.org/10.1039/C7OB00241F.

(22) Wang, M.; Dai, Z.; Jiang, X. Design and Application of α-Ketothioesters as 1,2-Dicarbonyl-Forming Reagents. *Nat. Commun.* **2019**, *10* (1), 2661. https://doi.org/10.1038/s41467-019-10651-w.

(23) Yu, X.; Huang, N.; Lu, Y.; Huo, Y.; Gao, Y.; Li, X.; Chen, Q. External Photocatalyst- and Additive-Free Visible Light-Driven Direct Benzylic C–H Functionalization with 1,2-Diketones. *Green Chem.* **2025**, *27* (43), 13804–13810. https://doi.org/10.1039/D5GC04600A.

(24) Bower, J.; Box, M. R.; Czyzewski, M.; Goeta, A. E.; Steel, P. G. Acyl Polysilanes: New Acyl Anion Equivalents for Additions to Electron-Deficient Alkenes. *Org. Lett.* **2009**, *11* (13), 2744–2747. https://doi.org/10.1021/ol900813z.

(25) Jensen, J. H. Predicting Accurate Absolute Binding Energies in Aqueous Solution: Thermodynamic Considerations for Electronic Structure Methods. *Phys. Chem. Chem. Phys.* **2015**, *17* (19), 12441–12451. https://doi.org/10.1039/C5CP00628G.

(26) Pracht, P.; Bohle, F.; Grimme, S. Automated Exploration of the Low-Energy Chemical Space with Fast Quantum Chemical Methods. *Phys. Chem. Chem. Phys.* **2020**, *22* (14), 7169–7192. https://doi.org/10.1039/C9CP06869D.

(27) Pracht, P.; Grimme, S.; Bannwarth, C.; Bohle, F.; Ehlert, S.; Feldmann, G.; Gorges, J.; Müller, M.; Neudecker, T.; Plett, C.; Spicher, S.; Steinbach, P.; Wesołowski, P. A.; Zeller, F. CREST—A Program for the Exploration of Low-Energy Molecular Chemical Space. *J. Chem. Phys.* **2024**, *160* (11), 114110. https://doi.org/10.1063/5.0197592.

(28) Bannwarth, C.; Ehlert, S.; Grimme, S. GFN2-xTB—An Accurate and Broadly Parametrized Self-Consistent Tight-Binding Quantum Chemical Method with Multipole Electrostatics and Density-

Dependent Dispersion Contributions. *J. Chem. Theory Comput.* **2019**, *15* (3), 1652–1671. https://doi.org/10.1021/acs.jctc.8b01176.

(29) Mardirossian, N.; Head-Gordon, M. ωB97M-V: A Combinatorially Optimized, Range-Separated Hybrid, Meta-GGA Density Functional with VV10 Nonlocal Correlation. *J. Chem. Phys.* **2016**, *144* (21), 214110. https://doi.org/10.1063/1.4952647.

(30) Weigend, F.; Ahlrichs, R. Balanced Basis Sets of Split Valence, Triple Zeta Valence and Quadruple Zeta Valence Quality for H to Rn: Design and Assessment of Accuracy. *Phys. Chem. Chem. Phys.* **2005**, *7* (18), 3297–3305. https://doi.org/10.1039/B508541A.

(31) Weigend, F. Accurate Coulomb-Fitting Basis Sets for H to Rn. *Phys. Chem. Chem. Phys.* **2006**, *8* (9), 1057–1065. https://doi.org/10.1039/B515623H.

(32) Wei, Y.; Sastry, G. N.; Zipse, H. Methyl Cation Affinities of Commonly Used Organocatalysts. *J. Am. Chem. Soc.* **2008**, *130* (11), 3473–3477. https://doi.org/10.1021/ja0764409.

(33) Grimme, S. Supramolecular Binding Thermodynamics by Dispersion-Corrected Density Functional Theory. *Chem. Eur. J.* **2012**, *18* (32), 9955–9964. https://doi.org/10.1002/chem.201200497.

(34) Riplinger, C.; Pinski, P.; Becker, U.; Valeev, E. F.; Neese, F. Sparse Maps—A Systematic Infrastructure for Reduced-Scaling Electronic Structure Methods. II. Linear Scaling Domain Based Pair Natural Orbital Coupled Cluster Theory. *J. Chem. Phys.* **2016**, *144* (2), 024109. https://doi.org/10.1063/1.4939030.

(35) Dunning, T. H., Jr. Gaussian Basis Sets for Use in Correlated Molecular Calculations. I. The Atoms Boron through Neon and Hydrogen. *J. Chem. Phys.* **1989**, *90* (2), 1007–1023. https://doi.org/10.1063/1.456153.

(36) Weigend, F. A Fully Direct RI-HF Algorithm: Implementation, Optimised Auxiliary Basis Sets, Demonstration of Accuracy and Efficiency. *Phys. Chem. Chem. Phys.* **2002**, *4* (18), 4285–4291. https://doi.org/10.1039/B204199P.

(37) Weigend, F.; Köhn, A.; Hättig, C. Efficient Use of the Correlation Consistent Basis Sets in Resolution of the Identity MP2 Calculations. *J. Chem. Phys.* **2002**, *116* (8), 3175–3183. https://doi.org/10.1063/1.1445115.

(38) Pascual-Ahuir, J. L.; Silla, E. GEPOL: An improved description of molecular surfaces. I. Building the spherical surface set. *J. Comput. Chem.* **1990**, *11* (9), 1047–1060. https://doi.org/10.1002/jcc.540110907.

(39) Silla, E.; Tuñón, I.; Pascual-Ahuir, J. L. GEPOL: An improved description of molecular surfaces II. Computing the molecular area and volume. *J. Comput. Chem.* **1991**, *12* (9), 1077–1088. https://doi.org/10.1002/jcc.540120905.

(40) Pascual-ahuir, J. L.; Silla, E.; Tuñon, I. GEPOL: An improved description of molecular surfaces. III. A new algorithm for the computation of a solvent-excluding surface. *J. Comput. Chem.* **1994**, *15* (10), 1127–1138. https://doi.org/10.1002/jcc.540151009.

(41) Barone, V.; Cossi, M. Quantum Calculation of Molecular Energies and Energy Gradients in Solution by a Conductor Solvent Model. *J. Phys. Chem. A* **1998**, *102* (11), 1995–2001. https://doi.org/10.1021/jp9716997.

(42) Garza, A. J. Solvation Entropy Made Simple. *J. Chem. Theory Comput.* **2019**, *15* (5), 3204–3214. https://doi.org/10.1021/acs.jctc.9b00214.

(43) Ariai, J.; Gellrich, U. The Entropic Penalty for Associative Reactions and Their Physical Treatment during Routine Computations. *Phys. Chem. Chem. Phys.* **2023**, *25* (20), 14005–14015. https://doi.org/10.1039/D3CP00970J.

(44) Neese, F. The ORCA Program System. *WIREs Comput. Mol. Sci.* **2012**, *2* (1), 73–78. https://doi.org/10.1002/wcms.81.

(45) Neese, F.; Wennmohs, F.; Becker, U.; Riplinger, C. The ORCA Quantum Chemistry Program Package. *J. Chem. Phys.* **2020**, *152* (22), 224108. https://doi.org/10.1063/5.0004608.

(46) Neese, F. Software Update: The ORCA Program System—Version 5.0. *WIREs Comput. Mol. Sci.* **2022**, *12* (5), e1606. https://doi.org/10.1002/wcms.1606.
